# Centre‐based early education interventions for improving school readiness: A systematic review

**DOI:** 10.1002/cl2.1363

**Published:** 2023-12-13

**Authors:** Claire J. McCartan, Jennifer Roberts, Julie‐Ann Jordan

**Affiliations:** ^1^ IMPACT Research Centre, Northern Health and Social Care Trust Holywell Hospital Antrim UK; ^2^ School of Social Sciences, Education and Social Work Queen's University Belfast Belfast UK

## Abstract

**Background:**

Globally, children are legally obliged to attend school at a certain age (ranging from 4 to 7 years old). Developmental differences are rarely considered at school entry nor are they always reflected in the teaching and learning environment. Children who start school without being ready to cope may be significantly disadvantaged. Failure at school can impact directly on long‐term outcomes such as unemployment, crime, adolescent pregnancy, and psychological and physical morbidity in adulthood. In contrast, experiencing success at school can impact positively on a child's self esteem, behaviour, attitude, and future outcomes. School readiness interventions aim to prepare a child for the academic content of education and the psychosocial competencies considered important for learning such as self‐regulation, listening, following instructions and learning to share in play and other social settings. There is a need for evidence of the effectiveness of centre‐based school readiness interventions.

**Objectives:**

To evaluate the effectiveness of centre‐based interventions for improving school readiness in preschool children.

**Search Methods:**

In October 2021 we searched CENTRAL, MEDLINE, Embase, ERIC, PsycINFO, ERIC, eight additional databases and three trials registers. Other eligible studies were identified through handsearches of reference lists, reports, reviews and relevant websites.

**Selection Criteria:**

We included randomised controlled trials (RCTs) and quasi‐RCTs comparing centre‐based school readiness interventions to no intervention, wait‐list control or treatment as usual (TAU) for children (aged three to 7 years before starting compulsory education). The primary outcomes were school readiness and adverse effects.

**Data Collection and Analysis:**

We used standard methodological procedures expected by Cochrane. We used GRADE to assess the certainty of evidence.

**Main Results:**

We included data from 32 trials involving 16,899 children (6590 included in at least one meta‐analysis). Four studies compared centre‐based early education interventions with no treatment controls. Twenty‐two trials compared an enriched school curriculum to treatment as usual (TAU). Children were aged between 3 and 7 years old (mean age 4.4 years), 51.7% were boys and at least 70% were from a racial/ethnic minority group. Most studies were conducted in the USA and mainly located in areas of high socioeconomic deprivation. Interventions were delivered in centre‐based settings (pre‐kindergarten or elementary schools), for at least one half day, 4 days per week over the academic year. Follow‐up ranged from up to 1 year (short‐term), 1–2 years (medium‐term) and over 2 years (long‐term). We judged the certainty of evidence to be very low to moderate across all outcome measures. We downgraded the certainty of the evidence because the included studies were at an unclear or high risk of bias due to poor reporting, imprecision arising from small sample sizes and wide confidence intervals, and inconsistency due to statistical heterogeneity. Most studies were considered to be low or unclear risk for selection, detection, performance, attrition, selective reporting, and other bias. Allocation bias was at high risk in 10 studies. The US federal government funded most of the studies.

**Comparison 1. Centre‐based early education interventions for improving school readiness versus no intervention**

*Cognitive development*. There may be little to no difference in cognitive development between centre‐based early education interventions and no intervention at long‐term follow‐up (MD: 3.28, 95% CI: 0.23 to 6.34; *p* = 0.04; 2 studies, 361 participants; low certainty evidence).

*Emotional well‐being and social competence*. There may be no clear difference in social skills in centre‐based early education interventions compared to the no intervention control group at short‐term follow‐up (SMD: −0.11, 95% CI: −0.54 to 0.33; *p* = 0.63; 3 studies, 632 participants; low certainty evidence). Heterogeneity for this outcome was substantial (*I*² = 71%).

*Health development*. Narrative analysis from a single study showed that centre‐based early education interventions may improve health development outcomes such as health checks, immunisation compliance and dental care (1 study, 142 participants; low certainty evidence).

None of the studies reported on school readiness, adverse effects, or physical development.

**Comparison 2. Centre‐based early education interventions for improving school readiness versus TAU**

*School readiness*. The evidence is very uncertain about the effect of centre‐based early education interventions compared to TAU on school readiness up to 1 year post‐intervention (SMD: 1.17, 95% CI: −0.61 to 2.95; *p* = 0.20; 2 studies, 374 participants; very low certainty evidence). Heterogeneity for this outcome was considerable (*I*² = 95%).

*Cognitive development*. The evidence is very uncertain about the effect on cognitive development between centre‐based early education interventions and TAU at long‐term follow‐up (MD: 9.34, 95% CI: −6.64 to 25.32; *p* = 0.25; 2 studies, 136 participants; very low certainty evidence). Heterogeneity for this outcome was considerable (*I*² = 92%).

*Emotional well‐being and social competence*. A meta‐analysis of 12 studies demonstrated there may be little to no difference in social skills between centre‐based early education interventions and TAU at short‐term follow‐up (SMD: 0.11, 95% CI: −0.05 to 0.28; *p* = 0.19; 12 studies, 4806 participants; low certainty evidence).

*Physical development*. Evidence from one study showed that centre‐based early education interventions likely have little to no difference in increasing fine motor skills compared to TAU at short‐term follow‐up (MD: 0.80, 95% CI: −1.11 to 2.71; 1 study, 334 participants; moderate certainty evidence).

None of the studies measured adverse effects or health development.

**Authors' Conclusions:**

We found very low, low and moderate‐certainty evidence that centre‐based interventions convey little to no difference to children starting school compared to no intervention or TAU, up to 1 year. More research, measuring relevant outcomes, conducted outside the USA, is required to improve programmes designed to meet the needs of children starting school.

## 1 PLAIN LANGUAGE SUMMARY

### 1.1 Do centre‐based early education programmes prepare children for starting school?

#### 1.1.1 Key messages

Centre‐based interventions may have little to no effect on school readiness but we are very uncertain about the results.

Future studies should consider how best to measure school readiness, over a longer timeframe, and be conducted in countries other than the USA.

### 1.2 What is school readiness?

School readiness covers elements of a child's development that promote learning such as: positive behaviour; emotional well‐being; social skills; and intelligence. Interventions can foster play and sharing, listening skills and emotional regulation which all help contribute to a positive learning environment. Being ready for school could have life‐long benefits.

### 1.3 What did we want to find out?

We wanted to find out if attending a centre‐based early education programme was better than:

– not attending any programme;
– receiving a standard pre‐school programme; and
– if there were any unwanted effects.

### 1.4 What did we do?

We searched for studies that compared centre‐based early education programmes with no programme or a standard curriculum. We compared and summarised the results of the studies and rated our confidence in the evidence, based on factors such as study methods and sizes.

### 1.5 What did we find?

We found 32 studies involving 16,899 children aged 3 to 7 years old. Four studies compared centre‐based early education interventions with no treatment. Twenty‐two trials compared an enriched school curriculum to treatment as usual. The largest study was in 3726 children and the smallest study was in 59 children. Studies were conducted around the world, mostly in the USA (28 studies). Studies, on average, lasted for 12 months; the longest trial lasted 6 years. The US Federal Government funded most studies.

### 1.6 Main results

Centre‐based early education programmes for improving school readiness probably make little to no difference to children receiving either no programme or a standard curriculum. We found evidence for:

– school readiness (2 studies involving 374 children);
– cognitive development (4 studies involving 497 children);
– emotional well‐being and social competence (15 studies involving 5438 children);
– health development (1 study involving 142 children);
– physical development (1 study in 334 children).

We found no studies to help answer our question on adverse effects.

### 1.7 What are the limitations of the evidence?

We have very low, low and moderate confidence in the evidence for a number of reasons:

– it is possible that people in the studies were aware of which treatment they were getting;
– the studies used different ways of delivering the intervention;
– not all of the studies provided data about everything that we were interested in;
– the evidence focused on specific academic outcomes whereas the question we wanted to answer was broader; and
– there are not enough studies to be certain about the results of our outcomes.

### 1.8 How up to date is this evidence?

The evidence is up to date to October 2021.

## 2 SUMMARY OF FINDINGS

### 2.1 Summary of findings 1

**Centre‐based early education interventions for improving school readiness compared to no treatment control**

**Table:** 

**Centre‐based early education interventions for improving school readiness compared to no treatment control**						
**Patient or population**: improving school readiness in preschool children **Setting**: centre‐based early education settings for children aged 3 to 7 years before starting compulsory education **Intervention**: centre‐based early education intervention for improving school readiness **Comparison**: no treatment control						
**Outcomes**	**Risk with no treatment control**	**Risk with school readiness intervention**	**Relative effect (95% CI)**	№ of participants **(studies)**	**Certainty of the evidence (GRADE)**	**Comments**
**School readiness** (not measured)	‐	‐	‐	‐	‐	‐
**Adverse effects** (not measured)	‐	‐	‐	‐	‐	‐
Cognitive development Assessed with: Stanford‐Binet Intelligence Scale (high score favourable, upper & lower limits: 160–80) Follow‐up: 2 + years	The mean score for the no treatment control group was **88.73 points**	The mean score in the treatment group was on average **3.28 pointshigher** (0.23 higher to 6.34 higher)	‐	361 (2 RCTs)	⊕⊕⊝⊝ **Low** ^**a,b**^	‐
Emotional well‐being & social competence (social skills) Assessed with: Social Skills & Positive Approaches to Learning (SSPAL; Zill, 2003b); (SSiS; Flowers, 2008); Ypsilanti Rating Scale (Kamii, 1967) Follow‐up: 1 year	See comment	The SMD was **0.11 standard deviations** lower in the treatment group (0.54 lower to 0.33 higher)	‐	632 (3 RCTs)	⊕⊕⊝⊝ **Low** ^**b,c**^	SMD of 0.11 represents a small effect size (Cohen, 1988)
**Physical development (not measured)**	‐	‐	‐	‐	‐	‐
Health development Follow‐up: 1 year	Children in the intervention group were more likely to have received a health check, be up‐to‐date with their immunisations and have had a dental examination and other health screenings in the previous 12 months compared to the wait‐list control.			142 1 (RCT)	⊕⊕⊝⊝ **Low** ^**a,b**^	‐
*The risk in the intervention group (and its 95% CI) is based on the assumed risk in the comparison group and the **relative effect** of the intervention (and its 95% CI). CI, confidence interval; MD, mean difference; SD, standard deviation; SMD, standardised mean difference						
GRADE Working Group grades of evidence **High certainty**: we are very confident that the true effect lies close to that of the estimate of the effect. **Moderate certainty**: we are moderately confident in the effect estimate: the true effect is likely to be close to the estimate of the effect, but there is a possibility that it is substantially different. **Low certainty**: our confidence in the effect estimate is limited: the true effect may be substantially different from the estimate of the effect. **Very low certainty**: we have very little confidence in the effect estimate: the true effect is likely to be substantially different from the estimate of effect.						

^a^Certainty of the evidence downgraded one level because the risk of bias was unclear or high risk in at least one of the included studies. ^b^Certainty of the evidence downgraded one level for imprecision because the optimal information size was <400 or the CIs included no effect, or both. ^c^Certainty of the evidence downgraded one level for inconsistency due to unexplained heterogeneity (90+%).

### 2.2 Summary of findings 2

**Centre‐based early education interventions for improving school readiness compared to treatment as usual**

**Table:** 

**Centre‐based early education interventions for improving school readiness compared to treatment as usual**						
**Patient or population**: improving school readiness **Setting**: centre‐based early education settings for children aged 3 to 7 years before starting compulsory education **Intervention**: centre‐based early education intervention for improving school readiness **Comparison**: treatment as usual						
**Outcomes**	**Risk with treatment as usual**	**Risk with school readiness intervention**	Relative effect **(95% CI)**	№ of participants **(studies)**	Certainty of the evidence **(GRADE)**	**Comments**
School readiness Assessed with: Kindergarten Readiness Test (Larson, 1988); International Development and Early Learning Assessment (IDELA; Save the Children, 2015) Follow‐up: 1 year	See comment	The SMD was **1.17 standard deviations higher** in the treatment group (0.61 lower to 2.95 higher)	‐	374 (2 RCTs)	⊕⊝⊝⊝ **Very low** ^**a,b,c**^	An SMD of 1.17 represents a large effect size (Cohen, 1988)
**Adverse effects** (not measured)	‐	‐	‐	‐	‐	‐
Cognitive development Assessed with: Stanford‐Binet Intelligence Scale (Terman, 1960) Follow‐up: 2 + years	The mean score for no treatment control group was **90.86 points**	The mean score in the treatment group was on average **9.34 points higher** (0.64 lower to 25.32 higher)	‐	136 (2 RCTs)	⊕⊝⊝⊝ **Very low** ^**a,b,c**^	‐
Emotional well‐being & social competence (social skills) Assessed with: Social Competence Scale, Teacher Rating (Fast Track, 2019); Cooper‐Farran Behaviour Rating Scales, Interpersonal Skills (Cooper, 1991); Teacher Behaviour Rating Scale (TBRS; Landry, 2000); citizenship grades; International Development & Early Learning Assessment, Socioemotional subtest (IDELA; Save the Children, 2015); Social Skills Rating System, Social Skills (SSRS; Gresham, 1990) Follow‐up: 1 year	See comment	The SMD was **0.11 standard deviations higher** in the treatment group (0.05 lower to 0.28 higher)	‐	4806 (12 RCTs)	⊕⊕⊝⊝ **Low** ^**a,c**^	An SMD of 0.11 represents a small effect size (Cohen, 1988)
Physical development (fine motor skills) Assessed with: International Development & Early Learning Assessment, Fine Motor subtest (IDELA; Save the Children, 2015) (Scores are calculated by the average percent correct for the tasks in each domain, higher scores are favourable) Follow‐up: 1 year	The mean score for no treatment control group was **6.6 points**	The mean score in the treatment group was on average **0.80 points higher** (1.11 lower to 2.71 higher)	‐	334 (1 RCT)	⊕⊕⊕⊝ **Moderate** ^**b**^	‐
**Health development** (not measured)	‐	‐	‐	‐	‐	‐
*The risk in the intervention group (and its 95% CI) is based on the assumed risk in the comparison group and the **relative effect** of the intervention (and its 95% CI). CI, confidence interval; MD, mean difference; RCT, randomised controlled trial; SD, standard deviation; SMD, standardised mean difference						
GRADE Working Group grades of evidence **High certainty**: we are very confident that the true effect lies close to that of the estimate of the effect. **Moderate certainty**: we are moderately confident in the effect estimate: the true effect is likely to be close to the estimate of the effect, but there is a possibility that it is substantially different. **Low certainty**: our confidence in the effect estimate is limited: the true effect may be substantially different from the estimate of the effect. **Very low certainty**: we have very little confidence in the effect estimate: the true effect is likely to be substantially different from the estimate of effect.						

^a^Certainty of the evidence downgraded one level because the risk of bias was unclear or high risk in at least one of the included studies. ^b^Certainty of the evidence downgraded one level for imprecision because the optimal information size was <400 and/or the CIs included both appreciable benefit and harm (SMD ± 0.05). ^c^Certainty of the evidence downgraded one level for inconsistency due to unexplained heterogeneity (90+%).

## 3 BACKGROUND

### 3.1 Description of the condition

In many parts of the world, children are legally obliged to attend school at a particular age. The age at which compulsory education begins can range between 4 and 7 years, depending on location. Developmental differences between children are rarely taken into consideration when setting the age of school entry, nor are they always reflected in the teaching and learning environment within mainstream education. Children who start school without being ready to cope with the requirements of formal education may be significantly disadvantaged (Fink, 2019; Ghandour, 2019; Pettinger, 2020; Sawhill, 2012) and early introduction to formal learning and the ‘schoolification’ (Grieshaber, 2018; Husa, 2005; Kamerman, 2005) of early childhood education can have negative consequences for a child's holistic development and well‐being (Bradbury, 2019; Elkind, 2001; Gunnarsdottir, 2014). Analysis of the Terman Life Cycle Study found that starting school early was associated with lower educational attainment, worse midlife adjustment and increased mortality risk (Kern, 2009). Other studies have found associations between earlier starting school age and higher rates of adolescent marriage and pregnancy (Nguyen, 2020), lower rates of college admission and adult earnings (Matta, 2016). There is some evidence that delaying formal schooling until 6 or 7 years of age (as in Finland, for example) may confer benefits; children perform better in school both in terms of academic attainment and behaviour when they have a higher school starting age (Cook, 2016; Dee, 2018; Dhuey, 2019). Datar (2006) found that delaying school starting age by 1 year significantly boosted test scores when children started formal education and analysis of Danish administrative data found that a 1 year delay in kindergarten entry dramatically reduced inattention/hyperactivity at age 7 (Dee, 2018). Analysis of university entrance examination data from Brazil found that 1 year delayed entry improved probability of college admission based on aptitude test scores and higher earnings once entering the labour market (Matta, 2016). Analysis of the UK National Child Development Study found that test scores at age seven were significant predictors of adult outcomes in educational attainment and the labour market at age 23 (Connolly, 1992) and 33 (Harmon, 1988; Robertson, 1996); those scoring in the lowest quartile at age seven earned on average 20% less than the rest of the sample (Currie, 2001).

The benefits of delaying kindergarten entrance are significantly larger for ‘at‐risk’ children, for example: children living in poverty; children with a disability; children of mothers with low educational attainment; children in lone parent families; or children who have English as a second language (Datar, 2006; Duncan, 1997; Lee, 2002; Lipina, 2009; Peterson, 2018; Zill, 1998). This may be because starting compulsory education later maximises the likelihood that children are developmentally ‘ready’ for school, or be explained by exposure to other preschool activities that facilitate school readiness, or both. In low‐ and middle‐income countries, increasing emphasis and priority is being placed on access to quality preschool education in international development policy (UNICEF, 2012); children in these economies face multiple disadvantages and increasing school enrolment, improving academic achievement and reducing school drop‐out are considered key to providing a route out of poverty.

#### 3.1.1 School readiness

School readiness is recognised as a composite of the readiness of an individual child and that of the environment into which s/he enters when starting school (Williams, 2019).

School readiness most often refers to a child's readiness for formal learning in a school setting. It is a multi‐dimensional concept that encompasses the behavioural, emotional and cognitive aspects of a child's development, alongside his or her adaptation to the classroom environment and being ‘ready to learn’ (National Education Goals Panel, 1991; UNICEF, 2012). Children who struggle in school include those who are academically (or cognitively) not able to cope, who have problems with communication or social skills, who are unable to follow directions, and who find it difficult to work on their own (poor concentration) or in groups (turn taking, collaboration) (see Chow, 2018; Diekstra, 2008; Durlak, 2011; Harrington, 2020). Children who start formal education ‘school ready’ are much more likely to learn, stay on in school and achieve educational and employment goals (CGECCD, 2008; Nonoyama‐Tarumi, 2009; Save the Children, 2004; Stith, 2003).

There is some debate around the precise definition of school readiness and how it should be assessed (Aiona, 2005). One view is that children are ready for school once they reach a certain age, others specify school readiness as a range of skills and competencies that a child is taught at home or in a childcare environment. Another view assesses readiness on multiple factors of the child's family (the family context and home environment), community (the level of resources and support made available to families with young children), services (extent of quality, accessibility, and affordability of programmes available locally to support families with young children), and early learning centres/schools (aspects such as school attainment levels and class sizes, which indicate the quality of education available).

For the purposes of this review, we defined school readiness in terms of the five domains set out by the National Education Goals Panel (National Education Goals Panel, 1997):

- *Physical development and health*—this incorporates a child's health, background, status, growth, and disability. The development of motor skills is also essential to school readiness, from the gross motor skills required in physical play and development to the fine motor skills used for writing and drawing.
- *Social and emotional development*—this involves a child's ability to interact with others and their capacity for self regulation. It encompasses children's self perception and their ability to understand other people's feelings, and interpret and communicate their own feelings.
- *Approaches to learning*—this refers to a child's attributes to apply their skills and knowledge, for example, curiosity, creativity, independence, co‐operativeness, and persistence.
- *Language and literacy*—this refers to a child's engagement with language in both written and oral forms.
- *Cognition and general knowledge*—conducting play‐oriented, exploratory activities that stimulate knowledge. It includes thinking and problem‐solving as well as developing knowledge about particular objects and how the world works. Mathematical knowledge, abstract thought, and imagination are included in this domain.

#### 3.1.2 Size of the problem

Research has estimated that 10% to 20% of school‐enroled children display emotional and behavioural barriers to learning significant enough to warrant formal intervention (Sugai, 2000). This figure rises to 30% to 50% in neighbourhoods with high levels of deprivation (Adelman, 2008). Analysis of the Millennium Cohort Study found that UK children from low‐ to middle‐income families were 5 months behind children from high‐income families in terms of vocabulary skills and had more behaviour problems (Washbrook, 2011). For those children living in poverty, persistent achievement gaps by social class can be identified as early as nursery stage, suggesting that the problem must be tackled before school (Brooks‐Gunn, 1997; Coley, 2002; Grantham‐McGregor, 2007; Lee, 2002; Walker, 2007; West, 2000).

#### 3.1.3 Consequences for children not ready for school

Success at school can impact positively on a child's self esteem, behaviour, attitude, and future success (Muenks, 2018; Pianta, 1996); failure at school can impact directly on long‐term outcomes such as unemployment, crime, teenage pregnancy, and psychological and physical morbidity in adulthood (Hertzman, 1996), and perpetuates the cycle of disadvantage. Children who start school with problems that interfere with their ability to settle, enjoy school, and learn are therefore significantly disadvantaged. Negative and antisocial behaviour is often related to poor academic performance, and for those experiencing emotional difficulties and family disruption, school drop‐out, academic failure and discipline problems at school are very much a risk (Alexander, 2001; Kutash, 2006; Loeber, 2000).

### 3.2 Description of the intervention

A range of different interventions have been developed to promote school readiness in young children across the globe, in low‐, middle‐, and high‐income economies. Most focus on preparing the child for the academic content of education (Jenkins, 2018), with a particular focus on literacy and numeracy, but many also concentrate on developing the psychosocial competencies important for learning, including self regulation, sitting still, listening, following instructions, and taking turns in conversation and play (Bodrova, 2019). As indicated above, emphasis is also placed on the readiness of the home, the school/early education setting, the community (i.e., the resources and support available) and the services available to a family with young children (Christensen, 2020). Parents and other caregivers have a profound impact on a child's learning, with diet, sleep, stress, and attachment all exerting an influence on a child's ability to develop and learn. Hence, some school readiness interventions include primary and community health care, parenting advice, and social services support to help parents with accessing benefits, job seeking, and health care advice, including nutrition and parenting skills (Nix, 2018). The US Head Start Programme, for example, offers family‐based interventions for at‐risk children that include targeted support for their mothers, such as mental health services, substance abuse counselling, employment assistance, housing assistance and continuing education (Lacy, 1997). Programmes vary in duration and intensity but often involve two or more part‐time sessions per week over a 12‐week or longer period in the months before a child starts school. Interventions are often targeted at low‐income families and those who do not speak English as their first language as they tend to be less ready for school. There are also specific programmes tailored for children with special needs.

Interventions, which may be provided via nationally funded programmes for preschool children, such as Head Start, vary in the range of educational, health, nutritional, and social services they offer, and in the teaching methods and curricula they provide; they may be tailored to the individual child. Interventions may focus on one or more of the following domains.

**Physical development and health**: many of the programmes also place emphasis on supporting parents to help their children. These schemes endorse positive discipline, promoting learning and developing by encouraging parents to work with their children, encouraging home reading, and reinforcing what is learned in the early education setting.

**Social and emotional development**: developing prosocial friendship skills, emotional understanding and expression, self control, and social problem‐solving skills. Play underpins many of the teaching strategies, and through the provision of appropriate indoor and outdoor play environments, children can learn about setting rules and consequences, explore and develop their sense of the world, communicate with others as they problem solve, take risks and make mistakes, and think creatively and imaginatively. Books are also used to explore difficult issues such as bullying or domestic violence.

**Language and literacy**: developing key pre‐literacy skills is embedded in many of the interventions. These help children develop their vocabulary and communication skills, and phonological awareness, and an understanding of print conveying meaning and letters creating a code of language. Strategies can include interactive reading programmes that encourage children to ask questions, discuss and retell stories or predict story endings, and require early education centre staff delivering the intervention to engage active listening, language expansion, and de‐contextualised talk. Rhymes and songs with mime and gesture are used to support language development. Children are encouraged to practise letter shapes and early writing skills in painting and drawing to develop their fine motor skills as a precursor to independent writing. Shared or paired reading is also used to enhance language and literacy skills, and promote an appreciation of books. These strategies are all used to develop pre‐reading and pre‐writing skills.

**Approaches to learning**: children are encouraged to explore new experiences to develop their curiosity and confidence in trying new things. In Maths and Science activities, they are encouraged to ask questions, form hypotheses or make guesses. Children are encouraged to read and write stories, and change or make up their own endings. Games including ‘I Spy’ can be used to extend natural curiosity. These kinds of activities are used to help children develop problem‐solving skills, apply persistence to achieve an outcome, and use their initiative to develop their independence. Creative play using role play and props and materials is also a method used to develop these competencies.

**Cognition and general knowledge**: mathematical concepts are introduced through play, with the use of mathematical vocabulary to describe everyday objects and positions. Story time and circle time is used to help relate informal mathematical knowledge to more formal mathematical concepts.

**Environmental readiness**: environmental factors can help support children's transition to school. This growing emphasis on the importance of environmental readiness reflects, in part, the needs of the growing number of children with working mothers and experiencing childcare outside the home and in childcare centres. Families with small children also need to have access to appropriate health care, affordable quality childcare, and to live in safe neighbourhoods. Many of the interventions reference classroom organisation and structure, which directs different types of learning through play in a variety of locations in the classroom. There is an emphasis on stimulating resources and equipment, including building blocks, art and science materials, books, and computer software.

### 3.3 How the intervention might work

Essentially, school readiness programmes seek to mitigate the risk factors associated with children facing poverty and disadvantage through the nurture and development of key skills and competencies required for formal learning, and by attempting to reduce the achievement gap that is already present once children start school. Using Head Start as an example (Head Start Resource Centre, 2011), and taking each of the above domains in turn, school readiness interventions seek to do the following.

- Ensure children are socially and emotionally ready, and able to:
  ∘ engage and maintain positive adult‐child relationships and interactions;
  ∘ maintain positive peer relations;
  ∘ display attention, emotional regulation, and appropriate classroom behaviour;
  ∘ follow rules; and
  ∘ develop a sense of self, self confidence, and identity.
- Ensure children have or develop adequate language and literacy skills, and are able to:
  ∘ build and use increasingly complex vocabulary;
  ∘ use language for conversation and communication; and
  ∘ engage with literature.
- Promote a positive approach to learning, such that children:
  ∘ show interest in varied topics and activities; and
  ∘ persist when working.
- Ensure children have or develop adequate cognitive skills and general knowledge, so that they are able to:
  ∘ use mathematics regularly; and
  ∘ ask questions, make predictions, develop hypotheses to gain understanding of their environment.
- Ensure that children are physically well, and able to:
  ∘ be healthy and safe;
  ∘ use large muscles to control movement, balance, etc.; and
  ∘ use fine motor skills.
- Provide a ‘ready environment’ such that:
  ∘ systems of early care and education are available to families to secure appropriate care and support services;
  ∘ schools recognise that each child has unique learning needs, and provide age‐appropriate and developmentally relevant early education learning environments, linked to other children's services;
  ∘ families are economically stable and parents are well informed about bringing up their children; and
  ∘ families have access to community‐based health care, including harm prevention, and the promotion of safe neighbourhoods and supportive communities.

#### 3.3.1 Immigrant children

It is also important to note that a number of school readiness programmes have been developed to target specific populations. Children from immigrant families often face multiple disadvantage: poverty, poorer mental and physical health, lower verbal interaction and shared literacy experiences at home, discrimination and access to poorer quality education (Brooks‐Gunn, 2005; Schofield, 2006; Waters, 2005; Yoshikawza, 2011). Specifically tailored interventions have been used to meet the needs of this group, which can include multi‐lingual approaches to teaching and culture‐specific classroom resources; sensitivity to discrimination by peers and educators; engaging parents in programme and curricular development; parent counselling; and gateway service provision for accessing health and social services.

Intervention programmes may differ in their emphasis or in the combination of factors they address. The focus of this review will be programmes that include literacy and numeracy skills along with a focus on social and emotional learning.

### 3.4 Why it is important to do this review

Failure at school can have a significant and lifelong impact on the social and physical well‐being of an individual, which can impact on future generations (Woodhead, 1985). Evidence suggests that school readiness is an important independent factor and predictor of future academic achievement (Morgan, 2016), however the role that poverty and income inequality plays in determining a child's long‐term educational and employment outcomes cannot be underestimated (Magnuson, 2009) particularly as the inequality gap continues to grow (Reardon, 2011). In recognition of the central role of deprivation, additional investment in state‐funded prekindergarten has lead to a two‐fold increase in HeadStart enrolments in the US between 2002 and 2014, rising from 14% of 4 year olds to 29% in 2014 (Office of Head Start, 2021). The COVID‐19 pandemic has had a hugely negative impact with estimates of 20 years of education gains lost as a result of global crisis (United Nations, 2021). Austerity measures in the UK has seen early years funding reduced (Stewart, 2015) and it is therefore important to review the evidence of its effectiveness. The economic and social investment return in early childhood education programmes is greater than other governmental human capital development programmes (UNICEF, 2012) and a worthwhile investment (Magnuson, 2016); however, worldwide only 50% of children have access to preschool education and in low‐income countries, this drops to 20% (World Bank, 2021). International evidence has estimated a 20% to 30% loss in income in countries where investment in preschool programmes is minimal (Grantham‐McGregor, 2007; Handa, 2008). School readiness is an integral part of the work towards universal access to basic quality education as set out in the UN's Sustainable Development Goals (United Nations, 2021), UNESCO's Millennium Development Goals (United Nations, 2000), Education for All (UNESCO, 2007; World Education Forum, 2000) and World Fit for Children (UNICEF, 2003). A systematic review (Petrosino, 2012) found that interventions aimed at improving school enrolment in developing countries were having a positive impact; children who are ‘ready to learn’ are more likely to stay on in school once enroled (UNICEF, 2012).

A number of systematic reviews have been conducted in aspects of early education. Miller and colleagues completed a systematic review on home‐based child development interventions for preschool children from socially disadvantaged families (Miller, 2012) and Mariano (2019) systematically reviewed the literature on factors associated with school readiness and later school‐age achievement. Another review of early childhood education programmes examined interventions targeting children experiencing poverty (Chambers, 2010), and meta‐analyses conducted by Camilli and colleagues (Camilli, 2010) and Darrow (Darrow, 2009) concentrated on literacy and cognitive‐focused interventions. However, there is no current evidence synthesis of the available evidence of school readiness interventions.

## 4 OBJECTIVES

To evaluate the effectiveness of centre‐based interventions for improving school readiness in preschool children.

## 5 METHODS

### 5.1 Criteria for considering studies for this review

#### 5.1.1 Types of studies

We included randomised controlled trials (RCTs) and quasi‐RCTs (i.e., trials where a quasi‐random method of allocation is used, such as alternation or date of birth), randomised at the individual or cluster level.

#### 5.1.2 Types of participants

Children aged 3 to 7 years before starting compulsory education.

#### 5.1.3 Types of interventions

We included centre‐based programmes delivering a school readiness intervention, compared with no intervention control, wait‐list control or treatment as usual (e.g., centres that do not have a school readiness programme or home‐based intervention). Centres were defined as organisations that offered onsite early education provision; for example, preschool, nursery unit, kindergarten, or registered childcare facility. Interventions/programmes had to target cognitive, pre‐reading/reading or pre‐writing/writing, and/or mathematical skills development as well as the prosocial behaviours associated with school readiness, for example, social‐emotional development, approaches to learning, physical well‐being, and creating a ready environment. Programmes had to provide educational services directly to the children, with or without parental involvement, lasting for at least 10 h per week for 2 months. We excluded: 1. educational interventions delivered only through home visits or in family childcare settings; 2. educational interventions provided solely to the parents; 3. programmes that specifically target children with special needs.

Comparators eligible for inclusion were no intervention control settings including wait‐list controls or children staying at home without any structured preschool education. Comparisons of interventions (typically an enriched school readiness curriculum) and treatment as usual settings (described either as non‐specific/generic curricula) were also eligible for inclusion. Enriched school curriculum typically offer enhanced additional learning opportunities through incorporating additional classroom resources and materials and may also include curriculum add‐ons such as teacher and parent training. Activities including shared reading, play‐based learning and use of the arts, music and science are promoted. The criteria for considering studies is detailed in the published protocol (Macdonald, 2014).

#### 5.1.4 Types of outcome measures

Where data were available, outcomes were reported as short term (up to 1 year), medium term (between 1 year and 2 years) and long term (over 2 years). If more than one outcome was used to measure the same competency in a study, we selected the most robust measure (validated) and/or most frequently used across included studies.

##### Primary outcomes

- School readiness*, as measured by scales such as the Bracken Basic Concepts Scale Revised (BBCS‐R; Bracken, 1998); Brigance Diagnostic Inventory of Early Development (Brigance, 1992; Glascoe, 1995); Developmental Indicators for the Assessment of Learning (DIAL‐R; Mardell‐Czudnowski, 1998); Early Development Instrument (Janus, 2007); Gesell School Readiness Test (GSRT; Haines, 1980).
- Adverse effects* (e.g., child anxiety, disengagement from education, school anxiety, lower educational attainment).

##### Secondary outcomes

###### Child outcomes

- Academic achievement (as measured by academic achievement test scores such as pre‐reading/reading, vocabulary, oral comprehension, phonological awareness, pre‐writing/writing, verbal skills and mathematics [e.g., the Vocabulary Subtest of the Stanford‐Binet Intelligence Test (Roid, 2003a, 2003b; Terman, 1960; Thorndike, 1986), Peabody Picture Vocabulary Test (PPVT; Dunn, 2007), the Wechsler Individual Achievement Test (Wechsler, 2002)]).
- Cognitive development* as measured by, for example, the Wechsler Intelligence Scale for Children (WISC‐IV; Wechsler, 2003), Non‐Reading Intelligence Tests (Young, 1989), the Expressive One‐Word Picture Vocabulary Test (EOWPVT; Brownell, 2000), Dimensional Change Card Sort (DCCS; Zelazo, 2006), parent/teacher rating of cognitive development (grade ratings, identification/placement in special needs programmes, etc.).
- Emotional well‐being and social competence* (as measured by behavioural assessments of social interaction, problem behaviours, social skills and competencies, child‐parent relationship/child‐teacher relationship).
- Health development* (as measured by access to health care and health status).
- Physical development*.

###### Adverse outcomes

- Parent stress

###### Economic costs

- Cost data: unit of costs of programme, costs per child

*We included all items marked with an asterix in the Summary of findings Table 1; Summary of findings Table 2.

**Table 1:** Cluster‐RCTs.

Study	Adjustment for clustering
Atteberry (2019)	N/A narrative analysis only
Barnes (2016)	Review authors adjusted for clustering using an ICC of 0.12
Barnett (2008)	N/A narrative analysis only
Bierman (2008)	Review authors adjusted for clustering using an ICC of 0.12
Blair (2014)	Review authors adjusted for clustering using an ICC of 0.12
Castro (2017)	N/A narrative analysis only
Coffman (1967)	N/A narrative analysis only
Courtier (2021)	Review authors adjusted for clustering using an ICC of 0.12
Diamond (2019)	N/A narrative analysis only
Farran (2015)	Review authors adjusted for clustering using an ICC of 0.12
Hsueh (2014)	Review authors adjusted for clustering using an ICC of 0.12
Lipsey (2009)	N/A narrative analysis only
Lonigan (2015)	Review authors adjusted for clustering using an ICC of 0.12
PCER (2008), Bright Beginnings & Creative Curriculum	Review authors adjusted for clustering using an ICC of 0.12
PCER (2008), Creative Curriculm	Review authors adjusted for clustering using an ICC of 0.12
PCER (2008), Doors to Discovery & Let's Begin	Review authors adjusted for clustering using an ICC of 0.12
PCER (2008), Language‐Focused Curriculum	Review authors adjusted for clustering using an ICC of 0.12
PCER (2008), Literacy Express + DLM + OCR Pre‐K	Review authors adjusted for clustering using an ICC of 0.12
PCER (2008), Project Construct	Review authors adjusted for clustering using an ICC of 0.12
PCER (2008), Ready, Set, Leap!	Review authors adjusted for clustering using an ICC of 0.12
Peisner‐Feinberg (2019)	Review authors adjusted for clustering using an ICC of 0.12
Puma (2005)	N/A narrative analysis only
Raver (2009)	Review authors adjusted for clustering using an ICC of 0.12
Soloman (2018)	N/A narrative analysis only
Weikart (1967)	Review authors adjusted for clustering using an ICC of 0.12
Yousafzai (2018)	Study authors had adjusted for clustering using an ICC of 0.14

*Note*: Review authors derived a design effect using the average cluster size and ICC (0.12) (design effect = 1 + (M − 1) × ICC (0.12)). The standard errors were then multiplied by the square root of the design effect to produce inflated standard errors.

Abbreviations: DLM, Dynamic Learning Maps; ICC, intra‐cluster correlation; M, mean; N/A, Not applicable; ORC, open court reading; PCER, Preschool Curriculum Evaluation Research Initiative; Pre‐K, pre‐kindergarten; RCTs, randomised controlled trials.

**Table 2:** Comparison 1. Sensitivity analyses.

Outcome	Follow‐up	Adjustment for clustering	Studies (N)	Effect estimate (MD) (95% CI)
***Academic achievement***				
Mathematics	Long term	None	2 (326)	0.25 (0.01 to 0.50)
		0.07	2 (326)	0.25 (−0.01 to 0.50)
		0.17	2 (326)	0.24 (−0.02 to 0.50)
Reading	Long term	None	2 (348)	0.11 (−0.11 to 0.34)
		0.07	2 (348)	0.09 (−0.14 to 0.31)
		0.17	2 (348)	0.07 (−0.17 to 0.31)
Vocabulary	Short term	None	3 (1313)	0.24 (0.11 to 0.37)
		0.07	3 (1313)	0.23 (0.10 to 0.37)
		0.17	3 (1313)	0.23 (0.09 to 0.36)
	Medium term	None	2 (779)	0.41 (0.26 to 0.55)
		0.07	2 (779)	0.42 (0.27 to 0.57)
		0.17	2 (779)	0.42 (0.27 to 0.58)
	Long term	None	2 (364)	0.29 (−0.08 to 0.66)
		0.07	2 (364)	0.32 (−0.03 to 0.68)
		0.17	2 (364)	0.37 (0.07 to 0.68)
***Cognitive development***				
Intelligence	Short term	None	2 (702)	0.27 (0.10 to 0.44)
		0.07	2 (702)	0.27 (0.10 to 0.45)
		0.17	2 (702)	0.28 (0.10 to 0.46)
	Medium term	None	2 (675)	0.38 (−0.07 to 0.83)
		0.07	2 (675)	0.41 (−0.03 to 0.84)
		0.17	2 (675)	0.45 (0.04 to 0.86)
	Long term	None	2 (361)	0.22 (0.01 to 0.43)
		0.07	2 (361)	0.24 (0.02 to 0.47)
		0.17	2 (361)	0.26 (0.02 to 0.49)
***Emotional well‐being & social competence***				
Problem behaviours	Short term	None	3 (632)	−0.14 (−0.35 to 0.08)
		0.07	3 (632)	−0.14 (−0.35 to 0.08)
		0.17	3 (632)	−0.14 (−0.35 to 0.08)
Social skills	Short term	None	3 (632)	−0.09 (−0.46 to 0.28)
		0.07	3 (632)	−0.10 (−0.51 to 0.31)
		0.17	3 (632)	−0.11 (−0.56 to 0.34)

*Note*: Statistical method used: MD (IV, Random, 95% CI).

Abbreviations: CI, confidence intervals; IV, inverse variance; MD, mean difference; *N*, number of participants.

### 5.2 Search methods for identification of studies

We ran the first searches between February and September 2014 using the strategies in Supporting Information: Appendix 1. We used an RCTs filter where appropriate and did not apply any date or language restrictions. When we re‐ran the searches in 2020, we had access to a different set of databases and database platforms and found that the original strategies retrieved an unmanageable number of records. The DPLPG information specialist revised the search strategies to increase the precision, making sure that any included studies from 2014 were captured by the new strategies. Additional search terms for a number of named intervention programmes identified from the first set of searches were included in the new strategies (Supporting Information: Appendix 2). We ran the revised searches in March 2020 from database inception onwards, and ran top‐up searches in October 2021.

#### 5.2.1 Electronic searches

We searched the following electronic databases up to October 2021. Changes to the sources searched over the course of this review are reported here Differences between protocol and review.

- Cochrane Central Register of Controlled Trials (CENTRAL; 2021 Issue 10), in the Cochrane Library, and which includes the Cochrane Developmental, Psychosocial and Learning Problems Specialised Register (searched 11 October 2021).
- MEDLINE Ovid (1946 to September Week 5 2021).
- Embase Ovid (1974 to 2021 October 8).
- PsycINFO Ovid (1806 to October Week 1 2021).
- Sociological Abstracts ProQuest (1952 to 11 March 2014).
- ERIC Proquest (1966 to 9 April 2014).
- ERIC EBSCOhost (1966 to 11 October 2021).
- British Education Index ProQuest (1975 to 10 March 2014).
- British Education Index EBSCOhost (1975 to 12 October 2021).
- Australian Education Index ProQuest (all available years searched 10 March 2014).
- Social Sciences Citation Index Web of Science, Clarivate (1970 to 12 October 2021).
- Conference Proceedings Citation Index—Social Science & Humanities Web of Science, Clarivate (1990 to 12 October 2021)
- *Cochrane Database of Systematic Reviews* (2021, Issue 10) in the Cochrane Library (searched 11 October 2021).
- Database of Abstracts of Reviews of Effects (DARE), in the Cochrane Library (searched 9 April 2014).
- EPISTEMONIKOS (www.epistemonikos.org; searched 12 October 2021).
- Campbell Collaboration Library (www.campbellcollaboration.org/better-evidence; searched 12 October 2021).
- EPPI‐Centre Evidence Library (eppi.ioe.ac.uk/cms/Default.aspx?tabid=56; searched 12 October 2021).
- EPPI‐Centre Database of Education Research (eppi.ioe.ac.uk/webdatabases/Intro.aspx?ID=6; searched 24 March 2014).
- WorldCat (www.worldcat.org; searched 25 March 2014).
- Networked Digital Library of Theses and Dissertations (NDLTD; www.ndltd.org; searched 25 March 2014).
- Digitala Vetenskapliga Arkivet (DIVA) (www.diva-portal.org; searched 24 March 2014).
- Trove Theses (trove.nla.gov.au; searched 25 March 2014).
- Theses Canada (www.bac-lac.gc.ca/eng/services/theses/Pages/theses-canada.aspx; searched 25 March 2014).
- National Academic Research and Collaborations Information System (NARCIS) (www.narcis.nl; searched 25 March 2014).
- ProQuest Open Access Dissertations & Theses (https://www.proquest.com/?defaultdiss=true; searched 8 October 2014).
- DART‐Europe E‐theses Portal (www.dart-europe.org/basic-search.php; searched 25 March 2014).
- ProQuest Dissertations & Theses Global (searched 12 October 2021).
- *meta*Register of Controlled Trials (*m*RCT; www.controlled-trials.com/mrct/; searched 7 May 2014)
- WHO International Clinical Trials Registry Platform (trialsearch.who.int/; searched 12 October 2021).
- ClinicalTrials.gov (clinicaltrials.gov; searched 12 October 2021).
- ISRCTN registry (www.isrctn.com/; searched 12 October 2021).

#### 5.2.2 Searching other resources

We examined the reference lists of relevant studies and reviews to identify further studies up to October 2021. We compiled a list of all these studies and asked experts in the field to forward any published or unpublished studies that we missed in oversight. We also searched the following websites of relevant organisations and government departments using the keywords in Supporting Information: Appendix 3: the What Works Clearinghouse (WWC; ies.ed.gov/ncee/wwc); the Network for Policy Research, Review and Advice on Education & Training (www.norrag.org); the UNICEF Evaluation and Research Database (ERD; www.unicef.org/evaldatabase); and Child Family Community Australia (Research Practice and Policy Information Exchange; www.aifs.gov.au/cfca). Grey literature sources were also included in the searches.

### 5.3 Data collection and analysis

In the following sections, we report only the methods we used in this review. Preplanned but unused methods are reported in Supporting Information: Appendix 4.

#### 5.3.1 Selection of studies

The 2014 search results were screened and assessed using Cochrane methods. For the top‐up searches conducted in 2020 and 2021, we used Cochrane's Screen4Me workflow to help assess the search results. Screen4Me comprises three components: known assessments—a service that matches records in the search results to records that have already been screened in Cochrane Crowd and been labelled as an *RCT* or as *Not an RCT*; the RCT classifier—a machine learning model that distinguishes RCTs from non‐RCTs, and if appropriate, Cochrane Crowd—Cochrane's citizen science platform where the Crowd help to identify and describe health evidence.

For more information about Screen4Me and the evaluations that have been done, please go to the Screen4Me web page on the Cochrane Information Specialist's portal: https://community.cochrane.org/organizational-info/resources/resources-groups/information-specialists-portal. In addition, more detailed information regarding evaluations of the Screen4Me components can be found in the following publications: Marshall 2018, Noel‐Storr 2020, Noel‐Storr 2021, Thomas 2021.

**Figure 1:**
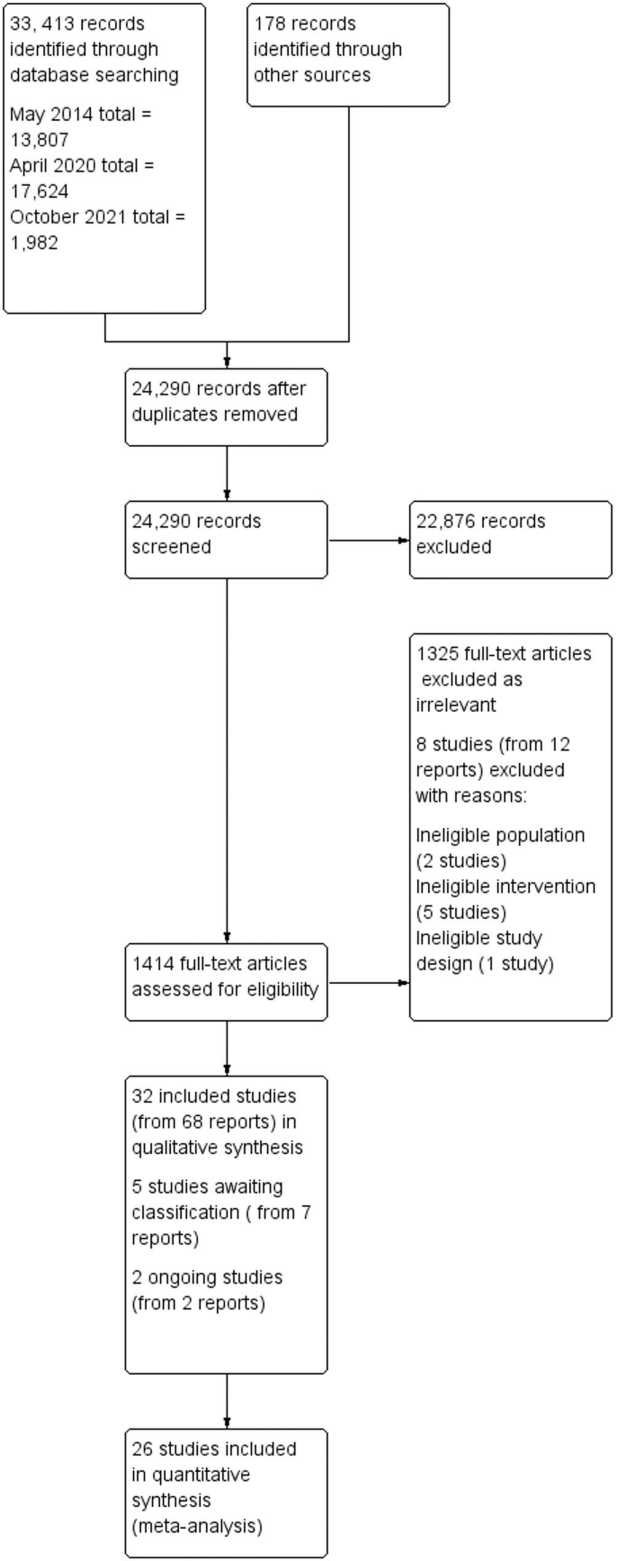
Study flow diagram ‘Selection of Studies@’.

Two reviewers (JD, JH, CMcC, JR) independently screened the titles and abstracts yielded by the search against the inclusion criteria. We obtained full‐text reports for all titles that appeared to meet the inclusion criteria or where there was any uncertainty. Two authors (JH, JR, or CMcC) independently screened the full‐text reports and decided whether they met the inclusion criteria. Additional information from study authors was sought where necessary to resolve questions about eligibility. We resolved disagreement through discussion. The results of the selection process were recorded in a PRISMA diagram (Moher 2015) (Figure 1). Reasons for excluding trials were recorded. Neither of the review authors were blinded to the journal titles or to the study authors or institutions.

#### 5.3.2 Data extraction and management

Two review authors (CMcC, JR) independently extracted data from each included study using a piloted data extraction form. Information was extracted on the following.

- Participant characteristics (age, gender, ethnicity, location, socio‐economic status)
- Intervention characteristics (including delivery, duration, curriculum content, outcomes and measures)
- Comparison characteristics (including whether the study included no intervention control, or treatment as usual)
- Study characteristics (setting, study design, sample size, blinding, follow‐up duration, attrition and handling of missing data, and methods of analysis, funding sources, conflicts of interest)
- Outcome data (relevant details on all primary and secondary outcome measures used and summary data including means, standard deviations, standard errors (for cluster RCTs), confidence intervals for continuous data.

When data were missing, CMcC contacted the study authors. Review authors resolved disagreements through discussion.

#### 5.3.3 Assessment of risk of bias in included studies

Two review authors (CMcC, JH or JR) assessed the risk of bias of included studies independently, using the Cochrane risk of bias tool (Higgins, 2012, Chapter 8).

For cluster‐randomised trials, we made an additional assessment listed as ‘Timing of recruitment of clusters’ based on the advice for dealing with cluster‐RCTs (Higgins, 2021, Chapter 23). For ‘Timing of recruitment of clusters’, we rated RCTs at ‘high’ risk of bias if the studies had recruited the clusters after randomisation and at ‘low’ risk of bias if recruitment occurred before randomisation. For the ‘missing outcome data’ domain, we checked to see if there were any missing clusters as this can also be a cause of bias in cluster RCTs. Disagreements were resolved by discussion and, if necessary, with involvement of a third review author (JAJ or JR). We used the tool to assign ratings of high, low or unclear risk of bias for each of the following domains: sequence generation; allocation concealment; blinding of participants and personnel; blinding of outcome assessment; incomplete outcome data; selective outcome reporting; and other sources of bias (e.g., stopping the trial early, baseline imbalances, choice of design, evidence of carry‐over in cross‐over trials, comparability of groups). We present the results in a risk of bias table, together with details of the available information that led to each judgement.

#### 5.3.4 Measures of treatment effect

We calculated the unadjusted treatment effects using Cochrane's software, Review Manager 5 (RevMan 5) (Review Manager 2014), where possible.

##### Continuous data

We calculated mean differences (where studies used the same measurement scale) or standardised mean differences (SMDs) (if studies used different measurement scales) and 95% CIs for continuous outcome measures. We checked that the direction of the outcome measures pooled in the meta‐analysis were the same and therefore no adjustments were required. Effect estimates were computed from P values, t statistics, analysis of variance (ANOVA) tables or other statistics, as appropriate. We calculated SMDs using Hedges g. We interpreted the SMDs using Cohen effect sizes: 0.2 represents a small effect, 0.5 a moderate effect and 0.8 a large effect (Cohen, 1988).

##### Economic issues

We summarised available data on the costs of programmes within the studies included in the review.

#### 5.3.5 Unit of analysis issues

##### Cluster‐randomised trials

Cluster‐randomised trials are included in this review. We assessed each cluster‐RCT to see if the analysis had accounted for clustering (Table 1). For any studies that had not adjusted for clustering we created an approximate analysis of the cluster‐RCT by inflating the standard errors (SE) (see section 23.1.3 of the *Cochrane Handbook for Systematic Reviewsof Interventions* [Higgins, 2021]). This method requires the intra‐cluster correlation coefficient (ICC), an estimate of the variability within and between clusters, for the RCT. Where a study does not report this, it is possible to use an external estimate of ICC. Two studies reported the ICC (Lonigan, 2015, based their power calculation on an ICC of 0.12 and Yousafzai, 2018, used an ICC of 0.14). We obtained external estimates of the ICC from similar studies (Bedard, 2019; Brotman, 2013; Fedewa, 2015; Matthews, 2009) that used an ICC of 0.1, 0.1, 0.22 and 0.18, respectively. We used an ICC estimate of 0.12 (based on Lonigan's adjustment) to generate an inflated SE. We ran sensitivity analyses using (1) no adjustment, (2) adjustment for clustering assuming a less conservative ICC of 0.07, and (3) adjustment for clustering assuming a more conservative ICC of 0.17 (Tables 2 and 3). All values of unadjusted SE and approximate adjusted SE plus data required to calculate them are listed in Supporting Information: Appendix 6. For all analyses the interpretation of the effects remained stable across the sensitivity analyses. For the remaining cluster‐randomised studies that had not accounted for clustering, a design effect was derived using the average cluster size and ICC (design effect = 1 + (M − 1) × ICC). The standard errors were then multiplied by the square root of the design effect to produce inflated standard errors. Data were entered into RevMan 5 (Review Manager, 2014). We calculated all overall effects using the inverse variance methods to minimise the imprecision of the pooled effect estimate.

**Table 3:** Comparison 2. Sensitivity analyses.

Outcome	Follow‐up	Adjustment for clustering	Studies (*N*)	Effect estimate (SMD) (95% CI)
***Academic achievement***				
General	Short term	None	2 (136)	0.14 (0.01 to 0.28)
		0.07	2 (136)	0.15 (−0.01 to 0.31)
		0.17	2 (136)	0.15 (−0.03 to 0.33)
Language	Short term	None	9 (2058)	0.06 (−0.05 to 0.16)
		0.07	9 (2058)	0.06 (−0.05 to 0.17)
		0.17	9 (2058)	0.06 (−0.06 to 0.18)
	Medium term	None	7 (1549)	0.06 (−0.06 to 0.19)
		0.07	7 (1549)	0.07 (−0.06 to 0.19)
		0.17	7 (1549)	0.06 (−0.09 to 0.21)
Mathematics	Short term	None	17 (6505)	0.16 (0.10 to 0.23)
		0.07	17 (6505)	0.18 (0.12 to 0.25)
		0.17	17 (6505)	0.19 (0.12 to 0.27)
	Medium term	None	10 (3050)	0.11 (0.02 to 0.19)
		0.07	10 (3050)	0.10 (0.01 to 0.18)
		0.17	10 (3050)	0.10 (0.00 to 0.20)
	Long term	None	3 (975)	0.56 (−0.09 to 1.22)
		0.07	3 (975)	0.74 (−0.12 to 1.60)
		0.17	3 (975)	0.57 (−0.10 to 1.24)
Phonics	Short term	None	9 (2095)	0.15 (0.04 to 0.26)
		0.07	9 (2095)	0.12 (0.01 to 0.24)
		0.17	9 (2095)	0.13 (0.02 to 0.24)
	Medium term	None	7 (1554)	0.05 (−0.06 to 0.15)
		0.07	7 (1554)	0.04 (−0.08 to 0.17)
		0.17	7 (1554)	0.04 (−0.11 to 0.19)
Reading	Short term	None	15 (5121)	0.18 (0.05 to 0.30)
		0.07	15 (5121)	0.18 (0.05 to 0.31)
		0.17	15 (5121)	0.19 (0.05 to 0.33)
	Medium term	None	11 (3470)	0.06 (−0.03 to 0.14)
		0.07	11 (3470)	0.05 (−0.02 to 0.13)
		0.17	11 (3470)	0.06 (−0.03 to 0.15)
	Long term	None	4 (1034)	0.56 (−0.10 to 1.22)
		0.07	4 (1034)	0.56 (−0.09 to 1.21)
		0.17	4 (1034)	0.56 (−0.08 to 1.20)
Vocabulary	Short term	None	15 (5225)	0.14 (0.07 to 0.21)
		0.07	15 (5225)	0.15 (0.08 to 0.22)
		0.17	15 (5225)	0.16 (0.08 to 0.24)
	Medium term	None	12 (3529)	0.11 (0.03 to 0.18)
		0.07	12 (3529)	0.12 (0.04 to 0.20)
		0.17	12 (3529)	0.12 (−0.07 to 0.31)
***Emotional well‐being & social competence***				
Emotional regulation	Short term	None	6 (2621)	0.13 (0.01 to 0.26)
		0.07	6 (2621)	0.14 (−0.00 to 0.28)
		0.17	6 (2621)	0.15 (−0.02 to 0.31)
	Medium term	None	2 (1368)	0.02 (−0.08 to 0.13)
		0.07	2 (1368)	0.03 (−0.10 to 0.17)
		0.17	2 (1368)	0.03 (−0.13 to 0.20)
Problem behaviours	Short term	None	10 (3536)	−0.06 (−0.20 to 0.07)
		0.07	10 (3536)	−0.08 (−0.20 to 0.05)
		0.17	10 (3536)	−0.09 (−0.21 to 0.02)
	Medium term	None	9 (2199)	0.02 (−0.06 to 0.11)
		0.07	9 (2199)	0.02 (−0.08 to 0.13)
		0.17	9 (2199)	0.02 (−0.10 to 0.15)
Social skills	Short term	None	12 (4806)	0.10 (−0.06 to 0.26)
		0.07	12 (4806)	0.11 (−0.05 to 0.27)
		0.17	12 (4806)	0.12 (−0.05 to 0.29)
	Medium term	None	9 (2312)	0.07 (−0.02 to 0.15)
		0.07	9 (2312)	0.06 (−0.04 to 0.17)
		0.17	9 (2312)	0.06 (−0.06 to 0.18)
	Long term	None	2 (856)	0.21 (−0.28 to 0.70)
		0.07	2 (856)	0.21 (−0.28 to 0.70)
		0.17	2 (856)	0.22 (−0.28 to 0.71)

*Note*: Statistical method used: SMD (IV, Random, 95% CI).

Abbreviations: CI, confidence intervals; IV, inverse variance; *N*, number of participants; SMD, standardised mean difference.

##### Studies with multiple treatment groups

In the primary analysis, where there were multiple arms of studies, we combined the results of all the eligible intervention groups (centre‐based school readiness programmes) and compared these with the combined results across all eligible control groups, making single pair‐wise comparisons. We investigated heterogeneity by disaggregating these groups and made multiple comparisons. Only one study had two control groups (Warden 1988) and these were disaggregated and compared. We used the approach of combined groups to determine summary estimates (Higgins, 2021, Chapter 10).

#### 5.3.6 Dealing with missing data

Where necessary, we contacted study authors to obtain any data not available in the published report (e.g., group means, standard deviations, details of dropouts or descriptive data regarding the interventions). For studies in which the missing data were not available, we conducted analyses using only the available data (missing data were not imputed). Details of which study authors we contacted and why are in the table of Characteristics of included studies and Table 4.

**Table 4:** Unsuccessful data requests.

Study	Response
***Included studies***	
Abbott‐Shim (2000)	No reply
Atteberry (2019)	No reply
Barnett (2008)	No reply
Castro (2017)	No reply
Coffman (1967)	Could not locate author
Diamond (2019)	No reply
Lipsey (2009)	No reply
Puma (2005)	No reply
Soloman (2018)	No reply
***Excluded studies***	
Di Lorenzo (1967)	Could not locate authors
Gür (2017)	No reply
Ramey (1974)	No reply
***Studies awaiting classification***	
Alpern (1966)	Author deceased
Ametjian (1965)	Author deceased
Karnes (1969)	Author deceased
Miller (1969)	No reply
Stukat (1971)	Could not locate authors

#### 5.3.7 Assessment of heterogeneity

We assessed clinical and methodological heterogeneity of studies with regard to study design and by comparing the distribution of study factors (e.g., individually randomised vs. cluster‐randomised trial, allocation concealment, blinding of outcome assessors, loss to follow‐up, intervention type). We assessed the extent of between trial differences and the consistency of meta‐analysis results in three ways: by visual inspection of the forest plots; by performing Chi‐squared tests (*χ*²) of heterogeneity (using a *p*‐value of less than 0.10 interpreted as evidence of heterogeneity); and by examining the *I*² statistic [the *I*² statistic describes approximately the proportion of variation in point estimates due to heterogeneity rather than sampling error]. We considered *I*² values less than 30% as indicating low heterogeneity, values in the range 30% to 70% as indicating moderate heterogeneity, and values between 70% to 90% as indicating substantial heterogeneity, and anything over 90% was considered substantial. We attempted to identify any significant determinants of heterogeneity categorised as moderate or high (Higgins, 2021; Ryan, 2014). The details of this information are included in the Characteristics of included studies tables and discussed in Section Main Results, 6. We explored the between study variance in random effects models using TAU². We conducted subgroup analysis to investigate heterogeneous results. We also conducted sensitivity analyses, where data permitted, and we discuss the possible reasons for any heterogeneity in Section Authors' Conclusions, 7.

#### 5.3.8 Assessment of reporting biases

To assess for small study effects, we drew funnel plots for the following outcomes where we had at least 10 studies in a meta‐analysis: academic achievement (maths, reading, vocabulary [short‐ and medium‐term] and emotional well‐being and social competence [problem behaviours and social skills (short‐term)]) (Sterne, 2011).

#### 5.3.9 Data synthesis

Where the interventions were similar in (i) age of children starting programme, (ii) content of programme delivered, (iii) intensity and duration of programme, we synthesised the results in a meta‐analysis. As we expected some degree of clinical heterogeneity across studies measuring similar effects, we used a random‐effects model (Higgins 2021, 10.10.4.1). We calculated all overall effects using inverse variance methods. Where data could not be pooled, we conducted a narrative synthesis.

#### 5.3.10 Subgroup analysis and investigation of heterogeneity

We had pre‐planned subgroup analysis for intervention intensity and duration, socioeconomic status and English language learners but were unable to conduct these due to insufficient studies. If more than one study had been included, we assessed heterogeneity using the *I*² statistic.

#### 5.3.11 Sensitivity analysis

To explore the impact of studies with high risk of bias on the robustness of the results of the review, we conducted sensitivity analyses by removing studies with a high risk of bias on baseline measurements and blinding of outcome assessment, and re‐analysed the remaining studies to determine whether these factors affected the results. We also re‐analysed the data using different statistical approaches (using fixed‐effects model) (Higgins, 2021, Chapter 10) to explore the impact of our decision to use a random‐effects model.

#### 5.3.12 Summary of findings and assessment of the certainty of the evidence

We have presented the main findings of the review in transparent and simple summary of findings tables. These provide key information about the certainty of evidence, the magnitude of effect of the interventions examined, and the sum of the available data for the main outcomes using the GRADEpro Guideline Development Tool (GRADEpro GDT 2021). We used GRADE to assess the certainty of evidence. Comparisons were made for centre‐based early education interventions for improving school readiness versus no intervention control and centre‐based early education interventions for improving school readiness versus treatment as usual for school readiness, adverse effects, cognitive development, emotional well‐being and social competence (social skills), health development and physical development. The following six elements are included in these tables.

- A list of all the outcomes
- A measure of the typical burden of these outcomes
- Absolute and relative magnitude of effect
- Numbers of participants and studies that address these outcomes
- A rating of the overall certainty of evidence for each outcome
- Additional comments

The GRADE (Schünemann, 2021; section 12.2) system assigns four levels of evidence that should be interpreted as follows.

- High: we are very confident that the true effect lies close to that of the estimate of the effect
- Moderate: we are moderately confident in the effect estimate; the true effect is likely to be close to the estimate of the effect, but there is a possibility that it is substantially different
- Low: our confidence in the effect estimate is limited; the true effect may be substantially different from the estimate of the effect.
- Very low: we have very little confidence in the effect estimate; the true effect is likely to be substantially different from the estimate of effect

Data from RCTs start at the high level of evidence and are then lowered by one or two levels for the following reasons.

- Serious (reduced by one level) or very serious (reduced by two levels) study limitation for risk of bias.
- Serious (reduced by one level) or very serious (reduced by two levels) inconsistency between study results.
- Some (reduced by one level) or major (reduced by two levels) uncertainty about directness (the correspondence between the population, the intervention, or the outcomes measured in the studies actually found, and those under consideration in our review).
- Serious (reduced by one level) or very serious (reduced by two levels) imprecision of the pooled estimate.
- Strong suspicion of publication bias (reduced by one level).

Using the GRADE approach, two review authors (CMcC/JR) independently graded the certainty of evidence as high, moderate, low or very low, according to the presence of the following five factors: limitations in design and implementation of available studies; indirectness of evidence; inconsistency of results; imprecision of results; and high likelihood of publication bias. Any disagreements with the GRADE ratings were discussed and resolved with a third author (J‐AJ).

## 6 RESULTS

### 6.1 Description of studies

#### 6.1.1 Results of the search

**Figure 2:**
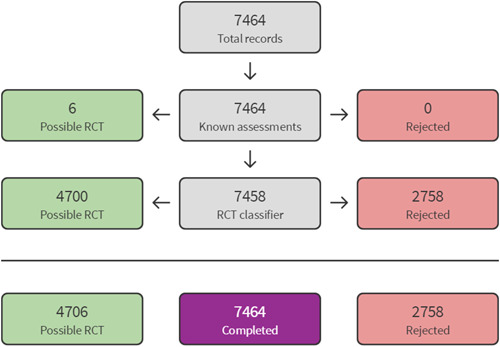
Screen4Me April pre‐2014 results.

**Figure 3:**
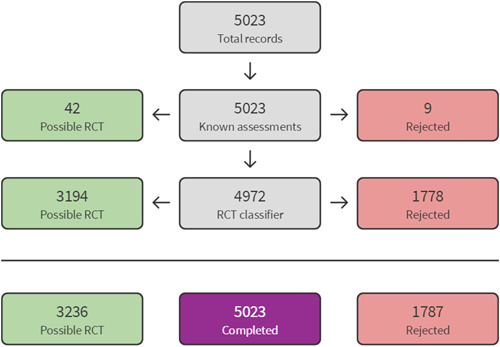
Screen4Me April Post 2014–2020 results.

**Figure 4:**
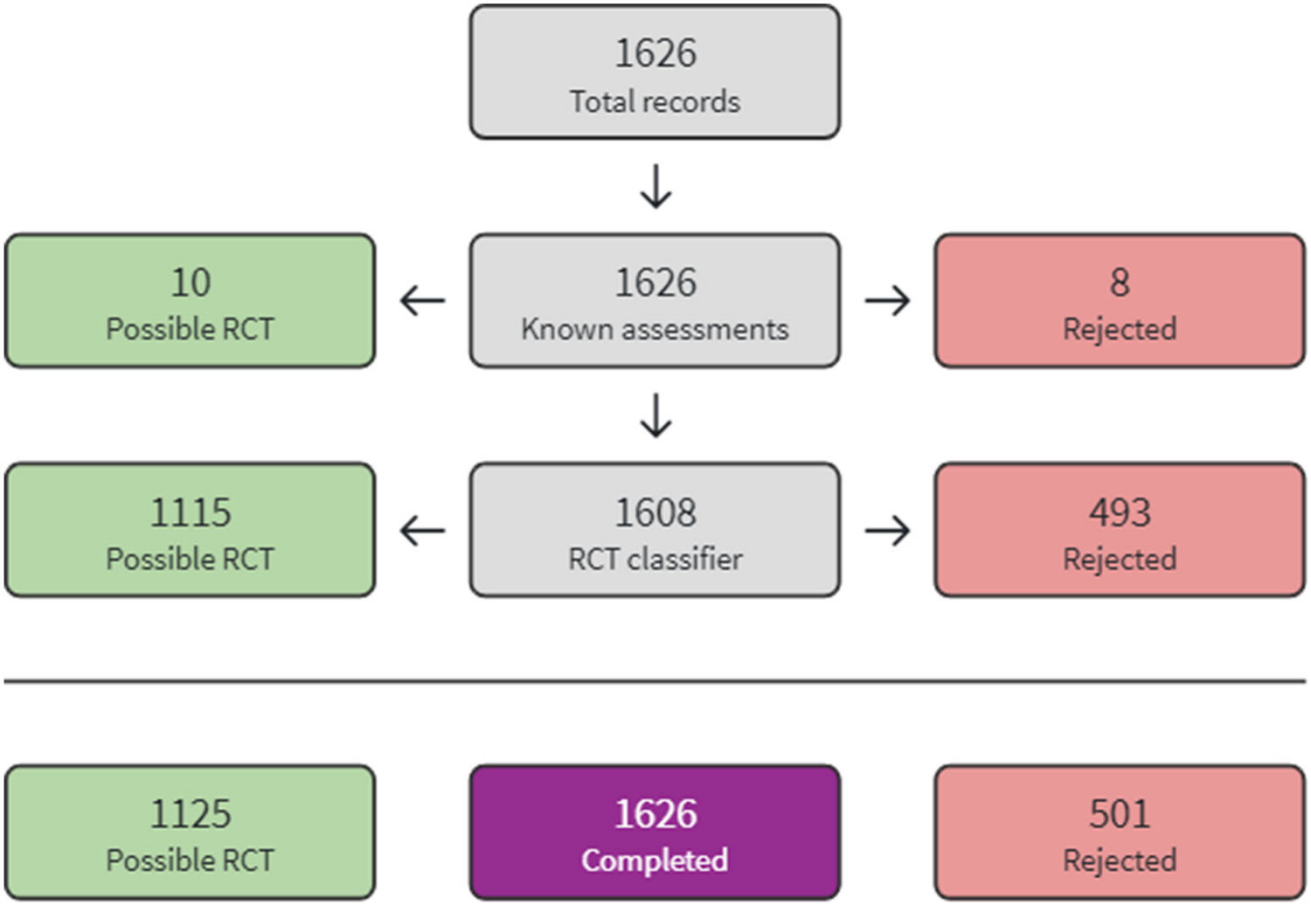
Screen4Me summary diagram October 2021.

The searches found a total of 33,591 search results. Of these, 33,143 were retrieved by database searches (13,807 in May 2014; 17,624 in April 2020; 1982 in October 2021), and 178 from other sources. A total of 9301 duplicates were removed and the remaining 24,290 records were screened for eligibility. Records from the 2014 searches were all screened manually. We used Cochrane's Screen4Me workflow to assist with screening the later searches (2020 and 2021) for potential reports of randomised trials. A total of 22,876 records were excluded at title and abstract stage. See study flow diagram in Figure 1. Of these, 5046 were excluded by the Screen4Me machine classifier (Figures 2, 3, 4), and a further 17,830 were excluded by manual screening. We obtained the full texts of 1414 papers that merited closer inspection. We excluded 1325 reports which we deemed to be irrelevant and a further 12 full text reports (8 studies) with reasons, which are reported in Characteristics of excluded studies tables.

Assessment of the remaining 77 full texts led to the identification of 32 included studies (68 reports; see Characteristics of included studies), 5 studies awaiting classification (from 7 reports) (see Characteristics of studies awaiting classification) and 2 ongoing studies (from 2 reports) (see Characteristics of ongoing studies). See study flow diagram in Figure 1. We excluded a total of 8 studies (12 full text reports) with reasons (see Figure 1).

#### 6.1.2 Included studies

We included 32 trials in this review. Useable data could not be sourced in eight studies and therefore narrative analysis only is reported (Table 5). Seven of the included studies were part of a large multi‐site trial run by the Preschool Curriculum Evaluation Research Initiative (PCER) (PCER, 2008). The trial had 12 sites across the USA but 5 of these did not meet our Criteria for considering studies for this review. We have decided to report the included PCER studies individually; each site was independent from the other study sites and different interventions used across these sites. An overview is provided in Table 5 and further details are in the Characteristics of included studies tables. A further 5 studies met the inclusion criteria but are categorised as awaiting classification (see Characteristics of studies awaiting classification tables) because of missing data. Every effort was made to contact the study authors; however, as some of the studies date back some years, some of the primary authors were retired or deceased. We identified two ongoing studies (see Characteristics of ongoing studies tables). All 32 included studies (5 individually randomised RCTs, 26 cluster‐randomised RCTs and 1 quasi‐randomised cluster RCT) in this review provided data on the effectiveness of centre‐based early education interventions for improving school readiness. The studies were published over a 59‐year period; the earliest was Weikart 1967, which began in 1962 and the most recent, Courtier 2021. Unpublished data were obtained from five trial authors (Bierman, 2008; Blair, 2014; Lillard, 2017; Lonigan, 2015; Yousafzai, 2018), including raw data and number of participants McCartan (2019) [pers comm]; McCartan (2020) [pers comm]; McCartan (2021a) [pers comm]; McCartan (2021b) [pers comm]; McCartan (2021c) [pers comm]. Furthermore, an additional 17 authors were contacted to request further information, but these requests were unsuccessful (see Table 4).

**Table 5:** Overview of included studies.

Study & design	Comparator (number of arms) Sample size	Curriculum intervention	Curriculum focus	Curriculum organisation & add‐ons	Curriculum add‐ons	Meta‐analysis	Primary outcomes	Secondary outcomes	Time points
Abbott‐Shim (2000) RCT	Wait list (1) *N* = 142	HeadStart	Comprehensive	Theme based	None	Yes	None	Emotional well‐being & social competence: problem behaviour & social skills	<1 year
Atteberry (2019) C‐RCT	TAU (1) *N* = 226	Full‐day kindergarten	Comprehensive	Theme based	None	No	Narrative analysis only		
Barnes (2016) C‐RCT	TAU (2) *N* = 541	Pre‐kindergarten Mathematics Tutorial + ATT	Numeracy Attention training	Manualised	None	Yes	None	Academic achievement: mathematics; and Emotional well‐being & social competence: emotional regulation	<1 year
Barnett (2008) C‐RCT	TAU (1) *N* = 210	Tools of the Mind	Language/literacy Numeracy Social/emotional	Manualised	Teacher training	No	Narrative analysis only		
Bierman (2008) C‐RCT	TAU (1) *N* = 356	HeadStart REDI + PATHS	Language & literacy Social/emotional	Manualised	Teacher training Home‐based component	Yes	None	Academic achievement: mathematics, phonics, reading, vocabulary; Emotional well‐being & social competence: problem behaviours & social skills; and Economic costs	1–2 years
Blair (2014) C‐RCT	TAU (1) *N* = 759	Tools of the Mind	Language/literacy Numeracy Social/emotional	Promoting skills	Teacher training	Yes	None	Academic achievement: general, mathematics, vocabulary; and Emotional well‐being & social competence: problem behaviours	<1 year
Blatt (1965) RCT	TAU (2) *N* = 59	Preschool programme + ‘responsive environment’	Literacy/literacy Numeracy Social/emotional & physical development Multi‐sensory	Experimental	Home‐based component	Yes	None	Academic achievement: general, language, reading; and Cognitive development	>2 years
Castro (2017) C‐RCT	TAU (1) *N* = 340	NNSR Professional Development Programme	Literacy/literacy, numeracy, and social/emotional learning in Spanish‐English dual language learners	Manualised	Teacher training	No	Narrative analysis only		
Coffman (1967) C‐RCT	No treatment & TAU (4) *N* = 229	Standard curriculum with add‐ons designed to address (motor, auditory‐language, or visual deficits) or assigned to a cognitive class (no deficits identified)	Comprehensive	Manualised	Personalised to child's needs	No	Narrative analysis only		
Courtier (2021) C‐RCT	TAU (2) *N* = 175	Montessori in public & private preschool settings	Active learning	Manualised	None	Yes	None	Academic achievement: mathematics, phonics, reading, vocabulary	<1 year
Deutsch (1971) RCT	Wait list (1) *N* = 404	Institute of Developmental Studies curriculum	Literacy Social/emotional	Physical classroom organisation	Teacher training Home‐based component	Yes	None	Cognitive development	>2 years
Diamond (2019) C‐RCT	TAU (1) *N* = 351	Tools of the Mind	Language/literacy Numeracy Social/emotional	Manualised	Teacher training	No	Narrative analysis only		
Farran (2015) C‐RCT	TAU (1) *N* = 877	Tools of the Mind	Language/literacy Numeracy Social/emotional	Manualised	Teacher training	Yes	None	Academic achievement: mathematics; and Emotional well‐being & social competence: social skills	>2 years
Hsueh (2014) C‐RCT	TAU (3) *N* = 933	Head Start CARES: Incredible Years Preschool PATHS; Tools of the Mind‐Play	Social/emotional Active learning	Manualised	Teacher training Home‐based component	Yes	None	Academic achievement: general; and Emotional well‐being & social competence: problem behaviours & social skills	<1 year
Lillard (2017) Q‐RCT	TAU (1) *N* = 141	Montessori	Active learning	Manualised	None	Yes	None	Academic achievement: mathematics, vocabulary	>2 years
Lipsey (2009) C‐RCT	Wait list (1) *N* = 2990	Tennessee Voluntary Pre‐k Programme	Range of pre‐approved curriculum	Manualised	None	No	Narrative analysis only		
Lonigan (2015) C‐RCT	TAU (2) *N* = 760	Preschool PATHS + Literacy Express + Pre‐K Maths	Literacy Numeracy Social/emotional	Manualised	Home‐based component	Yes	None	Academic achievement: reading, vocabulary; and Emotional well‐being & social competence: social skills	<1 year
PCER (2008), Bright Beginnings & Creative Curriculum C‐RCT	TAU (2) *N* = 309	Bright Beginnings Creative Curriculum	Literacy Active learning	Manualised	Teacher Training Home‐based component	Yes	None	Academic achievement: language, mathematics, phonics, reading, vocabulary; and Emotional well‐being & social competence: problem behaviours & social skills	1–2 years
PCER (2008), Creative Curriculm C‐RCT	TAU (1) *N* = 194	Creative Curriculum	Comprehensive	Interest areas	Teacher training	Yes	None	Academic achievement: language, mathematics, phonics, reading, vocabulary; and Emotional well‐being & social competence: problem behaviours & social skills	1–2 years
PCER (2008), Doors to Discovery & Let's Begin C‐RCT	TAU (2) *N* = 297	Doors to Discovery Let's Begin with the Letter People	Literacy	Theme based	Teacher training Home‐based component	Yes	None	Academic achievement: language, mathematics, phonics, reading, vocabulary; and Emotional well‐being & social competence: problem behaviours & social skills	1–2 years
PCER (2008), Language‐Focused Curriculum C‐RCT	TAU (1) *N* = 195	Language‐Focused Curriculum	Literacy	Theme based Manualised	Teacher training	Yes	None	Academic achievement: language, mathematics, phonics, reading, vocabulary; and Emotional well‐being & social competence: problem behaviours & social skills	1–2 years
PCER (2008), Literacy Express + DLM + OCR Pre‐K C‐RCT	TAU (3) *N* = 297	Literacy Express DLM Early Childhood Express + Open Court Reading Pre‐K	Literacy	Theme based Manualised	None	Yes	None	Academic achievement: language, mathematics, phonics, reading, vocabulary; and Emotional well‐being & social competence: problem behaviours & social skills	1–2 years
PCER (2008), Project Construct C‐RCT	TAU (1) *N* = 231	Project Construct	Social/emotional Multi‐sensory	Theme based	Teacher training	Yes	None	Academic achievement: language, mathematics, phonics, reading, vocabulary; and Emotional well‐being & social competence: problem behaviours & social skills	1–2 years
PCER (2008), Ready, Set, Leap! C‐RCT	TAU (1) *N* = 286	Ready Steady Leap!	Multi‐sensory Active learning	Manualised	Teacher training Home‐based component	Yes	None	Academic achievement: language, mathematics, phonics, reading, vocabulary; and Emotional well‐being & social competence: problem behaviours & social skills	1–2 years
Peisner‐Feinberg (2019) C‐RCT	Wait list (1) *N* = 582	Creative Curriculum; Opening the World of Learning; High/Scope Preschool Curriculum; Tools of the Mind; Investigator Club; Prekindergarten Learning System; Passports: Experiences for Pre‐K Success	Literacy Social/emotional	Manualised	None	Yes	None	Academic achievement: language; and Emotional well‐being & social competence: problem behaviours & social skills	<1 year
Puma (2005) C‐RCT	TAU (1) *N* = 3723	HeadStart	Comprehensive	Manualised	Home‐based component	No	Narrative analysis only		
Raver (2009) C‐RCT	TAU (1) *N* = 467	Chicago School Readiness Project	Social/emotional	Manualised	Teacher training	Yes	None	Academic achievement: mathematics, reading, vocabulary	>2 years
Soloman (2018) C‐RCT	TAU (1) *N* = 195	Tools of the Mind	Language/literacy Numeracy Social/emotional	Manualised	Teacher training	No	Narrative analysis only		
Van de Riet (1972) RCT	TAU (1) *N* = 86	Learning to Learn	Sequential active learning programme	Manualised	Home‐based component	Yes	None	Academic achievement: general, language, reading; Cognitive development; and Physical development	>2 years
Warden (1988) RCT	TAU (1) *N* = 80	Developmentally appropriate curriculum	Language & literacy Active learning	Manualised	Home‐based component	Yes	School readiness	Academic achievement: mathematics	<1 year
Weikart (1967) C‐RCT	No treatment (1) *N* = 123	High/Scope Preschool Curriculum	Cognitive & social/emotional Active learning	Manualised	Teacher Training Home‐based component	Yes	None	Academic achievement: general, language, reading; Cognitive development; Emotional well‐being & social competence: problem behaviours; and Economic costs	>2 years
Yousafzai (2018) C‐RCT	TAU (1) *N* = 340	Youth Leaders for Early Childhood Assuring Children are Prepared for School (LEAPS) based on High/Scope	Comprehensive	Theme‐based	Teacher/community youth leader training Home‐based component	Yes	School readiness	Academic achievement: mathematics; Emotional well‐being & social competence: social skills; and Physical development	<1 year

Abbreviations: ATT, Attention Tutorial Intervention; CARES, Classroom‐Based Approaches & Resources for Emotion & Skill promotion; C‐RCT, cluster randomised control trial; DLM, Dynamic Learning Maps; *N*, number of participants; NNSR, Nuestros Ninos School Readiness; PATHS, Promoting Alternative Thinking Strategies; PCER, Preschool Curriculum Evaluation Researcher (PCER) Consortium; Q‐RCT, quasi‐randomised controlled trial; RCT, randomised controlled trial; REDI, REsearch‐based, Developmentally Informed; TAU, treatment as usual.

##### Study design

Most trials (81.3%) were randomised at a cluster level. Of the 26 trials randomised by cluster, randomisation across most studies was performed at a classroom level. In Yousafzai (2018), villages were randomised. Five studies randomised individual children (Abbott‐Shim, 2000; Blatt, 1965; Deutsch, 1971; Van de Riet, 1972; Weikart, 1967). One study randomised children in the intervention group, but not the control group (Lillard, 2017). Warden 1988 did not provide information about the randomisation process.

###### Treatment arms

A total of 23 studies allocated participants to one of two groups, the experimental and control group. One study, allocated participants to one of four active treatment groups (Coffman, 1967); Hsueh (2014) and PCER (2008), Literacy Express + DLM + OCR Pre‐K had three active treatment groups. In six studies, participants were allocated to one of two treatment groups. Details of treatment arms are described in Table 5.

##### Population

Children ranged in age from as young as 3 years old (Blair, 2014; Hsueh, 2014; Lillard, 2017; PCER, 2008, Bright Beginnings & Creative Curriculum; Weikart, 1967) to 7 years old (Deutsch, 1971). Race/ethnicity and socioeconomic status was not uniformly reported in studies. In a number of studies the majority of participants were African American, with percentages ranging from 17% (Lillard, 2017) to 100% in (Deutsch, 1971; Soloman, 2018; Van de Riet, 1972 and Weikart, 1967). The majority of studies reported ethnicity and half (*k* = 16) had a majority sample from a racial/ethnic minority group (most commonly, African American or Latino). A number of studies (*k* = 6) identified low socio‐economic groups or areas as target populations and were located in areas of high social and economic deprivation (Abbott‐Shim, 2000; Blatt, 1965; PCER, 2008, Language‐Focused Curriculum; Raver, 2009; Van de Riet, 1972; Weikart, 1967). We included three studies of Head Start programmes in our analyses; these programmes specifically target low‐income areas as a means of tackling poverty and social exclusion.

##### Setting

Twenty‐eight included studies were conducted in the USA, two in Canada (Diamond, 2019; Soloman, 2018), one in France (Courtier, 2021) and one trial was conducted in Pakistan (Yousafzai, 2018). Interventions were delivered in centre‐based settings, usually pre‐kindergarten or elementary schools for at least one half day, 4 days per week over one academic year. Trials lasted between 1 and 6 years. Sample size ranged from 59 (Blatt, 1965) to 3723 (Puma, 2005). The mean sample size was 528. The total number of participants across the studies was 16,899.

##### Interventions

Interventions in this review took a number of different forms and had either been designed specifically for trial or were adaptations or enrichments of an existing curriculum. Named interventions included Head Start, Bright Beginnings, Creative Curriculum, Montessori, PATHS and Tools of the Mind. The rationale of the included interventions were designed to equip young children with pre‐literacy and numeracy skills, develop approaches to self‐regulation and promote good social skills and reduce problem behaviours that can impede with learning and establishing bonds within their peer group and other important adults beyond their family circle. These were developed in line with national‐, state‐ or international‐educational guidance (predominantly US‐based) based on common standards in curriculum content, methods, teacher training requirements and additional add‐ons where appropriate. Most had some established theoretical underpinnings using recognised pedagogical approaches developed by Bodrova (1996); Montessori, Vygotsky (1967), and others. Despite the focus being on school readiness, all interventions focused on academic attainment, with a particular focus on early literacy and numeracy. Eighteen studies identified and discussed specific aspects of teacher training or professional development activities, ranging from resources and guides to summer school attendance (Weikart, 1967). Eleven studies included some involvement with the home or parents as part of their programme plan. For most, this was either in the form of take‐home work or parent training programmes; two studies included home visits (Blatt, 1965 and Weikart, 1967). All studies were implemented by practising teachers within the confines of their classrooms. Some classroom settings were designed in a particular way to support curriculum learning or specialist materials were made available to structure learning through play.

No one theme or topic was included consistently across studies and curricula included a range of foci which were chosen for a variety of reasons. All studies included topics and content which were age‐appropriate and which would normally be included as part of an early years curriculum. All studies reported a multi‐dimensional intervention that targeted a number of different skills. A summary of the main curricular content is included in the overview of included studies (Table 5). A number of studies mentioned a specific focus on literacy and emerging literacy, and numeracy (Barnes, 2016). Social and emotional aspects were a key factor and active learning approaches and multi‐sensory learning environments were featured (Courtier, 2021; Hsueh, 2014; Lillard, 2017; PCER, 2008, Bright Beginnings & Creative Curriculum; PCER, 2008, Ready, Set, Leap!; Van de Riet, 1972; Warden, 1988; Weikart, 1967). Some curricula were organised according to themes and were structured sequentially (PCER, 2008, Doors to Discovery & Let's Begin; PCER, 2008, Language‐Focused Curriculum; PCER, 2008, Literacy Express + DLM + OCR Pre‐K). Programmes varied in terms of structure and flexibility. A number were heavily manualised and included a structured programme which teachers worked through sequentially. Other programmes were less structured and did not include a specified programme to follow, instead teachers created tasks and learning experiences which reflected the themes of the intervention. Programmes were delivered for at least one half day, 4 days a week over an academic year (fall to spring).

##### Comparators

Of the 32 trials included in this review, the majority (81.3%) compared the intervention to TAU. The remaining trials compared the effectiveness of the intervention to a wait‐list control (Abbott‐Shim, 2000; Deutsch, 1971; Lipsey, 2009; Peisner‐Feinberg, 2019) or no intervention control (Weikart, 1967). One study had two control groups, no intervention and TAU (Coffman, 1967).

Standard TAU school readiness interventions included: Creative Curriculum (PCER, 2008, Creative Curriculm); HeadStart (Abbott‐Shim, 2000; Bierman, 2008); Investigator Club Prekindergarten Learning System (Peisner‐Feinberg, 2019); Opening the World of Learning (Peisner‐Feinberg, 2019); Passports: Experiences for Pre‐K Success (Peisner‐Feinberg, 2019).

##### Heterogeneity

Clinical heterogeneity. There was no unexpected variability in the clinical heterogeneity in each included study. The participants in each included study were of a similar age (mean 4.3 years, range 3 to 7 years). The interventions all followed a similar format and delivery. Similar outcomes were measured by all included studies.

##### Outcomes

Despite school readiness being the main objective in the intervention, the main outcome (school readiness) was only measured in two studies and none of the studies reported adverse effects. Other studies measured a range of outcomes, including academic, cognitive and behavioural measures. Data were pooled, and little to no difference was observed between centre‐based early education interventions and no treatment control or TAU. The sample size was small and there was considerable heterogeneity in the study designs making it difficult to reach firm recommendations, with the evidence downgraded to very low certainty. See Table 6.

**Table 6:** Outcome measures.

Primary outcome measures					
*School readiness*					
Sub‐domain	Measure	Scoring	Study	Comparison	Meta‐analysis
Not applicable	Kindergarten Readiness Test (Larson, 1988)	High score favourable 94%–100%: above average 77%–92%: average 63%–75%: lower average 45%–61% & below: below average/questionable readiness	1 study: Warden (1988)	TAU	Yes
	International Development and Early Learning Assessment (IDELA; Save the Children, 2015)	High score favourable Range reported: 66.2–29.0	1 study: Yousafzai (2018)		

**Secondary outcome measures**					
***Academic achievement***					
**Sub‐domain**	**Measure**	**Scoring**	**Study**	**Comparison**	**Meta‐analysis**
General achievement	California Achievement Test (Tiegs, 1957)	High score favourable Upper & lower limits: 99–1	1 study: Weikart (1967)	Control	No
	Social Skills Rating System, Academic Competence scale (SSRS; Gresham, 1990)	High score favourable Upper & lower limits: 20–0	1 study: Blair (2014)	TAU	Yes
	School achievement test	High score favourable Range reported: 30.7–25.7	1 study: Blatt (1965)		
	Academic Rating Scale (National Center for Education Statistics, 2021)	High score favourable 30‐items based on 3‐point Likert scale Scores are averages across the survey items in each sub scale (language & literacy; mathematical thinking; general knowledge)	1 study: Hsueh (2014)		
	School grades	High score favourable	1 study: Van de Riet (1972)		
Language	Illinois Test of Psycholinguistic Abilities (ITPA; McCarthy, 1961)	High score favourable Standardised M 10 (SD 3)	1 study: Weikart 1967	Control	Yes
			2 studies: Blatt (1965); Van de Riet (1972)	TAU	Yes
	TOLD Grammatical Understanding sub scale (Newcomer, 1997)	High score favourable 237 items across 6 sub scales One point given for correct responses & zero for incorrect but ceiling/discontinue scores are applied for some items	8 studies: Bierman (2008); PCER (2008), Bright Beginnings & Creative Curriculum; PCER (2008), Creative Curriculm; PCER (2008), Doors to Discovery & Let's Begin; PCER (2008), Language‐Focused Curriculum; PCER (2008), Literacy Express + DLM + OCR Pre‐K; PCER (2008), Project Construct; PCER (2008), Ready, Set, Leap!		
Mathematics	Metropolitan Achievement Test, Computation subtest (Bixler, 1958)	High score favourable Upper & lower limits: 100–0	1 study: Deutsch (1971)	Control	Yes
	Woodcock Johnson, Qualitative Concepts subtest (McGrew, 2001)	High score favourable Standardised M 100 (SD 15)	1 study: Peisner‐Feinberg (2019)		
	California Achievement Test, Arithmetic subtest (Tiegs, 1957)	High score favourable Upper & lower limits: 99–1	1 study: Weikart (1967)		
	Test of Early Mathematics Ability, 3rd Edition* (TEMA‐3; Ginsburg, 2003)	High score favourable below 69: ‘very poor’; 70–79: poor 80–89: below average 90–110: average 111–120: above average 121–130: superior greater than 131: very superior	1 study: Barnes (2016)	TAU	Yes
	Stanford Achievement Test, Arithmetic subtest (Kelley, 1952)	High score favourable 1–22: below average 23–76: average 77–99: above average	1 study: Van de Riet (1972)		
	Woodcock‐Johnson Applied Problems test (McGrew, 2001)	High score favourable Upper & lower limits: 160–40 Standardised M 100 (SD 15)	11 studies: Blair (2014); Courtier (2021); Farran (2015); Lillard (2017); PCER (2008), Bright Beginnings & Creative Curriculum; PCER (2008), Creative Curriculm; PCER (2008), Doors to Discovery & Let's Begin; PCER (2008), Language‐Focused Curriculum; PCER (2008), Literacy Express + DLM + OCR Pre‐K; PCER (2008), Project Construct; PCER (2008), Ready, Set, Leap!		
	Academic Rating Scale, Mathematical Thinking (National Center for Education Statistics, 2021)	High score favourable 30 items based on 3‐point Likert scale Scores are averages across the survey items in each sub scale (language & literacy; mathematical thinking; general knowledge)	1 study: Hsueh (2014)		
	Child Math Assessment (Starkey, 2012)	High score favourable Upper & lower limits: not standardised 0–1 score	1 study: Lonigan (2015)		
	Early Maths Skills score (Zill, 2003a)	High score favourable 19 items	1 study: Raver (2009)		
	Metropolitan Readiness Test, Quantitative subtest (Bixler, 1958)	High score favourable Upper & lower limits: 100–0	1 study: Warden (1988)		
	International Development and Early Learning Assessment, Emergent Mathematics (IDELA; Save the Children, 2015)	High score favourable	1 study: Yousafzai (2018)		
Phonics	Evaluation des fonctions cognitives et Apprentissages, Phonologie subtest (Billard, 2012)	High score favourable Scored by number of correctly repeated syllables (0–28)	1 study: Courtier (2021)	TAU	Yes
	Comprehensive Test of Phonological Processing (CTOPP), Elision subtest, Kindergarten (Wagner, 1999)	High score favourable Yields six types of normative scores: age equivalents, grade equivalents, percentile ranks, subtest scaled scores, composite indexes, and developmental scores Subtest scaled scores M 10 (SD 3) Composite score indexes M 100 (SD 15)	Bierman, 2008; PCER (2008), Bright Beginnings & Creative Curriculum; PCER (2008), Creative Curriculm; PCER (2008), Doors to Discovery & Let's Begin; PCER (2008), Language‐Focused Curriculum; PCER (2008), Literacy Express + DLM + OCR Pre‐K; PCER (2008), Project Construct; PCER (2008), Ready, Set, Leap!		
Reading	California Achievement Test, Reading subtest (Tiegs, 1957)	High score favourable Upper & lower limits: 99–1	1 study: Deutsch (1971)	Control	Yes
	Woodcock‐Johnson, Letter Word Identification subtest (McGrew, 2001)	High score favourable Standardised M 100 (SD 15)	1 study: Peisner‐Feinberg (2019)		
	Metropolitan Achievement Test, Reading Subtest (Bixler, 1958)	High score favourable Upper & lower limits: 100–0	1 study: Weikart (1967)		
	Test of Preschool Early Literacy (TOPEL; Lonigan, 2007) Print Knowledge subtest	High score favourable 36 items in Print Knowledge sub scale, 1 of 3 sub scales, total 98 items	Bierman (2008); Lonigan (2015)	TAU	Yes
	Woodcock‐Johnson, Letter Word Identification subtest (McGrew, 2001)	High score favourable Standardised M 100 (SD 15)	Blair (2014); Farran (2015); Lillard (2017); PCER (2008), Bright Beginnings & Creative Curriculum; PCER (2008), Creative Curriculm; PCER (2008), Doors to Discovery & Let's Begin; PCER (2008), Language‐Focused Curriculum; PCER (2008), Literacy Express + DLM + OCR Pre‐K; PCER (2008), Project Construct; PCER (2008), Ready, Set, Leap!		
	Lee Clark Reading Readiness Test (Lee, 1934)	High score favourable Upper & lower limits: 50–0	Blatt (1965)		
	Evaluation Des fonctions cognitives et des Apprentissages, Lecture subtest (Billard, 2012)	High score favourable Scored by number correctly completed items (0–70)	Courtier (2021)		
	Letter Naming Score (Zill, 2003b)	High school favourable Upper & lower limits: 26–0, calculated to total %	Raver (2009)		
	Diagnostic Reading Test (Spache, 1963)	High score favourable Upper & lower limits: 15–0	Van de Riet (1972)		
	Metropolitan Achievement Test, Beginning Reading Subtest (Bixler, 1958)	High score favourable Upper & lower limits: 100–0	Warden (1988)		
Vocabulary	Peabody Picture Vocabulary Test* (PPVT; Dunn, 1965, 2007)	High score favourable Standardised M 100 (SD 15)	2 studies: Deutsch (1971); Weikart (1967)	Control	Yes
	Woodcock Johnson, Picture Vocabulary subtest (McGrew, 2001)	High score favourable Upper & lower limits: 160–40 Standardised M 100 (SD 15)	1 study: Peisner‐Feinberg (2019)		
	Evaluation du Language Oral (Khomsi, 2001)	High score favourable Number of items correctly answered (0–20)	1 study: Courtier (2021)	TAU	Yes
	Peabody Picture Vocabulary Test* (PPVT; Dunn, 1965, 2007)	High score favourable Upper & lower limits: 160–40 Standardised M 100 (SD 15)	11 studies: Blatt (1965); Farran (2015); Lillard (2017); PCER (2008), Bright Beginnings & Creative Curriculum; PCER (2008), Creative Curriculm; PCER (2008), Doors to Discovery & Let's Begin; PCER (2008), Language‐Focused Curriculum; PCER (2008), Literacy Express + DLM + OCR Pre‐K; PCER (2008), Project Construct; PCER (2008), Ready, Set, Leap!; Raver (2009)		
	Expressive One‐Word Picture Vocabulary Test (EOWPVT; Brownell, 2000)	High score favourable Standardised M 100 (SD 15)	3 studies: Bierman (2008); Blair (2014); Lonigan (2015)		
***Cognitive development***					
Intelligence	Stanford‐Binet Intelligence Scale (Terman, 1960)	High score favourable Upper & lower limits: 160–80	2 studies: Deutsch (1971); Weikart (1967)	Control	Yes
			2 studies: Blatt (1965); Van de Riet (1972)	TAU	Yes
***Emotional well‐being and social competence***					
Emotional regulation	Assessment of Children's Emotion Skills (ACES; Schultz, 2004)	High score favourable Upper & lower limits: 12–0	1 study: Bierman (2008)	TAU	Yes
	Emotion Regulation Checklist (ERC; Shields, 1997)	High score favourable Upper & lower limits: 32–0	1 study: Blair (2014)		
	Cooper‐Farran Behaviour Rating Scales, Work‐Related Skills sub scales (Cooper, 1991)	High score favourable Upper & lower limits: 147–0	1 study: Farran (2015)		
	Head Toes Knees Shoulders task (Ponitz, 2009)	High score favourable Upper & lower limits: 52–0	1 study: Courtier (2021)		
	Preschool Self‐Regulation Assessment (PSRA; Smith‐Donald, 2007)	High score favourable 28 items	1 study: Raver (2009)		
	International Development and Early Learning Assessment, Self‐regulation subtest (IDELA; Save the Children, 2015)	High score favourable	1 study: Yousafzai (2018)		
Problem behaviour	Problem Behaviour Index (no reference)	Low score favourable	1 study: Abbott‐Shim (2000)	Control	Yes
	Social Skills Improvement System, Problem Behaviours sub scale (SSiS; Flowers, 2008)	Low score favourable Standardised M 100 (SD 15)	1 study: Peisner‐Feinberg (2019)		
	Ypsilanti Rating Scale (Kamii, 1967)	Low score favourable	1 study: Weikart (1967)		
	Challenging Situations Task, Aggressive Responses (CST; Denham, 1994)	Low score favourable	1 study: Bierman (2008)	TAU	Yes
	General Behaviour Problems score based on items from the Strengths & Difficulties Questionnaire (SDQ; Goodman, 2001)	Low score favourable Teachers completed 20 items on a 0 (not true) to 2 (certainly true) scale	1 study: Blair (2014)		
	Behaviour Problems Index (BPI; Zill, 1990)	Low score favourable Upper & lower limits: 52–0	1 study: Hsueh (2014)		
	Social Skils Rating System, Problem Behaviour (SSRS; Gresham, 1990)	30 items based on 3‐point Likert scale Total social skills score sum of 30 items Low score favourable Standardised M 100 (SD 15)	7 studies: PCER (2008), Bright Beginnings & Creative Curriculum; PCER (2008), Creative Curriculm; PCER (2008), Doors to Discovery & Let's Begin; PCER (2008), Language‐Focused Curriculum; PCER (2008), Literacy Express + DLM + OCR Pre‐K; PCER (2008), Project Construct; PCER (2008), Ready, Set, Leap!		
Social skills	Social Skills & Positive Approaches to Learning (SSPAL; Zill, 2003b)	Low score favourable 28‐item teacher‐report measure based on 3‐point Likert scale	1 study: Abbott‐Shim (2000)	Control	No
	Social Skills Improvement System, Social Skills sub scale (SSiS; Flowers, 2008)	High score favourable Standardised M 100 (SD 15)	1 study: Peisner‐Feinberg (2019)		
	Ypsilanti Rating Scale (Kamii, 1967)	High score favourable	1 study: Weikart (1967)		
	Social Competence Scale, Teacher Rating (Fast Track, 2019)	High score favourable 13 items rated on 6‐point Likert scale	1 study: Bierman (2008)	TAU	Yes
	Cooper‐Farran Behaviour Rating Scales, Interpersonal Skills (Cooper, 1991)	High score favourable 21‐item survey based on 7‐point Likert scale Individual score calculated with average of survey items	2 studies: Farran (2015); Hsueh (2014)		
	Teacher Behaviour Rating Scale (TBRS; Landry, 2000)	High score favourable 50 items, 12 sub‐scales items on each sub scale were rated on a 4‐point (1 = rarely occurred to 4 = often occurred) quantity scale and 3‐point quality scale	1 study: Lonigan (2015)		
	Citizenship grades	High score favourable	1 study: Van de Riet (1972)		
	International Development and Early Learning Assessment, Socioemotional subtest (IDELA; Save the Children, 2015)	High score favourable	1 study: Yousafzai (2018)		
	Social Skills Rating System, Social Skills (SSRS; Gresham, 1990)	High score favourable 30 items based on 3‐point Likert scale Total social skills score sum of 30 items Standardised M 100 (SD 15)	7 studies: PCER (2008), Bright Beginnings & Creative Curriculum; PCER (2008), Creative Curriculm; PCER (2008), Doors to Discovery & Let's Begin; PCER (2008), Language‐Focused Curriculum; PCER (2008), Literacy Express + DLM + OCR Pre‐K; PCER (2008), Project Construct; PCER (2008), Ready, Set, Leap!		
***Health development***					
Health‐related outcomes	Health check status Immunization history Dental attendance Healthy eating Exercise	High score favourable	1 study: Abbott‐Shim (2000)	Control	No
***Physical development***					
Perceptual motor skills	Bender Gestalt (error score) (Koppitz, 1964)	Low score favourable	1 study: Van de Riet (1972)	TAU	No
Fine motor skills	International Development and Early Learning Assessment, Fine Motor subtest (IDELA; Save the Children, 2015)	High score favourable	1 study: Yousafzai (2018)		
Gross motor skills	International Development and Early Learning Assessment, Gross Motor subtest (IDELA; Save the Children, 2015)	High score favourable	1 study: Yousafzai (2018)		
***Adverse outcomes***					
None reported					
***Economic costs***					
Cost	Total costs and cost per child (US $)		1 study: Weikart (1967)	Control	No
			1 study: Bierman (2008)	TAU	

Abbreviations: DLM, Dynamic Learning Maps; M, Mean; ORC, open court reading; PCER, Preschool Curriculum Evaluation Research Initiative; Pre‐K, pre‐kindergarten; SD: Standard Deviation; TAU, Treatment as usual; TOLD, Test of Language Development; US $, US dollars.

###### Primary outcomes

###### School readiness

Two studies measured school readiness (Warden, 1988; Yousafzai, 2018). Assessments were administered to intervention and control groups at post‐test at the end of the pre‐kindergarten year (short‐term, up to 12 months). Warden (1988) assessed students again at long‐term follow‐up, 2 years post‐intervention.

###### Adverse effects

None of the studies assessed adverse effects.

###### Secondary outcomes

A number of standardised measures were included across each of the secondary outcome domains. These were categorised into sub domains pertaining to child outcomes, notably academic achievement (general achievement, language, mathematics, phonics, reading, vocabulary), cognitive development (intelligence), emotional well‐being and social competence (emotional functioning, problem behaviour, social skills), health development, and physical development; adverse outcomes (parent stress); and economic costs.

###### Child outcomes

**Academic achievement.** A range of standardised instruments and other measures were used to assess different aspects of academic achievement. These were categorised into a number of different sub‐domains: general achievement; language; mathematics; phonics; reading; and vocabulary.

Four studies reported general academic achievement, measured using standardised tools or teacher‐reported assessments of academic competence, school achievement or reporting of school grades. Assessments were conducted at short‐, medium‐ and long‐term follow‐up. One study used the California Achievement Test (Tiegs, 1957) and academic progress in specific competencies/subject areas were commonly measured using standardised measures including the Social Skills Rating System, Academic Competence scale (SSRS; Gresham, 1990) and Academic Rating Scale (National Center for Education Statistics, 2021).

Language skills were measured at short‐term follow‐up in 10 studies (Table 6), at medium‐term follow‐up in 7 studies, and at long‐term follow‐up three studies. Three studies used the Illinois Test of Psycholinguistic Abilities (ITPA; McCarthy, 1961) but the majority used the TOLD Grammatical Understanding sub scale (Newcomer, 1997).

Nineteen trials measured mathematics competency, at short‐ (Table 6), medium‐ (10 studies) and long‐term follow‐up (5 studies). Measures included: the Arithmetic subtest of the California Achievement Test (Tiegs, 1957); Metropolitan Achievement Test, Computation and Quantitative sub tests (Bixler, 1958); Woodcock Johnson, Qualitative Concepts and Applied Problems sub tests (McGrew, 2001); Test of Early Mathematics Ability, 3rd Edition* (TEMA‐3; Ginsburg, 2003); Stanford Achievement Test, Arithmetic subtest (Kelley, 1952); Academic Rating Scale, Mathematical Thinking (National Center for Education Statistics, 2021); Child Math Assessment (Starkey, 2012); Early Maths Skills score (Zill, 2003a); International Development and Early Learning Assessment, Emergent Mathematics (IDELA; Save the Children, 2015).

Nine trials measured progress in phonics using the Comprehensive Test of Phonological Processing (CTOPP)*, Elision subtest, Kindergarten (Wagner, 1999) and one study used the Evaluation des fonctions cognitives et Apprentissages, Phonologie subtest (Billard, 2012). Children were assessed at short‐ and medium‐term follow‐up (Table 6).

For assessment of reading progress, a range of standardised measures were used and children were assessed at short‐ (15 studies) (Table 6), medium‐ (11 studies) and long‐term follow‐up (6 studies). The Peabody Picture Vocabulary Test (PPVT; Dunn, 1965, 2007) and the Spanish language version (Test de Vocabulario en Imagenes Peabody (TVIP; Dunn, 1986) was used in most studies to assess vocabulary. Children were assessed in 15 trials at short‐ (Table 6), medium‐ (12 studies) and long‐term follow‐up in 5 studies.

**Cognitive development.** Cognitive development was measured in four studies and the same instrument was used by all authors (Stanford‐Binet Intelligence Scale; Terman, 1960) at short‐ and medium‐follow‐up and all four studies assessed children at long‐term follow‐up.

**Emotional well‐being and social competence.** Emotional well‐being and social competence assessments were categorised across three different areas: emotional regulation; problem behaviours; and social skills. Emotional regulation was measured in six studies at short‐term follow‐up using a range of standardised measures (Table 6). Blair 2014 and Farran 2015 assessed children at medium‐term follow‐up and Farran 2015 repeated the assessment at long‐term follow‐up.

Problem behaviours were assessed in 13 trials at short‐term follow‐up, 9 trials at medium‐term follow‐up (Table 6) and one study (Weikart, 1967) assessed children more than 2 years post‐intervention. The Problem Behaviour scale of the Social Skills Rating System (SSRS; Gresham, 1990) was used in the 7 PCER trials. Different measures were used across the remaining six trials that assessed problem behaviours: Abbott‐Shim used the Problem Behaviour Index (no citation given); Bierman (2008) used the Challenging Situations Task; Blair used the General Behaviour Problems scale; Hseuh used the Behaviour Problems Index; Peisner‐Feinberg (2019) used the Social Skills Improvement System (SSiS; Gresham, 2007); and Weikart (1967) used Vinter's Pupil Behaviour Inventory (Vinter, 1966).

Social skills were assessed in 18 trials at short‐term follow‐up. Twelve studies repeated assessments at medium‐term follow‐up (Table 6) and three studies assessed children at long‐term follow‐up (Farran, 2015; Van de Riet, 1972; Weikart, 1967). The Social Skills scale of Gresham's Social Skills Rating System (SSRS; Gresham, 1990) was used across the PCER trials. Peisner‐Feinberg 2019 used a different scale by the same author, the Social Skills sub‐scale of the Social Skills Improvement System (SSiS; Gresham, 2007). Teacher‐report scales were used by Bierman (2008), and Lonigan (2015), and the Cooper‐Farran Behaviour Scales were used in two trials (Farran, 2015; Hsueh, 2014). Yousafzai (2018) used the Socioemotional subtest of the IDELA (Save the Children, 2015).

**Health development.** Only one study reported health‐related outcomes at short‐term follow‐up. Abbott‐Shim (2000) recorded the health check status, immunization history, dental attendance, healthy eating and exercise in the children included in their Head Start study.

**Physical development.** Two studies reported physical development measures of fine and gross motor skills. Yousafzai (2018) used the Fine Motor and Gross Motor sub‐tests of the International Development and Early Learning Assessment (IDEAL; Save the Children, 2015) at short‐term follow‐up and Van de Riet (1972) measured perceptual motor skills using error scores of the Bender Gestalt test for young children (Koppitz, 1964) at long‐term follow‐up.

###### Adverse outcomes: Parental stress

No studies measured parental stress.

###### Economic costs

Two studies analysed total costs and cost per child (Bierman, 2008 and Weikart, 1967).

##### Funding sources

These are listed in the Characteristics of included studies tables. Most studies were funded by agencies in the US federal government. Yousafzai (2018) was funded by the Saving Brains Programme, Grand Challenges Canada and UNICEF.

#### 6.1.3 Excluded studies

We excluded eight studies from this review. Five studies were excluded because they did not meet the duration or intensity specified in the protocol (Gür, 2017; O'Connor, 2014; Pears, 2012; Tzuriel, 1998; Tzuriel, 1999). One study was excluded because it specifically targeted children with special educational needs (Hodges, 1967), and another because it delivered the same intervention to all children in the trial but compared outcomes by family socio‐economic status (Di Lorenzo, 1967). The eighth study was excluded because the Carolina Abecedarian programme was delivered to participants from birth, making it difficult to identify the active treatment for ages 3 to 5 (Ramey, 1974).

##### Studies awaiting classification

We identified five studies awaiting classification. Results were either not reported (Alpern, 1966) or incomplete. Attempts to contact authors were unsuccessful (Table 4). The majority of these trials were also US‐based and one in Sweden (Stukat, 1971). A Montessori curriculum was delivered in one (Karnes, 1969).

Details of these studies can be found in the Characteristics of studies awaiting classification tables.

##### Ongoing studies

We identified two ongoing studies (Howard, 2019 and Yousafzai, 2021). The purpose of the Howard 2019 project is to evaluate the impact of the HighScope Preschool Curriculum (HSPC) on child, teacher, and classroom outcomes in preschool programmes serving children from low‐income backgrounds in the USA. Using a sample of 1600 children, 400 preschool teachers (lead and assistant) and 100 programme preschool centre administrators, researchers will evaluate the efficacy of the curriculum, examine how the curriculum is implemented, and determine the mechanisms through which HSPC affects child outcomes. They will evaluate the impact of HSPC on children's pre‐academic and social behavioural skills at the end of preschool and on early indicators of school achievement at the end of kindergarten. The second study is led by Aisha Yousafzai and is an extension of the Youth Leaders for Early Childhood Assuring Children are Prepared for School (LEAPS) trial using a stepped wedge design. See the Characteristics of ongoing studies table for further details.

### 6.2 Risk of bias in included studies

**Figure 5:**
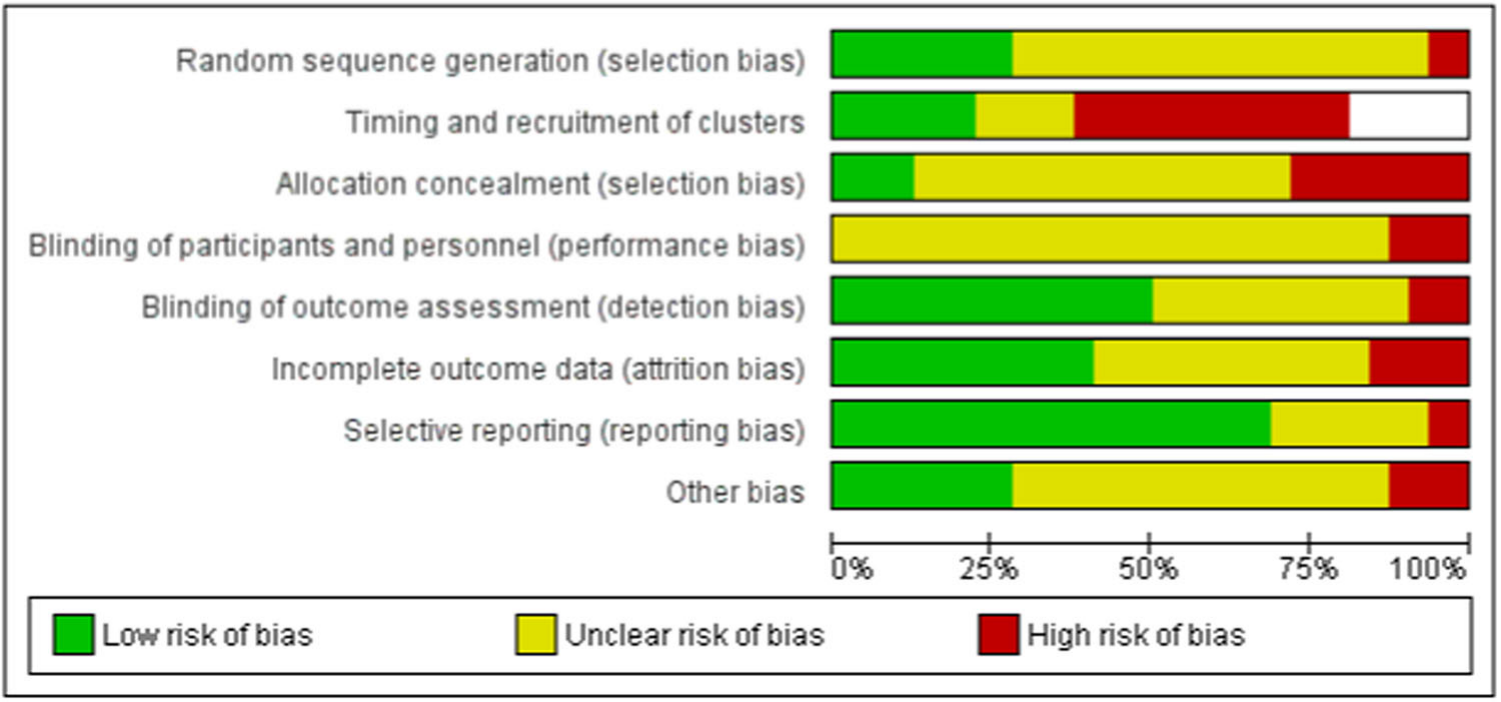
Risk of bias graph Risk of bias in Included Studies.

**Figure 6:**
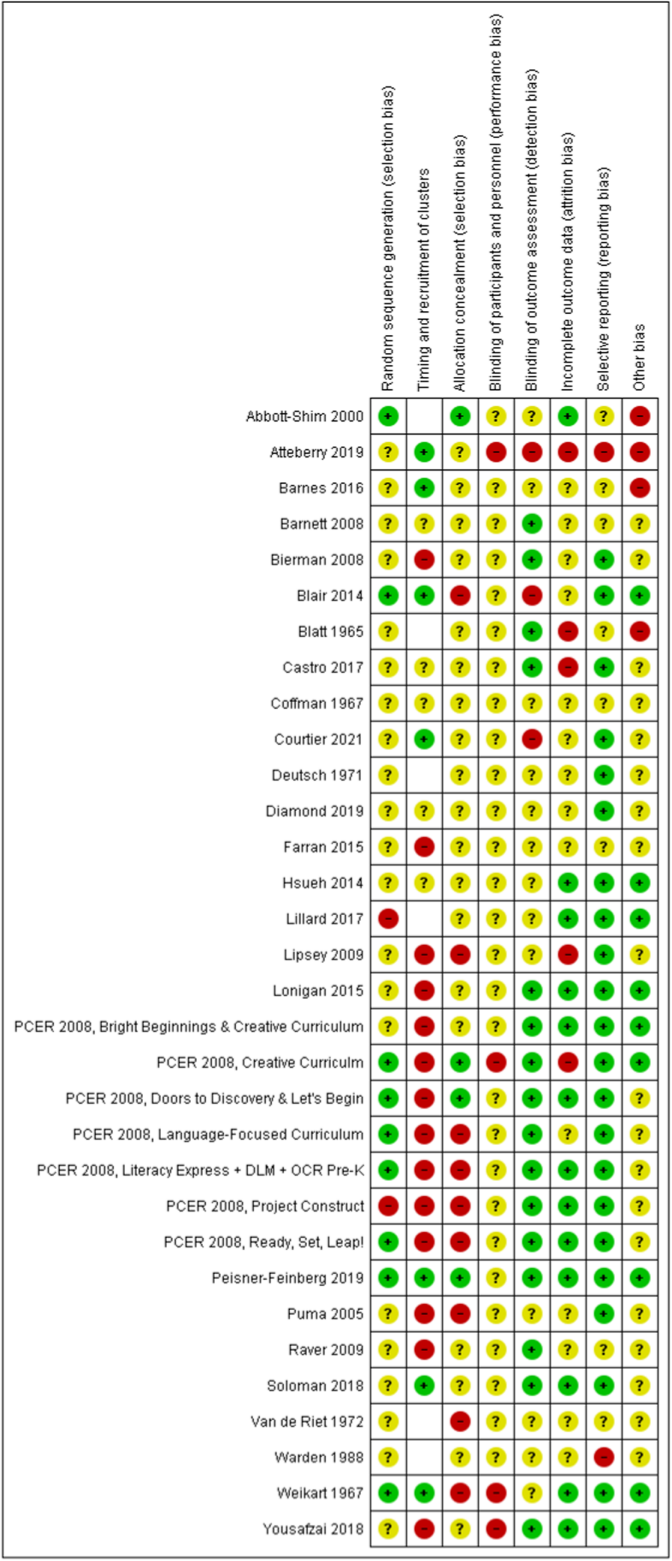
Risk of bias summary. Risk of bias in Included Studies.

Full details of the ‘Risk of bias’ assessment for each study can be found in the Characteristics of included studies tables and is displayed graphically in Figure 5. A ‘Risk of bias’ summary is displayed for each study in Figure 6.

#### 6.2.1 Allocation

##### Random sequence generation

We assessed the risk of selection bias from randomisation to be low in 9 studies (Abbott‐Shim, 2000; Blair, 2014; PCER, 2008, Creative Curriculm; PCER, 2008, Doors to Discovery & Let's Begin; PCER, 2008, Language‐Focused Curriculum; PCER, 2008, Literacy Express + DLM + OCR Pre‐K; PCER, 2008, Ready, Set, Leap!; Peisner‐Feinberg, 2019; Weikart, 1967). Studies were rated as low risk when they had fully outlined the steps taken during randomisation and had taken steps to ensure researchers and participants were blind to the group selection processes. Studies that used random number generators or other random means of group assignment were deemed to be low risk. We assessed two studies to have a high risk of selection bias from poor randomisation (Lillard, 2017; PCER, 2008, Project Construct); this included assignment based on the number of classrooms or children available. The remaining 21 studies did not provide enough detail about the randomisation process and were therefore assessed at unclear risk of bias.

##### Timing and recruitment of clusters

We assessed the risk of bias relating to the timing and recruitment of clusters in the cluster‐RCTs. We assessed 7 studies as being low risk because they either recruited children before settings had been randomised (Atteberry, 2019; Barnes, 2016; Blair, 2014; Courtier, 2021; Peisner‐Feinberg, 2019; Soloman, 2018; Weikart, 1967). Five studies did not provide enough detail and were rated as unclear (Barnett, 2008; Castro, 2017; Coffman, 1967; Diamond, 2019; Hsueh, 2014) and in six studies, this risk of bias domain was not applicable (i.e., RCTs without clusters [Abbott‐Shim, 2000; Blatt, 1965; Deutsch, 1971; Lillard, 2017; Van de Riet, 1972; Warden, 1988]). The remaining 14 studies recruited children after randomisation and were assessed as being high risk.

##### Allocation concealment

We considered the risk of selection bias to be low in four studies across allocation concealment (Abbott‐Shim, 2000; PCER, 2008, Creative Curriculm; PCER, 2008, Doors to Discovery & Let's Begin; Peisner‐Feinberg, 2019). Studies were deemed as low risk when researchers and participants were blinded to group allocation. We assessed 19 studies as having an unclear risk of bias as they did not give sufficient information about how allocation took place. Nine studies were rated at high risk of selection bias: schools were allocated to a particular setting based on the number of classrooms or available local population; three studies used consecutive block randomisation; one study used test scores to randomise (Van de Riet, 1972); Lillard (2017) randomised taking into account neighbourhood, sibling, and staff preferences and; Weikart (1967) included transport availability and sibling attendance in the randomisation process.

#### 6.2.2 Blinding

##### Performance bias

We considered the majority of studies (*k* = 28) to be at unclear risk of performance bias; none of them provided enough detail to provide a confident assessment. Four studies were assessed as high risk. In PCER (2008), Creative Curriculm, the control and intervention groups were housed in the same setting and implementation fidelity data suggest that the control group teachers were doing some of the activities on the Creative Curriculum fidelity checklist. In Atteberry (2019), teachers were not randomised, which increased the risk of performance bias. In Weikart (1967), younger siblings of children already in the project were assigned to the same group, so assignment was by family rather than child. In addition, occasional exchanges of children between groups had to be made because of the inconvenience for half‐day preschool for working mothers and transportation difficulties for some families. Blinding was not possible in Yousafzai (2018) because as part of the community‐based strategy, the LEAPs ECCE centres were signposted in villages and some data collection visits took place near to the centres.

##### Detection bias

Detection bias was judged to be low in half of included studies (*k* = 16). Assessors in these studies had undergone specific training and were blinded to the setting when conducting child assessments. Not enough information was provided in 13 studies and the risk was therefore assessed as unclear. Three studies were assessed as high risk. Assessments were conducted in family homes or neutral settings for control children in Atteberry (2019). Blair (2014) stated that elements of the Tools of the Mind curriculum made it impossible to blind assessors and in Courtier (2021), authors reported that assessors were not blinded.

#### 6.2.3 Incomplete outcome data

Of the 32 studies, we assessed 13 as being at low risk of attrition bias because of very low attrition or attrition that was balanced across the arms of the study. Attrition was well explained where it occurred. Fourteen studies did not provide enough detail and were judged to be unclear risk of bias. We assessed five studies at high risk of bias because they continued to test children no longer part of the trial (Blatt, 1965), attrition was high (19%) and treatment fidelity could not be guaranteed (Castro, 2017) or parental consent to test children was absent in three quarters of the cohort (Lipsey, 2009). There is substantial missing data across both experimental and control groups in Atteberry (2019) and in PCER (2008), Creative Curriculm, 15% of the sample was missing at follow‐up.

#### 6.2.4 Selective reporting

We rated 22 studies at low risk of reporting bias because they reported prespecified outcome measures. Eight studies were deemed to be an unclear risk as they did not provide enough information. Of these eight, four studies did not have protocols making it difficult to judge whether there was reporting bias; we judged these studies to be at unclear risk of bias (Abbott‐Shim, 2000; Blatt, 1965; Raver, 2009; Van de Riet, 1972). Blair (2014) also did not report all outcome measures in their journal article, this was considered unclear risk. We rated two studies at high risk of bias because not all outcomes were reported in the full report (Atteberry, 2019; Warden, 1988).

#### 6.2.5 Other potential sources of bias

We considered four studies to be at high risk of bias. Abbott‐Shim (2000) reported study design decisions that were made in close partnership with the developers of the Head Start programme. The sample was skewed towards the intervention group in Atteberry (2019), which they felt may introduce bias that they could not account for. In Barnes (2016), the TAU condition differed across the two states, with teachers in Texas delivering twice the amount of maths teaching compared to the classrooms in California. Blatt (1965) made changes to the methodology during the course of the trial from ‘what was planned before the formal phase of the investigation started but we will try to make it clear that the vagaries of field research often caused us to depart from our plans to such an extent that the departure was more significant than the original plan itself’ (Blatt, 1965, p. 63). Twenty studies were assessed as having unclear risk of other bias, and eight studies were rated as low.

**Figure 7:**
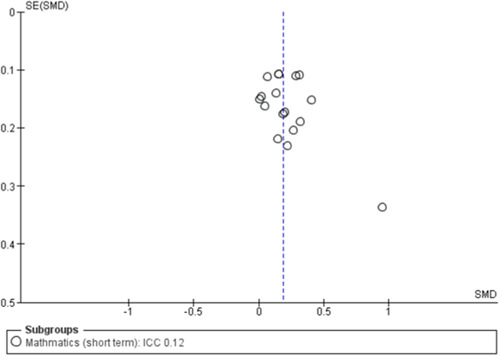
Funnel plot of comparison: 5 Funnel Plot Data, outcome: 5.1 Academic achievement (Maths short term).

**Figure 8:**
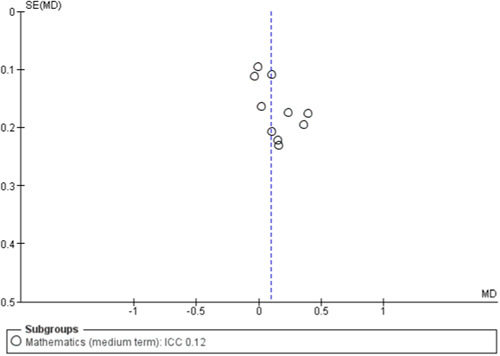
Funnel plot of comparison: 5 Funnel Plot Data, outcome: 5.2 Academic achievement: maths (medium term).

**Figure 9:**
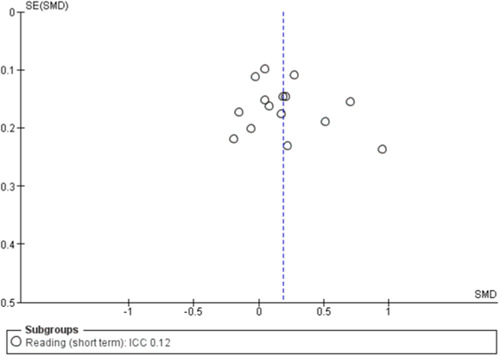
Funnel plot of comparison: 5 Funnel Plot Data, outcome: 5.3 Academic achievement (Reading short term).

**Figure 10:**
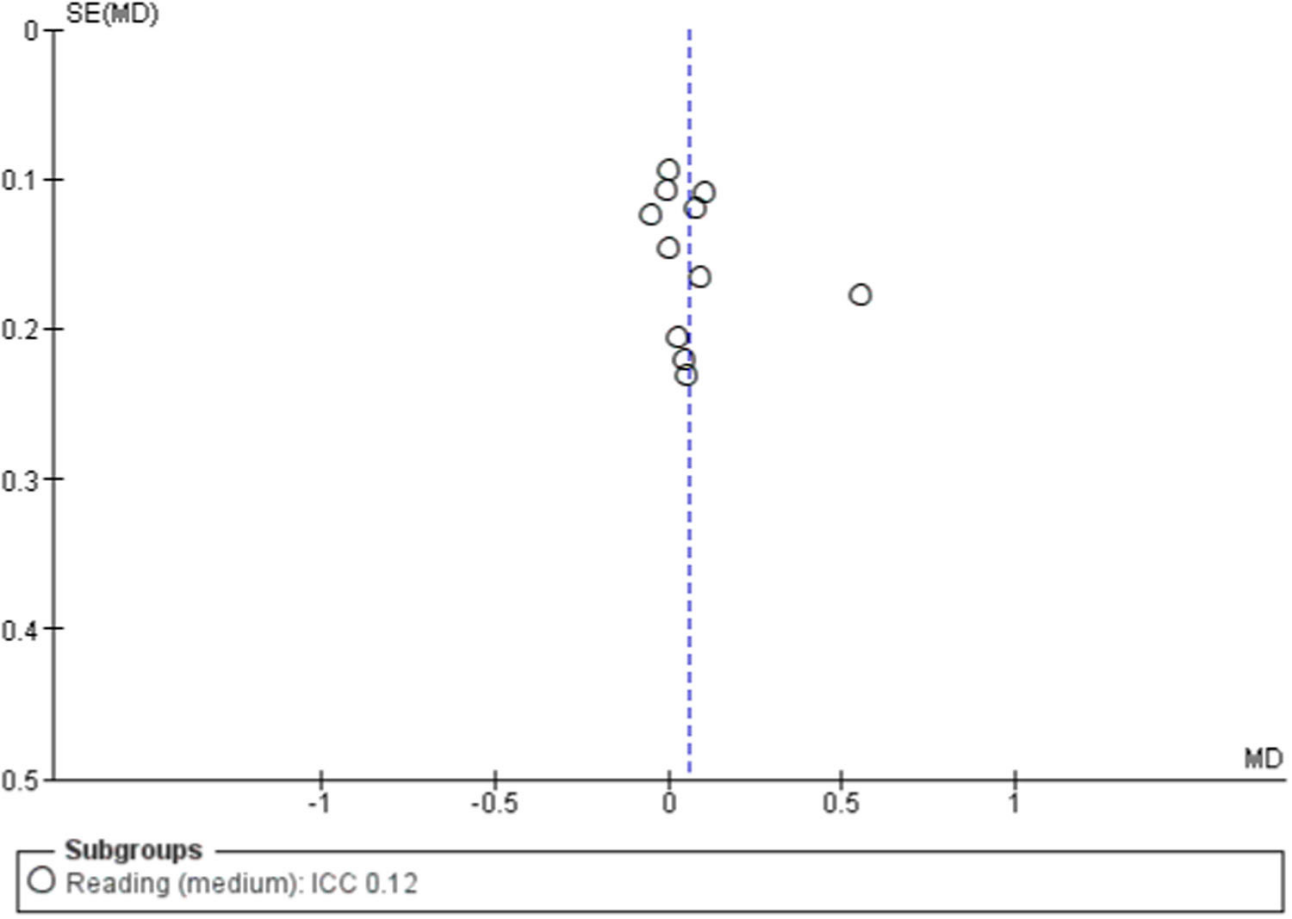
Funnel plot of comparison: 5 Funnel Plot Data, outcome: 5.4 Academic achievement (Reading medium term).

**Figure 11:**
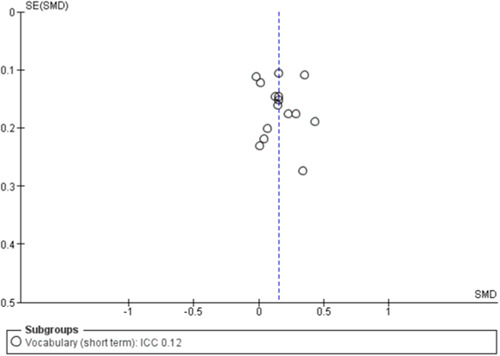
Funnel plot of comparison: 5 Funnel Plot Data, outcome: 5.5 Academic achievement (Vocabulary short term).

**Figure 12:**
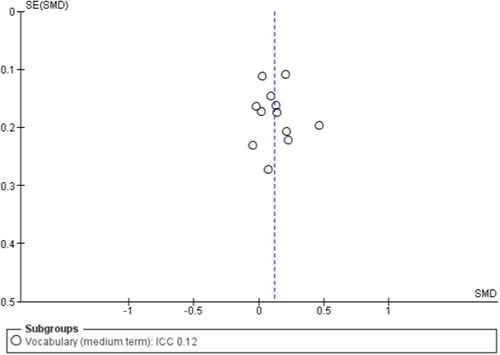
Funnel plot of comparison: 5 Funnel Plot Data, outcome: 5.6 Academic achievement (Vocabulary medium term).

**Figure 13:**
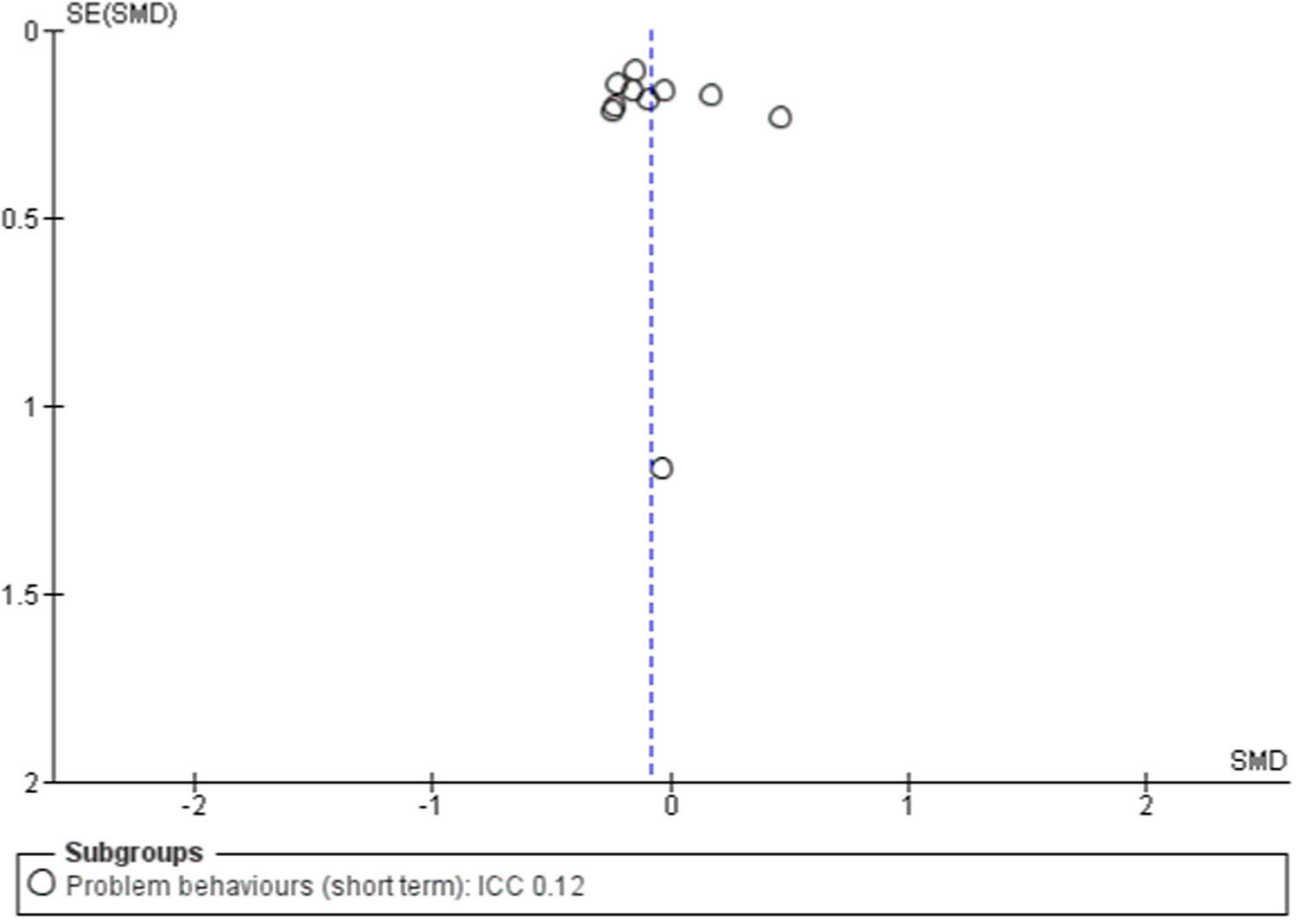
Funnel plot of comparison: 5 Funnel Plot Data, outcome: 5.7 Emotional well‐being & social competence (Problem Behaviours short term).

**Figure 14:**
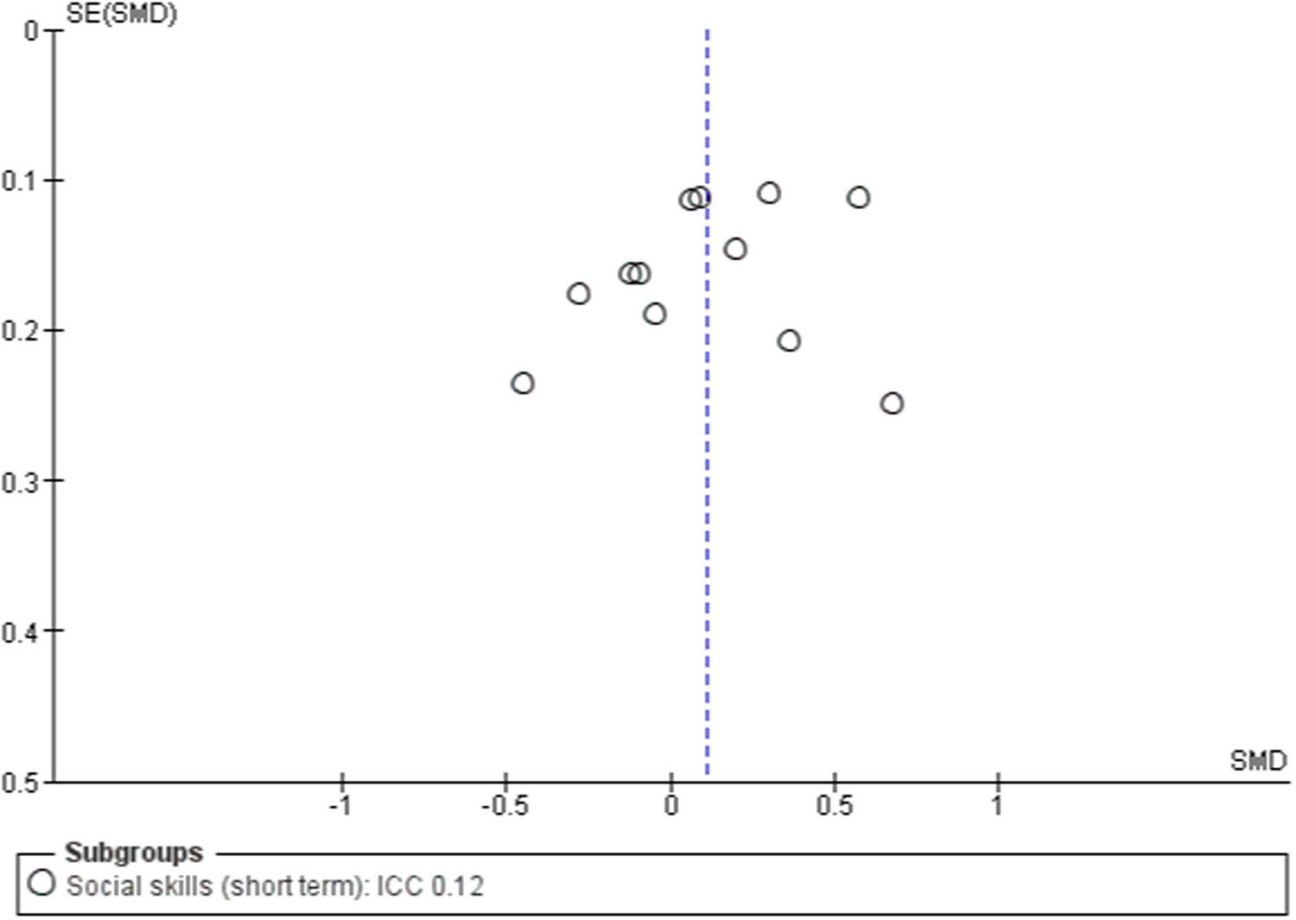
Funnel plot of comparison: 5 Funnel Plot Data, outcome: 5.9 Emotional well‐being & social competence (Social Skills short term).

For the 8 meta‐analyses that included 10 or more studies, funnel plots were inspected (Figures 7, 8, 9, 10, 11, 12, 13, 14). None of the funnel plots showed severe asymmetry that could potentially indicate publication or other forms of bias. Of the included studies, nine were deemed to be low risk of other sources of bias (Blair, 2014; Hsueh, 2014; Lillard, 2017; Lonigan, 2015; PCER, 2008, Bright Beginnings & Creative Curriculum; PCER, 2008, Creative Curriculm; Peisner‐Feinberg, 2019; Weikart, 1967; Yousafzai, 2018). The remaining 19 studies were classed as unclear in this domain, because they did not provide enough information; one study, Bierman (2008), was deemed unclear in this domain as they included some financial rewards for taking part in the research.

##### Heterogeneity

###### Methodological heterogeneity

We used a random‐effects models to account for the expected heterogeneity. There was no unexpected variability in the methodological heterogeneity in each included study. All included studies made use of a RCT design and all had similar levels of low or unclear risk of bias. Allocation bias was rated as high risk in nine studies and the certainty of evidence was downgraded to account for this.

The results are presented for the two main comparisons across the primary and secondary outcomes: intervention verus control and intervention versus treatment as usual. A number of standardised measures were included across each of the secondary outcome domains. These were categorised into sub domains of the secondary outcome measures. These were: academic achievement (general achievement, language, mathematics, phonics, reading, vocabulary); cognitive development (intelligence); emotional well‐being and social competence (emotional regulation, problem behaviour, social skills); adverse outcomes (parent stress); health development; physical development; and economic costs.

### 6.3 Effects of interventions

A summary of the results of the meta‐analyses are detailed below. Results from single studies that were not suitable to combine with other studies in a meta‐analysis are also reported. Some measures were not combined either because the time point did not synchronise or the outcome variable was too different (e.g., parent report vs. outcome assessor report, or measured different domains within an outcome).

#### 6.3.1 Comparison 1. Centre‐based early education interventions for improving school readiness versus no intervention

Where data were available, results are reported at short‐term (up to 12 months), medium‐term (1 to 2 years) and long‐term follow‐up (over 2 years).

##### Primary outcomes

No studies reported the primary outcomes of school readiness or adverse effects.

##### Secondary outcomes

###### Academic achievement

*General achievement* was measured in one study. There was little to no difference between centre‐based early education interventions compared to no intervention at long‐term follow‐up (MD: 16.10, 95% CI: −10.23 to 42.43; *p* = 0.23; 1 study, 95 participants; Analysis 1.1).

*Language* was assessed in one study. There was little to no difference between centre‐based early education interventions compared to no intervention at short‐term follow‐up (MD: 0.12, 95% CI: −0.22 to 0.46; *p* = .49; 1 study, 117 participants; Analysis 1.1) and long‐term follow‐up (MD: 0.23, 95% CI: −0.32 to 0.78; *p* = 0.42; 1 study, 102 participants).

*Mathematics* at long‐term follow‐up was reported in two studies. A small effect in favour of centre‐based interventions was observed; albeit, there is uncertainty around the exact nature of the effect as the confidence intervals were wide and dipped slightly below 0 (MD: 0.24, 95% CI: −0.01 to 0.50; *p* = 0.06; Tau² = 0.00; *I*² = 0%; 2 studies, 326 participants; Analysis 1.2).

*Reading* at long‐term follow‐up was assessed by two studies demonstrating no clear difference between centre‐based interventions and no intervention (SMD: 0.08, 95% CI: −0.16 to 0.31; *p* = 0.52; Tau² = 0.00; *I*² = 0%; 2 studies, 348 participants; Analysis 1.3), with CIs spanning both harmful and beneficial effects.

*Vocabulary* three studies demonstrated that centre‐based interventions slightly increased vocabulary compared to no intervention at short‐term follow‐up (SMD: 0.23, 95% CI: 0.09 to 0.37; *p* = 0.001; Tau² = 0.00; *I*² = 0%; 3 studies, 1313 participants; Analysis 1.3). The effect marginally increased at medium‐term follow‐up in two studies (MD: 0.42, 95% CI: 0.27 to 0.57; *p* < 0.00001; Tau² = 0.00; *I*² = 0%; 2 studies, 779 participants; Analysis 1.2). Gains were sustained at long‐term follow‐up for centre‐based interventions (MD: 0.35, 95% CI: 0.02 to 0.68; *p* = 0.04; Tau² = 0.02; *I*² = 33%; 2 studies, 364 participants; Analysis 1.2), the confidence intervals were quite wide and covered small through to medium sized effects.

###### Cognitive development

Intelligence was measured in two studies. Based on the certainty of the evidence and the effect size there appeared to be little to no difference between centre‐based interventions at no intervention at short‐term (MD: 3.47, 95% CI: 1.25 to 5.69; *p* = 0.002; Tau^2^ = 0.00; *I*² = 0%; 2 studies, 702 participants; Analysis 1.4), medium‐term (MD: 5.44, 95% CI: −1.00 to 11.87; *p* = 0.10; Tau² = 16.51; *I*² = 73%; 2 studies, 675 participants; Analysis 1.4) and long‐term follow‐up (MD: 3.28, 95% CI: 0.23 to 6.34; *p* = 0.04; Tau²  = 0.00; *I*² = 0%; 2 studies, 361 participants; low‐certainty evidence; Analysis 1.4). Substantial heterogeneity was observed at medium‐term follow‐up.

###### Emotional well‐being and social competence

*Problem behaviour* For problem behaviour at short‐term follow‐up evidence suggests there is little to no difference between centre‐based interventions compared to no intervention (SMD: −0.14, 95% CI: −0.35 to 0.08; *p* = 0.21; Tau² = 0.01; *I*² = 23%; 3 studies, 632 participants; Analysis 1.5).

*Social skills* were assessed using different measures and showed there may be no clear difference being observed between centre‐based interventions compared to no intervention at short‐term follow‐up (SMD: −0.11, 95% CI: −0.54 to 0.33; *p* = 0.63; Tau² = 0.10; *I*² = 71%; 3 studies, 632 participants; low certainty evidence; Analysis 1.6).

###### Health development

One study (Abbott‐Shim, 2000), measured health‐related outcomes, (1 study, 142 participants). This was based on a parent‐report health questionnaire and reported as a percentage total. Parents of children in the intervention group reported higher positive engagement in health services compared to the wait list control. Differences were reported between health‐related outcomes in the centre‐based intervention compared to the wait‐list control group. 100% of parents reported having taken their child for: a well care check up (compared to 7.7% of the control group); growth (74.4% control group), hearing (82.1% control group), vision (82.1% control group) and urine (64.9% control group) health screenings; 100% of the intervention group had a record of their child's immunisations compared to 10.3% of the control group; and 100% of the intervention group were up‐to‐date with their child's immunisation programme compared to 2.6% of the control group. 88.6% of the children in the intervention had had a dental examination in the previous 12 months compared to 48.7% in the wait‐list control group. The evidence is very uncertain about the effect of the intervention based on data from a single study.

###### Economic costs

The children recruited in the Weikart (1967) study have been followed up for an extensive period, with the last data sweep gathered when they were 50 years old. A wide array of rich data has been collected over the years and more complex modelling of cost–benefit analyses has been conducted taking into account a range of different educational, crime, and employment outcomes. Based on a 1993 publication, with most participants attending the programme for two school years, the average cost per participant was USD 12,356. The authors reported that this reflected a taxpayer return on investment of USD 7.16 on the dollar. In 2013, the return to society was estimated at USD 341,732 per participant on an investment of USD 20,019 per participant (USD 11,273 per participant per year), representing USD 16.14 per dollar invested (Belfield, 2006). 80% of this return was spent on the general public, 20% on each participant. 88% of the return came from crime savings, the remaining 12% from education, welfare and additional taxes based on higher earnings. Male participants cost the public 41% less in crime costs per person, USD 967,420 less over their lifetimes. Preschool participants earned 14% more per person than those who did not attend the preschool programme, equating to USD 206,567 (based on the 2013 USD rate) more over their lifetimes.

No studies assessed physical development or adverse outcomes: parent stress.

#### 6.3.2 Comparison 2. Centre‐based early education interventions for improving school readiness versus treatment as usual

##### Primary outcomes

###### School readiness

The evidence is very uncertain about the effect of centre‐based interventions on school readiness compared to treatment as usual at short‐term follow‐up (SMD: 1.17, 95% CI: −0.61 to 2.95; *p* = 0.20; Tau^2^ = 1.57; *I* ^2^ = 95%; 2 studies, 374 participants; very low certainty evidence; Analysis 2.1).

###### Adverse effects

No adverse effects were reported.

##### Secondary outcomes

###### Academic achievement

*General achievement* The evidence suggests that based on the certainty of the evidence and the effect size there is little to no difference in general achievement in centre‐based interventions compared to treatment as usual at long‐term follow‐up (SMD: 0.73, 95% CI: 0.22 to 1.24; *p* = 0.005; Tau² = 0.07; *I*² = 50%; 2 studies, 136 participants; Analysis 2.3).

*Language skills* were assessed in 9 studies (Analysis 2.3). The pooled analysis showed no clear difference between enriched centre‐based interventions and TAU at short (SMD: 0.06, 95% CI: −0.05 to 0.17; *p* = 0.31; Tau² = 0.00; *I*² = 0%; 9 studies, 2058 participants) and medium‐term follow‐up (one to 2 years) (MD: 0.06, 95% CI: −0.08 to 0.20; *p* = 0.37; Tau² = 0.00; *I*² = 0%; 6 studies, 1549 participants; Analysis 2.2). Two studies reported findings at long‐term follow‐up and also observed little to no difference with a lack of precision (MD: 0.39, 95% CI: −0.30 to 1.08; *p* = 0.27; Tau² = 0.19; *I*² = 74%; 2 studies, 137 participants; Analysis 2.2).

*Mathematics* was assessed in 17 studies (Analysis 2.3) at short‐term follow‐up with pooled analysis showing slight improvement in enriched centre‐based interventions compared with TAU (SMD: 0.19, 95% CI: 0.12 to 0.26; *p* < 0.00001; Tau² = 0.00; *I*² = 0%; 17 studies, 6590 participants). Ten studies assessed maths at medium‐term follow‐up, favouring enriched centre‐based interventions compared to TAU but the effect size remained small, albeit the CIs highlight that the effect size may range from negligible to small (MD: 0.10, 95% CI: 0.01 to 0.19; *p* = 0.04; Tau² = 0.00; *I*² = 0%; 10 studies, 3132 participants; Analysis 2.2). Three studies reported outcomes at long‐term follow‐up with no clear difference between enriched centre‐based interventions versus TAU and considerable heterogeneity (SMD: 0.57, 95% CI: −0.10 to 1.24; *p* = 0.10; Tau² = 0.31; *I*² = 91%; 3 studies, 975 participants; Analysis 2.3).

*Phonics* was measured in nine studies (Analysis 2.2). No clear differences were observed between centre‐based interventions and TAU at short‐term follow‐up (MD: 0.12, 95% CI: −0.01 to 0.24; *p* = 0.07; Tau² = 0.01; *I*² = 18%; 9 studies, 2237 participants) but there was little to no difference at medium‐term follow‐up (MD: 0.04, 95% CI: −0.10 to 0.18; *p* = 0.59; Tau² = 0.00; *I*² = 0%; 7 studies, 1554 participants; Analysis 2.2).

*Reading* Data was pooled from 15 studies at short‐term follow‐up and showed that centre‐based interventions slightly increased reading compared to TAU (SMD: 0.18, 95% CI: 0.05 to 0.32; *p* = 0.007; Tau² = 0.04; *I*² = 65%; 15 studies, 5081 participants; Analysis 2.2), slight effect at medium‐term follow‐up (MD: 0.06, 95% CI: −0.02 to 0.14; *p* = 0.17; Tau² = 0.00; *I*² = 0%; 11 studies, 3470 participants; Analysis 2.2). At long‐term follow‐up, a medium effect size favouring enriched centre‐based interventions versus TAU was found, however, the confidence intervals showed there was a lack of precision in the data and the heterogeneity was considerable (SMD: 0.56, 95% CI: −0.08 to 1.21; *p* = 0.09; Tau² = 0.39; *I*² = 91%; 4 studies, 1034 participants; Analysis 2.2).

*Vocabulary* measures pooled analysis of 15 studies (Analysis 2.3) showed a small effect favouring the enriched centre‐based interventions at short‐term follow‐up (SMD: 0.16, 95% CI: 0.08 to 0.23; Tau² = 0.00; *p* < 0.00001; *I*² = 0%; 15 studies, 5114 participants). Measures were repeated at medium‐term follow‐up in 12 studies; pooled analysis of these yielded a similar effect size at medium term (SMD: 0.12, 95% CI: 0.03 to 0.21; Tau² = 0.00; *p* = 0.01; *I*² = 0%; 12 studies, 3529 participants). Three trials assessed vocabulary at long‐term follow‐up and found no clear difference between the intervention and TAU groups (SMD: 0.11, 95% −0.06 to 0.29; Tau² = 0.00; *p* = 0.21; *I*² = 0%; 3 studies, 966 participants).

###### Cognitive development

Pooled analysis from two studies shows there may be no clear evidence of a difference with wide confidence intervals between centre‐based interventions and TAU (MD: 9.34, 95% CI: −6.64 to 25.32; Tau² = 121.84; *p* = 0.25; *I*² = 92%; 2 studies, 136 participants; very low certainty evidence; Analysis 2.4). There was considerable heterogeneity and there was a lack of precision in the data.

###### Emotional well‐being and social competence

*Emotional regulation* A meta analysis of 6 studies showed a small increase in emotional regulation at short‐term follow‐up in favour of enriched centre‐based interventions (SMD: 0.17, 95% CI: 0.06 to 0.27; Tau² = 0.00; *p* = 0.001; *I*² = 5%; 6 studies, 2796 participants; Analysis 2.5). Two studies repeated the measures at medium‐term follow‐up and no clear difference between enriched centre‐based interventions and TAU (SMD: 0.03, 95% CI: −0.12 to 0.18; Tau² = 0.00; *p* = 0.68; *I*² = 0%; 2 studies, 1368 participants; Analysis 2.5).

*Problem behaviours* Pooled analysis of 10 studies showed no clear difference in problem behaviours at short‐term follow‐up (SMD: −0.09, CI: 95% −0.20 to 0.03; Tau² = 0.00; *p* = 0.15; *I*² = 11%; 10 studies; 3536 participants; moderate‐certainty evidence; Analysis 2.6) or at medium‐term follow‐up (SMD: 0.02, 95% CI: −0.09 to 0.14; Tau² = 0.00; *p* = 0.68; *I*² = 0%; 9 studies, 2199 participants; Analysis 2.6) in enriched centre‐based interventions compared with TAU.

*Social skills* A meta‐analysis of 12 studies demonstrated there may be no clear difference between enriched centre‐based interventions and TAU at short‐term follow‐up (SMD: 0.11, 95% CI: −0.05 to 0.28; Tau² = 0.06; *p* = 0.19; *I*² = 73%; 12 studies, 4806 participants; low certainty evidence; Analysis 2.5), medium‐term (SMD: 0.07, 95% CI: −0.04 to 0.18; Tau² = 0.00; *p* = 0.22; *I*² = 0%; 9 studies, 2451 participants), and long‐term follow‐up (SMD: 0.21, 95% CI: −0.28 to 0.71; Tau² = 0.10; *p* = 0.39; *I*² = 74%; 2 studies, 856 participants).

###### Physical development

Two studies reported physical development measures and showed that there is likely no clear differences between enriched centre‐based interventions compared to the TAU control group in fine motor skills (MD: 0.80, 95% CI: −1.11 to 2.71; *p* = 0.41; 1 study, 334 participants; moderate certainty evidence; Analysis 2.7) and gross motor skills (MD: 1.60, 95% CI: −0.70 to 3.90; *p* = 0.17; 1 study, 334 participants) at short‐term follow‐up in Yousafzai (2018). Perceptual motor skills were measured at long‐term follow‐up by Van de Riet (1972), errors were reduced in the enriched centre‐based interventions compared to TAU (MD: −2.47, 95% CI: −3.86 to −1.08; *p* = 0.0005; 1 study, 78 participants; Analysis 2.8). The confidence intervals showed there was a lack of precision in the data.

###### Economic costs

Bierman (2008) calculated overall costs and divided the sum by all the children participating to calculate the per participant costs. Based on 2008 US dollars, per child, the point estimate was approximately USD 191 (range USD 169 to USD 391). Total direct costs were calculated over a 2‐year period to reflect the higher set‐up costs in Year 1 of the implementation. Total costs for Year 1 were USD130,263, falling to USD74,624 in Year 2.

No studies reported on adverse outcomes: parent stress or health development.

## 7 DISCUSSION

### 7.1 Summary of main results

We identified 32 trials (involving 16,899 individual children) that assessed the effectiveness of centre‐based school readiness programmes designed to prepare children aged between 3 and 7 years for starting formal education. Four studies compared school readiness interventions with no treatment controls. Twenty‐two trials compared an enriched school curriculum to TAU. Despite school readiness being the main objective in the intervention, the main outcome (school readiness) was only measured in two studies and none of the studies reported adverse effects. Data were pooled, and very low certainty evidence suggests that there is little or no difference between an enriched curriculum compared to TAU. The sample size was small and there was considerable heterogeneity in the study designs making it difficult to reach firm recommendations, with the evidence downgraded to very low certainty.

**Comparison 1**

School readiness was not measured in any of the included studies in this comparison.

We found little to no difference between centre‐based interventions and no intervention controls across most of the academic achievement outcomes at all time points. All data had a degree of imprecision and inconsistency with wide confidence intervals and small sample sizes. Only in assessments of vocabulary, were slight increases observed at short‐ and medium‐term follow‐up in centre‐based interventions compared to no intervention control; these comparisons typically had a larger sample size potentially reducing the levels of imprecision.

The evidence of cognitive development was of low certainty, having similar levels of imprecision and inconsistency to the academic achievement data. Again, little to no difference was observed between centre‐based interventions compared to no treatment controls.

Little to no differences were observed over time across the different categories of emotional well‐being and social competence (problem behaviours and social skills).

Only one study examined health development outcomes but only narrative analysis was possible with this data and no conclusions on the effectiveness of this intervention can be drawn.

Bierman 2008 calculated overall costs and divided the sum by all the children participating to calculate the per participant costs. Based on 2008 US dollars (USD), per child, the point estimate was approximately USD 191 (range USD 169 to USD 391). Total direct costs were calculated over a 2‐year period to reflect the higher set‐up costs in Year 1 of the implementation. Total costs for Year 1 were USD 130,263, falling to USD 74,624 in Year 2.

No studies reported on adverse outcomes (parent stress).

**Comparison 2**

Despite school readiness being the main objective in the intervention, the main outcome (school readiness) was only measured in two studies but the evidence was very uncertain about the effectiveness of the intervention. None of the studies reported adverse effects. There were little to no clear differences in academic achievement outcomes measuring general achievement, language skills or phonics. There was evidence that the intervention slightly increased mathematics and vocabulary at short‐ and medium‐term follow up. There were also short‐term gains in reading in the intervention group compared to TAU.

Centre‐based interventions slightly increased emotional regulation at short‐term compared to TAU however, the other measures of emotional well‐being and social competence showed little to no differences over time across the three different categories of emotional regulation, problem behaviours and social skills.

Two studies reported physical development measures. Little to no difference was reported in enriched centre‐based interventions compared to the TAU control group in fine motor skills, gross motor skills and perceptual motor skills at short‐term follow‐up.

The evidence suggests that attending a centre‐based preschool programme may slightly increase children's early literacy and numeracy skills and promote emotional regulation helpful for beginning formal education, but any minimal gains are not sustained at long term follow‐up. This review was unable to determine what specific factors lead to increased school readiness. Elements we considered central to preparing children for school did not form part of the assessment battery and therefore some of the more complex concepts relating to school readiness were not measured. Considering the investment in preschool education, more research is needed to assess which elements of pre‐school programmes benefit children most. Further data on a range of outcomes, including the health and physical development of children, attachment, parent and community intervention components and economic analyses would help inform future planning and development.

### 7.2 Overall completeness and applicability of evidence

All of the studies included children between the ages of 3 and 7 with the majority including children in a 1‐year age range. The interventions included in the review were typically ‘enriched’ school readiness curricula that were delivered for at least 3 h per day, four + days per week over a period of around 7 months. All studies included a randomly selected intervention group and in a number of cases this was done at the classroom or school level. There was a lack of research on some of the core competencies considered important for preparing a child for starting school. These include physical and health development (fine and gross motor skills, physical play, healthy growth and family engagement with appropriate public health services), attachment, approaches to learning (curiosity, creativity, independence), and social and emotional development (interacting with others and self‐regulation). Although some studies did measure emotional regulation, problem behaviours and social skills, not all did. Only one study examined physical health, one other measured fine and gross motor skills, and a third considered perceptual motor skills. The focus instead concentrates on the ‘academic’ side of education and success in achievement measures in maths, language, literacy and intelligence. Another consideration is the length of time typical follow‐up assessments were conducted and understanding whether these are long enough to observe sustained effects of an intervention. The majority of studies followed‐up pupils up to 1 year post‐intervention and this may not be long enough to measure if gains are sustained over time. The majority of the studies were conducted in the USA; consideration must be taken of any cultural, educational, legal and healthcare differences compared to other jurisdictions. Only one study was conducted in a low‐middle income country (Pakistan; Yousafzai, 2018). A range of socio‐economic areas were targeted with many studies specifically taking part in areas of socio‐economic deprivation. As such, generalisability to other countries and contexts cannot be assumed.

### 7.3 Quality of the evidence

For the outcomes that we included in our summary of findings tables, we assessed the certainty of evidence as very low, low and moderate. It is important to note that school readiness is a complex concept and includes a series of competencies that were not measured or measured in different ways. We assessed the instruments used and selected studies for meta‐analysis that attempted to measure very similar concepts using robust measures. Nonetheless, this approach still presents some challenges for reporting clinically and educationally relevant data.

#### 7.3.1 Centre‐based early education interventions for improving school readiness versus no intervention

The emotional well‐being and social competence and health development outcome measures were downgraded by two levels to low certainty for imprecision because of the very wide CIs and inconsistency for substantial heterogeneity (*I*² = 71%). The cognitive development outcome was downgraded by two levels to low certainty (Summary of findings Table 1). Evidence was downgraded by one level because the risk of bias was unclear or high risk in at least one of the included studies. The outcome was also downgraded one level for imprecision because the optimal information size was <400, and the CIs included both appreciable benefit and harm (SMD ± 0.05). Further research is likely to have an important impact in our confidence in the estimate of effect and may change the estimate. For comparisons with no treatment, heterogeneity was substantial for two outcomes: intelligence (medium‐term); and social skills (short‐term).

#### 7.3.2 Centre‐based early education interventions for improving school readiness versus TAU

The school readiness outcome measures were downgraded by three levels to very low certainty due to the small sample size, imprecision and inconsistency due to unexplained heterogeneity (*I*² = 95%). The cognitive development outcome was downgraded by three levels to very‐low certainty, the sample size was small and there was some imprecision because the CIs included both appreciable benefit and harm (SMD ± 0.05) and there was considerable unexplained heterogeneity (*I*² = 73%) and therefore the generalisability of the results cannot be ascertained. The emotional well‐being and social competence outcome measures were downgraded by two levels to low certainty because the risk of bias was unclear or high risk in at least one of the included studies and there was inconsistency (substantial heterogeneity *I*² = 95%). The physical development outcome was downgraded one level to moderate certainty due to sample size and the CIs included both appreciable benefit and harm (SMD ± 0.05) (Summary of findings Table 2). For comparisons with treatment as usual, the between studies variance reached significance for the following outcomes: language (long term), mathematics (long term), reading (short‐term), reading (long term), intelligence (long term), social skills (short‐term), and social skills (long term). The between‐studies variance was also significant for problem behaviours (short‐term), but only when the 0.07 ICC was used. In eight of the analyses that contained significant heterogeneity, the number of studies was too few to consider further exploration via meta‐regression or subanalyses (i.e., below 10; Higgins, 2021, Chapter 12). For the other three analyses with significant heterogeneity, while the number of studies did exceed 10, there was insufficient distribution of the covariates values across studies (Higgins, 2021, Chapter 12) to consider further exploration. Additional covariates were considered including mean child age, parental curriculum component, and second language learners; however, the number of studies meeting these criteria was less than four, and therefore subgroup analyses were not possible.

### 7.4 Potential biases in the review process

Studies which report positive results are more likely to achieve publication, resulting in a potential bias in the review. We made every effort to minimise this potential by implementing our search strategy within a range of bibliographic databases alongside sources of unpublished studies and by contacting authors with published work within the field. In a further attempt to consider publication bias, we searched for published protocols from the included trials to inspect for differences between intended and reported outcomes. The results of eight trials are not included in this review which may be a source of potential bias. We used funnel plots to investigate publication bias.

### 7.5 Agreements and disagreements with other studies or reviews

Our findings are much in line with other published evaluations that have identified short‐ or long‐term gains for school readiness interventions in terms of cognitive development and some academic areas but the effect sizes are not substantial. This may be associated with the difficulty in conducting randomised trials in universal interventions. As with our review, other reviews of Head Start have found long‐term effects for some cognitive outcomes, however, no gains were observed for other important aspects of school readiness (US DHHS, 2022). Our review found that school readiness is not routinely measured. While the High/Scope Perry Preschool Project (Weikart, 1967) has demonstrated long‐term benefits for their cohort of children, other trials have not seen the same effects (Schweinhart, 2013). We found variation in curriculum offered and problems with the fidelity of curriculum implementation, the randomisation and blinding processes along with the lack of a parental component, teacher training and appropriate resourcing may affect the success of programmes or outcomes measured (Schweinhart, 2003). Camilli (2010) conducted a meta‐analysis of 123 studies and concluded that preschool education was beneficial for cognitive outcomes and social skills but found that the provision of additional services were associated with negative gains. Evidence from the What Works Clearinghouse (https://ies.ed.gov/ncee/wwc/) confirmed small or no discernable effects on a range of outcomes relating to the curricula included in a number of the trials (Bright Beginnings, Creative Curriculum, Curiosity Corner, Doors to Discovery, Let's begin with the Letter People, Ready Steady Leap! and Tools of the Mind); however, Literacy Express had a medium to large effect for early literacy and language skills based on three studies. In contrast, a 2010 best evidence synthesis concluded that six programmes demonstrated effectiveness, including Curiosity Corner and Ready, Steady Leap! (Chambers, 2010).

One of the included studies and a landmark trial, the Perry Preschool Project (Weikart, 1967), identified similar short‐term gains in their cohort of children. Weikart and colleagues have spent decades following up this small cohort and causal analysis conducted by the authors suggests that the temporary preschool programme effect can act as a gateway to a longer‐term impact where social skills and positive behaviour underpins future success (Heckman, 2013). Long‐term follow‐up of this cohort of children has revealed higher levels of optimism on high school graduation, higher earnings, home and second car ownership, and greater relationship stability; children in the intervention group were also less likely to receive welfare support or be involved in the criminal justice system compared to the children who did not receive the intervention.

## 8 AUTHORS' CONCLUSIONS

### 8.1 Implications for practice

This systematic review found very low, low and moderate certainty evidence that centre‐based school readiness interventions or enriched school readiness interventions make little to no difference in school readiness outcomes. Interventions may convey marginal benefits for children who do not attend preschool or who receive a standard preschool generic curriculum on specific academic areas such as mathematics, reading and vocabulary but any gains were not sustained over the long‐term. There were a lack of studies measuring school readiness, health development and physical development. No studies assessed adverse effects.

This review has implications for how practice consider the attributes of school readiness, how best to measure them and support children to attain competence in them. How long any gains can be expected to be sustained in this very young population also needs to be explored.

Uncertainty remains about how effective these interventions are over the long term or if they are cost‐effective.

### 8.2 Implications for research

#### 8.2.1 Outcomes

Future studies should consider the concept of school readiness and how best to measure related outcomes. Future studies should attempt to measure outcomes for as long as possible.

#### 8.2.2 Study design

Limitations in the existing evidence are due to studies with small sample sizes, variation in outcome measures and sub‐optimal RCT designs without no treatment control groups. Better reporting of the conduction of trials including information about the randomisation and allocation process, performance and detection bias would also help increase confidence in the certainty of evidence. Designing trials that control for some level of preschool education, this could include seeking caregiver information which would allow for subgroup analyses to explore any differences.

#### 8.2.3 Concept and measurement

The evaluation of the active elements of enriched curricula would be important evidence to gather as well as measuring curricular add‐ons such as home‐based components that involve parents, specialist teacher training or the delivery of manualised programmes.

#### 8.2.4 Setting

More trials conducted outside the USA would help develop the global evidence base.

The results of this systematic review are important to consider globally due to the large differences in the age which young children begin formal education.

More randomised controlled trials that focus on early intervention, both parental intervention and child intervention in the years preceding entry to school would allow for a more thorough consideration of the factors that impact school readiness. A move away from focusing on academic outcomes and more skill‐based outcomes would link in more closely with the concept of school readiness. Studies need to be consistent and direct in terms of their target population, the targeted skill areas and the outcomes measured to allow for a more thorough consideration of the problem.

## DATA AND ANALYSES

**Comparison 1**

**Centre‐based early education interventions for improving school readiness versus no treatment**

**Table:** 

Outcome or subgroup title	No. of studies	No. of participants	Statistical method	Effect size
1.1 Academic achievement (MDs, FE)	1		Mean Difference (IV, Fixed, 95% CI)	Totals not selected
1.1.1 General (long term)	1		Mean Difference (IV, Fixed, 95% CI)	Totals not selected
1.1.2 Language (short‐term): ICC 0.12	1		Mean Difference (IV, Fixed, 95% CI)	Totals not selected
1.1.3 Language (long‐term): ICC 0.12	1		Mean Difference (IV, Fixed, 95% CI)	Totals not selected
1.2 Academic achievement (MDs, RE)	3		Mean Difference (IV, Random, 95% CI)	Subtotals only
1.2.1 Mathmatics (long term): ICC 0.12	2	326	Mean Difference (IV, Random, 95% CI)	0.24 [−0.01, 0.50]
1.2.2 Vocabulary (short term): ICC 0.12	3	1313	Mean Difference (IV, Random, 95% CI)	0.24 [0.09, 0.38]
1.2.3 Vocabulary (medium term): ICC 0.12	2	779	Mean Difference (IV, Random, 95% CI)	0.42 [0.27, 0.57]
1.2.4 Vocabulary (long term): ICC 0.12	2	364	Mean Difference (IV, Random, 95% CI)	0.35 [0.02, 0.68]
1.3 Academic achievement (SMDs)	3		Std. Mean Difference (IV, Random, 95% CI)	Subtotals only
1.3.1 Reading (long term): ICC 0.12	2	348	Std. Mean Difference (IV, Random, 95% CI)	0.08 [−0.16, 0.31]
1.3.2 Vocabulary (short term): ICC 0.12	3	1313	Std. Mean Difference (IV, Random, 95% CI)	0.23 [0.09, 0.37]
1.4 Cognitive development (MDs)	2		Mean Difference (IV, Random, 95% CI)	Subtotals only
1.4.1 Intelligence (short term): ICC 0.12	2	702	Mean Difference (IV, Random, 95% CI)	3.47 [1.25, 5.69]
1.4.2 Intelligence (medium term): ICC 0.12	2	675	Mean Difference (IV, Random, 95% CI)	5.44 [−1.00, 11.87]
1.4.3 Intelligence (long term): ICC 0.12	2	361	Mean Difference (IV, Random, 95% CI)	3.28 [0.23, 6.34]
1.5 Emotional well‐being & social competence: problem behaviours (short term) (SMDs)	3	632	Std. Mean Difference (IV, Random, 95% CI)	−0.14 [−0.35, 0.08]
1.6 Emotional well‐being & social competence: social skills (short term) (SMDs)	3	632	Std. Mean Difference (IV, Random, 95% CI)	−0.11 [−0.54, 0.33]

**Comparison 2**

**Centre‐based early education interventions for improving school readiness versus treatment as usual**

**Table:** 

Outcome or subgroup title	No. of studies	No. of participants	Statistical method	Effect size
2.1 School readiness (short‐term) (SMDs)	2	374	Std. Mean Difference (IV, Random, 95% CI)	1.17 [−0.61, 2.95]
2.2 Academic achievement (MDs)	14		Mean Difference (IV, Random, 95% CI)	Subtotals only
2.2.1 Language (medium term): ICC 0.12	7	1549	Mean Difference (IV, Random, 95% CI)	0.06 [−0.08, 0.20]
2.2.2 Language (long term): ICC 0.12	2	137	Mean Difference (IV, Random, 95% CI)	0.39 [−0.30, 1.08]
2.2.3 Mathematics (medium term): ICC 0.12	10	3050	Mean Difference (IV, Random, 95% CI)	0.10 [0.01, 0.19]
2.2.4 Phonics (short term) ICC 0.12	9	2270	Mean Difference (IV, Random, 95% CI)	0.12 [−0.01, 0.24]
2.2.5 Phonics (medium term) ICC 0.12	7	1554	Mean Difference (IV, Random, 95% CI)	0.04 [−0.10, 0.18]
2.2.6 Reading (medium) ICC 0.12	11	3470	Mean Difference (IV, Random, 95% CI)	0.06 [−0.02, 0.14]
2.3 Academic achievement (SMDs)	20		Std. Mean Difference (IV, Random, 95% CI)	Subtotals only
2.3.1 General (long term): ICC 0.12	2	136	Std. Mean Difference (IV, Random, 95% CI)	0.73 [0.22, 1.24]
2.3.2 Language (short term): ICC 0.12	9	2058	Std. Mean Difference (IV, Random, 95% CI)	0.06 [−0.05, 0.17]
2.3.3 Mathmatics (short term): ICC 0.12	17	6505	Std. Mean Difference (IV, Random, 95% CI)	0.19 [0.12, 0.26]
2.3.4 Mathematics (long term) ICC 0.12	3	975	Std. Mean Difference (IV, Random, 95% CI)	0.57 [−0.10, 1.24]
2.3.5 Reading (short term) ICC 0.12	15	5121	Std. Mean Difference (IV, Random, 95% CI)	0.18 [0.05, 0.32]
2.3.6 Reading (long term) ICC 0.12	4	1034	Std. Mean Difference (IV, Random, 95% CI)	0.56 [−0.08, 1.21]
2.3.7 Vocabulary (short term) ICC 0.12	15	5108	Std. Mean Difference (IV, Random, 95% CI)	0.16 [0.08, 0.23]
2.3.8 Vocabulary (medium term) ICC 0.12	12	3529	Std. Mean Difference (IV, Random, 95% CI)	0.12 [0.03, 0.21]
2.3.9 Vocabulary (long term) ICC 0.12	3	956	Std. Mean Difference (IV, Random, 95% CI)	0.11 [−0.06, 0.29]
2.4 Cognitive development (long‐term) (MDs)	2	136	Mean Difference (IV, Random, 95% CI)	9.34 [−6.64, 25.32]
2.5 Emotional well‐being & social competence (SMDs)	16		Std. Mean Difference (IV, Random, 95% CI)	Subtotals only
2.5.1 Emotional regulation (short term): ICC 0.12	6	2796	Std. Mean Difference (IV, Random, 95% CI)	0.17 [0.06, 0.27]
2.5.2 Emotional regulation (medium term): ICC 0.12	2	1368	Std. Mean Difference (IV, Random, 95% CI)	0.03 [−0.12, 0.18]
2.5.3 Social skills (short term): ICC 0.12	12	4806	Std. Mean Difference (IV, Random, 95% CI)	0.11 [−0.05, 0.28]
2.5.4 Social skills (medium term) ICC 0.12	9	2312	Std. Mean Difference (IV, Random, 95% CI)	0.07 [−0.04, 0.18]
2.5.5 Social skills (long term) ICC 0.12	2	856	Std. Mean Difference (IV, Random, 95% CI)	0.21 [−0.28, 0.71]
2.6 Emotional well‐being & social competence: problem behaviours (SMDs)	10		Std. Mean Difference (IV, Random, 95% CI)	Subtotals only
2.6.1 Problem behaviours (short term): ICC 0.12	10	3536	Std. Mean Difference (IV, Random, 95% CI)	−0.09 [−0.20, 0.03]
2.6.2 Problem behaviours (medium term): ICC 0.12	9	2199	Std. Mean Difference (IV, Random, 95% CI)	0.02 [−0.09, 0.14]
2.7 Physical development (short‐term)	1		Mean Difference (IV, Fixed, 95% CI)	Totals not selected
2.7.1 Fine motor skills (short term)	1		Mean Difference (IV, Fixed, 95% CI)	Totals not selected
2.7.2 Gross motor skills (short term)	1		Mean Difference (IV, Fixed, 95% CI)	Totals not selected
2.8 Physical development: perceptual motor skills (long‐term)	1		Mean Difference (IV, Fixed, 95% CI)	Totals not selected

**Comparison 3**

**Funnel plot data**

**Table:** 

Outcome or subgroup title	No. of studies	No. of participants	Statistical method	Effect size
3.1 Academic achievement: maths (short term)	17		Std. Mean Difference (IV, Random, 95% CI)	Subtotals only
3.1.1 Mathmatics (short term): ICC 0.12	17		Std. Mean Difference (IV, Random, 95% CI)	0.19 [0.12, 0.26]
3.2 Academic achievement: maths (medium term)	10		Mean Difference (IV, Random, 95% CI)	Subtotals only
3.2.1 Mathematics (medium term): ICC 0.12	10		Mean Difference (IV, Random, 95% CI)	0.10 [0.01, 0.19]
3.3 Academic achievement: reading (short term)	15		Std. Mean Difference (IV, Random, 95% CI)	Subtotals only
3.3.1 Reading (short term): ICC 0.12	15		Std. Mean Difference (IV, Random, 95% CI)	0.18 [0.05, 0.32]
3.4 Academic achievement: reading (medium term)	11		Mean Difference (IV, Random, 95% CI)	Subtotals only
3.4.1 Reading (medium): ICC 0.12	11		Mean Difference (IV, Random, 95% CI)	0.06 [−0.02, 0.14]
3.5 Academic achievement: vocabulary (short term)	15		Std. Mean Difference (IV, Random, 95% CI)	Subtotals only
3.5.1 Vocabulary (short term): ICC 0.12	15		Std. Mean Difference (IV, Random, 95% CI)	0.16 [0.08, 0.23]
3.6 Academic achievement: vocabulary (medium term)	12		Std. Mean Difference (IV, Random, 95% CI)	Subtotals only
3.6.1 Vocabulary (medium term): ICC 0.12	12		Std. Mean Difference (IV, Random, 95% CI)	0.12 [0.03, 0.21]
3.7 Emotional well‐being & social competence: problem behaviours (short term)	10		Std. Mean Difference (IV, Random, 95% CI)	Subtotals only
3.7.1 Problem behaviours (short term): ICC 0.12	10		Std. Mean Difference (IV, Random, 95% CI)	−0.09 [−0.20, 0.03]
3.8 Emotional well‐being & social competence: social skills (short term)	12		Std. Mean Difference (IV, Random, 95% CI)	Subtotals only
3.8.1 Social skills (short term): ICC 0.12	12		Std. Mean Difference (IV, Random, 95% CI)	0.11 [−0.05, 0.28]

## CONTRIBUTIONS OF AUTHORS

Geraldine Macdonald (GM) developed the concept of the review, and with CM, designed the review. CM co‐ordinated the review. Title, abstract and full‐text screening was conducted by CM and JR. CM and JR extracted the data from included studies. CM and JR assessed the risk of bias. CM, JR and Julie‐Ann Jordan (J‐AJ) assessed the certainty in the body of evidence and CM and J‐AJ interpreted the data. CM, JR and JAJ conducted the analysis and writing up of the results of the review. CM is the guarantor for the review.

## DECLARATIONS OF INTEREST

Julie‐Ann Jordan is a Research Psychologist within the IMPACT Research Centre, Northern Health and Social Care Trust, Northern Ireland.

Claire McCartan: has declared that she has no conflicts of interest.

Jennifer Roberts: has declared that she has no conflicts of interest.

## SOURCES OF SUPPORT

### 1 Internal sources

- Queen's University Belfast, UK

In‐kind support for Claire McCartan and Jennifer Roberts' time.

### 2 External sources

- Health and Social Care Research and Development Division, Public Health Agency, Northern Ireland, UK

Cochrane Fellowships were awarded to Claire McCartan and Jennifer Roberts to produce this review. The funder had no role in the design, conduct or publication of the review.

## DIFFERENCES BETWEEN PROTOCOL AND REVIEW

### 1 Criteria for considering studies for this review > Types of interventions

The original protocol stated we would compare interventions to no treatment control or treatment as usual. We added wait‐list control as a comparison and studies were screened to include this comparison. Preschool provision and school readiness interventions have almost become universal worldwide, therefore it was necessary to introduce a realistic comparator to attempt to measure their effectiveness.

After advice from one of the authors (JR is a trained primary school teacher and lecturer in Education), we decided to compare ‘enriched curriculum’ versus treatment as usual. Enriched curricula typically offer children experiences beyond the traditional curriculum by expanding subject areas and looking at them in more depth or from a different perspective. This approach actively encourages children to try new experiences and activities that may not strictly fit within the curriculum but are designed to help build life skills, resilience, motivation, team working and social responsibility. Enriched curricula often have access to additional resources, enhanced teacher training and may include a home‐based, parent training component and provide a distinct contrast to standard curriculum. Collecting these data was considered an important element of establishing the research evidence that could usefully inform the design of future interventions.

### 2 Search methods for identification of studies > Electronic searches

We made some changes to the electronic searches over the course of this review.

- CINAHL Plus was removed from the list of sources post‐protocol because of the lack of relevant and unique coverage on this topic.
- The following sources were not included in the top‐up searches for the reasons given.
  ∘ Sociological Abstracts had extensive overlap with ERIC and the Social Services Citation Index.
  ∘ Australian Education Index was no longer available to the team after 2014.
  ∘ DARE was withdrawn from the Cochrane Library before the top‐up searches. We used EPISTEMONIKOS as an alternative source of systematic reviews.
  ∘ ClinicalTrials.com was omitted from the 2014 searches, but searched for all available years in subsequent searches.
  ∘ *The meta*Register of Controlled Trials service (*m*RCT) came under review after 2014. Instead, we searched all years of the ISRCTN registry and WHO ICTRP as an international source of controlled trials.
  ∘ EPPI‐Centre Database of Education Research was last updated in 2007 so there was new content at the time of the top‐up searches.
  ∘ We searched DIVA Theses, Trove Theses, Theses Canada, Theses DART, NARCIS, NDLTD, WorldCat and PQDT Theses up to 2014. These free sources were replaced with ProQuest Dissertations & Theses Global when it became available to us in 2020. This provided a more flexible and efficient means of searching for international dissertations.
- We made changes to the MEDLINE search strategy in 2020 to improve the precision of the search, and adapted the changes for other databases. We also searched for named programmes that we identified in eligible studies found by the first set of searches.

### 3 Data collection and analysis

#### Assessment of risk of bias in included studies

Following advice from the Methods Editor, for cluster‐randomised trials, we made an additional assessment listed as ‘Timing of recruitment of clusters’ based on the advice for dealing with cluster‐RCTs (Higgins, 2012, Chapter 23). For ‘Timing of recruitment of clusters’, we rated RCTs at ‘high’ risk of bias if the studies had recruited the clusters after randomisation and at ‘low’ risk of bias if recruitment occurred before randomisation. For the ‘missing outcome data’ domain, we checked to see if there were any missing clusters as this can also be a cause of bias in cluster RCTs.

#### Data synthesis

In line with recommendations published since the protocol was drafted, we chose to use fixed‐effect models for meta‐analyses with four or fewer studies and random‐effects models when there were more than four studies in the meta‐analyses (e.g., Jackson, 2017; Lin, 2020).

#### Subgroup analysis and investigation of heterogeneity

We had pre‐planned subgroup analyses for intervention intensity and duration, socioeconomic status and English language learners but were unable to conduct these due to insufficient studies. We assessed heterogeneity using the *I* ^2^ statistic as originally planned in the protocol.

## CHARACTERISTICS OF STUDIES

**Characteristics of included studies [ordered by study ID]**

Abbott‐Shim 2000

**Table:** 

***Study characteristics***		
Methods	**Design**: randomised controlled trial	
Participants	**Location/setting**: southern urban setting, USA; Head Start centres Sample size: 142 **Mean age**: intervention group = 55.01 (SD 3.56) months; control group = 54.97 (SD 3.70) months **Sex**: intervention group = 51.5% boys; control group = 65.9% boys **Race/ethnicity**: not reported, although the community is located within an almost entirely African‐American, low‐income, inner city environment	
Interventions	**Intervention (*n* = 82)**: operates a theme‐based curriculum with a wealth of classroom resources providing topic‐specific materials (big books, puzzles, play props) that children may use to develop concepts related to the theme in selected Head Start centres. Minimum 7 months attendance, 8:30 AM–2:30 PM **Control (*n* = 60)**: wait‐list control; 46.3% were in alternative care (21.1% family day care/home, 78.9% centre‐based care), mean hours = 40.4 (SD 7.2). The quality of care for the control group children was not collected.	
Outcomes	Eligible outcomes *Secondary outcomes* Academic achievement, measured with: ∘ Peabody Picture Vocabulary Test – 3rd edition (PPVT‐III; Dunn, 1997) (not reported: full data not available) ∘ Metropolitan Early Childhood Assessment Programme (M‐KIDS Preliteracy Inventory; Nurss, 1995) (not reported: full data not available) ∘ Early Phonemic Awareness Profile (Dickinson, 1997) (not reported: full data not available) Emotional well‐being & social competence, measured with: ∘ The Family & Children's Experiences Survey, parent interview (FACES, 1997) (not reported: full data not available) ∘ Problem Behaviour Index (no reference) ∘ Social Skills & Positive Approach to Learning (SSPAL; Zill, 2003b) Health development, measured with: ∘ Eating nutritious & healthful foods (not reported: full data not available) Ineligible outcomes: none **Timing of outcome measurement**: baseline (September to October 1998), T2 short‐term (January to February 1999), T3 short‐term (March to May 1999)	
Notes	Study start date: January 1998 Study end date: 1999 **Funding source**: Administration on Children, Youth, & Families, US Department of Health & Human Services Conflict(s) of interest: none stated **Comment(s)**: none	

***Risk of bias***		
**Bias**	**Authors' judgement**	**Support for judgement**
Random sequence generation (selection bias)	Low risk	**Comment** Identification numbers were randomly generated, then used to allocate children to experimental or control groups.
Allocation concealment (selection bias)	Low risk	**Quote** ‘All of the HS teachers and family service workers were blind to the design and the random assignment procedures used in this study. The staff employed by the Head Start partner had participated in research over the last 3 years, and their involvement during this study year was no different than other years. They were aware that research was being conducted in the classrooms as part of the partnership, but they did not know about the HS Effectiveness sub‐study in particular’. (p. 10)
Blinding of participants and personnel (performance bias) All outcomes	Unclear risk	**Comment** Not reported in enough detail
Blinding of outcome assessment (detection bias) All outcomes	Unclear risk	**Comment** While measures were administered by trained assessors it does not say if they were blinded or not. Parent reports had potential for a high risk of bias because of self‐report.
Incomplete outcome data (attrition bias) All outcomes	Low risk	**Comment** Efforts to retain as many participants as possible were good. Reasons for attrition are very clear.
Selective reporting (reporting bias)	Unclear risk	**Comment** No protocol to check that all outcomes are reported.
Other bias	High risk	**Quote** ‘The study was conducted in the context of a close partnership with the Head Start Programme. The research design strategy was based, in part, on decisions that were intentionally made within the partnership to satisfy thoughtful input from the Head Start program personnel’ (p. 207)

Atteberry 2019

**Table:** 

***Study characteristics***		
Methods	**Design**: cluster‐randomised controlled trial	
Participants	**Location/setting**: Denver, Colorado, USA Sample size: 226 **Mean age**: total sample = 4.4 years **Sex**: treatment group = 48.2% boys; control group = 49.1% boys **Race/ethnicity**: 74% Hispanic	
Interventions	**Intervention (*n* = 114)**: full day pre‐K, the largest differences between full‐and half‐day are in napping (96 vs. 0 min) and eating (52 vs. 17 min). Full‐day students receive 3.7 h per week of reading instruction (relative to 1.3 h for half day), 2.4 h per week in mathematics (1.0 for half day), and 1.4 h of social studies and maths (0.9 h for half day). Students in full‐day classrooms also receive double the hours per week in nonacademic activities such as visual/performing arts, play (structured and unstructured), and transitions between activities. **Control (*n* = 112)**: half day pre‐K	
Outcomes	Eligible outcomes *Secondary outcomes* Academic achievement, measured with: ∘ Peabody Picture Vocabulary Test – 3rd edition (PPVT‐III; Dunn, 1997) ∘ Teaching Strategies (TS) GOLD Language, Literacy and Math Scores (Heroman, 2010) Cognitive development, measured with: ∘ Early Screening Inventory‐Revised (ESI‐R; Meisels, 1997) ∘ TS GOLD overall score (Heroman, 2010) ∘ TS GOLD Cognitive Score (Heroman, 2010) Emotional well‐being & social competence, measured with: ∘ TS GOLD Social‐Emotional Score (Heroman, 2010) Physical development, measured with: ∘ TS GOLD Physical Development Score (Heroman, 2010) Ineligible outcomes: none **Timing of outcome measurement**: baseline (fall 2016), T1 (spring 2017)	
Notes	Study start date: 2016 Study end date: 2017 **Funding source**: Westminster Public School District Conflict(s) of interest: none reported **Comment(s)**: results were reported as causal effects and we were unable to make contact with the author (Table 1)	

***Risk of bias***		
**Bias**	**Authors' judgement**	**Support for judgement**
Random sequence generation (selection bias)	Unclear risk	**Comment** The block randomisation process is not described.
Timing and recruitment of clusters	Low risk	**Comment** Randomisation was performed after recruitment of participants.
Allocation concealment (selection bias)	Unclear risk	**Comment** Not reported in enough detail
Blinding of participants and personnel (performance bias) All outcomes	High risk	**Quote** ‘teachers were not randomly assigned to full versus half‐day classrooms’ (p. 543)
Blinding of outcome assessment (detection bias) All outcomes	High risk	**Comment** No reference to blinding is made and the study team conducted all assessments including home visiting children or meeting families in neutral settings to conduct assessments who were in the control group.
Incomplete outcome data (attrition bias) All outcomes	High risk	**Comment** There is substantial missing data across both experimental and control groups. The authors used a range of statistical methods to explore the missingness in the data and while they conclude that the missing data is not statistically significant, they treat the outcome measures as exploratory given the high rates of missingness. Missing data may also have an impact on the clusters.
Selective reporting (reporting bias)	High risk	**Comment** Only two outcomes measured are reported
Other bias	High risk	**Quote** ‘In the current study, 86% of children offered a full‐day slot enrolled in WPS compared with 62% of those offered a half‐day slot. Although we have carefully considered the implications of this nonrandom sorting on our findings, we cannot fully account for bias that may be introduced into our analysis here’. (p. 555)

Barnes 2016

**Table:** 

***Study characteristics***		
Methods	**Design**: cluster‐randomised controlled trial	
Participants	**Location/setting**: Texas, California, USA **Sample size**: 541 (95 classrooms) **Mean age**: treatment group 1 = 4.50 (SD 0.27) years; treatment group 2 = 4.53 (SD 0.27) years; control group = 4.46 (SD 0.26) years **Sex**: treatment group 1 = 55.2% boys; treatment group 2 = 50% boys; control group = 52.2% boys **Race/ethnicity**: 71.7% Hispanic, 17.9% African American, 3.7% Mixed/other, 2.8% Unknown, 2.2% Caucasian, 1.7% Asian American	
Interventions	**Intervention 1 (*n* = 181)**: Pre‐kindergarten Mathematics Tutorial (PKMT; Klein, 2004a) + ATT (Attention Intervention). The PKMT programme comprises 20 maths activities that use concrete materials to engage PK children and to support their mathematical learning of concepts related to number, arithmetic, space, geometry, and measurement. It was implemented (4 days/week for 24 weeks). The activities were delivered by a trained tutor to pairs of children in their dominant language outside of the classroom. The intervention was implemented over 24 weeks, with a new maths activity introduced each week (20 weeks). ATT consists of 2 types of attention‐training games designed to improve and sustain attention, respond to stimulus and nurture the associated neural changes that control attention (Rueda, 2005, 2012). **Intervention 2 (*n* = 180)**: PKMT programme; comprises 20 maths activities that use concrete materials to engage PK children and to support their mathematical learning of concepts related to number, arithmetic, space, geometry, and measurement **Control (*n* = 180)**: treatment as usual; children did not receive either the tutorial maths intervention or attention training	
Outcomes	Eligible outcomes *Secondary outcomes* Academic achievement, measured with: ∘ Child Maths Assessment (Starkey, 2012) ∘ Test of Early Mathematics Ability – 3rd Edition (TEMA‐3; Ginsburg, 2003) Cognitive development, measured by: Working memory, phonological awareness, acuity of approximate number system (not reported) Emotional well‐being & social competence, measured with: ∘ Attention Networks Test (Child‐ANT; Rueda, 2004) Ineligible outcomes: none reported **Timing of outcome measurement**: T1 post test (short‐term)	
Notes	Study start date: 2012 Study end date: 2014 **Funding source**: Institute of Education Sciences (NCSER), US Department of Education to the University of Texas at Austin Conflict(s) of interest: none reported **Comment(s)**: none	

***Risk of bias***		
**Bias**	**Authors' judgement**	**Support for judgement**
Random sequence generation (selection bias)	Unclear risk	**Comment** The randomisation process is not described in detail.
Timing and recruitment of clusters	Low risk	**Comment** Settings were randomised after children were recruited.
Allocation concealment (selection bias)	Unclear risk	**Comment** Not reported in enough detail
Blinding of participants and personnel (performance bias) All outcomes	Unclear risk	**Comment** Not reported in enough detail
Blinding of outcome assessment (detection bias) All outcomes	Unclear risk	**Comment** No detail is provided on the blinding of assessors however they were trained to conduct assessments.
Incomplete outcome data (attrition bias) All outcomes	Unclear risk	**Quote** Attrition is accounted for ‘The reasons for not being able to assess children at posttest were as follows in order of occurrence: withdrawal from school; relocation out of region or state; withdrawal of consent due to change in guardianship’ (p. 582). It is unclear how whether missing data affected the clusters.
Selective reporting (reporting bias)	Unclear risk	**Comment** Measures of other cognitive abilities examined in this study (working memory, phonological awareness, acuity of approximate number system) are not described or reported in this paper.
Other bias	High risk	**Comment** The TAU condition differed across the two states, with teachers in Texas delivering twice the amount of maths teaching compared to the classrooms in California.

Barnett 2008

**Table:** 

***Study characteristics***		
Methods	**Design**: cluster‐randomised controlled trial	
Participants	**Location/setting**: New Jersey, USA; centre‐based school setting in urban district **Sample size**: 210 at baseline **Mean age**: not reported; 54% 4‐year olds, 46% 3‐year olds **Sex**: intervention = 53.8% boys; control = 52.4% boys **Race/ethnicity**: intervention = 92.5% Latino/Hispanic, 3.8% African‐American, 3.5% Asian; control = 92.6% Latino/Hispanic, 1.2% African‐American, 3.7% Asian, 2.5% multi‐racial	
Interventions	**Intervention (*n* = 88 at baseline)**: Tools of the Mind focuses on the development of self‐regulation while teaching literacy and maths skills. Developed by Bodrova (1996), it is based on the theories and practical insights on cognitive development of Luria (1966) and Vygotsky (1978), including the promotion of self‐regulation through a comprehensive system of activities. Basic principles of the curriculum include: (1) children construct their own knowledge; (2) development cannot be separated from its social context; (3) learning can lead development; and (4) language plays a central role in mental development Bodrova (2007). All classrooms met the statutory requirement of at least 6 h per day, 180 days per year. **Control (*n* = 122 at baseline)**: Balanced literacy curriculum with themes, the control curriculum was developed by the local school district teachers and administration during the 3 years before the study. The curriculum developed by the school district was based on the idea that literacy should be taught to young children in a balanced way (i.e., through controlled classrooms, with a greater emphasis on teacher‐imposed control and less on children regulating each other and themselves. All classrooms met the statutory requirement of at least 6 h per day, 180 days per year.	
Outcomes	Eligible outcomes *Secondary outcomes* Academic achievement, measured with: ∘ Early Childhood Environment Rating Scale (ECERS‐R; Harms, 2003) Language‐Reasoning ∘ Expressive One‐Word Picture Vocabulary Test (EWOPVT; Brownell, 2000) ∘ Get Ready to Read (Whitehurst, 2001) ∘ IDEA Oral Language Proficiency Test (OLPT; Ballard, 1980) ∘ Peabody Picture Vocabulary Test – 3rd edition (PPVT‐III; Dunn, 1997) ∘ Support for Early Literacy Assessment (SELA; Smith, 2001) ∘ Woodcock‐Johnson Psycho‐Educational Battery – Revised (WJ‐R) Applied Problems (McGrew, 2001) ∘ WJ‐R Letter‐Word Identification subtest (McGrew, 2001) Cognitive development, measured with: ∘ Wechsler Preschool Primary Scale of Intelligence (WPPSI; Wechsler, 2003) (Animal Pegs subtest) (cognitive development) Emotional well‐being & social competence, measured with: ∘ Problem Behaviours Scale of the Social Skill Rating System (Gresham, 1990) **Ineligible outcomes** Frequency of use of scaffolding techniques, measured with: ∘ Preschool Classroom Implementation (PCI) rating scale (outcome not detailed in protocol) Classroom quality, measured with: ∘ ECER‐S Activities, Interactions, Parents & Staff Personal Care Subscale, Programme, Space & Furnishings, Total Score (outcome not detailed in protocol) **Timing of outcome measurement**: baseline; post test (not reported)	
Notes	Study start date: 2002 Study end date: 2003 **Funding source**: part of the state‐financed, full‐day preschool education programme Conflicts of interest: none stated **Comment(s)**: data were not reported fully and we were unable to make contact with the author (Table 1)	

***Risk of bias***		
**Bias**	**Authors' judgement**	**Support for judgement**
Random sequence generation (selection bias)	Unclear risk	**Comment** The block randomisation is not described in detail.
Timing and recruitment of clusters	Unclear risk	**Comment** Unclear sequence of randomisation
Allocation concealment (selection bias)	Unclear risk	**Comment** Teachers were randomly assigned to treatment or control classrooms but allocation procedure is not adequately described.
Blinding of participants and personnel (performance bias) All outcomes	Unclear risk	**Quote** ‘Efforts were made to control treatment diffusion between the two groups of teachers by placing all the treatment classes on one floor of the building and all the control classes on another floor … All classrooms looked very similar physically before the Tools curriculum intervention. For example, the school district ordered all classrooms exactly the same amount and type of furniture, toys, art supplies and books’. (p. 303)
Blinding of outcome assessment (detection bias) All outcomes	Low risk	**Quote** ‘Our team video‐taped classrooms and another team that was completely blind to treatment or control status coded the video tapes’ (p. 306)
Incomplete outcome data (attrition bias) All outcomes	Unclear risk	**Quote** ‘It was not possible to conduct extensive analyses of attrition, because most attrition in this study was due to lack of active consent from parents before any data collection’. (p. 303)
Selective reporting (reporting bias)	Unclear risk	**Comment** All outcome measures are reported in full.
Other bias	Unclear risk	**Comment** No other sources of potential bias were identified.

Bierman 2008

**Table:** 

***Study characteristics***		
Methods	**Design**: cluster‐randomised controlled trial	
Participants	**Location/setting**: USA; Head Start centres **Sample size**: 356 (44 classrooms) **Age**: 4 years **Sex**: 46% boys **Race/ethnicity**: Head Start‐REDI (Research‐based, Developmentally‐informed) = 39% minority ethnic; Head Start = 45% minority ethnic; total sample: 17% Hispanic; 25% African‐American	
Interventions	**Intervention (*n* = 296)**: Head Start‐REDI was designed as an enrichment intervention that could be integrated into the existing framework of Head Start programmes using the High/Scope or Creative Curriculum. The intervention involved brief lessons, ‘hands on’ extension activities and specific teaching strategies linked empirically with the promotion of: (1) social‐emotional competencies (using the Preschool PATHS Curriculum (Domitrovich, 1999); and (2) language development and emergent literacy skills. Take‐home materials were provided to parents to enhance skill development at home. **Control (*n* = 60)**: treatment as usual; Head Start curriculum	
Outcomes	Eligible outcomes *Secondary outcomes* Academic achievement, measured with: ∘ Expressive One‐Word Picture Vocabulary Test (EOWPVT; Brownell, 2000) ∘ Test of Language Development (TOLD; Newcomer, 1997) – Grammatical Understanding subtest; Sentence Imitation subtest ∘ Test of Preschool Early Literacy (TOPEL; Lonigan, 2007) – Blending subtest; Elision subtest; Print Knowledge subtest Cognitive development, measured with: ∘ Adapted Leiter‐R Assessor Report (Roid, 2003a) Emotional well‐being & social competence, measured with: ∘ Assessment of Children's Emotion Skills (ACES; Schultz, 2004) ∘ Challenging Situations Task (CST; Denham, 1994) – Aggressive Responses; Competent Responses; Inept Responses ∘ Emotion Recognition Questionnaire (Ribordy, 1988) ∘ Preschool Self‐Regulation Assessment (Task orientation) (Smith‐Donald, 2007) ∘ Preschool Social Behaviour Scale – Teacher Form (PSBS; Crick, 1997) ∘ Social Competence Scale (CPPRG, 1995) – Teacher Rating; Parent Rating; Observer Rating ∘ Teacher Observation of Child Adaptation – Revised (TOCA‐R; Werthamer‐Larsson, 1991) Economic costs, measured by: ∘ Total direct costs (personnel: supervision/administration, contract personnel; non‐personnel: materials, travel costs, facilities; training: supervision/administration, teacher payments, training materials, meeting costs, travel for training) ∘ Average cost per participant **Ineligible outcomes** Learning engagement at school and at home, measured with: ∘ ADHD Rating Scale (DuPaul, 1991) (not detailed in protocol) ∘ Teacher rating of learning engagement at school (authors' own) (not detailed in protocol) **Timing of outcome measurement**: baseline; T2 post test (short‐term); T3 kindergarten (medium term)	
Notes	Study start date: 2003 Study end date: 2005 **Funding source**: Interagency School Readiness Consortium by National Institute of Child Health and Human Development grants HD046064 and HD43763 Conflict(s) of interest: none reported **Comment(s)**: none	

***Risk of bias***		
**Bias**	**Authors' judgement**	**Support for judgement**
Random sequence generation (selection bias)	Unclear risk	**Quote** Insufficient information, ‘Using a stratified randomization process, classes were divided into groups based on demographic characteristics of the population served, location, length of school day. Within stratified groups, centres were randomly assigned to intervention or control conditions’ (p. 6)
Timing and recruitment of clusters	High risk	**Comment** Participants were recruited after clusters had been randomised.
Allocation concealment (selection bias)	Unclear risk	**Comment** Not reported in enough detail
Blinding of participants and personnel (performance bias) All outcomes	Unclear risk	**Comment** No blinding, but outcome not likely to be influenced by lack of blinding
Blinding of outcome assessment (detection bias) All outcomes	Low risk	**Quote** ‘Although teachers knew which children were receiving intervention, child assessors, observers, and parents were naive concerning the intervention/control status of the children’. (p. 7)
Incomplete outcome data (attrition bias) All outcomes	Unclear risk	**Comment** No information given on attrition or missing data from clusters or individuals within clusters.
Selective reporting (reporting bias)	Low risk	**Comment** All measures reported
Other bias	Unclear risk	**Comment** Financial incentives were offered to both parents and teachers to complete the assessments.

Blair 2014

**Table:** 

***Study characteristics***		
Methods	**Design**: cluster‐randomised controlled trial	
Participants	Location/setting: USA **Sample size**: 759 (79 classrooms in 29 schools) **Mean age**: not reported **Sex**: intervention group = 48% boys; control group = 49% boys **Race/ethnicity**: 73% White, 2% African American, 7% Hispanic, 4% Asian, 1% Native American, 13% Multi‐race, and 1% Other	
Interventions	**Intervention (*n* = 443; 42 classrooms)**: Tools of the Mind; teaching practices and activities of Tools of the Mind are designed to promote academic learning and ability by broadly focusing on multiple aspects of self‐regulation including executive functions, social and emotion regulation skills, the control of attention, and the regulation of stress response physiology. Teachers and teaching assistants in the Tools of the Mind classrooms were trained in a 2‐year professional development cycle. In Year 1 teachers had 4 workshops spread across the year with a total of 5 days of training. Year 2 had 3 training workshops spread across the year with 3 days of training. Each school had a Tools coach that worked with the Tools trainer to provide in‐classroom coaching once every other week during Year 1 and then once a month in Year 2. **Control (*n* = 316; 37 classrooms)**: treatment as usual; classrooms in the control schools used a combination of commercial literacy and mathematics curricula and followed state standards for the development of science and social studies curricula, a typical scenario for kindergarten classrooms. Tools and control classroom curricula meet Massachusetts State Standards and are aligned with the Common Core Standards. No classrooms in the control condition contained activities resembling those in the Tools of the Mind classrooms.	
Outcomes	Eligible outcomes *Secondary outcomes* Academic achievement, measured with: ∘ Expressive One‐Word Picture Vocabulary Test (EOWPVT; Brownell, 2000) ∘ Woodcock‐Johnson III Tests of Achievement (WJ‐III; (Woodcock, 2001) Applied Problems subtest, Letter‐Word Identification subtest, Reading Vocabulary subtest Cognitive development, measured with: ∘ Dimensional Change Card Sort task (NIH Toolbox version) (Zelazo, 2013) ∘ Flanker with Reverse Flanker task (Diamond, 2007) ∘ Forward/Backward Digit Span task (Rudel, 1974) ∘ Hearts and Flowers task (Davidson, 2006) ∘ Raven Coloured Progressive Matrices test (Raven, 1956) Emotional well‐being & social competence, measured with: ∘ Dot‐Probe task (Kujawa, 2011) ∘ Emotion Regulation Checklist (ERC; Shields, 1997) ∘ Social Skills Rating System (SSRS; Gresham, 1990) Social Skills ∘ Strengths and Difficulties Questionnaire (SDQ; Goodman, 2001) ∘ Student–teacher Relationship Scale‐Short Form (STRS; Pianta, 2001) ∘ Teacher Social Competence Rating Scale (TSCRS; Kam, 1998) **Ineligible outcomes** Neuroendocrine and neurocognitive function, measured with: ∘ Saliva samples assayed for neuroendocrine function, cortisol and alpha amylase (not detailed in protocol) Measures of speed processing, measured with: ∘ Reaction time task (Fitzpatrick, 2014) (not detailed in protocol) ∘ Rapid automatised naming task (Denkla, 1976) (not detailed in protocol) **Timing of outcome measurement**: baseline (October), T2 short‐term (March), T3 short‐term (October)	
Notes	Study start date: 2010 Study end date: 2014 **Funding source**: Institute of Education Sciences grant R305A 100058 Conflict(s) of interest: none stated **Comment(s)**: none	
***Risk of bias***		
**Bias**	**Authors' judgement**	**Support for judgement**
Random sequence generation (selection bias)	Low risk	**Comment** Schools were blocked according to district, free or subsidise school meals, school size, state test scores, and number of kindergarten classrooms, and randomly assigned using computer‐generated randomisation. Randomisation resulted in small and acceptable differences in the number of schools randomised to the treatment versus control groups.
Timing and recruitment of clusters	Low risk	**Comment** Settings were randomised after children were recruited.
Allocation concealment (selection bias)	High risk	**Quote** ‘We attempted to recruit six children per classroom in each of the 2 years of the study. Recruitment was conducted through flyers sent to children's homes and through parent night activities. Parents provided written consent for children to participate and children provided verbal assent. In instances in which more than 6 children in a classroom expressed interest in participation, we enrolled the first 6 children for whom consent forms were returned by parents and assigned all other children with returned consents to a waiting list’. (p. 3)
Blinding of participants and personnel (performance bias) All outcomes	Unclear risk	**Comment** It was not possible to blind participants or teachers.
Blinding of outcome assessment (detection bias) All outcomes	High risk	**Quote** ‘Efforts were made to blind outcome assessors but the nature of Tools tasks it may have been obvious which condition the group had been assigned’. (p. 3)
Incomplete outcome data (attrition bias) All outcomes	Unclear risk	**Comment** Although missing data was accounted for, there were missing data from some clusters. **Quote** ‘Approximately 5% of participants [N= 34] were lost to follow‐up from fall to spring and replaced with participants on waiting lists in classrooms in which more than 6 participants expressed interest in participating in the study or in some classrooms with newly recruited participants’. (p. 3).
Selective reporting (reporting bias)	Low risk	**Comment** All outcomes have been reported
Other bias	Low risk	**Comment** No other sources of potential bias were identified.

Blatt 1965

**Table:** 

***Study characteristics***		
Methods	**Design**: randomised controlled trial	
Participants	**Location/setting**: ‘the most socially & economically deprived areas in metropolitan Boston; centre‐based special school setting’ (p. 47) **Sample size**: 59; pilot sample = 14 **Mean age**: intervention group 1 = 3.2 (SD 0.57) years, pilot sample = 5.0 years; intervention group 2 = 3.2 years; control group = 3.2 years, pilot sample = 5.3 years **Sex**: intervention group 1 = 61.1% boys; intervention group 2 = 55% boys; control group = 42.9% boys **Race/ethnicity**: not reported	
Interventions	**Intervention 1 (*n* = 18)**: preschool & responsive environments; ‘Optimal preschool education’ plus Moore's (1960, 1961, 1963) ‘autotelic’ ‘Responsive Environment’ using a typewriter to learn to read, write & type through fun, curiosity, self‐discovery. Socio‐emotional: help children learn how to function socially in a group instruction situation, arise curiosity, promote attitudes of inquisitiveness & positive learning. Cognitive: specific training in psychological functions fundamental to later acquisition of academic skills. Pre‐reading/writing & maths also referenced (language development, auditory discrimination, auditory memory, visual discrimination, memory, quantitative thinking, creative thinking. Physical development: motor co‐ordination **Intervention 2 (*n* = 20)**: preschool programme only, classes were held 5 days per week, 2 x 3 h sessions per day (Year 1, 166 days; Year 2, 147 days) **Control (*n* = 21)**: wait‐list and treatment as usual (5 local neighbourhood preschool programmes, duration not reported)	
Outcomes	Eligible outcomes *Secondary outcomes* Academic achievement, measured with: ∘ Illinois Test of Psycholinguistic Abilities (ITPA; McCarthy, 1961) ∘ Lee Clark Reading Readiness Test (Lee, 1934) ∘ Peabody Picture Vocabulary Test (PPVT; Dunn, 1959) ∘ School achievement Cognitive development, measured with: ∘ Stanford‐Binet Intelligence Scale (Terman, 1960) Emotional well‐being & social competence, measured with: ∘ Vineland Social Maturity Scale (Doll, 1953) ∘ Test Taking Behaviour (author's own) ∘ Sociogram (author's own) **Ineligible outcomes** Musical ability, measured with: ∘ Gesell and Ilg Norms for Musical Ability (Gesell, 1946) (outcome not detailed in protocol) Family achievement, measured with: ∘ Average of school grades and school behaviour of siblings of study children (outcome not detailed in protocol) Overall rating of differentiation and form level, measured with: ∘ Rorschach Inkblot Test (Vernon, 1933) (outcome not detailed in protocol) Test of responsive environment, measured with: ∘ Typewriter test (outcome not detailed in protocol) Environmental factors, measured with: ∘ Warner Index of Status Characteristics (Warner, 1960) (outcome not detailed in protocol) **Timing of outcome measurement**: baseline (July 1962), T2 short‐term (May 1963), T3 medium term (1964), T4 long term (1965)	
Notes	Study start date: 1962 Study end date: 1965 **Funding source**: Co‐operative Research Programme of the Office of Education, US Department of Health, Education & Welfare Conflict(s) of interest: none reported **Comment(s)**: none	

***Risk of bias***		
**Bias**	**Authors' judgement**	**Support for judgement**
Random sequence generation (selection bias)	Unclear risk	**Comment** Not reported
Allocation concealment (selection bias)	Unclear risk	**Comment** Not reported
Blinding of participants and personnel (performance bias) All outcomes	Unclear risk	**Comment** Not reported
Blinding of outcome assessment (detection bias) All outcomes	Low risk	**Quote** ‘During all testing, psychological examiners who had no previous connection with the study and were, therefore, disinterested in its results were employed. Every effort was made to assure ourselves that the psychological examiners were unaware of whether any particular child was an experimental or a non‐experimental child. This “blind” was completely successful in the third and fourth testings, partially successful in the second testing, and not applicable in the first testing because, obviously, groups were selected after the testing. Furthermore great care was taken to have each testing take place in a well controlled situation for both experimental and non‐experimental subjects. To achieve this control, all of the study children were brought into a common setting for a testing period in May of each year following the first year of intervention’. (p. 67)
Incomplete outcome data (attrition bias) All outcomes	High risk	**Comment** Although they state that only one child lost to the programme at T4, they maintained contact and testing of children who had moved away and were no longer receiving intervention.
Selective reporting (reporting bias)	Unclear risk	**Comment** Not reported
Other bias	High risk	**Quote** ‘We will often refer, specifically, to what was planned before the formal phase of the investigation started but we will try to make it clear that the vagaries of field research often caused us to depart from our plans to such an extent that the departure was more significant than the original plan itself’. (p. 63)

Castro 2017

**Table:** 

***Study characteristics***		
Methods	**Design**: cluster‐randomised controlled trial	
Participants	**Location/setting**: California, Florida, and North Carolina, USA **Sample size**: 340 Spanish‐English dual language learners (DLLs) **Mean age**: 4 years **Sex**: treatment group = 50% boys; control group = 55% boys **Race/ethnicity**: 45% Central American, 55% Mexican	
Interventions	**Intervention (*n* = not reported)**: Nuestros Ninos School Readiness (NNSR) Professional Development Programme; includes an integrative approach to teacher professional development and intervention aimed at promoting language, literacy, socio‐emotional development and mathematics learning in Spanish‐English DLLs. The programme in participating schools was delivered in tandem with the Creative Curriculum or High Scope curriculum. The content of the professional development institutes is documented in a handbook given to all teachers in the treatment group (Castro, 2011). The handbook contains six modules that include core concepts, teaching strategies, and classroom resources for DLLs. **Control (*n* = not reported)**: treatment as usual; Creative Curriculum or High Scope	
Outcomes	Eligible outcomes *Secondary outcomes* Academic achievement, measured with: ∘ Receptive One‐Word Picture Vocabulary Tests (ROWPVT; Martin, 2010) ∘ Name Writing Task (Yaden, 2004) ∘ Test of Early Mathematics Ability – Third Edition (TEMA‐3; Ginsburg, 2003) ∘ NNSR Receptive Word Knowledge Probe (RWKP; Castro, 2017) ∘ NNSR Expressive Word Knowledge Probe (EWKP; Castro, 2017) ∘ Woodcock‐Johnson Psycho‐Educational Battery‐Revised (WJ‐III; Woodcock, 1991, 1995) Letter‐Word Identification subtest* ∘ WJ‐III (Woodcock 1991, 1995) Picture Vocabulary subtest* ∘ Phonological Awareness Task (PAT; Miccio, 2002) Emotional well‐being & social competence, measured with: ∘ Social Competence and Behaviour Evaluation Preschool Edition (SCBE; LaFreniere, 1996) **Ineligible outcomes** Quality of teacher‐student interactions, measured with: ∘ The Classroom Assessment Scoring System (CLASS; Pianta, 2006) (outcome not detailed in protocol) Quality of classroom environment and teachers' practices, measured with: ∘ The Early Language and Literacy Classroom Observation Dual Language Learners (ELLCO‐DLL; Castro, 2005) (outcome not detailed in protocol) Assessment of language interactions in linguistically diverse classrooms, measured with: ∘ The Language Interaction Snapshot (LISn; Sprachman, 2009) **Timing of outcome measurement**: beginning and end of pre‐k year	
Notes	Study start date: 2010 Study end date: 2012 **Funding source**: National Institute of Child Health and Development (NICHD) Conflict(s) of interest: none reported **Comment(s)**: no breakdown of *N* reported, no author response from email request for raw data	

***Risk of bias***		
**Bias**	**Authors' judgement**	**Support for judgement**
Random sequence generation (selection bias)	Unclear risk	**Quote** ‘Random assignment to treatment (teachers received the NNSR PD intervention in addition to their curriculum) or control (only their curriculum) conditions was conducted at the programme/site level to maximize independence and ensure adequate statistical power’. (p. 192)
Timing and recruitment of clusters	Unclear risk	**Comment** Sequence not described.
Allocation concealment (selection bias)	Unclear risk	**Comment** Not described in detail
Blinding of participants and personnel (performance bias) All outcomes	Unclear risk	**Comment** Not described in detail
Blinding of outcome assessment (detection bias) All outcomes	Low risk	**Quote** ‘Data collectors were blind to group assignments (i.e., control and/or intervention groups)’. (p. 192)
Incomplete outcome data (attrition bias) All outcomes	High risk	**Quote** ‘Attrition was a limitation of the study – a 19% classroom attrition rate over the 2 years of intervention. Although, these teachers were replaced and we provided PD for them to catch up, these staff changes may have affected the study results. The attrition rate may explain the low level of implementation fidelity of about half of the teachers, as noted above. On the other hand, our fidelity results may have been affected by the way we collected the data. More precise forms of collecting fidelity of implementation which have been currently proposed take into account different aspects of fidelity such as adherence … Our measure focused on adherence to the NNSR strategies but did not provide a measure of dosage, quality of delivery or participant responsiveness, information that could have been useful for further interpreting the results’. (p. 201) This also may have impacted on data missing at a cluster level.
Selective reporting (reporting bias)	Low risk	**Comment** All outcomes have been reported
Other bias	Unclear risk	**Comment** No other sources of potential bias were identified

Coffman 1967

**Table:** 

***Study characteristics***		
Methods	**Design**: cluster‐randomised controlled trial	
Participants	**Location/setting**: Missouri, USA; half‐day pre‐kindergarten classes **Sample size**: 229 at baseline **Mean age**: not reported, all 4 years old **Sex**: intervention = 49.5% boys; control = not reported **Race/ethnicity**: not reported	
Interventions	**Intervention (*n* = 90)**: the curriculum emphasised the developmental skills basic to intellectual growth, but also included the usual framework of the nursery school activities which foster social, physical, and emotional maturation. The children were assigned to one of four classes following assessment of individual strengths and weaknesses. Three of the classes were given a specific programme of approximately 20 min a day emphasising the area of particular weakness (motor (EM), auditory‐language (EA), or visual (EV)) or assigned to a cognitive class (EC). The majority of the children in the cognitive class had shown no major weakness in these areas, and major emphasis for these children was put on the development of cognitive skills, such as associating, classifying, ordering, and remembering information. **Intervention (*n* = 90)**: experimental motor group (EM) *n* = 21; experimental auditory‐language group (EA) *n* = 23; experimental visual group (EV) *n* = 21; experimental cognitive group (EC) *n* = 25 **Control (*n* = 139**): Group 1 (*n* = 73): private nursery experience Group 2 (*n* = 66): no school experience	
Outcomes	Eligible outcomes *Secondary outcomes* Academic achievement, measured with: ∘ Peabody Picture Vocabulary Test (PPVT; Dunn, 1965) ∘ Three Dimensional Auditory Discrimination Test (Proger, 1970) ∘ Illinois Test of Psycholinguistic Abilities (ITPA; McCarthy, 1961) Physical development, measured with: ∘ Beery‐Buktenica Developmental Form Sequence (physical development) (Beery, 1997) ∘ Gross Motor Observations (physical development) Ineligible measures: none **Timing of outcome measurement**: baseline and post test (6 months)	
Notes	Study start date: 1966 Study end date: 1967 **Funding source**: Office of Education, US Department of Health, Education, and Welfare Conflicts of interest: none reported **Comment(s)**: data were not reported fully and we were unable to make contact with the author (Table 1).	

***Risk of bias***		
**Bias**	**Authors' judgement**	**Support for judgement**
Random sequence generation (selection bias)	Unclear risk	**Quote** ‘To maintain anonymity the 277 applicants identified only by a code number, were divided into 2 reasonably “matched” groups from which to draw the experimental & control children. This was done to assure comparability of the groups for end‐of‐year comparisons. The children were “matched” on ITPA L.Q., PPVT, IQ, age, sex & the public school which the children would attend kindergarten the following year’. (p. 4)
Timing and recruitment of clusters	Unclear risk	**Comment** Sequence is unclear
Allocation concealment (selection bias)	Unclear risk	**Quote** ‘The experimental group was designated by chance. As withdrawals from the experimental group occurred, “matching” replacements were drawn from the control group & from the remaining 69 applicants to comprise the final experimental & control groups of 100 children each. The children were assigned to one of 4 classes following assessment of individual strengths & weaknesses – the motor, auditory‐language & visual classes were specifically organised to help children overcome a weakness in a specific skills area. The cognitive class was provided for children whose developmental skills were intact’. (p. 5)
Blinding of participants and personnel (performance bias) All outcomes	Unclear risk	**Comment** Not described in detail
Blinding of outcome assessment (detection bias) All outcomes	Unclear risk	**Comment** Not described in detail
Incomplete outcome data (attrition bias) All outcomes	Unclear risk	**Comment** No reference to missing data
Selective reporting (reporting bias)	Unclear risk	**Comment** No study protocol provided
Other bias	Unclear risk	**Comment** No other sources of potential bias were identified

Courtier 2021

**Table:** 

***Study characteristics***		
Methods	**Design**: cluster‐randomised controlled trial	
Participants	**Location/setting**: Lyon, France; public preschool Sample size: 176 **Mean age**: intervention 1 M = 5.95 years (SD 0.28); intervention 2 M = 5.99 (SD 0.27); control M = 5.98 (SD 0.29) **Sex**: Intervention 1 49.1% boys; Intervention 2 48.7% boys; Control 57.8% boys **Race/ethnicity**: not collected	
Interventions	Intervention 1 (*n* = 53): Montessori‐public Intervention 2 (*n* = 45): Montessori‐private **Control (*n* = 78**): TAU conventional French public curriculum	
Outcomes	Eligible outcomes *Secondary outcomes* Academic achievement, measured with: ∘ Evaluation du Language Oral (Khomsi, 2001) ∘ Evaluation des fonctions cognitive et Apprentissages (Phonologie & Lecture sub tests; Billard, 2012) ∘ Scalar task (Stiller, 2015) ∘ Woodcock Johnston‐III Applied Problems subtest (Woodcock, 2001) ∘ Counting task (adapted from Lipton, 2005) ∘ Quantitative knowledge (token task) based on maths standards for children in kindergarten in France (Ministère de l'Education, 2015) Emotional well‐being & social competence, measured with: ∘ Corsi Block Tapping Test (Corsi, 1972) ∘ Dictator Game (Benenson, 2007) ∘ Evaluation des fonctions cognitives et des Apprentissages (Planification subtest; Billard, 2012) ∘ Head Toes Knees Shoulders task (Ponitz, 2009) ∘ Resource allocation task (inspired from Huppert, 2019) ∘ Theory of Mind (Wellman, 2004) ∘ Social problem Solving Task‐Revised (Rubin, 1988) Ineligible outcomes: none **Timing of outcome measurement**: post test (short term – end of kindergarten year, June)	
Notes	Study start date: 2016 Study end date: 2019 **Funding source**: Institut Carnot de l'Éducation Auvergne Rhône Alpes (ICE—AuRA PR06) Conflicts of interest: none reported **Comment(s)**: none	

***Risk of bias***		
**Bias**	**Authors' judgement**	**Support for judgement**
Random sequence generation (selection bias)	Unclear risk	**Quote** ‘This assignment was fully randomized except for children with disabilities and staff children (those were excluded from the study)’. (p. 2072)
Timing and recruitment of clusters	Low risk	**Comment** Settings were randomised after children were recruited.
Allocation concealment (selection bias)	Unclear risk	**Comment** Not described in detail
Blinding of participants and personnel (performance bias) All outcomes	Unclear risk	**Comment** Not described in detail
Blinding of outcome assessment (detection bias) All outcomes	High risk	**Quote** ‘assessors of the children were not blind to which group the children were in’. (p. 2084)
Incomplete outcome data (attrition bias) All outcomes	Unclear risk	**Quote** ‘Out of the 210 children, who had been enrolled in their respective schools for 3 years, 14 did not participate in the study because their parents did not give their consent. From the original sample of 196 children, children were excluded if they were not fluent in French (*N* = 3), had a diagnosed disability (*N* = 4), changed pedagogy at some point (*N* = 12), and were related to the staff (*N* = 1)’ (p. 2072). Missing data is not reported so unable to assess with this would have affected the clusters.
Selective reporting (reporting bias)	Low risk	**Comment** All outcomes have been reported.
Other bias	Unclear risk	**Comment** No other sources of potential bias were identified

Deutsch 1971

**Table:** 

***Study characteristics***		
Methods	**Design**: randomised controlled trial	
Participants	Location/setting: USA Sample size: 404 **Mean age**: not reported; range 4–7 years **Sex**: not reported **Race/ethnicity**: 100% Black	
Interventions	**Intervention (*n* = 275)**: The Institute of Developmental Studies (IDS) curriculum was designed to develop competencies most useful in later learning. These included the cognitive areas of language, perception and concept formation, and the area of self‐concept. As the programme developed, aspects of the curriculum were supplemented. The curriculum focuses on making materials and tasks appropriate to each child's developmental level allowing pupils to progress at their own pace. Teacher training and supervision, as well as parental engagement (parent training) also played a part in the core aims of this programme. In addition, it incorporated elements of other curricula, including the Stern method, Sullivan Reading materials and Houghton‐Mifflin Math Manual. **Control (*n* = 129)**: There were 3 different control groups: control self‐selected (Css) (no treatment, were randomised to the control group; however, parents wanted them to participate in the programme); Kindergarten control (Kc) (no treatment, did not receive pre‐kindergarten) and; C1 (no treatment, did not receive pre‐k or kindergarten, but started school at grade 1).	
Outcomes	Eligible outcomes *Secondary outcomes* Academic achievement, measured with: ∘ Metropolitan Achievement Tests, Arithmetic sub tests – Computation & Problem Solving (Hildreth, 1965) ∘ Metropolitan Achievement Tests, Reading sub tests – Word Knowledge & Reading (Hildreth, 1965) ∘ Peabody Picture Vocabulary Test (PPVT; Dunn, 1959) ∘ Reading Prognosis Test (author's own) Cognitive development, measured with: ∘ Lorge‐Thorndike Intelligence Tests (Lorge, 1954) ∘ Stanford‐Binet Intelligence Scale (Terman, 1960) Ineligible outcomes: none **Timing of outcome measurement**: baseline (pre pre‐k), T2 short‐term (post pre‐k or beginning of pre‐k), T3 long‐term (post‐k or pre‐first grade), T4 extended long‐term (3rd grade)	
Notes	Study start date: 1963 Study end date: 1968 **Funding source**: Office of Economic Opportunity, Washington (DC); Office of Education, Washingon (DC) Conflict(s) of interest: none reported **Comment(s)**: none	

***Risk of bias***		
**Bias**	**Authors' judgement**	**Support for judgement**
Random sequence generation (selection bias)	Unclear risk	**Quote** Not reported fully, ‘these children, chosen randomly from the total *N* constituted a control group’. (p. 33)
Allocation concealment (selection bias)	Unclear risk	**Comment** Not reported in enough detail
Blinding of participants and personnel (performance bias) All outcomes	Unclear risk	**Comment** Not reported in enough detail
Blinding of outcome assessment (detection bias) All outcomes	Unclear risk	**Comment** These tests were given by Institute personnel who were trained and experienced in test administration. Children within experimental and control groups, and from the various schools were randomly assigned to testers, so each tester administered measures to subjects from all groups. It was not possible to vary systematically the race and sex of testers, because at the time the evaluation began, qualified black testers were too few in number in the New York area.
Incomplete outcome data (attrition bias) All outcomes	Unclear risk	**Comment** Not reported in enough detail
Selective reporting (reporting bias)	Low risk	**Comment** Outcomes well described
Other bias	Unclear risk	**Comment** No other sources of potential bias were identified.

Diamond 2019

**Table:** 

***Study characteristics***		
Methods	**Design**: cluster‐randomised controlled trial	
Participants	Location/setting: Canada Sample size: 351 **Mean age**: intervention group = 5.03 (SD = 0.5) years; control group = 5.10 (SD = 0.6) years **Sex**: intervention = 50% boys; control = 48% boys **Race/ethnicity**: not reported	
Interventions	**Intervention (*n* = 172)**: The Tools of the Mind curriculum ‘is grounded in the idea that social‐emotional development and improving EFs, especially inhibitory control, is as important as teaching academic skills and content. Developed by educational psychologists, Bodrova and Leong (Bodrova 2007), Tools is based on the work of Vygotsky (Vygotsky, 1967, 1978) and has been revised and improved over 23 years of iterative research and implementation’. (p. 5) **Control (*n* = 180)**: treatment as usual; full‐day kindergarten	
Outcomes	Eligible outcomes *Secondary outcomes* Academic achievement, measured with: ∘ Developmental Reading Assessment (DRA2; Beaver, 2006) ∘ Reading, Writing, Maths teaching and assessment tools (BCT, 2019) Emotional well‐being & social competence, measured with: ∘ Social Inclusion and other Prosocial Behaviour, Attention‐Regulation and Self‐Control teaching and assessment tools (BCT, 2019) Ineligible outcomes: none **Timing of outcome measurement**: baseline (first month of school), T1 (post intervention in May)	
Notes	Study start date: not reported Study end date: not reported **Funding source**: The random assignment of classes and training of control‐group and Tools teachers was funded by the British Columbia Ministry of Health and the British Columbia Mental Health Foundation. Funds from the Spencer Foundation, the Bezos Family, partial salary support from a Canada Research Chair award and from two National Institute on Drug Abuse research grants helped to support the lead author (AD) during the time this study was conducted and the manuscript was written. Conflict(s) of interest: none **Comment(s)**: data were not reported fully and we were unable to make contact with the author (Table 1).	

***Risk of bias***		
**Bias**	**Authors' judgement**	**Support for judgement**
Random sequence generation (selection bias)	Unclear risk	**Comment** Not described in detail
Timing and recruitment of clusters	Unclear risk	**Comment** Not described in detail
Allocation concealment (selection bias)	Unclear risk	**Comment** Not described in detail
Blinding of participants and personnel (performance bias) All outcomes	Unclear risk	**Comment** Not described in detail
Blinding of outcome assessment (detection bias) All outcomes	Unclear risk	**Quote** ‘Students' attitudes and behaviour were reported by teachers. Teachers responded to an online survey (using the Survey Monkey platform) with multiple‐choice questions and open‐ended opportunities to elaborate’. (p. 7)
Incomplete outcome data (attrition bias) All outcomes	Unclear risk	**Comment** Not described in detail
Selective reporting (reporting bias)	Low risk	**Comment** All outcomes reported
Other bias	Unclear risk	**Comment** No other sources of potential bias were identified.

Farran 2015

**Table:** 

***Study characteristics***		
Methods	**Design**: cluster‐randomised controlled trial	
Participants	**Location/setting**: Tennessee and North Carolina, USA **Sample size**: 877 (60 classrooms) **Mean age**: intervention group = 54.1 (SD = 3.6) months; control group = 54.6 (SD = 3.7) months **Sex**: intervention group = 53% boys; control group = 58% boys **Race/ethnicity**: intervention group = 39% White, 29% Black, 24% Hispanic, 6% Asian, 1% Multi‐racial, 1% Other minority; control group = 41% White, 23% Black, 25% Hispanic, 6% Asian, 4% Multi‐racial, 1% Other minority	
Interventions	**Intervention (*n* = 498)**: Tools of the Mind; a curriculum which involved teacher training and implements a play‐based curriculum in classrooms **Control (*n* = 379)**: treatment as usual; standard Pre‐K	
Outcomes	Eligible outcomes *Secondary outcomes* Academic achievement, measured with: ∘ Adaptive Language Inventory (ALI; Feagans, 1983, 1995) ∘ Woodcock‐Johnson Psycho‐Educational Tests of Achievement (WJ‐III) (Woodcock, 2001) Academic Knowledge, Letter‐Word Identification, Oral Comprehension, Picture Vocabulary, and Applied Problems subtest Emotional well‐being & social competence, measured with: ∘ Cooper‐Farran Behaviour Rating Scales – Interpersonal Skills and Work‐Related Skills sub scales (Cooper, 1991) ∘ Copy Design task (Osborn, 1984) ∘ Corsi Blocks (Corsi, 1972) ∘ Dimensional Change Card Sort (DCCS; Zelazo, 2006) ∘ Head Toes Knees Shoulders (HTKS; Ponitz, 2009) ∘ Peg Tapping (Diamond, 1996) ∘ Self‐Regulation Assessor Rating (SAR; Smith‐Donald, 2007) Ineligible outcomes: none **Timing of outcome measurement**: baseline (fall pre‐k), T1 short‐term (spring pre‐k), T2 medium‐term (spring k), T3 long‐term (spring 1st grade)	
Notes	Study start date: 2009 Study end date: 2014 **Funding source**: The 5‐year research study was funded by a grant from the US Department of Education Institute of Education Sciences (R305A090533) Conflict(s) of interest: none **Comment(s)**: none	

***Risk of bias***		
**Bias**	**Authors' judgement**	**Support for judgement**
Random sequence generation (selection bias)	Unclear risk	**Comment** Not enough information about the randomisation process given.
Timing and recruitment of clusters	High risk	**Comment** Settings were randomised before children were recruited.
Allocation concealment (selection bias)	Unclear risk	**Comment** Not reported in enough detail
Blinding of participants and personnel (performance bias) All outcomes	Unclear risk	**Comment** Not reported in enough detail
Blinding of outcome assessment (detection bias) All outcomes	Unclear risk	**Comment** Not reported in enough detail
Incomplete outcome data (attrition bias) All outcomes	Unclear risk	**Comment** Not reported in enough detail
Selective reporting (reporting bias)	Unclear risk	**Comment** Not reported in enough detail
Other bias	Unclear risk	**Comment** Not reported in enough detail

Hsueh 2014

**Table:** 

***Study characteristics***		
Methods	**Design**: cluster‐randomised controlled trial	
Participants	**Location/setting**: Midwest/Plains, West and South, USA; Head Start centres in 5 states in 3 regions **Sample size**: 933 (155 classrooms) **Mean age**: 3.47 (SD 0.31) years **Sex**: 49.35% boys **Race/ethnicity**: not reported	
Interventions	**Intervention 1 (*n* = 246; 41 classrooms)**: Incredible Years; designed to enhance children's social‐emotional development. Teachers are trained to create an organised classroom that supports children's ability to learn by watching others (social learning) and children's behaviour regulation in the context of positive teacher‐child relationships. In an Incredible Years classroom, the teacher uses praise, clear commands, and consistent limit‐setting to encourage appropriate and positive behaviours instead of singling out children who are misbehaving. For example, during an activity where children are asked to sit quietly, the teacher might say, ‘I really like the way Juan is sitting with his hands in his lap’. (p. ES‐4) **Intervention 2 (*n* = 226; 37 classrooms)**: Preschool PATHS; an instructional approach to enhancing children's social‐emotional development through lessons and activities focused on children's understanding of emotions (emotion knowledge) and social problem‐solving skills, as well as through teacher modelling and support. In a PATHS classroom, teachers talk about their feelings and encourage children to think about their and others' feelings to help children understand and learn about emotions in the context of social interactions. For example, in a group activity, the teacher might point out facial cues, like a smile, that show that children are feeling happy. **Intervention 3 (*n* = 241; 37 classrooms)**: Tools of the Mind—Play; requires teachers to restructure the room and school day, with large blocks of time devoted to supporting and structuring (scaffolding children's make‐believe play and role‐playing games. By scaffolding students' play, teachers aim to enhance the children's planning skills, understanding of social roles, memory and capacity for focused attention, and social‐emotional understanding. For the Head Start CARES study, Tools of the Mind developers compressed the original 2‐year curriculum into a 1‐year enhancement focused on play, the central element of Tools of the Mind. For example, in a Tools of the Mind—Play classroom, a child might draw a picture showing that she intends to play house and will be the mother. Through a series of exchanges with the child, the teacher would seek to expand the complexity of the child's role play by asking questions like, ‘What will you do as the mother? How could you make dinner for your child? How would you get the food to cook dinner?’ (p. ES‐4). In doing so, the teacher helps to build the child's self‐regulation skills, planning skills, and ability to assume various perspectives through the role‐playing activity. **Control Group (*n* = 220; 40 classrooms)**: Head Start CARES (Classroom‐based Approaches and Resources for Emotion and Social skill promotion)	
Outcomes	Eligible outcomes *Secondary outcomes* Academic achievement, measured with: ∘ Academic Rating Scale (ARS; National Center for Education Statistics, 2021) Emotional well‐being & social competence, measured with: ∘ Behaviour Problems Index (BPI; Zill, 1990) ∘ Social Skills Rating Scale (SSRS; Gresham, 1990) ∘ Cooper‐Farran Behavioural Rating Scale (CFBRS; Cooper, 1991) ∘ Student–teacher Relationship Scale (STRS; Pianta, 2001) Ineligible outcomes: none **Timing of outcome measurement**: baseline (fall), T1 short‐term (spring)	
Notes	Study start date: 2010 Study end date: 2011 **Funding source**: Office of Head Start and the Office of Planning, Research and Evaluation in the Administration for Children and Families, US Department of Health and Human Services, contract number HHSP23320072909YC Conflict(s) of interest: funded by Head Start **Comment(s)**: none	

***Risk of bias***		
**Bias**	**Authors' judgement**	**Support for judgement**
Random sequence generation (selection bias)	Unclear risk	**Comment** Not enough detail provided about the randomisation process
Timing and recruitment of clusters	Unclear risk	**Comment** Not enough detail provided
Allocation concealment (selection bias)	Unclear risk	**Comment** Not enough detail provided
Blinding of participants and personnel (performance bias) All outcomes	Unclear risk	**Comment** Not enough detail provided
Blinding of outcome assessment (detection bias) All outcomes	Unclear risk	**Comment** Not enough detail provided
Incomplete outcome data (attrition bias) All outcomes	Low risk	**Comment** Attrition reported and analysed
Selective reporting (reporting bias)	Low risk	**Comment** All outcomes reported
Other bias	Low risk	**Comment** None identified

Lillard 2017

**Table:** 

***Study characteristics***		
Methods	**Design**: quasi‐randomised controlled trial	
Participants	**Location/setting**: Connecticut, USA; public schools **Sample size**: 141 (intervention group = 11 classrooms; control group = 51 schools) **Mean age**: intervention group = 41.31 months; control group = 41.00 months **Sex**: intervention group = 55.7% boys; control = 53.5% boys **Race/ethnicity**: intervention = 48% Caucasian, 17% African American,16% Hispanic, 3% Asian, 16% Multi‐ethnic; control 37% Caucasian, 15% African American, 23% Hispanic, 4% Asian, 20% Multi‐ethnic	
Interventions	**Intervention (*n* = 70)**: Montessori; child‐directed, freely chosen activity and academic content **Control (*n* = 71)**: treatment as usual at both private and publicly funded schools	
Outcomes	Eligible outcomes *Secondary outcomes* Academic achievement, measured with: ∘ Woodcock‐Johnson Psycho‐Educational Battery‐Revised (WJ‐R; Woodcock, 2001) Applied Problems, Calculation, Letter‐Word, Picture Vocabulary sub tests ∘ Academic achievement measure (Z‐scores combined of Math, Letter‐Word and Picture Vocabulary, e.g., Lipsey, 2017) Emotional well‐being & social competence, measured with: ∘ Copy Design subtest from the Visuospatial Processing section (Nespy‐II; Korkman, 2007) ∘ Head‐Toes‐Knees‐Shoulders (HTKS; Ponitz, 2009) ∘ Puzzle Task (Smiley, 1994) ∘ Social Problem‐Solving Test‐Revised (Rubin, 1988) ∘ Theory of Mind scale (Wellman, 2014) **Ineligible outcomes** Children's enjoyment of academic (school and reading) and leisure (media and play) tasks, measured with: ∘ Questionnaire developed by the authors (outcome not detailed in protocol) Creativity, measured with: ∘ Alternative Uses (Guilford, 1973) (outcome not detailed in protocol) **Timing of outcome measurement**: baseline (fall), T1 short‐term (spring pre‐k), T2 medium‐term (spring k), T3 long‐term (spring 1st grade)	
Notes	Study start date: 2010 Study end date: 2016 **Funding source**: Brady Education Foundation Conflict(s) of interest: none reported **Comment(s)**: none	

***Risk of bias***		
**Bias**	**Authors' judgement**	**Support for judgement**
Random sequence generation (selection bias)	High risk	**Quote** The lottery was done by computer, ‘lottery selection was random except for neighbourhood, sibling, and staff preferences’ (p. 5)
Allocation concealment (selection bias)	Unclear risk	**Quote** ‘2 study children were admitted to a Montessori via the sibling preference; their siblings had presumably been admitted at random so the latent parent characteristics the lottery was intended to control for were still present. One control child had been admitted to Montessori but did not attend because the parents “did not like the neighborhood the school was in”’ (p. 5)
Blinding of participants and personnel (performance bias) All outcomes	Unclear risk	**Comment** Intervention effects *could* be due to being in a Montessori school, but could also be related to any one of a number of intra‐ and inter‐school factors that the method of randomisation couldn't balance out.
Blinding of outcome assessment (detection bias) All outcomes	Unclear risk	**Comment** Intervention effects *could* be due to being in a Montessori school, but could also be related to any one of a number of intra‐ and inter‐school factors that the method of randomisations couldn't balance out.
Incomplete outcome data (attrition bias) All outcomes	Low risk	**Comment** Over 4 years, 174 children were admitted to the study; 141 were retained in the final sample. Of these 141, 122 children were tested at all 4 time points, and 19 were tested at 3 time points. Of these 19, one joined the study at Time 2, 2 missed one test session, and 16 moved or crossed over between Time 3 and Time 4. 11 of these were in Montessori and 5 were control children. The control children who were lost had all moved; this lost subset of control children had performed significantly lower in academic achievement at earlier time points than the control children who did not move. The Montessori children who were lost at Time 4 did not significantly differ from those who remained in the study. Thus attrition patterns bias Time 4 results toward better outcomes for the control sample. For the variables reported here and the remaining children, 2.6% of data is missing due to experimenter error, child non‐compliance, or interruptions in testing.
Selective reporting (reporting bias)	Low risk	**Comment** Outcome measures reported match those in methods section
Other bias	Low risk	**Comment** None identified

Lipsey 2009

**Table:** 

***Study characteristics***		
Methods	**Design**: cluster‐randomised controlled trial	
Participants	**Location/setting**: Tennessee, USA; nursery school Sample size: 2990 **Mean age**: ITT group = 53.2 months; control group = 53.3 months (pooled SD 3.47) **Sex**: ITT group 49.5% boys; control group 48.9% boys **Race/ethnicity**: ITT group White 66.4%, Black 19.6%, Hispanic 14.0%; control group White 67.4% Black 19.5% Hispanic 13.2%	
Interventions	**Intervention (*n* = 1852)**: Tennessee Voluntary Pre‐k Programme; minimum instruction time of 5.5 h per day, 5 days per week in classes of no more than 20 students by a state‐licensed teacher endorsed for early childhood education. The programme must use an approved curriculum from a list provided by the Tennessee State Department of Education. **Control (*n* = 1138)**: wait list	
Outcomes	Eligible outcomes *Secondary outcomes* Academic achievement, measured with: ∘ Woodcock Johnson (WJ) Letter Word Identification; Spelling; Oral Comprehension; Picture Vocabulary; Passage Comprehension; Applied Problems; Quantative Concepts; Calculation (McGrew 2001) ∘ WJ Composite (mean of all scores except Passage Comprehension & Calculation & Composite) Ineligible outcomes: none **Timing of outcome measurement**: baseline; post test (kindergarten); follow‐up 1, 2 & 3 (grades 1st, 2nd & 3rd)	
Notes	Study start date: 2008 Study end date: 2012 **Funding source**: Institute of Education Sciences, US Department of Education and the US Department of Health and Human Services Conflicts of interest: none stated **Comment(s)**: data were not reported fully and we were unable to make contact with the author (Table 1)	

***Risk of bias***		
**Bias**	**Authors' judgement**	**Support for judgement**
Random sequence generation (selection bias)	Unclear risk	**Quote** ‘All their eligible applicants were placed on a list that was then sorted into random order by the research team’. (p. 4)
Timing and recruitment of clusters	High risk	**Comment** Settings were randomised before children were recruited.
Allocation concealment (selection bias)	High risk	**Quote** ‘Program staff were asked to fill their pre‐k seats in the order that children appeared on these randomised lists. The procedure was refined for the second cohort to ask program staff to attempt to contact a parent at least three times on different days and times to offer admission. Only if they were unable to contact a parent after these attempts or the parent declined the offer, were they to go to the next child on the list whose parents had not yet been contacted. Once the slots in a given program were filled, the remaining children became a waiting list’. (p. 157)
Blinding of participants and personnel (performance bias) All outcomes	Unclear risk	**Comment** Not enough detail provided
Blinding of outcome assessment (detection bias) All outcomes	Unclear risk	**Comment** Not enough detail provided
Incomplete outcome data (attrition bias) All outcomes	High risk	**Comment** Less than a quarter of parents gave consent for testing.
Selective reporting (reporting bias)	Low risk	**Comment** All outcomes are reported
Other bias	Unclear risk	**Comment** None identified

Lonigan 2015

**Table:** 

***Study characteristics***		
Methods	**Design**: cluster‐randomised controlled trial	
Participants	**Location/setting**: Florida (49 centres) and Texas (52 centres), USA; 101 preschool centres, including 63 Head Start centres, 32 private preschool centres and 6 charter/regular public school programmes **Sample size**: 760 randomised at baseline (110 classrooms) **Mean age**: 4.48 (SD 0.43) years; treatment group = 4.6 years; control group = 4.7 years **Sex**: 47% boys **Race/ethnicity**: not reported	
Interventions	**Intervention 1 (*n* = 300)**: Implicit Socioemotional Experimental Curriculum (Implicit SE); created by combining the primary elements of Literacy Express (LEC) and Pre‐K Mathematics. LEC is a comprehensive curriculum that uses 10 thematic units to provide an integrated environment for both teacher and child‐initiated learning across the full range of early learning domains (e.g., language and literacy, science and maths, motor skills). Pre‐K Mathematics is a supplemental curriculum consisting of 7 sequenced units of activities addressing number sense, arithmetic reasoning, spatial and geometric reasoning, patterns, measurement and logical reasoning. PATHS is a supplemental curriculum to help young children develop specific strategies that promote reflective responses and mature thinking skills, become more self‐motivated and enthusiastic about learning, obtain information necessary for social understanding and prosocial behaviour, increase their ability to generate creative alternative solutions to problems, and learn to anticipate and evaluate situations, behaviours, and consequences. In Implicit SE, teacher PD and written guidance on general classroom and behaviour management skills were included, but these skills were not the primary focus of any specific classroom instructional activity. **Intervention 2(*n* = 309)**: Explicit Socioemotional Experimental Curriculum (Explicit SE); in Explicit SE, the elements of the PATHS curriculum were included in addition to the general classroom and behaviour management skills of the Implicit SE curriculum. In this version, there were specific teacher‐directed, child‐focused, classroom activities designed to promote children's socio emotional development. Because only selected components of each of the three curricula were included in the combined versions, this study was not a test of any of the individual curricula. **Control (*n* = 142)**: treatment as usual; 10 different general, literacy or maths‐focused curricula were in use in centres assigned to the control condition. Although most curricula were in use by just a few enters, seven control enters in Texas reported using the Scholastic Early Childhood Programme. Teachers in the control group continued to use their existing curriculum throughout the study.	
Outcomes	Eligible outcomes *Secondary outcomes* Academic achievement, measured with: ∘ Child Math Assessment (CMA; Klein, 2004b) ∘ Diagnostic Evaluation of Language Variations (DELV; Seymour, 2003) ∘ Expressive One‐Word Picture Vocabulary Test (EWOPVT; Brownell, 2000) ∘ Test of Preschool Early Literacy (TOPEL; Lonigan 2007) – Phono subtest; Elision subtest; Print Knowledge subtest Emotional well‐being & social competence, measured with: ∘ Social Competence and Behaviour Evaluation – Social Competence, Anxiety, Anger & Aggression sub scales (SCBE; LaFreniere, 1996) ∘ Emotional understanding, adapted from Bullock (1985) ∘ Teacher Behaviour Rating Scale (TBRS; Landry, 2000) Ineligible outcomes: none **Timing of outcome measurement**: baseline (fall pre‐k), T1 short‐term (spring pre‐k)	
Notes	Study start date: not reported Study end date: not reported **Funding source**: Eunice Kennedy Shriver National Institute of Child Health and Human Development **Conflict(s) of interest**: Several of the authors are authors or coauthors of the preschool curricula used to develop the comprehensive curriculum used in this study. Authors Lonigan, Phillips, and Clancy are coauthors of Literacy Express Comprehensive Preschool Curriculum and receive occasional royalties; authors Starkey and Klein are coauthors of the Pre‐K Mathematics curriculum and receive occasional royalties; author Domitrovich is the coauthor of Promoting Alternative Thinking Strategies and receives royalties from Channing‐Bete Inc., through an agreement managed by the Pennsylvania State University Individual Conflict of Interest Committee. In addition, Lonigan is the lead author for the Test of Preschool Early Literacy and receives occasional royalties; authors Starkey and Klein are lead authors for the Child Math Assessment and receives occasional royalties; and author Jill de Villiers is a coauthor of the Diagnostic Evaluation of Language Variation–Screening Test and receives occasional royalties (author Peter de Villiers was a consultant for the development of this test, but receives no royalties). **Comment(s)**: none	

***Risk of bias***		
**Bias**	**Authors' judgement**	**Support for judgement**
Random sequence generation (selection bias)	Unclear risk	**Comment** No details reported
Timing and recruitment of clusters	High risk	**Comment** Settings were randomised before children were recruited.
Allocation concealment (selection bias)	Unclear risk	**Comment** No details reported
Blinding of participants and personnel (performance bias) All outcomes	Unclear risk	**Comment** It was not possible to blind the students and teachers to their curricular assignment.
Blinding of outcome assessment (detection bias) All outcomes	Low risk	**Comment** To the extent possible, assessment staff members and observation staff members were blind to a classroom's assigned condition and were unaware of the study's hypotheses. Before data collection commenced, all assessment and observation staff members received training and practice on the measures they used.
Incomplete outcome data (attrition bias) All outcomes	Low risk	**Quote** ‘Of the 855 children assessed in the fall, 760 (89%; 374 in Florida, 386 in Texas) children completed the end‐of‐year posttest assessments’. (p. 1778)
Selective reporting (reporting bias)	Low risk	**Comment** Reported results match those in measures
Other bias	Low risk	**Comment** None identified

PCER 2008, Bright Beginnings & Creative Curriculum

**Table:** 

***Study characteristics***		
Methods	**Design**: cluster‐randomised controlled trial	
Participants	**Location/setting**:Tennessee, USA; state pre‐kindergarten classrooms **Sample size**: 309 (36 classrooms) **Mean age**: intervention group 1 = 3.2 (SD 0.57) years, pilot sample = 5.0 years; intervention group 2 = 3.2 years; control group 3.2 years, pilot sample = 5.3 years **Sex**: intervention group 1 = 61.1% boys; intervention group 2 = 55% boys; control group = 42.9% boys **Race/ethnicity**: not reported	
Interventions	**Intervention 1 (*n* = 101)**: Creative Curriculum; theoretically underpinned by the work of Brazelton and Maslow, Erikson and Greenspace, Piaget and Vyotsky, it includes developmentally appropriate goals and objectives for children within four main categories of interest: social/emotional, physical, cognitive and language. The social/emotional stage helps promote independence, self‐confidence and self‐control. Within this stage, children learn how to make friends, how to have group interactions and how to follow rules. The physical stage is intended to increase children's large and small motor skills. The cognitive stage is associated with thinking skills. Children learn how to solve problems, ask questions and think critically. The language stage deals with communication. Children learn how to communicate with others, listen and participate in conversations, and recognise various forms of print. In this stage, children begin to recognise letters and words and begin writing for a purpose. **Intervention 2 (*n* = 103)**: Bright Beginnings; based in part on High/Scope and Creative Curriculum, with an additional emphasis on literacy skills. The curriculum consists of nine thematic units designed to enhance children's cognitive, social, emotional, and physical development. Each unit includes concept maps, literacy lessons, early childhood centre activities, and home activities. Special emphasis is placed on the development of early language and literacy skills. Parent involvement is a key component of the programme. Classes were held 5 days per week, 2 × 3 h sessions per day (Year 1 = 166 days; Year 2 = 147 days) **Control (*n* = 105)**: treatment as usual; in the control classrooms, teachers used teacher‐developed curricula with a focus on basic school readiness	
Outcomes	Eligible outcomes *Secondary outcomes* Academic achievement, measured with: ∘ Child Math Assessment – Abbreviated composite score (CMA‐A; Klein, 2002) ∘ Comprehensive Test of Phonological Processing (CTOPP; Wagner, 1999), Elision subtest ∘ Peabody Picture Vocabulary Test – 3rd edition (PPVT‐III; Dunn, 1997) ∘ Preschool Comprehensive Test of Phonological & Print Processing (Pre‐CTOPPP; Lonigan, 2002), Elision subtest ∘ Test of Early Reading Ability – 3rd edition (TERA‐3; Reid, 2001) ∘ Test of Language Development (TOLD; Newcomer, 1997), Grammatical Understanding subtest ∘ Woodcock‐Johnson Achievement Test – 3rd edition (WJ‐R‐III; McGrew, 2001) Applied Problems, Letter‐Word Identification & Spelling sub tests Cognitive development, measured with: ∘ Building Blocks, Shape Composition Task (Clements, 2003) Social competence & emotional well‐being, measured with: ∘ Learning Behaviours Scale (LBS; McDermott, 2000), Kindergarten ∘ Social Skills Rating System (SSRS; Gresham, 1990) Social Skills & Problem Behaviours **Ineligible outcomes** Global classroom quality (outcome not detailed in protocol), measured with: ∘ ECERS‐R Teacher‐child interaction, measured with: ∘ Arnett Caregiver Interaction Scale (CIS; Arnett, 1986) (outcome not detailed in protocol) Teacher instructional practices, measured with: ∘ TBRS Book Reading; Oral Language; Phonological Awareness; Print & Letter Knowledge; Written Expression; & Math Concept (Landry, 2002) (outcome not detailed in protocol) **Timing of outcome measurement**: baseline (fall pre‐k), T2 short‐term (spring pre‐k), T3 medium term (spring k)	
Notes	Study start date: 2002 Study end date: 2005 **Funding source**: Institute of Education Sciences, US Department of Education **Conflict(s) of interest**: members of the PCER research team developed measures included in the PCER Child Assessment Battery: Child Math Assessment; Building Blocks, Shape Composition Task; Pre‐CTOPPP, Elision subtest (this assessment was not commercially available at the time it was selected for inclusion or during data collection. A revised version of the assessment became commercially available after data collection, Dr Lonigan has a financial interest in this measure.). **Comment(s)**: none	

***Risk of bias***		
**Bias**	**Authors' judgement**	**Support for judgement**
Random sequence generation (selection bias)	Unclear risk	**Comment** Block random assignment was used. Blocking differed by team and included demographics (e.g., similar neighbourhoods or schools), type of preschool programme (e.g., Head Start or public preschool), feeder elementary school performance, and teacher qualifications (e.g., education level and certification).
Timing and recruitment of clusters	High risk	**Comment** Settings were randomised before children were recruited.
Allocation concealment (selection bias)	Unclear risk	**Comment** Not reported in enough detail
Blinding of participants and personnel (performance bias) All outcomes	Unclear risk	**Comment** Not reported in enough detail
Blinding of outcome assessment (detection bias) All outcomes	Low risk	**Comment** The blinding of assessors is not reported, however, they were subject to a rigorous training programme and therefore considered as low risk.
Incomplete outcome data (attrition bias) All outcomes	Low risk	**Quote** ‘Twenty‐one classrooms were randomly assigned to treatment or control condition, all of which remained in the study throughout the pre‐kindergarten year. For the child assessment, the baseline (fall 2003) response rate was 100 percent, the spring 2004 response rate was 95 percent, and the kindergarten follow‐up response rate was 98 percent’. (p. 45)
Selective reporting (reporting bias)	Low risk	**Comment** All outcomes reported
Other bias	Low risk	**Comment** None identified

PCER 2008, Creative Curriculm

**Table:** 

***Study characteristics***		
Methods	**Design**: cluster‐randomised controlled trial	
Participants	**Location/setting**: North Carolina and Georgia, USA; Head Start classrooms **Sample size**: 194 (18 classrooms) **Mean age**: intervention group = 4.5 years; control group = 4.5 years **Sex**: intervention group = 44.2% boys; control group = 47.4% boys **Race/ethnicity**: 2.9% White, 85% African American, and 7.5% Hispanic	
Interventions	**Intervention (*n* = 97)**: Creative Curriculum; a comprehensive curriculum for 3‐ to 5‐year‐old children. The curriculum addresses four areas of development (social/emotional, physical, cognitive and language development). **Control (*n* = 97)**: treatment as usual; teachers used teacher‐developed, nonspecific curricula	
Outcomes	Eligible outcomes *Secondary outcomes* Academic achievement, measured with: ∘ Child Math Assessment – Abbreviated composite score (CMA‐A; Klein, 2002) ∘ Comprehensive Test of Phonological Processing (CTOPP; Wagner, 1999), Elision subtest ∘ Peabody Picture Vocabulary Test – 3rd edition (PPVT‐III; Dunn, 1997) ∘ Preschool Comprehensive Test of Phonological & Print Processing (Pre‐CTOPPP; Lonigan, 2002), Elision subtest ∘ Test of Early Reading Ability – 3rd edition (TERA‐3; Reid, 2001) ∘ Test of Language Development (TOLD; Newcomer, 1997), Grammatical Understanding subtest ∘ Woodcock‐Johnson Achievement Test – 3rd edition (WJ‐R‐III; McGrew, 2001) Applied Problems, Letter‐Word Identification & Spelling sub tests Cognitive development, measured with: ∘ Building Blocks, Shape Composition Task (Clements, 2003) Emotional well‐being & social competence, measured with: ∘ Learning Behaviours Scale (LBS; McDermott, 2000), Kindergarten ∘ Social Skills Rating System (SSRS; Gresham, 1990) Social Skills & Problem Behaviours **Ineligible outcomes** Global classroom quality (outcome not detailed in protocol), measured with: ∘ ECERS‐R Teacher‐child interaction, measured with: ∘ Arnett Caregiver Interaction Scale (CIS; Arnett, 1986) (outcome not detailed in protocol) Teacher instructional practices, measured with:TBRS Book Reading; Oral Language; Phonological Awareness; Print & Letter Knowledge; Written Expression; & Math Concept (Landry, 2002) (outcome not detailed in protocol) **Timing of outcome measurement**: baseline (fall pre‐k), T2 short‐term (spring pre‐k), T3 medium term (spring k)	
Notes	Study start date: 2002 Study end date: 2005 **Funding source**: Institute of Education Sciences, US Department of Education **Conflict(s) of interest**: members of the PCER research team developed measures included in the PCER Child Assessment Battery: Child Math Assessment; Building Blocks, Shape Composition Task; Pre‐CTOPPP, Elision subtest (this assessment was not commercially available at the time it was selected for inclusion or during data collection. A revised version of the assessment became commercially available after data collection, Dr Lonigan has a financial interest in this measure.). **Comment(s)**: none	

***Risk of bias***		
**Bias**	**Authors' judgement**	**Support for judgement**
Random sequence generation (selection bias)	Low risk	**Comment** Random number generator was used
Timing and recruitment of clusters	High risk	**Quote** ‘Randomization of teachers within centres was done during the pilot year (2002–03) of the study, and the same assignments were maintained for the second year (2003–04) of curriculum implementation. At the end of the pilot year, the North Carolina site retained eight (four treatment and four control) of the 10 classrooms. Two classrooms were dropped because they were funded by the state's More at Four program, had degreed teachers, and had problems with high rates of teacher attrition. The Georgia site retained 10 out of 10 classrooms’. (p. 58)
Allocation concealment (selection bias)	Low risk	**Comment** Randomisation and allocation was concealed
Blinding of participants and personnel (performance bias) All outcomes	High risk	**Comment** Treatment and control classrooms were housed in the same centres. Teachers within the Head Start centres worked closely together. There may have been a few instances where a treatment group teacher inadvertently shared aspects from the treatment curriculum content or training with a control teacher. The research team conducted focus groups of both groups of teachers and there were not many comments about sharing. The site‐specific implementation fidelity data suggests that the control group teachers were doing some of the activities on the Creative Curriculum fidelity checklist. However, many of the items focused on generally accepted early childhood practice and were not curriculum‐specific. It is not possible to attribute the control group scores to a contamination effect.
Blinding of outcome assessment (detection bias) All outcomes	Low risk	**Comment** The blinding of assessors is not reported, however, they were subject to a rigorous training programme and therefore considered as low risk.
Incomplete outcome data (attrition bias) All outcomes	High risk	**Comment** Eighteen classrooms were randomly assigned to treatment and control conditions. All 18 classrooms remained in the study throughout the pre‐kindergarten year. For the child assessment, the baseline (fall 2003) response rate was 98 percent, the spring 2004 response rate was 90 percent, and the kindergarten follow‐up response rate was 85 percent.
Selective reporting (reporting bias)	Low risk	**Comment** Outcomes fully reported
Other bias	Low risk	**Comment** None identified

PCER 2008, Doors to Discovery & Let's Begin

**Table:** 

***Study characteristics***		
Methods	**Design**: cluster‐randomised controlled trial	
Participants	**Location/setting**: Texas, USA; Head Start and public pre‐kindergarten programmes **Sample size**: 297 (44 classrooms) **Mean age**: treatment group 1 = 4.6 years; treatment group 2 = 4.7 years; control group = 4.7 years **Sex**: treatment group 1 = 55.6% boys; treatment group 2 = 54.0% boys; control group = 54.3% boys **Race/ethnicity**: 30.1% White, 13.3% African American, and 43.4% Hispanic	
Interventions	**Intervention 1 (*n* = 100)**: Doors to Discovery; based on oral language, phonological awareness, concepts of print, alphabet knowledge and writing, and comprehension **Intervention 2 (*n* = 101)**: Let's Begin with the Letter People curriculum; focuses on specific literacy and language skills, including oral language, phonological and phonemic awareness, and letter knowledge in multiple contexts **Control (*n* = 96)**: teacher‐developed, nonspecific curricula	
Outcomes	Eligible outcomes *Secondary outcomes* Academic achievement, measured with: ∘ Child Math Assessment – Abbreviated composite score (CMA‐A; Klein, 2002) ∘ Comprehensive Test of Phonological Processing (CTOPP; Wagner, 1999), Elision subtest ∘ Peabody Picture Vocabulary Test – 3rd edition (PPVT‐III; Dunn, 1997) ∘ Preschool Comprehensive Test of Phonological & Print Processing (Pre‐CTOPPP; Lonigan, 2002), Elision subtest ∘ Test of Early Reading Ability – 3rd edition (TERA‐3; Reid, 2001) ∘ Test of Language Development (TOLD; Newcomer, 1997), Grammatical Understanding subtest ∘ Woodcock‐Johnson Achievement Test – 3rd edition (WJ‐R‐III; McGrew, 2001) Applied Problems, Letter‐Word Identification & Spelling sub tests Cognitive development, measured with: ∘ Building Blocks, Shape Composition Task (Clements, 2003) Emotional well‐being & social competence, measured with: ∘ Learning Behaviours Scale (LBS; McDermott, 2000), Kindergarten ∘ Social Skills Rating System (SSRS; Gresham, 1990) Social Skills & Problem Behaviours **Ineligible outcomes** Global classroom quality (outcome not detailed in protocol), measured with: ∘ ECERS‐R Teacher‐child interaction, measured with: ∘ Arnett Caregiver Interaction Scale (CIS; Arnett, 1986) (outcome not detailed in protocol) Teacher instructional practices, measured with: ∘ TBRS Book Reading; Oral Language; Phonological Awareness; Print & Letter Knowledge; Written Expression; & Math Concept (Landry, 2002) (outcome not detailed in protocol) **Timing of outcome measurement**: baseline (fall pre‐k), T2 short‐term (spring pre‐k), T3 medium term (spring k)	
Notes	Study start date: 2002 Study end date: 2005 **Funding source**: Institute of Education Sciences, US Department of Education **Conflict(s) of interest**: members of the PCER research team developed measures included in the PCER Child Assessment Battery: Child Math Assessment; Building Blocks, Shape Composition Task; Pre‐CTOPPP, Elision subtest (this assessment was not commercially available at the time it was selected for inclusion or during data collection. A revised version of the assessment became commercially available after data collection, Dr Lonigan has a financial interest in this measure.). **Comment(s)**: none	

***Risk of bias***		
**Bias**	**Authors' judgement**	**Support for judgement**
Random sequence generation (selection bias)	Low risk	**Comment** Randomised using names in a hat
Timing and recruitment of clusters	High risk	**Comment** Settings were randomised before children were recruited.
Allocation concealment (selection bias)	Low risk	**Comment** Allocation was adequately concealed
Blinding of participants and personnel (performance bias) All outcomes	Unclear risk	**Comment** Not reported in enough detail
Blinding of outcome assessment (detection bias) All outcomes	Low risk	**Comment** The blinding of assessors is not reported, however, they were subject to a rigorous training programme and therefore considered as low risk.
Incomplete outcome data (attrition bias) All outcomes	Low risk	**Comment** Forty‐four classrooms were randomly assigned to treatment and control conditions. All 44 classrooms remained in the study throughout the pre‐kindergarten year. For the child assessment, the baseline (fall, 2003) response rate was 99 percent, the spring 2004 response rate was 94 percent, and the kindergarten follow‐up response rate was 94 percent.
Selective reporting (reporting bias)	Low risk	**Comment** All outcomes reported
Other bias	Unclear risk	**Comment** Not reported in enough detail

PCER 2008, Language‐Focused Curriculum

**Table:** 

***Study characteristics***		
Methods	**Design**: cluster‐randomised controlled trial	
Participants	**Location/setting**: Virginia, USA; Head Start and public pre‐kindergarten classrooms **Sample size**: 195 (14 classrooms) **Mean age**: intervention group = 4.6 years; control group = 4.6 years **Sex**: treatment group = 52.8% boys; control group = 52.7% boys **Race/ethnicity**: 70.8% White, 20.8% African American, and 4.5% Hispanic	
Interventions	**Intervention (*n* = 97)**: Language‐Focused Curriculum; designed for use with 3‐ to 5‐year‐old children with language limitations. The curriculum components include: thematic organisation of content by day, week and month; use of daily dramatic play to teach and use new linguistic concepts; use of both teacher‐led and child‐led activities to organise daily experiences; explicit attention to oral language goals across the day; and teacher use of the eight key ‘language stimulation techniques’ (p. 109) when interacting with children in the classroom. **Control (*n* = 98)**: treatment as usual; teachers reported using High/Scope curriculum materials, but the extent of High/Scope curriculum implementation in the control classrooms was not formally assessed	
Outcomes	Eligible outcomes *Secondary outcomes* Academic achievement, measured with: ∘ Child Math Assessment – Abbreviated composite score (CMA‐A; Klein, 2002) ∘ Comprehensive Test of Phonological Processing (CTOPP; Wagner, 1999), Elision subtest ∘ Peabody Picture Vocabulary Test – 3rd edition (PPVT‐III; Dunn, 1997) ∘ Preschool Comprehensive Test of Phonological & Print Processing (Pre‐CTOPPP; Lonigan, 2002), Elision subtest ∘ Test of Early Reading Ability – 3rd edition (TERA‐3; Reid, 2001) ∘ Test of Language Development (TOLD; Newcomer, 1997), Grammatical Understanding subtest ∘ Woodcock‐Johnson Achievement Test – 3rd edition (WJ‐R‐III; McGrew, 2001) Applied Problems, Letter‐Word Identification & Spelling sub tests Cognitive development, measured with: ∘ Building Blocks, Shape Composition Task (Clements, 2003) Emotional well‐being & social competence, measured with: ∘ Learning Behaviours Scale (LBS; McDermott, 2000), Kindergarten ∘ Social Skills Rating System (SSRS; Gresham, 1990) Social Skills & Problem Behaviours **Ineligible outcomes** Global classroom quality (outcome not detailed in protocol), measured with: ∘ ECERS‐R Teacher‐child interaction, measured with: ∘ Arnett Caregiver Interaction Scale (CIS; Arnett, 1986) (outcome not detailed in protocol) Teacher instructional practices, measured with: ∘ TBRS Book Reading; Oral Language; Phonological Awareness; Print & Letter Knowledge; Written Expression; & Math Concept (Landry, 2002) (outcome not detailed in protocol) **Timing of outcome measurement**: baseline (fall pre‐k), T2 short‐term (spring pre‐k), T3 medium term (spring k)	
Notes	Study start date: 2002 Study end date: 2005 **Funding source**: Institute of Education Sciences, US Department of Education **Conflict(s) of interest**: members of the PCER research team developed measures included in the PCER Child Assessment Battery: Child Math Assessment; Building Blocks, Shape Composition Task; Pre‐CTOPPP, Elision subtest (this assessment was not commercially available at the time it was selected for inclusion or during data collection. A revised version of the assessment became commercially available after data collection, Dr Lonigan has a financial interest in this measure.). **Comment(s)**: none	

***Risk of bias***		
**Bias**	**Authors' judgement**	**Support for judgement**
Random sequence generation (selection bias)	Low risk	**Comment** Adequately randomised
Timing and recruitment of clusters	High risk	**Comment** Clusters were randomised before children were recruited.
Allocation concealment (selection bias)	High risk	**Comment** Within each block, the highest numbered preschool was assigned to treatment and the next to control, and this process was repeated until all preschools were assigned.
Blinding of participants and personnel (performance bias) All outcomes	Unclear risk	**Comment** Not reported in enough detail
Blinding of outcome assessment (detection bias) All outcomes	Low risk	**Comment** The blinding of assessors is not reported, however, they were subject to a rigorous training programme and therefore considered as low risk.
Incomplete outcome data (attrition bias) All outcomes	Unclear risk	**Comment** Fourteen classrooms were randomly assigned to treatment or control condition. All 14 classrooms remained in the study from the beginning of the pre‐kindergarten year through the spring of the pre‐kindergarten year. For the child assessment, the baseline (fall 2003) response rate was 93 percent, the spring 2004 response rate was 96 percent, and the kindergarten follow‐up response rate was 97 percent. One cluster dropped out which may have affected missing data.
Selective reporting (reporting bias)	Low risk	**Comment** Outcomes fully reported
Other bias	Unclear risk	**Comment** Not reported in enough detail

PCER 2008, Literacy Express + DLM + OCR Pre‐K

**Table:** 

***Study characteristics***		
Methods	**Design**: cluster‐randomised controlled trial	
Participants	**Location/setting**: Florida, USA; public pre‐kindergarten programmes **Sample size**: 297 (17 classrooms) **Mean age**: intervention group 1 = 4.6 years; intervention group 2 = 4.6 years; control group = 4.6 years **Sex**: intervention group 1 = 52.7% boys; intervention group 2 = 51.0% boys; control group = 59.3% boys **Race/ethnicity**: 29.6% White, 58.9% African American and 5.6% Hispanic	
Interventions	**Intervention 1 (*n* = 99)**: Literacy Express; a preschool, literacy‐focused curriculum, which is designed to promote children's emergent literacy skills. The curriculum is structured around thematic units. **Intervention 2 (*n* = 101)**: research‐based instruction from Open Court Reading Pre‐K integrated with the comprehensive instructional framework of DLM Early Childhood Express. Children received instruction that is intended to provide them with a strong foundation in oral language and print awareness as well as research‐based instruction in phonics and early decoding and comprehension skills. **Control (*n* = 97)**: treatment as usual; school district provided High/Scope training	
Outcomes	Eligible outcomes *Secondary outcomes* Academic achievement, measured with: ∘ Child Math Assessment – Abbreviated composite score (CMA‐A; Klein, 2002) ∘ Comprehensive Test of Phonological Processing (CTOPP; Wagner, 1999), Elision subtest ∘ Peabody Picture Vocabulary Test – 3rd edition (PPVT‐III; Dunn, 1997) ∘ Preschool Comprehensive Test of Phonological & Print Processing (Pre‐CTOPPP; Lonigan, 2002), Elision subtest ∘ Test of Early Reading Ability – 3rd edition (TERA‐3; Reid, 2001) ∘ Test of Language Development (TOLD; Newcomer, 1997), Grammatical Understanding subtest ∘ Woodcock‐Johnson Achievement Test – 3rd edition (WJ‐R‐III; McGrew, 2001) Applied Problems, Letter‐Word Identification & Spelling sub tests Cognitive development, measured with: ∘ Building Blocks, Shape Composition Task (Clements, 2003) Emotional well‐being & social competence, measured with: ∘ Learning Behaviours Scale (LBS; McDermott, 2000), Kindergarten ∘ Social Skills Rating System (SSRS; Gresham, 1990) Social Skills & Problem Behaviours **Ineligible outcomes** Global classroom quality (outcome not detailed in protocol), measured with: ∘ ECERS‐R Teacher‐child interaction, measured with: ∘ Arnett Caregiver Interaction Scale (CIS; Arnett, 1986) (outcome not detailed in protocol) Teacher instructional practices, measured with: ∘ TBRS Book Reading; Oral Language; Phonological Awareness; Print & Letter Knowledge; Written Expression; & Math Concept (Landry, 2002) (outcome not detailed in protocol) **Timing of outcome measurement**: baseline (fall pre‐k), T2 short‐term (spring pre‐k), T3 medium term (spring k)	
Notes	Study start date: 2002 Study end date: 2005 **Funding source**: Institute of Education Sciences, US Department of Education **Conflict(s) of interest**: Literacy Express is a curriculum developed by the research team. However, the developers did not conduct the impact analyses reported. Members of the PCER research team developed measures included in the PCER Child Assessment Battery: Child Math Assessment; Building Blocks, Shape Composition Task; Pre‐CTOPPP, Elision subtest (this assessment was not commercially available at the time it was selected for inclusion or during data collection. A revised version of the assessment became commercially available after data collection, Dr Lonigan has a financial interest in this measure.). **Comment(s)**: none	

***Risk of bias***		
**Bias**	**Authors' judgement**	**Support for judgement**
Random sequence generation (selection bias)	Low risk	**Comment** Schools were randomly assigned (using the random function in Excel).
Timing and recruitment of clusters	High risk	**Comment** Settings were randomised before children were recruited.
Allocation concealment (selection bias)	High risk	**Comment** Schools were grouped into triplets. The three preschools within each triplet were randomly assigned with one assigned to the first intervention curriculum, one going to the second intervention curriculum, and one going to the control.
Blinding of participants and personnel (performance bias) All outcomes	Unclear risk	**Comment** Not reported in enough detail
Blinding of outcome assessment (detection bias) All outcomes	Low risk	**Comment** The blinding of assessors is not reported, however, they were subject to a rigorous training programme and therefore considered as low risk.
Incomplete outcome data (attrition bias) All outcomes	Low risk	**Comment** Seventeen schools were randomly assigned to one of two treatment conditions or to the control condition. All 17 schools remained in the study throughout the pre‐kindergarten year. For the child assessment, the fall 2003 response rate was 95 percent; the spring 2004 pre‐kindergarten response rate was 96 percent; and the kindergarten follow‐up response rate was 80 percent.
Selective reporting (reporting bias)	Low risk	**Comment** Outcomes fully reported
Other bias	Unclear risk	**Comment** Not reported in enough detail

PCER 2008, Project Construct

**Table:** 

***Study characteristics***		
Methods	**Design**: cluster‐randomised controlled trial	
Participants	**Location/setting**: Missouri, USA; full‐day child‐care centres **Sample size**: 231 (21 classrooms) **Mean age**: intervention group = 4.6 years; control group = 4.7 years **Sex**: intervention group = 45.0% boys; control group = 45.4% boys **Race/ethnicity**: 64.8% White, 25.5% African American and 2.8% Hispanic	
Interventions	**Intervention (*n* = 123)**: Project Construct approach; organised around 29 goals for students that are set within a context of four developmental domains (cognitive, representational, socio moral and physical) **Control (*n* = 108)**: treatment as usual; teacher‐developed, generic curricula	
Outcomes	**Eligible outcomes** *Secondary outcomes* Academic achievement, measured with: ∘ Child Math Assessment – Abbreviated composite score (CMA‐A; Klein, 2002) ∘ Comprehensive Test of Phonological Processing (CTOPP; Wagner, 1999), Elision subtest ∘ Peabody Picture Vocabulary Test – 3rd edition (PPVT‐III; Dunn, 1997) ∘ Preschool Comprehensive Test of Phonological & Print Processing (Pre‐CTOPPP; Lonigan, 2002), Elision subtest ∘ Test of Early Reading Ability – 3rd edition (TERA‐3; Reid, 2001) ∘ Test of Language Development (TOLD; Newcomer, 1997), Grammatical Understanding subtest ∘ Woodcock‐Johnson Achievement Test – 3rd edition (WJ‐R‐III; McGrew, 2001) Applied Problems, Letter‐Word Identification & Spelling sub tests Cognitive development, measured with: ∘ Building Blocks, Shape Composition Task (Clements, 2003) Emotional well‐being & social competence, measured with: ∘ Learning Behaviours Scale (LBS; McDermott, 2000), Kindergarten ∘ Social Skills Rating System (SSRS; Gresham, 1990) Social Skills & Problem Behaviours **Ineligible outcomes** Global classroom quality (outcome not detailed in protocol), measured with: ∘ ECERS‐R Teacher‐child interaction, measured with: ∘ Arnett Caregiver Interaction Scale (CIS; Arnett, 1986) (outcome not detailed in protocol) Teacher instructional practices, measured with: ∘ TBRS Book Reading; Oral Language; Phonological Awareness; Print & Letter Knowledge; Written Expression; & Math Concept (Landry, 2002) (outcome not detailed in protocol) **Timing of outcome measurement**: baseline (fall pre‐k), T2 short‐term (spring pre‐k), T3 medium term (spring k)	
Notes	**Study start date**: 2002 **Study end date**: 2005 **Funding source**: Institute of Education Sciences, US Department of Education **Conflict(s) of interest**: members of the PCER research team developed measures included in the PCER Child Assessment Battery: Child Math Assessment; Building Blocks, Shape Composition Task; Pre‐CTOPPP, Elision subtest (this assessment was not commercially available at the time it was selected for inclusion or during data collection. A revised version of the assessment became commercially available after data collection, Dr Lonigan has a financial interest in this measure.). **Comment(s)**: none	

***Risk of bias***		
**Bias**	**Authors' judgement**	**Support for judgement**
Random sequence generation (selection bias)	High risk	**Comment** The Missouri research team identified and recruited a convenience sample of preschools from urban and rural locations in Missouri. Along with the Missouri researchers, Mathematica Policy Research Inc. (MPR) determined the unit of random assignment for this research site. The MPR research staff randomly assigned preschool centres to treatment and control conditions because a preschool operated only one classroom or it was not feasible to vary the curriculum condition within a school.
Timing and recruitment of clusters	High risk	**Comment** Settings were randomised before children were recruited.
Allocation concealment (selection bias)	High risk	**Comment** The MPR research staff randomly assigned preschool centres to treatment and control conditions because a preschool operated only one classroom or it was not feasible to vary the curriculum condition within a school.
Blinding of participants and personnel (performance bias) All outcomes	Unclear risk	**Comment** Not reported in enough detail
Blinding of outcome assessment (detection bias) All outcomes	Low risk	**Comment** The blinding of assessors is not reported; however, they were subject to a rigorous training programme and therefore considered as low risk.
Incomplete outcome data (attrition bias) All outcomes	Low risk	**Comment** A total of 26 classrooms/teachers (13 control and 13 treatment classrooms) were recruited at the beginning of the study. The final sample included 23 teachers and classrooms (11 control and 12 treatment classrooms) because two preschool programmes (housing a total of three preschool classrooms) were dropped from the final study sample. One programme (two classrooms) was closed and another programme (one classroom) was folded into an existing preschool programme because of low enrolment numbers. These changes resulted in a loss of two preschool programmes and three preschool classrooms (two control classrooms and one treatment classroom) in fall of the pre‐kindergarten year. For the child assessment, the fall 2003 response rate was 99 percent, the spring 2004 pre‐kindergarten response rate was 90 percent, and the kindergarten follow‐up response rate was 81%.
Selective reporting (reporting bias)	Low risk	**Comment** All outcomes reported
Other bias	Unclear risk	**Comment** Not reported in enough detail

PCER 2008, Ready, Set, Leap!

**Table:** 

***Study characteristics***		
Methods	**Design**: cluster‐randomised controlled trial	
Participants	**Location/setting**: New Jersey, USA; pre‐kindergarten settings **Sample size**: 286 (39 classrooms) **Mean age**: intervention group = 4.5 years; control group = 4.5 years **Sex**: intervention group = 51.7% boys; control group = 56.8% boys **Race/ethnicity**: 78.4% African American and 20.1% Hispanic	
Interventions	**Intervention (*n* = 149)**: Ready, Set, Leap! A comprehensive, pre‐kindergarten curriculum that combines research‐based instructional approaches with multisensory technology. The curriculum is structured around 9 thematic units, each with 120 detailed lesson plans. **Control (*n* = 137)**: treatment as usual; High/Scope curriculum	
Outcomes	**Eligible outcomes** *Secondary outcomes* Academic achievement, measured with: ∘ Child Math Assessment – Abbreviated composite score (CMA‐A; Klein, 2002) ∘ Comprehensive Test of Phonological Processing (CTOPP; Wagner, 1999), Elision subtest ∘ Peabody Picture Vocabulary Test – 3rd edition (PPVT‐III; Dunn, 1997) ∘ Preschool Comprehensive Test of Phonological & Print Processing (Pre‐CTOPPP; Lonigan, 2002), Elision subtest ∘ Test of Early Reading Ability – 3rd edition (TERA‐3; Reid, 2001) ∘ Test of Language Development (TOLD; Newcomer, 1997), Grammatical Understanding subtest ∘ Woodcock‐Johnson Achievement Test (3rd edition) (WJ‐R‐III; McGrew, 2001) Applied Problems, Letter‐Word Identification & Spelling sub tests Cognitive development, measured with: ∘ Building Blocks, Shape Composition Task (Clements, 2003) Emotional well‐being & social competence, measured with: ∘ Learning Behaviours Scale (LBS; McDermott, 2000), Kindergarten ∘ Social Skills Rating System (SSRS; Gresham, 1990) Social Skills & Problem Behaviours **Ineligible outcomes** Global classroom quality (outcome not detailed in protocol), measured with: ∘ ECERS‐R Teacher‐child interaction, measured with: ∘ Arnett Caregiver Interaction Scale (CIS; Arnett, 1986) (outcome not detailed in protocol) Teacher instructional practices, measured with: ∘ TBRS Book Reading; Oral Language; Phonological Awareness; Print & Letter Knowledge; Written Expression; & Math Concept (Landry, 2002) (outcome not detailed in protocol) **Timing of outcome measurement**: baseline (fall pre‐k), T2 short‐term (spring pre‐k), T3 medium term (spring k)	
Notes	**Study start date**: 2002 **Study end date**: 2005 **Funding source**: Institute of Education Sciences, US Department of Education **Conflict(s) of interest**: members of the PCER research team developed measures included in the PCER Child Assessment Battery: Child Math Assessment; Building Blocks, Shape Composition Task; Pre‐CTOPPP, Elision subtest (this assessment was not commercially available at the time it was selected for inclusion or during data collection. A revised version of the assessment became commercially available after data collection, Dr Lonigan has a financial interest in this measure.). **Comment(s)**: none	

***Risk of bias***		
**Bias**	**Authors' judgement**	**Support for judgement**
Random sequence generation (selection bias)	Low risk	**Comment** MPR grouped classrooms into blocks of two or more and randomly assigned half the classrooms in each block to the treatment group and half to the control group.
Timing and recruitment of clusters	High risk	**Comment** Settings were randomised before children were recruited.
Allocation concealment (selection bias)	High risk	**Comment** Within each block, the highest numbered preschool was assigned to treatment and the next to control, and this process was repeated until all preschools were assigned.
Blinding of participants and personnel (performance bias) All outcomes	Unclear risk	**Comment** Not reported in enough detail
Blinding of outcome assessment (detection bias) All outcomes	Low risk	**Comment** The blinding of assessors is not reported, however, they were subject to a rigorous training programme and therefore considered as low risk.
Incomplete outcome data (attrition bias) All outcomes	Low risk	**Comment** Thirty‐nine classrooms were randomly assigned to treatment and control conditions. All 39 classrooms remained in the study throughout the pre‐kindergarten year. For the child assessment, the fall 2003 response rate was 96%; the spring 2004 pre‐kindergarten response rate was 92%; and the kindergarten follow‐up response rate was 87%.
Selective reporting (reporting bias)	Low risk	**Comment** All outcomes reported
Other bias	Unclear risk	**Comment** Not reported in enough detail

Peisner‐Feinberg 2019

**Table:** 

***Study characteristics***		
Methods	**Design**: cluster‐randomised controlled trial	
Participants	**Location/setting**: North Carolina, USA; pre‐kindergarten settings in 2 counties—in one county, the programme was administered by the public school system and in the other by a local Smart Start partnership **Sample size**: 582 (140 classrooms) **Mean age**: intervention group = 4.5 years; control group = 4.5 years **Sex**: intervention group = 51.7% boys; control group = 56.8% boys **Race/ethnicity**: 163 children (intervention group = 132; control group = 31) were Spanish‐speaking dual language learners (DLLs)	
Interventions	**Intervention (*n* = 473)**: North Carolina pre‐kindergarten Programme—range of different, approved, early childhood curricula, including Creative Curriculum, Opening the World of Learning, High/Scope Preschool Curriculum, Tools of the Mind, Investigator Club Prekindergarten Learning System and Passports: Experiences for Pre‐K Success **Control(*n* = 109)**: wait‐list	
Outcomes	**Eligible outcomes** *Secondary outcomes* Academic achievement, measured with: ∘ Batería III Woodcock‐Muñoz Pruebas de Aprovechamiento (Muñnoz‐Sandoval, 2005) ∘ Pre‐IPT Oral (IDEA Proficiency Test; Stansfield, 1990) ∘ Woodcock‐Johnson Achievement Test – 3rd edition (WJ‐R‐III; McGrew, 2001) Applied Problems, Letter‐Word Identification, Quantitative Concepts, Passage Comprehension, Picture Vocabulary sub tests Cognitive development, measured with: ∘ Forward Digit Span English/Spanish (reference not cited) ∘ Head‐Toes‐Knees‐Shoulders Test (HTKS; Ponitz, 2009) Social competence & emotional well‐being, measured with: ∘ Social Skills Improvement System (SSiS; Flowers, 2008), Social Skills and Problem Behaviours sub scales **Ineligible outcomes**: none **Timing of outcome measurement**: baseline (fall pre‐k), T2 short‐term (spring pre‐k)	
Notes	**Study start date**: 2017 **Study end date**: 2018 **Funding source**: North Carolina Division of Child Development and Early Education, Department of Health and Human Services **Conflict(s) of interest**: none reported **Comment(s)**: none	

***Risk of bias***		
**Bias**	**Authors' judgement**	**Support for judgement**
Random sequence generation (selection bias)	Low risk	**Comment** Within each county, children who were eligible for and had applied to the NC Pre‐K programme were randomly assigned into either the treatment (NC Pre‐K) or control (wait list) group. In county A, randomisation was conducted using simple random sampling. In county B, randomisations occurred within income strata to meet programme requirements (80% at or below 75% state median income and 20% above). Each county provided the researchers with a subset of their lists of eligible applicants for randomisations into treatment (NC Pre‐K) and control (wait list) groups using a unique identification number. Before assignment, the programme confirmed that all children were eligible for NC Pre‐K, based on criteria related to age and family income.
Timing and recruitment of clusters	Low risk	**Comment** Children were randomised first
Allocation concealment (selection bias)	Low risk	**Comment** The number of assignments to the treatment group was made based on the number of slots the programme needed to fill, with remaining applicants in the pool randomly assigned to the control group.
Blinding of participants and personnel (performance bias) All outcomes	Unclear risk	**Comment** Unclear whether any blinding was conducted.
Blinding of outcome assessment (detection bias) All outcomes	Low risk	**Comment** All data collectors were trained to specified certification/proficiency standards on all measures before gathering data.
Incomplete outcome data (attrition bias) All outcomes	Low risk	**Comment** The rate of attrition was low overall; 570 children remained in the study from fall to spring during the pre‐k year and 12 had only fall data. Comparisons of children who remained in the study (i.e., those with both fall and spring data) vs those who left the study (i.e., those with only fall data) revealed a few significant differences between these two groups for initial skill levels and demographic characteristics.
Selective reporting (reporting bias)	Low risk	**Comment** All outcomes reported
Other bias	Low risk	**Comment** None identified

Puma 2005

**Table:** 

***Study characteristics***		
Methods	**Design**: cluster‐randomised controlled trial	
Participants	**Location/setting**: 23 different states across the USA; 383 randomly selected Head Start centres that had sufficient numbers of applicants for the 2003–03 programme year to allow for the creation of a control group without Head Start slots to go unfilled **Sample size**: 3723 **Mean age**: not reported; 2 age cohorts, newly‐entering 3 and 4 year olds **Sex**: 46.8% boys **Race/ethnicity**: 2 age cohorts. Age 3 cohort: 32.8% Black; 37.4% Hispanic; 29.8% White/Other. Age 4 cohort: 17.5% Black; 51.6% Hispanic; 30.8% White/Other	
Interventions	**Intervention (*n* = 2360)**: Head Start. Since its beginning in 1965 as part of the war on poverty, Head Start's goal has been to boost the school readiness of low‐income children. It is based on the ‘whole child’ model and provides comprehensive services that include preschool education, medical, dental and mental health care, nutrition services and efforts to help parents foster their child's development. The average number of hours spent per week on the programme was between 24 and 28 h. **Control (*n* = 1363)**: no Head Start; control group did not have access to Head Start but could enrol in other early childhood programmes or non‐Head Start services selected by their parents	
Outcomes	**Eligible outcomes** *Secondary outcomes* Academic achievement, measured with: ∘ Letter Naming task (Head Start Quality Research Centre, [QRC; Head Start QRC, 2002]) ∘ Peabody Picture Vocabulary Test (PPVT; Dunn, 2007) ∘ McCarthy Scales of Children's Abilities, Draw‐A‐Design Task (McCarthy, 1970) ∘ Preschool Comprehensive Test of Phonological and Print Processing (CTOPP; Wagner, 1999), Print Awareness and Elision Subtests ∘ Story and Print Concepts Task (Clay, 1985, Mason, 1989, Teale, 1988) ∘ Writing Name task, modelled after the Comprehensive Assessment Programme (CAP) Early Childhood Diagnostic Instrument (Mason, 1989) ∘ Woodcock‐Johnson composite scoresAcademic Applications, Academic Skills, Applied Problems, Basic Reading, Calculation, Math Reasoning, Oral Comprehension, Passage Comprehension, Phonetic Skills/Word Attack, Letter‐Word Identification, Quantitative Concepts, Spelling, Writing Sample sub tests (McGrew, 2001) ∘ Spanish Language: Receptive Vocabulary (TVIP; Dunn, 1986); Bateria WM Identificacion de letras y palabra Cognitive development, measured with: ∘ Colour names and counting sub tests of the CAP Early Childhood Diagnostic Instrument (Clay, 1985; Mason 1989; Teale 1988) Emotional well‐being and social competence, measured with: ∘ Sustained Attention Task (Adapted Leiter‐R Assessor Report (Roid 2003a) – not reported ‘limitation made it difficult to interpret the scores and thus the Leiter was eliminated from the analysis’. (pp. 3–6) Health development, measured with: ∘ Child received dental care ∘ Child needs ongoing health care ∘ Child had care for injury in the last month ∘ Child has health insurance coverage ∘ Child's overall health status is excellent/good **Ineligible outcomes** Parenting style, measured with: ∘ Impact on parenting practices (parent spanked, used time out, read to child in last week), parental safety practices scale, family cultural enrichment scale, parenting style (authoritarian, authoritative, neglectful, permissive) **Timing of outcome measurement**: baseline; post test (spring of 1st grade), Age 3 cohort was followed for a total of 4 years – 2 years of Head Start participation (i.e., Head Start year and age 4, kindergarten and 1st Grade) and age 4 cohort was followed for 3 years – 1 year of Head Start participation (i.e., Head Start year, kindergarten and 1st Grade)	
Notes	**Study start date**: 2002 **Study end date**: 2006 **Funding source**: Office of Planning, Research and Evaluation, Administration for Children and Families, US Department of Health and Human Services **Conflicts of interest**: none stated **Comment(s)**: data were not reported fully and we were unable to make contact with the author (Table 1)	

***Risk of bias***		
**Bias**	**Authors' judgement**	**Support for judgement**
Random sequence generation (selection bias)	Unclear risk	**Comment** While efforts were made to randomised children, the randomisation procedure is not described and Individual Head Start centres applied their usual criteria for enroling children into the programme based on need.
Timing and recruitment of clusters	High risk	**Comment** Settings were randomised before children were recruited.
Allocation concealment (selection bias)	High risk	**Comment** Allocation was based on child need and two lists were created to create the treatment and control groups.
Blinding of participants and personnel (performance bias) All outcomes	Unclear risk	**Comment** Blinding to treatment was not possible but this may not have impacted on performance bias.
Blinding of outcome assessment (detection bias) All outcomes	Unclear risk	**Comment** Unclear whether outcome assessors were blinded. Assessments based on parent and teacher report were also included, as well as assessments conducted in the family home may also be subject to bias.
Incomplete outcome data (attrition bias) All outcomes	Unclear risk	**Comment** Attrition is not reported in detail.
Selective reporting (reporting bias)	Low risk	**Comment** All outcome measures reported.
Other bias	Unclear risk	**Comment** None identified

Raver 2009

**Table:** 

***Study characteristics***		
Methods	**Design**: cluster‐randomised controlled trial	
Participants	**Location/setting**: Chicago, USA; Head Start classrooms **Sample size**: 467 (35 classrooms); 543 randomised at baseline, 509 remained in the study by spring. Data reported on 449 (observational assessments were conducted on a stratified, randomly selected sub sample (*n* = 172), approximately 6 children per classroom) **Mean age**: 49.4 (SD 8.0) months **Sex**: 47% boys **Race/ethnicity**: 65% Black, 28% Hispanic	
Interventions	**Intervention (*n* = 238; 2 classrooms at 9 sites, total of 18 classrooms)**: Chicago School Readiness Project (CSRP); CRSP is based on several theoretical models of preschool children's behavioural problems and aims to improve low‐income preschool children's school readiness by improving their behavioural and emotional adjustment. It includes intensive training strategies for teachers to support them to provide effective support, have better classroom management and avoid teacher burnout. Two additional components were introduced: a weekly Mental Health Consultant provided 20 weeks of coaching to support teachers to implement the new techniques; and stress reduction workshops. The intervention was trauma‐informed, recognising the impact of poverty‐related risks to children's mental health and school adjustment, and 3–5 children in each classroom were provided with a child‐focused mental health consultation in the spring of pre‐k. **Control (*n* = 229; 2 classrooms at 8 sites, 1 classroom at 1 site, total of 17 classrooms)**: teaching assistant; each class was assigned a teaching assistant to control for the additional staff (mental health consultant) in the intervention group	
Outcomes	**Eligible outcomes** *Secondary outcomes* Academic achievement, measured with: ∘ Letter naming ∘ Early math skills score ∘ Peabody Picture Vocabulary Test – 3rd edition (PPVT‐III; Dunn, 1997) Emotional well‐being & social competence, measured with: ∘ The Achenbach System of Empirically Based Assessment (ASEBA) Caregiver‐Teacher Report Form (C‐TRF; Achenbach, 2001) ∘ Behaviour Problems Index (BPI; Zill, 1990) ∘ Penn Interactive Peer Play Scale (PIPPS; Fantuzzo, 2007; Milfort, 2002), administered to sub sample ∘ Preschool Self‐Regulation Assessment (PSRA; Smith‐Donald, 2007), using 2 Executive Function tasks (Balance Beam [Murray, 2002] & Pencil Tap adapted from peg‐tapping task [Blair, 2002; Diamond, 1996]) and 4 effortful control ‘delay’ tasks (Toy Wrap, Toy Wait, Snack Delay, & Tongue Task [Murray 2002]) ∘ 2 additional PSRA (Smith‐Donald, 2007) tasks (Gift Return & Block Task) were not reported because they ‘yielded data with insufficient variance and were therefore omitted’ (p. 369) from the analysis **Ineligible outcomes** Classroom environment, measured with: ∘ Classroom Assessment Scoring System (CLASS; La Paro, 2004) (outcome not detailed in protocol) ∘ Teacher characteristics (outcome not detailed in protocol) ∘ Site characteristics (outcome not detailed in protocol) Child environment, measured with: ∘ Early Childhood Environment Rating Scale (ECERS‐R; Harms, 2003) (outcome not detailed in protocol) **Timing of outcome measurement**: baseline (pre pre‐k), T2 short‐term (post pre‐k), T3 medium term (post kindergarten), T4 long term (3rd grade)	
Notes	**Study start date**: 2004 **Study end date**: 2006 **Funding source**: Office of Education, Washington Bureau of Research **Conflict(s) of interest**: none reported **Comment(s)**: none	

***Risk of bias***		
**Bias**	**Authors' judgement**	**Support for judgement**
Random sequence generation (selection bias)	Unclear risk	**Quote** Pairwise randomisation procedure unspecified ‘classrooms within each site were randomly selected for participation’ (p. 2)
Timing and recruitment of clusters	High risk	**Comment** Settings were randomised before children were recruited.
Allocation concealment (selection bias)	Unclear risk	**Comment** Not reported in enough detail
Blinding of participants and personnel (performance bias) All outcomes	Unclear risk	**Comment** Not reported in enough detail
Blinding of outcome assessment (detection bias) All outcomes	Low risk	**Quote** ‘Trained observers, who were blind to randomization, assessed the quality of children's classrooms using the Classroom Assessment Scoring System’ (p. 17)
Incomplete outcome data (attrition bias) All outcomes	Unclear risk	**Quote** ‘At baseline, a total of 543 children participated in CSRP. By the spring, the number of participating children was reduced to 509. Nearly all of the exits were due to children voluntarily leaving the Head Start programme, though one child was requested to leave the Head Start programme and one parent opted to withdraw her child from participating in CSRP’. (p. 10) Analyses reports on *n* = 449, missing data appears to account for lower numbers reported
Selective reporting (reporting bias)	Unclear risk	**Comment** Not reported in enough detail
Other bias	Unclear risk	**Comment** Not reported in enough detail

Soloman 2018

**Table:** 

***Study characteristics***		
Methods	**Design**: cluster‐randomised controlled trial	
Participants	**Location/setting**: Ontario, Canada; urban daycare centres, 20 sites **Sample size**: 195 **Mean age**: intervention group = 45.1 (range 37.2–55.34) months; control group = 45.9 (range 36.5–62.3) months **Sex**: intervention group = 54.7% boys; control group = 52.8% boys **Race/ethnicity**: 100% Black	
Interventions	**Intervention (*n* = 106, 10 classes)**: Tools of the Mind (Bodrova, 2007); a play‐based, preschool and kindergarten curriculum that emphasises self‐control, language and literacy skills. The present study involved the preschool version of the programme. **Control (*n* = 89, 10 classes)**: Playing to Learn (YMCA PTL; Eden, 2001; Martin 2015); a play‐based, preschool curriculum. A critical difference between Tools and YMCA PTL is that, whereas Tools is a more teacher‐directed, prescribed approach, YMCA PTL is a child‐centred, emergent curriculum. The teacher's primary roles are to establish a safe, secure, social environment and to facilitate learning through play, following the child's interest. The set up of the physical environment is seen as essential to encouraging quality play (but not necessarily pretend play). YMCA PTL classrooms resemble home‐like environments. A wide variety of materials are available to encourage play that supports children's social, emotional and academic learning. Children who become disengaged may be enticed by different aspects of the environment to re‐engage in play.	
Outcomes	**Eligible outcomes** *Primary outcome* School readiness, measured with: ∘ Early Development Instrument (EDI; Janus, 2007) *Secondary outcomes* Academic achievement, measured with: ∘ Expressive Vocabulary Test (EVT‐4; Williams, 2007) ∘ Get Ready to Read (GRTR‐R; Whitehurst, 2001) ∘ Peabody Picture Vocabulary Test – 4th edition (PPVT‐4; Dunn, 1997) ∘ Point‐to‐X (PTX; Wynn, 1992) Cognitive development, measured with: ∘ Day/Night task (Gerstadt, 1994) ∘ Head‐To‐Toes version of the Head‐Shoulders‐Knees‐Toes task (HTT; Ponitz, 2009) Emotional well‐being & social competence, measured with: ∘ Strengths and Difficulties Questionnaire – Parent and Teacher versions (SDQ‐P, SDQ‐T; Goodman, 1997, 1999) ∘ Social Competence and Behaviour Evaluation Scale (SCBE‐30; LaFreniere, 1995) **Ineligible outcomes**: none **Timing of outcome measurement**: baseline, T2 (8 months later), T3 (post‐intervention)	
Notes	**Study start date**: March 2012 **Study end date**: June 2013 **Funding source**: Hospital for Sick Children, Canada Research Chairs Programme, and support in kind by the YMCA of Canada **Conflict(s) of interest**: none reported **Comment(s)**: data were not reported fully and we were unable to make contact with the author (Table 1)	

***Risk of bias***		
**Bias**	**Authors' judgement**	**Support for judgement**
Random sequence generation (selection bias)	Unclear risk	**Comment** Not reported in detail
Timing and recruitment of clusters	Low risk	**Comment** Settings were randomised after children were recruited.
Allocation concealment (selection bias)	Unclear risk	**Comment** Not reported in detail
Blinding of participants and personnel (performance bias) All outcomes	Unclear risk	**Comment** Not reported in detail
Blinding of outcome assessment (detection bias) All outcomes	Low risk	**Quote** ‘The observers were blind to the study hypotheses and to the assignment of sites to the two curricula’ (p. 10)
Incomplete outcome data (attrition bias) All outcomes	Low risk	**Comment** While attrition was high in the sample, analysis to determine the impact of attrition was conducted.
Selective reporting (reporting bias)	Low risk	**Comment** All outcomes reported
Other bias	Unclear risk	**Comment** Not reported in enough detail

Van de Riet 1972

**Table:** 

***Study characteristics***		
Methods	**Design**: randomised controlled trial	
Participants	**Location/setting**: Florida, USA **Sample size**: 86 **Mean age**: 62 months **Sex**: not reported **Race/ethnicity**: 100% Black	
Interventions	**Intervention (*n* = 44)**: Learning to Learn Programme; a sequential early childhood approach organised and operated so that it (a) is appropriate to the stage of cognitive development of the child, (b) makes maximal use of the child's abilities, (c) uses a planned sequence of environmental stimulation based on a knowledge of the stages of cognitive development, (d) emphasises the process of learning, (e) guides and structures the learning experiences with the goal of self‐support and coping on his own rather than presenting the child with a large amount of random, unorganised stimulation. The teacher facilitates and evaluates the child's learning and the co‐operation of parents is enlisted to supplement the school programme with a home programme. Parents participate in monthly instructional sessions to highlight their role in their child's educational development. Children participated in the programme for either 2 or 3 consecutive (1968–69 to 1970–71) years through first grade. **Control (*n* = 42)**: Office of Economic Opportunity sponsored day care, kindergarten and public school first grade	
Outcomes	**Eligible outcomes** *Secondary outcomes* Academic achievement, measured with: ∘ Spache Diagnostic Reading Test Instructional Reading Level (Spache, 1963) ∘ Stanford Achievement Test II Arithmetic sub‐test (Madden, 1970) ∘ Illinois Test of Psycholinguistic Abilities (ITPA; McCarthy, 1961) Vocal Encoding & Audio‐Vocal Association sub tests ∘ School grades Cognitive development, measured with: ∘ Stanford‐Binet Intelligence Scale (Terman, 1960) ∘ Bender Gestalt Perceptual motor (error score) (Koppitz, 1964) Emotional well‐being and social competence, measured with: ∘ Self‐concept Florida Key (Purkey, 1973) ∘ Achievement motivation – teacher ratings ∘ Citizenship grades (social skills) **Ineligible outcomes**: none **Timing of outcome measurement**: baseline (fall pre‐k), T2 short‐term (end of kindergarten), T3 long‐term (post 2nd Grade)	
Notes	**Study start date**: 1968 **Study end date**: 1971 **Funding source**: Division of Research & Evaluation, Office of Child Development, US Department of Health, Education & Welfare **Conflict(s) of interest**: none reported **Comment(s)**: none	

***Risk of bias***		
**Bias**	**Authors' judgement**	**Support for judgement**
Random sequence generation (selection bias)	Unclear risk	**Comment** Not reported
Allocation concealment (selection bias)	High risk	**Comment** The children were divided and placed either in the E group or C group by matching their performance on the Stanford Binet Intelligence Scale.
Blinding of participants and personnel (performance bias) All outcomes	Unclear risk	**Comment** Not reported
Blinding of outcome assessment (detection bias) All outcomes	Unclear risk	**Comment** Not reported
Incomplete outcome data (attrition bias) All outcomes	Unclear risk	**Comment** Not reported
Selective reporting (reporting bias)	Unclear risk	**Comment** Not reported
Other bias	Unclear risk	**Comment** Not reported

Warden 1988

**Table:** 

***Study characteristics***		
Methods	**Design**: randomised controlled trial	
Participants	**Location/setting**: Virginia, USA; elementary school setting **Sample size**: 80 **Mean age**: not reported, range 4–6 years **Sex**: not reported **Race/ethnicity**: not reported	
Interventions	**Intervention (*n* = 40)**: a developmentally appropriate curriculum, as advocated by the National Association of the Education of Young Children on kindergarten readiness and achievement **Control (*n* = 40)**: treatment as usual; children within the same school district that did not attend the preschool programme	
Outcomes	**Eligible outcomes** *Primary outcomes* School readiness, measured with: ∘ Kindergarten Readiness Test (Larson, 1988) Academic achievement, measured with: ∘ Metropolitan Readiness Test *–* Pre‐reading, Beginning Reading, Quantitative, Story Comprehension sub‐tests **Ineligible outcomes**: none **Timing of outcome measurement**: baseline, post test (short‐term)	
Notes	**Study start date**: 1996 **Study end date**: 1998 **Funding source**: Office of Education, Washington Bureau of Research **Conflict(s) of interest**: none reported **Comment(s)**: none	

***Risk of bias***		
**Bias**	**Authors' judgement**	**Support for judgement**
Random sequence generation (selection bias)	Unclear risk	**Comment** Not reported
Allocation concealment (selection bias)	Unclear risk	**Comment** Not reported
Blinding of participants and personnel (performance bias) All outcomes	Unclear risk	**Comment** Not reported
Blinding of outcome assessment (detection bias) All outcomes	Unclear risk	**Comment** Not reported
Incomplete outcome data (attrition bias) All outcomes	Unclear risk	**Comment** No attrition data provided
Selective reporting (reporting bias)	High risk	**Comment** All measures not reported in report
Other bias	Unclear risk	**Comment** Not reported in enough detail

Weikart 1967

**Table:** 

***Study characteristics***		
Methods	**Design**: cluster‐randomised controlled trial	
Participants	**Location/setting**: Indiana, USA; nursery school **Sample size**: 123 (10 classrooms) **Mean age**: not reported, 3 and 4 years old **Sex**: not reported **Race/ethnicity**: 100% African American	
Interventions	**Intervention (*n* = 58)**: High/Scope Perry Preschool; delivered to economically disadvantaged children with family circumstances, including limited education, low income or welfare, many single parents and high risk of school failure based on a battery of assessments. The High/Scope was high quality, active learning delivered during 3‐h classes, 5 days a week with a 1.5‐h home visit each week. It is loosely based on Piaget's developmental theories. Each child constructs their own learning experience within a classroom environment designed by the teaching staff. This plan‐do‐review cycle develops the child's initiative, sense of responsibility, problem‐solving ability, social co‐operation and individual competence in a variety of psychomotor and intellectual skills. **Control (*n* = 65)**: no treatment; control children remained at home within the family, without a programme	
Outcomes	**Eligible outcomes** *Secondary outcomes* Academic achievement, measured with: ∘ California Achievement Test ∘ Illinois Test of Psycholinguistic Abilities (ITPA; McCarthy, 1961) ∘ Leiter International Performance Scale (Roid, 2003a) ∘ Peabody Vocabulary Test – 3rd edition (PPVT‐III; Dunn, 1997) Cognitive development, measured with: ∘ Stanford‐Binet Intelligence Scale (Terman, 1960) ∘ Wechsler Intelligence Scales (Wechsler, 2002) Emotional well‐being & social competence, measured with: ∘ Pupil Behaviour Inventory ∘ Ypsilanti Rating Scale (Kamii, 1967) **Ineligible outcomes** (not included in protocol) Long term education, social and economic outcomes, measured with: ∘ School records 5–19 years ∘ Youth Interview age 15 ∘ Young Adult Interview ∘ Case Study Interview ∘ Social Services case files ∘ Police arrest records ∘ Earning & income status (home ownership, car ownership, welfare receipt) age 27 ∘ Commitment to marriage age 27 ∘ Educational performance by age 27 **Timing of outcome measurement**: baseline (fall 1962); T2 short‐term (spring 1963, end of pre‐k); T3 medium term (spring 1964, end of k); T4 long term (spring 1965, end of 1st grade), data were collected for 4 cohorts of children (0, 1, 2, 3). Other follow‐up data have been collected on this cohort, well into adulthood.	
Notes	**Study start date**: 1962 **Study end date**: 2012 **Funding source**: Institute of Education Sciences, US Department of Education and US Department of Health and Human Services **Conflict(s) of interest**: none stated **Comment(s)**: none	

***Risk of bias***		
**Bias**	**Authors' judgement**	**Support for judgement**
Random sequence generation (selection bias)	Low risk	**Quote** Children were ranked in order of their Stanford‐Binet scores, then sorted into 2 groups (odd/even). If these groups had unequal sex ratios or unequal SES ratings, they were equated by exchanging children with similar Standord‐Binet scores. Groups roughly equated in this manner were assigned to treatment conditions by ‘flipping a coin’ (p. 7).
Timing and recruitment of clusters	Low risk	**Comment** Settings were randomised after children were recruited.
Allocation concealment (selection bias)	High risk	**Comment** Following Waves 0 and 1, these procedures had to be qualified somewhat by practical considerations. Younger siblings of children already in the project were assigned to the same group, so assignment was by family rather than child. Occasional exchanges of children between groups had to be made because of the inconvenience for half‐day preschool for working mothers and transportation difficulties for some families.
Blinding of participants and personnel (performance bias) All outcomes	High risk	**Comment** Following Waves 0 and 1, these procedures had to be qualified somewhat by practical considerations. Younger siblings of children already in the project were assigned to the same group, so assignment was by family rather than child. Occasional exchanges of children between groups had to be made because of the inconvenience for half‐day preschool for working mothers and transportation difficulties for some families.
Blinding of outcome assessment (detection bias) All outcomes	Unclear risk	**Quote** ‘During the preschool years, testing was not “blind.” The examiners that administered the standardised tests (Stanford‐Binet, PPVT, ITPA, Leiter) knew which children were attending preschool and which were not’. (p. 31) Once children entered school, testing became ‘blind’. Post‐treatment testers had little if any knowledge of the purposes of the experiment.
Incomplete outcome data (attrition bias) All outcomes	Low risk	**Comment** Attrition was very low and is reported.
Selective reporting (reporting bias)	Low risk	**Comment** All outcomes are reported
Other bias	Low risk	**Comment** None identified

Yousafzai 2018

**Table:** 

***Study characteristics***		
Methods	**Design**: cluster‐randomised controlled trial	
Participants	**Location/setting**: Sindh, Pakistan; pre‐school **Sample size**: 340 (10 villages) **Mean age**: intervention group = 55.2 (SD 7.6) months; control group = 54.3 (SD 7.8) months **Sex**: intervention group = 50.6% boys; control group = 51.2% boys **Race/ethnicity**: not reported	
Interventions	**Intervention (*n* = 170; 5 villages)**: Youth Leaders for Early Childhood Assuring Children are Prepared for School (LEAPS) early childhood care and education curriculum; a new programme based on the pedagogy of the High Scope early childhood education approach and aligned with the Government of Pakistan's early childhood education curriculum. The seven learning areas are: (1) approaches to learning; (2) social‐emotional development; (3) physical development and health; (4) language, literacy and communication; (5) mathematics; (6) creative arts; and (7) my world. Female community youth leaders (aged 18–24 years) recruited from within each community supported family and community‐engagement strategies by holding quarterly parent meetings, monthly meetings with the village community health worker and the local primary school teacher, and sharing information with the National Commission of Human Development (NCHD) and district officials in quarterly town hall meetings. Two classes were run per day for 5 days per week; class duration was 3 h. Leaders had completed at least 10 years of schooling and were given 1‐month basic training and received at least twice monthly support from a mentor. **Control (*n* = 170; 5 villages)**: treatment as usual; control children had access to usual educational services provided by the government	
Outcomes	**Eligible outcomes** *Primary outcome* School readiness, measured with: ∘ International Development and Early Learning Assessment (IDELA; Save the Children, 2015) *Secondary outcomes* Academic achievement, measured with: ∘ IDELA Emergent Mathematics subtest (IDELA; Save the Children, 2015) ∘ IDELA Emergent Literacy subtest (IDELA; Save the Children, 2015) Cognitive development, measure with: ∘ Corsi Block‐Tapping Test (Noël, 2009) ∘ Dimensional Change Card Sort (DCCS; Zelazo, 2006) Physical development, measured with: ∘ IDELA Fine Motor subtest (IDELA; Save the Children, 2015) ∘ IDELA Gross Motor subtest (IDELA; Save the Children, 2015) Emotional well‐being and social competence, measured with ∘ IDELA Socioemotional subtest (IDELA; Save the Children, 2015) ∘ IDELA Self‐regulation subtest (IDELA; Save the Children, 2015) ∘ Knock and Tap Assessment (Molfese, 2010) ∘ Peg Tap Assessment (Molfese, 2010) **Ineligible outcomes**: none **Timing of outcome measurement**: baseline (December 2015), post‐test short‐term (August to September 2016)	
Notes	**Study start date**: November 2015 **Study end date**: September 2016 **Funding source**: co‐funded by the Saving Brains Programme, Grand Challenges Canada and UNICEF **Conflict(s) of interest**: none stated **Comment(s)**: none	

***Risk of bias***		
**Bias**	**Authors' judgement**	**Support for judgement**
Random sequence generation (selection bias)	Unclear risk	**Comment** Ten clusters were randomly sampled, stratified by rural or peri‐urban location. Using simple randomisation, five clusters were assigned to the intervention group and five clusters were assigned to the control group.
Timing and recruitment of clusters	High risk	**Comment** Settings were randomised before children were recruited.
Allocation concealment (selection bias)	Unclear risk	**Comment** Not enough detail reported
Blinding of participants and personnel (performance bias) All outcomes	High risk	**Comment** Blind**i**ng was not possible because as part of the community‐based strategy, the LEAPs ECCE centres were signposted in villages and some data collection visits took place near to the centres.
Blinding of outcome assessment (detection bias) All outcomes	Low risk	**Comment** The data collection team, comprising four data collectors, four community‐based child development assessors and a field supervisor, were independent of the intervention team.
Incomplete outcome data (attrition bias) All outcomes	Low risk	**Comment** Attrition reported
Selective reporting (reporting bias)	Low risk	**Comment** All outcomes reported
Other bias	Low risk	**Comment** None identified

Abbreviations: ADHD, attention deficit hyperactivity disorder; ECERS, Early Childhood Environment Rating Scale; ITT, intention to treat; *n*, number; NCSER, National Center for Special Education Research; PATHS, Promoting Alternative Thinking Strategies Programme; SD, standard deviation; T, time; TAU, treatment as usual; US, United States.

Characteristics of excluded studies [ordered by study ID]

**Table:** 

Study	Reason for exclusion
Di Lorenzo 1967	**Design**: a range of different kindergarten programmes were offered; however, the control setting is not described. Children were randomly assigned based on socio‐economic status, and only this comparison was made post‐intervention.
Gür 2017	**Design**: duration and intensity of intervention did not meet Criteria for considering studies for this review
Hodges 1967	**Design**: children were selected from a ‘severely psychosocially disadvantaged population’ (p. 11) and were included on the basis of low and extremely low scores on the Stanford‐Binet Intelligence Test. The intervention was designed for ‘retarded kindergarten age’ (p. 11) children and did not meet the Criteria for considering studies for this review.
O'Connor 2014	**Design**: duration and intensity of intervention did not meet Criteria for considering studies for this review
Pears 2012	**Design**: duration and intensity of intervention did not meet Criteria for considering studies for this review
Ramey 1974	**Population**: children were randomised to treatment and control and received the intervention from birth to 5 years old. It is not possible to separate the effects of the intervention delivered between birth and 3 years and the age of children we specified in our protocol; therefore, does not meet the Criteria for considering studies for this review.
Tzuriel 1998	**Design**: duration and intensity of intervention did not meet Criteria for considering studies for this review
Tzuriel 1999	**Design**: duration and intensity of intervention did not meet Criteria for considering studies for this review

Characteristics of studies awaiting classification [ordered by study ID]

Alpern 1966

**Table:** 

Methods	**Design**: cluster‐randomised controlled trial
Participants	**Location/setting**: Indiana, USA; nursery school **Sample size**: 44 **Age**: mean age not reported, ‘4 year olds’ (p. 1) **Sex**: not reported **Race/ethnicity**: 100% Negro (understood to be Black or African American)
Interventions	**Intervention (*n* = 22)**: ‘enrichment program’ (p. 1); aimed to (1) increase the children's language skills; (2) develop positive attitudes toward concepts of teacher, learning and school; and (3) increase knowledge of middle‐class values and experiences. Operated 3 times per week over a 7‐month period **Control(*n* = 22)**: ‘the control group did not have this experience’ (p. 1)
Outcomes	**Eligible outcomes** *Primary outcome* School readiness, measured with: ∘ Metropolitan Readiness Test (Bixler, 1958) *Secondary outcome* Cognitive development, measured with: ∘ Stanford‐Binet Intelligence Scale (Terman, 1960) **Ineligible outcomes**: none **Timing of outcome measurement**: baseline and post test
Notes	**Study start date**: not reported **Study end date**: not reported **Funding source**: US Government **Conflicts of interest**: none stated **Comment(s)**: no results were reported and we were unable to make contact with the author who is now deceased

Ametjian 1965

**Table:** 

Methods	**Design**: cluster‐randomised controlled trial
Participants	**Location/setting**: California, USA; nursery school **Sample size**: 60 at baseline **Mean age**: intervention = 48.38 (SD 4.3) months; control = 47.03 (SD 6.7) months **Sex**: intervention = 41.7% boys; control = 50.0% boys **Race/ethnicity**: intervention = 54.2% African‐American, 45.8% White; reported as control = 52.7% African‐American, 47.2% White
Interventions	**Intervention (*n* = 24 at baseline)**: Lincoln Preschool enrichment programme; aimed to (1) increase the children's language skills; (2) develop positive attitudes toward concepts of teacher, learning and school; and (3) increase knowledge of middle‐class values and experiences. Operated 3 times per week over a 7‐month period **Control (*n* = 36 at baseline)**: not reported
Outcomes	**Eligible outcomes** *Secondary outcomes* Cognitive development, measured by: ∘ mental age in months Emotional well‐being & social competence, measured with: ∘ San Francisco Social Competency Scale (no reference cited) ∘ initiative‐responsibility score (no reference cited) **Ineligible outcomes**: none **Timing of outcome measurement**: post test
Notes	**Study start date**: 1964 **Study end date**: not reported **Funding source**: US Government **Conflicts of interest**: none stated **Comment(s)**: data were not reported fully and we were unable to make contact with the author who is now deceased

Karnes 1969

**Table:** 

Methods	**Design**: cluster‐randomised controlled trial
Participants	**Location/setting**: Champaign‐Urbana, Illinois, USA; various preschool settings **Sample size**: 75 (intervention only, control group size not reported) **Mean age**: not reported **Sex**: 50% boys **Race/ethnicity**: 67% Black
Interventions	**Intervention (*n* = 75)**: 5 preschool interventions **Traditional**: in which the major goals were to promote the personal, social, motor, and general language development of the children. Teachers were instructed to capitalise on opportunities for incidental and informal learning, to encourage the children to talk and to ask questions, and to stimulate their interest in the world around them. Music, story, and art activities were scheduled regularly. Outdoor play was a part of the daily routine; indoor play focused on a doll and housekeeping centre, a vehicle and block centres, and a small toy centres. **Community‐integrated**: provided a traditional nursery school experience similar to the one above. These centres were licensed by the state and were sponsored by community groups and classes were composed predominantly of middle‐ and upper‐class Caucasian children. **Montessori**: where the specific nature of the ‘prepared environment’ raised the level of structure within the Montessori classroom beyond that of the two traditional programmes. The Montessori teacher did not, however, maintain the high level of specific control over the actions of the children required by the teachers in the two highly structured programmes. Structure in the Montessori programme derived not from direct teacher‐child interaction but from the prescribed manner in which the child learned from the materials. **Ameliorative**: in which verbalisations in conjunction with the manipulation of concrete materials were considered to be the most effective means of establishing new language responses. Each class unit (*n* = 15) was divided into three groups on the basis of Binet IQ with one teacher for each group. The daily schedule was divided into 3 20‐min structured learning periods: math concepts; language arts and reading readiness; and science‐social studies. The low pupil‐teacher ratio allowed for differentiation of instruction to provide a high success ratio for each child. Immediate correction of incorrect responses (often through the repetition of model sentences or through duplicate layouts of small manipulative materials) and reinforcement of appropriate responses (usually through praise) assured the children of their competencies in handling curricular requirements and enhanced their intrinsic motivation to learn. **Direct verbal**: in which intensive oral drill in verbal and logical patterns was chosen as the mode for instruction since disadvantaged children were considered adequate in perceptual and motoric skills but inadequate in verbal and abstract skills. The class unit was divided into 3 groups of 5 children, initially on the basis of Stanford‐Binet IQ scores and teacher evaluation. Each of the 3 teachers conducted a 20‐min learning period (language, arithmetic, or reading) for the 3 groups. Children attended daily sessions of approximately 2 h and 15 min for a period of no less than 7 or more than 8 months. **Control**: not reported
Outcomes	**Eligible outcomes** *Primary outcome* School readiness, measured with: ∘ Metropolitan Readiness Test (Bixler, 1958) *Secondary outcomes:* Academic achievement, measured with: ∘ Illinois Test of Psycholinguistic Abilities (ITPA; McCarthy, 1961) ∘ Peabody Picture Vocabulary Test (PPVT; Dunn, 1965) Cognitive development, measured with: ∘ Marianne Frostig Developmental Test of Visual Perception (Frostig, 1964) ∘ Stanford‐Binet Intelligence Scale (Terman, 1960) **Ineligible outcomes**: none **Timing of outcome measurement**: baseline; post test (end of preschool); and follow‐up (end of kindergarten year)
Notes	**Study start date**: 1964 **Study end date**: 1967 **Funding source**: Office of Education, US Department of Health, Education, and Welfare **Conflicts of interest**: none stated **Comment(s)**: data were not reported fully and we were unable to make contact with the author who is now deceased

Miller 1969

**Table:** 

Methods	**Design**: cluster‐randomised controlled trial
Participants	**Location/setting**: Kentucky, USA; Head Start centres **Sample size**: 248 **Mean age**: 50.48 months **Sex**: 46.8% boys **Race/ethnicity**: 89.9% Black; 9.7% White; 0.4% Other
Interventions	**Intervention (*n* = 214)**: different curricula within Head Start settings, 14 classes were involved, 4 in each tradition (Bereiter‐Engelmann, Demonstration and Research Centre for Early Education (DARCEE), Traditional) and 2 Montessori classes. Dose and duration are not reported. **Bereiter‐Engelmann (*n* = 64)**: skills‐training programme, which emphasises the acquisition of the tools of academic progress (i.e., the ability to handle linguistic and numerical symbols) **DARCEE programme (*n* = 64)**: emphasises the development of skills and incorporates explicit attempts to develop attitudes related to learning. Children are taught in groups, tasks are carefully analysed and sequenced. Techniques include extrinsic reinforcement, manipulation of materials, practice, and a very heavy emphasis on language. It also includes intensive work with mothers. **Montessori programme (*n* = 33)**: characterised by a high degree of structure in respect to the analysis and sequencing of tasks, combined with great flexibility in that each child is expected to pursue his own interests. The teacher's role is to provide opportunities for interaction with materials which are appropriate for the child's level of achievement, consistent with their interests and not beyond their capacity. The programme emphasises sensory stimulation, manipulation, and intrinsic motivation as techniques in learning. Language, competition, and reinforcement are de‐emphasised. **Traditional Head Start (*n* = 53)**: emphasises enrichment of experience; individual differences; and a climate of freedom & learning by doing **Control (*n* = 34 at baseline)**: children not attending kindergarten
Outcomes	**Eligible outcomes** *Secondary outcomes* Academic achievement, measured with: ∘ Preschool Inventory (PSI; Caldwell, 1965) ∘ California Achievement Test (Tiegs, 1957) ∘ Wepman Auditory Discrimination Test (academic achievement – phonics) (Wepman, 1960). The tests were not administered to all children, only ‘middle class children were tested at the end of their kindergarten year with the following tests’ (p. 1). ∘ Children's Auditory Discrimination Inventory (Stern, 1969). The tests were not administered to all children, only ‘middle class children were tested at the end of their kindergarten year with the following tests’ (p. 1). Cognitive development, measured with: ∘ Stanford‐Binet Intelligence Test (Terman, 1960) ∘ WISC‐R (Wechsler, 2003) ∘ Basic Concept Inventory (Engelmann, 1967) The tests were not administered to all children, only ‘middle class children were tested at the end of their kindergarten year with the following tests’ (p. 1). ∘ Cincinnati Autonomy Battery (Banta, 1970) (4 of the 7 tests of this assessment were used: The Curiosity Box; The Replacement Puzzle; The Dog and Bone Test; Embedded Figures Test). A sub‐sample of *n* = 84 were assessed on the following outcomes. Academic achievement, measured with: ∘ Peabody Picture Vocabulary Test (PPVT; Dunn, 1965) ∘ arithmetic ∘ Expressive Vocabulary Test (Williams, 2007) Emotional well‐being & social competence, measured with: ∘ Behaviour Inventory **Ineligible outcomes**: none **Timing of outcome measurement**: baseline; T1 (after 1st 8 weeks of pre‐kindergarten); post test (end of pre‐kindergarten); follow‐up 1 (end of kindergarten); follow‐up 2 (end of 1st grade); follow‐up 2 (end of 2nd grade); follow‐up 3 (end of 8th grade)
Notes	**Study start date**: 1968 **Study end date**: 1978 **Funding source**: Office of Economic Opportunity and Department of Health, Education and Welfare—Public Health Service **Conflicts of interest**: none stated **Comment(s)**: data were not reported fully and we were unable to make contact with the author (Table 1)

Stukat 1971

**Table:** 

Methods	**Design**: cluster‐randomised controlled trial
Participants	**Location/setting**: Göteborg (Sweden); nursery school setting **Sample size**: 1000 **Mean age**: not reported; all 6 years old **Sex**: not reported **Race/ethnicity**: not reported
Interventions	**Intervention (*n* = 500)**: curriculum focused on 10 different domains to develop competencies Respect and understand the worth of the individual: understand own worth, and the worth of others Respect and understand material values: able to take care of own belongings; able to look after others' belongings; can look after common belongings (e.g., the environment) Respect and understand the rules and standards for human coexistence: able to reach democratic decisions; possess the capacity to behave helpfully; possess the capacity to co‐operate Respect and understand emotional reactions: understand own and others' expressions of feeling; undesirable expressions of feeling; develop desirable expressions of feeling Able to convey a message: able to pass on the content of a message; to have the correct pronunciation; increase vocabulary; ale to follow correct grammatical usage Able to receive an interpret a message: able to comprehend the content of a message; obtain an increased auditory memory; able to listen critically Proficient in preparatory reading and writing skills: able to analyse a text; obtain an increased visual memory; improve eye‐hand co‐ordination; understand the difference between left and right; carry out linguistic sound analyses Able to identify, name and describe: identify, name and describe qualities; able to relate observations to previous experiences; able to identify, name and describe numbers; able to identify, quantify and to describe the primary groups of society; able to identify, name and describe social institutions Able to classify: by characteristics, number, series Able to observe changes: change before and after **Control (*n* = 500)**: nursery school without the experimental programme
Outcomes	**Eligible outcomes** *Primary outcome* School readiness, measured with: ∘ Test of Readiness for School Attendance (Malmqvist, no reference cited) *Secondary outcomes* Academic achievement, measured with: ∘ vocabulary test (newly constructed) Cognitive development, measured with: ∘ general knowledge test (newly constructed) Emotional well‐being & social competence, measured with: ∘ attitude test (newly constructed) ∘ observations of standardised play situations **Ineligible outcomes** Hearing, measured by: ∘ Sound Analysis Test (Jerger, 1966) Teacher attitudes, measured by: ∘ attitudes of the experimental teachers to the programme **Timing of outcome measurement**: baseline (1969) and post test (1970, 1972)
Notes	**Study start date**: 1969 **Study end date**: 1972 **Funding source**: not reported **Conflicts of interest**: none stated **Comment(s)**: none

**IQ**: intelligence quotient; ***n***: number; **SD**: standard deviation; **US**: United States

Characteristics of ongoing studies [ordered by study ID]

Howard 2019

**Table:** 

Study name	HighScope Preschool Curriculum and Professional Development Study
Methods	**Design**: randomised controlled trial
Participants	**Location/setting**: USA; low‐income preschool settings **Sample size**: 1600 children, 400 preschool teachers (lead and assistant) and 100 programme preschool centre administrators **Mean age**: not reported **Sex**: not reported **Race/ethnicity**: the sample will include primarily White and African‐American children
Interventions	**Intervention (*n* = 73)**: HighScope Preschool Curriculum has five core principles (1) active learning, (2) adult‐child interactions, (3) appropriate learning environment, (4) consistent daily routines, and (5) child assessment. Teachers receive training to support children's learning in eight content areas: (1) approaches to learning; (2) social/emotional development; (3) physical development/health; (4) language, literacy, communication; (5) mathematics; (6) creative arts; (7) science and technology; and (8) social studies. The intervention includes 20 days of training for teachers and teaching assistants in Year 1 and two coaching sessions for lead teachers in Year 2. **Control (*n* = 63)**: TAU; most of the centres will continue to use the Creative Curriculum
Outcomes	**Eligible outcomes** *Primary outcomes* School readiness, measured with: ∘ South Carolina Readiness Assessment (SC Education Oversight Committee, 2020) *Secondary outcomes* Academic achievement, measured with: ∘ Woodcock Johnson Applied Problems, Letter‐Word Identification, and Spelling sub scales (McGrew, 2001) ∘ Peabody Picture Vocabulary Test‐Fourth Edition (PPVT; Dunn, 2007) ∘ Teacher Report of School Accomplishments Emotional well‐being and social competence, measured with: ∘ Pencil Tapping Task (Blair, 2002; Diamond, 1996) and the Balance Beam Task (Murray, 2002) ∘ Preschool Learning Behaviours Scale (McDermott, 2000) **Ineligible outcomes (not detailed in protocol)** Classroom quality, measured with: ∘ Classroom Assessment Scoring System (La Paro, 2004) School quality, measured by: ∘ state school report card ratings Fidelity of implementation measures, measured with: ∘ Programme Quality Assessment observation tool (High/Scope, 2003) **Timing of outcome measurement**: baseline and short‐term (post test)
Starting date	2015
Contact information	**Contact Name**: Eboni C Howard **Address**: American Institutes for Research, 1000 Thomas Jefferson Street NW, Washington (DC) 20007‐3835 **Telephone**: not reported **Email**: not reported
Notes	**Funding**: This research was supported by the Institute of Education Sciences, US Department of Education, through Grant R305A150049 awarded to AIR

Yousafzai 2021

**Table:** 

Study name	Yousafzai 2021
Methods	**Design**: stepped‐wedge cluster randomised trial
Participants	**Location/setting**: rural Sindh, Pakistan; village settings **Sample size**: 33 clusters per step (total of 99 clusters); 1089 child‐caregiver dyads **Mean age**: 3.5–5.5 years **Sex**: not reported **Race/ethnicity**: Pakistani
Interventions	**Intervention (*n* =)**: Youth Leaders for Early Childhood Assuring Children are Prepared for School (LEAPS) is a youth‐led ECCE programme that trains female youth, aged 18–24 years, as Community Youth Leaders (CYLs) to deliver high‐quality ECCE for children, 3.5–5.5 years, in rural Sindh, Pakistan.
Outcomes	**Eligible outcomes** *Primary outcomes* School readiness, measured with: ∘ International Development & Early Learning Assessment (IDELA; Save the Children, 2015) *Secondary outcomes* Emotional well‐being & social competence, measured with: ∘ Corsi Block‐Tapping Test (Noël, 2009) ∘ Knock & Tap assessment (Molfese, 2010) ∘ Peg Tap assessment (Molfese, 2010) ∘ Dimensional Change Card Sort (Carlson, 2005; Frye, 1995) ∘ Head Toes Knees Shoulders task (Ponitz, 2009) **Ineligible outcomes (not detailed in protocol)** Youth Executive functioning skills, measured with: ∘ Backward Word Span Task (Davis, 1995) ∘ Corsi Block‐Tapping Test (Kessels, 2000) ∘ Number Stroop Effect (Stroop, 1935) ∘ Trail Making Task (Lezak, 1995; Reitan, 1993) Youth personal & professional development, measured by: ∘ self‐reported questionnaire Youth self‐reported depressive symptoms, measured with: ∘ Self‐Reporting Questionnaire‐20‐item (SRQ‐20; Beusenberg, 1994) Classroom structural & process quality, measured with: ∘ an adaptation of the Measure of Early Learning Environments (MELQO; UNICEF, 2017) ∘ Instrument for Measuring Quality of Early Childhood Education in Colombia (IMCEIC; Ponguta, 2019)
Starting date	January 2019
Contact information	**Contact Name**: Aisha K. Yousafzai **Address**: 665 Huntington Ave., Boston, MA 02115 **Telephone**: +1‐857‐318‐8691 **Email**: ayousafzai@hsph.harvard.edu
Notes	**Funding**: provided by Dubai Cares and Saving Brains Programme Grand Challenges Canada (award number TTS‐1808‐17846)

Abbreviations: ECCE, Early Childhood Care and Education; *n*, number; TAU, treatment as usual; US, United States.

## Supporting information

Supporting information.

Supporting information.
