# Supplementary material for: Centre‐based early education interventions for improving school readiness: A systematic review
Source: Campbell Syst Rev. 2023 Dec 13;19(4):e1363. doi: 10.1002/cl2.1363 (PMC10718474; doi:10.1002/cl2.1363)
Supplement: Supplementary file 1 — Supporting information. [file CL2-19-e1363-s001.docx]

Appendices

## Appendix 1. Search strategies 2014

### Cochrane Central Register of Controlled Trials (CENTRAL)

#1(school near/3 read*)
#2(early next education):ti,ab
#3#1 or #2
#4MeSH descriptor: [Early Intervention (Education)] this term only
#5#3 or #4

### MEDLINE Ovid

1. "Early Intervention (Education)"/
2. (early intervention adj10 education$).tw.
3. (learn$ adj3 (prepar$ or ready or readiness$)).tw.
4. (academic$ adj3 (prepar$ or ready or readiness$)).tw.
5. ((preschool$ or pre-school$) adj3 (prepar$ or ready or readiness$)).tw.
6. (school$ adj3 (prepar$ or ready or readiness$)).tw.
7. (early adj3 education$).tw.
8. (literac$ adj3 (achieve$ or acquisition or develop$ or learn$ or promot$ or skill$)).tw.
9. (literac$ adj3 (prepar$ or ready or readiness$)).tw.
10. (reading adj3 (achieve$ or acquisition or develop$ or learn$ or promot$ or skill$)).tw.
11. (reading adj3 (prepar$ or ready or readiness$)).tw.
12. (language$ adj3 (achieve$ or acquisition or develop$ or learn$ or promot$ or skill$)).tw.
13. (language adj3 (prepar$ or ready or readiness$)).tw.
14. (social adj3 (develop$ or competenc$ or learn$)).tw.
15. (emotion$ adj3 (competenc$ or develop$ or learn$)).tw.
16. performance skill$.tw.
17. (personal adj3 develop$).tw.
18. (health adj3 physical develop$).tw.
19. (math$ adj3 (achieve$ or acquisition or develop$ or learn$ or promot$ or skill$)).tw.
20. (math$ adj3 (prepar$ or ready or readiness$)).tw.
21. (learn$ adj3 approach$).tw.
22. (cogni$ adj3 (acquisition or develop$ or promot$ or skill$)).tw.
23. (communica$ adj3 (acquisition or develop$ or promot$ or skill$)).tw.
24. (knowl$ adj3 (acquisition or develop$ or promot$ or skill$)).tw.
25. "Head Start".tw.
26. "California$ Healthy Start".tw.
27. "SAIL".tw.
28. "Sure Start".tw.
29. "Incredible Years".tw.
30. "Project STAR".tw.
31. "Parents Learning Actively with Youngsters".tw.
32. "Foundations for Success".tw.
33. "Family Check up".tw.
34. "Healthy Children Ready to Learn".tw.
35. (home adj3 school$).tw.
36. (transition adj3 school$).tw.
37. or/1-36
38. Schools/
39. Schools, Nursery/
40. child day care centres/
41. ((early adj2 education$) or early years or ECCE).tw.
42. (creche$ or nurser$ or kindergarten$ or kinder-garten$ or preschool$ or pre-primary or preprimary or playgroup$ or play-group$ or pre-school$ or (child$ adj3 centre$) or (child$ adj3 center$)).tw.
43. settings based.tw.
44. ((school$ or classroom) adj2 (based or setting$)).tw.
45. elementary school$.tw.
46. child care/
47. (child-care or child care or childcare).tw.
48. 46 or 47
49. (centre$ or center$ or facilit$ or "out of home" or polic$ or program$ or scheme$ or setting$).tw.
50. 48 and 49
51. Day Care/
52. (daycare$ or day-care$ or daycentre$ or daycenter$ or (centre-based adj3 care$) or (center-based adj3 care$) or (day$ adj3 (centre$ or center$))).tw.
53. or/51-52
54. exp Infant/
55. exp child/
56. (infant$ or baby or babies or toddler$ or child$ or boy$ or girl$ or pre-kindergarten$ or prekindergarten$ or preschool$ or pre-school$).tw.
57. or/54-56
58. 53 and 57
59. 38 or 39 or 40 or 41 or 42 or 43 or 44 or 45 or 50 or 58
60. 37 and 59
61. randomized controlled trial.pt.
62. controlled clinical trial.pt.
63. randomi#ed.ab.
64. placebo$.ab.
65. drug therapy.fs.
66. randomly.ab.
67. trial.ab.
68. groups.ab.
69. or/61-68
70. exp animals/ not humans.sh.
71. 69 not 70
72. 60 and 71

### Embase OVID

1. early childhood intervention/
2. school readiness.mp.
3. (learn$ adj3 (prepar$ or ready or readiness$)).tw.
4. (reading$ adj3 (prepar$ or ready or readiness$)).tw.
5. ((school$ or preschool$ or pre-school4) adj3 (prepar$ or ready or readiness$)).tw.
6. (academic$ adj3 (prepar$ or ready or readiness$)).tw.
7. (literac$ adj3 (prepar$ or ready or readiness$)).tw.
8. (literac$ adj3 (achieve$ or acquisition or develop$ or learn$ or promot$ or skill$)).tw.
9. (reading$ adj3 (achieve$ or acquisition or develop$ or learn$ or promot$ or skill$)).tw.
10. (social adj3 (develop$ or competenc$ or learn$)).tw.
11. (emotion$ adj3 (develop$ or competenc$ or learn$)).tw.
12. (personal adj3 develop$).tw.
13. (health adj3 physical develop$).tw.
14. (math$ adj3 (develop$ or competenc$ or learn$)).tw.
15. (math$ adj3 (prepar$ or ready or readiness$)).tw.
16. (math$ adj3 (achieve$ or acquisition or develop$ or promot$ or skill$)).tw.
17. (cogni$ adj3 (acquisition or develop$ or promot$ or skills$)).tw.
18. (knowl$ adj3 (acquisition or develop$ or promot$ or skills$)).tw.
19. "Head Start".tw.
20. "California$ Healthy Start".tw.
21. "Sure Start".tw.
22. "Incredible Years".tw.
23. "Project Star".tw.
24. "Foundations for Success".tw.
25. "Family Check Up".tw.
26. "healthy children ready to learn".tw.
27. or/1-26
28. exp Clinical trial/
29. Randomized controlled trial/
30. Randomization/
31. Single blind procedure/
32. Double blind procedure/
33. triple blind procedure/
34. crossover procedure/
35. placebo/
36. randomi#ed.tw.
37. RCT.tw.
38. (random$ adj3 (allocat$ or assign$)).tw.
39. randomly.ab.
40. trial.ab.
41. ((singl$ or doubl$ or trebl$ or tripl$) adj3 (blind$ or mask$)).tw.
42. placebo$.tw.
43. prospective study/
44. (crossover or cross-over).tw.
45. prospective.tw.
46. or/28-45
47. 27 and 46
48. kindergarten/ or nursery school/ or primary school/
49. nursery/
50. settings based.tw.
51. ((school$ or classroom) adj2 (based or setting$)).tw.
52. ((early adj2 education) or early years or ECCE).tw.
53. (creche$ or nurser$ or kindergarten$ or kinder-garten$ or preschool$ or pre-primary or preprimary or playgroup$ or play-group$ or pre-school$ or (child$ adj3 centre$) or (child$ adj3 center$)).tw.
54. elementary school$.tw.
55. 48 or 49 or 50 or 51 or 52 or 53 or 54
56. day care/
57. (daycare$ or day-care$ or daycentre$ or daycenter$ or (centre-based adj3 care$) or (center-based adj3 care$) or (day$ adj3 (centre$ or center$))).tw.
58. 56 or 57
59. (infant$ or baby or babies or toddler$ or child$ or boy$ or girl$ or pre-kindergarten$ or prekindergarten$ or preschool$ or pre-school$).tw.
60. child/
61. infant/
62. 59 or 60 or 61
63. 58 and 62
64. 55 or 63
65. exp animals/ not humans.sh.
66. 46 not 65
67. 47 and 65
68. 47 and 62 and 65

### PsycINFO Ovid

1. exp School Readiness/
2. (school$ adj3 (prepar$ or ready or readiness$)).tw.
3. (academic$ adj3 (prepar$ or ready or readiness$)).tw.
4. ((preschool$ or pre-school$) adj3 (prepar$ or ready or readiness$)).tw.
5. (early adj3 education$).tw.
6. (literac$ adj3 (achieve$ or acquisition or develop$ or learn$ or promot$ or skill$)).tw.
7. (literac$ adj3 (prepar$ or ready or readiness$)).tw.
8. (reading adj3 (achieve$ or acquisition or develop$ or learn$ or promot$ or skill$)).tw.
9. (reading adj3 (prepar$ or ready or readiness$)).tw.
10. (language$ adj3 (achieve$ or acquisition or develop$ or learn$ or promot$ or skill$)).tw.
11. (language adj3 (prepar$ or ready or readiness$)).tw.
12. (social adj3 (develop$ or competenc$ or learn$)).tw.
13. (emotion$ adj3 (competenc$ or develop$ or learn$)).tw.
14. performance skills.tw.
15. (personal adj3 develop$).tw.
16. (health adj3 physical develop$).tw.
17. (math$ adj3 (achieve$ or acquisition or develop$ or learn$ or promot$ or skill$)).tw.
18. (math$ adj3 (prepar$ or ready or readiness$)).tw.
19. (learn$ adj3 approach$).tw.
20. (cogni$ adj3 (acquisition or develop$ or promot$ or skill$)).tw.
21. (communica$ adj3 (acquisition or develop$ or promot$ or skill$)).tw.
22. (knowl$ adj3 (acquisition or develop$ or promot$ or skill$)).tw.
23. project head start/
24. "Head Start".tw.
25. "California$ Healthy Start".tw.
26. "SAIL".tw.
27. "Sure Start".tw.
28. "Incredible Years".tw.
29. "Project STAR".tw.
30. "Parents Learning Actively with Youngsters".tw.
31. "Foundations for Success".tw.
32. "Family Check Up".tw.
33. "Healthy Children Ready to Learn".tw.
34. (home adj3 school$).tw.
35. (transition adj3 school$).tw.
36. or/1-35
37. clinical trials/
38. (randomis$ or randomiz$).tw.
39. (random$ adj3 (allocat$ or assign$)).tw.
40. ((clinic$ or control$) adj trial$).tw.
41. ((singl$ or doubl$ or treb$ or tripl$) adj3 (blind$ or mask$)).tw.
42. (crossover$ or "cross over$").tw.
43. random sampling/
44. Experiment Controls/
45. Placebo/
46. placebo$.tw.
47. exp program evaluation/
48. treatment effectiveness evaluation/
49. ((effectiveness or evaluat$) adj3 (stud$ or research$)).tw.
50. or/37-49
51. 36 and 50
52. ("160" or "180").ag.
53. (child$ or schoolage$ or boy$ or girl$ or kindergart$ or pre-kindergart$ or prekindergart$ or preschool$ or pre-school$).tw.
54. ("160" or "180" or (child$ or schoolage$ or boy$ or girl$ or kindergart$ or pre-kindergart$ or prekindergart$ or preschool$ or pre-school$)).ag.
55. (51 and ("160" or "180" or (child$ or schoolage$ or boy$ or girl$ or kindergart$ or pre-kindergart$ or prekindergart$ or preschool$ or pre-school$))).ag.

### Sociological Abstracts ProQuest

Unavailable because of technical issues accessing this file

### ERIC ProQuest 2014

To be provided by authors

(((SU.EXACT("School Readiness") OR SU.EXACT("Learning Readiness") OR SU.EXACT("Reading Readiness") OR SU.EXACT("Writing Readiness")) OR (TI((school OR reading OR writing OR learn*) NEAR/3 (ready OR readiness OR prepared*)) OR AB((school OR reading OR writing OR learn*) NEAR/3 (ready OR readiness OR prepared*))) OR (("Head Start" OR "California* Healthy Start" OR "SAIL" OR "Sure Start" OR "Incredible Years" OR "Project Star" OR "Parents Learning Actively with Youngsters" OR "Foundations for Success" OR "Family Check Up" OR "Healthy Children Ready to Learn") AND (TI(readiness OR preparedness) OR AB(readiness OR preparedness) OR AB(readiness OR preparedness)))) AND ((SU.EXACT("Longitudinal Studies") OR SU.EXACT("Control Groups") OR SU.EXACT("Program Effectiveness") OR SU.EXACT("Program Effectiveness") OR SU.EXACT("Experimental Groups") OR SU.EXACT("Followup Studies") OR SU.EXACT("Comparative Analysis")) OR (TI(random[*7] OR intervention[*1] OR experiment[*2] OR trial[*1]) OR AB (random[*7] OR intervention[*1] OR experiment[*2] OR trial[*1])))) AND (SU.EXACT("Toddlers") OR SU.EXACT("Young Children") OR SU.EXACT("Early Experience") OR SU.EXACT("Infants") OR SU.EXACT("Early Childhood Education") OR SU.EXACT("Preschool Children") OR TI(baby OR babies OR infant* OR preschool* OR pre-school* OR kindergarten* OR child*) OR AB(baby OR babies OR infant* OR preschool* OR pre-school* OR kindergarten* OR child*))

### British Education Index ProQuest 2014

((SU.EXACT("School Readiness") OR SU.EXACT("Learning Readiness") OR SU.EXACT("Reading Readiness") OR SU.EXACT("Early Reading") OR SU.EXACT("Beginning Reading") OR SU.EXACT("Writing Readiness") OR SU.EXACT(CHILD CARE CENTERS) OR SU.EXACT("Interpersonal Competence") OR SU.EXACT("Mathematics Skills") OR SU.EXACT("Mathematics Achievement") OR SU.EXACT("Mathematics Activities") OR SU.EXACT("Physical Development") OR SU.EXACT("Communication Skills") OR ((school OR read* OR write* OR learn*) NEAR/3 (ready OR readiness OR prepared*))) OR ((("Head Start" OR "California* Healthy Start" OR "SAIL" OR "Sure Start" OR "Incredible Years" OR "Project Star" OR "Parents Learning Actively with Youngsters" OR "Foundations for Success" OR "Family Check Up" OR "Healthy Children Ready to Learn") AND (SU.EXACT("Readiness")) OR TI(readiness OR preparedness) OR AB(readiness OR preparedness)))) AND (SU.EXACT("Longitudinal Studies") OR SU.EXACT("Control Groups") OR SU.EXACT("Program Effectiveness") OR SU.EXACT("Outcomes of Education") OR SU.EXACT("Experimental Groups") OR SU.EXACT("Followup Studies") OR SU.EXACT("Comparative Analysis") OR random[*7] OR intervention[*1] OR experiment[*2] OR trial[*1]) AND (SU.EXACT("Toddlers") OR SU.EXACT("Young Children") OR SU.EXACT("Early Experience") OR SU.EXACT("Infants") OR SU.EXACT("Early Childhood Education") OR SU.EXACT("Preschool Children") OR (baby or babies or infant* OR preschool* OR preschool* OR kindergarten* OR child*))

### Australian Education Index ProQuest 2014

Unavailable because of technical issues accessing this file

### Social Sciences Citation Index and Conference Proceedings Citation Index - Social Science & Humanities Web of Science

Unavailable because of technical issues accessing this file

### Cochrane Database of Systematic Reviews

Unavailable because of technical issues accessing this file

### Database of Abstracts of Reviews of Effects (DARE), in the Cochrane Library

#1 (school readiness):ti,ab
#2 (early next education):ti,ab
#3 #1 or #2
#4 MeSH descriptor: [Early Intervention (Education)] this term only
#5 #3 or #4

### Campbell Collaboration Library

Unavailable because of technical issues accessing this file

### EPPI-Centre Database of Education Research

School readiness

### WorldCat

Limited to dissertations and theses

Unavailable because of technical issues accessing this file

### Networked Digital Library of Theses and Dissertations (NDLTD)

Unavailable because of technical issues accessing this file

### Digitala Vetenskapliga Arkivet (DIVA )

Unavailable because of technical issues accessing this file

### Trove Theses

Unavailable because of technical issues accessing this file

### Theses Canada

Unavailable because of technical issues accessing this file

### National Academic Research and Collaborations Information System (NARCIS)

Unavailable because of technical issues accessing this file

### ProQuest Open Access Dissertations & Theses

Unavailable because of technical issues accessing this file

### DART-Europe E-theses Portal

Unavailable because of technical issues accessing this file

### *meta*Register of Controlled Trials

school readiness

## Appendix 2. Search strategies from 2020 onwards

### Cochrane Central Register of Controlled Trials (CENTRAL)

#1 MeSH descriptor: [Early Intervention, Educational] this term only
#2 (prepare* or readiness or ready)
#3 #1 and #2
#4 (school* NEAR/3 (prepar* or ready or readiness*))
#5 ((kindergar* or nurser* or preschool* or pre-school* or prekindergar* or pre-kindergar* or pre-k or preprimary or pre-primary) NEAR/3 (prepar* or ready or readiness*))
#6 #3 or #4 or #5
#7 ((academic* or education* or language or literac* or math* or reading) NEAR/3 (prepar* or ready or readiness* ))
#8 (cognit* NEAR/3 (acquisition or develop* or skill*)) NEAR/5 ( prepare* or readiness or ready or preschool* or pre-school* or "PRE K" or PRE-K or early next years or early near/3 education)
#9 (communicat* NEAR/3 (acquisition or develop* or skill*)) NEAR/5 ( prepare* or readiness or ready or preschool* or pre-school* or "PRE K" or PRE-K or early next years or early near/3 education)
#10 (emotion* NEAR/3 (ready or readiness or prepared* or develop* or competenc* or learn*)) NEAR/5 ( prepare* or readiness or ready or preschool* or pre-school* or "PRE K" or PRE-K or early next years or early near/3 education)
#11 (knowledg* NEAR/3 develop*) NEAR/5 ( prepare* or readiness or ready or preschool* or pre-school* or "PRE K" or PRE-K or early next years or early near/3 education)
#12 (language NEAR/3 (acquisition or develop* or skill*)) NEAR/5 ( prepare* or readiness or ready or preschool* or pre-school* or "PRE K" or PRE-K or early next years or early near/3 education)
#13 (learn* next approach* or "approach* to learning") NEAR/5 ( prepare* or readiness or ready orpreschool* or pre-school* or "PRE K" or PRE-K or early next years or early near/3 education)
#14 (literacy NEAR/1 (acquisition or develop* or skill*)) NEAR/5 ( prepare* or readiness or ready or preschool* or pre-school* or "PRE K" or PRE-K or early next years or early near/3 education)
#15 (motor NEXT develop* or motor NEXT skill*) NEAR/5 ( prepare* or readiness or ready or preschool* or pre-school* or "PRE K" or PRE-K or early next years or early near/3 education)
#16 (academic* NEAR/1 (achievement or attainment or performance)) NEAR/5 ( prepare* or readiness or ready or preschool* or pre-school* or "PRE K" or PRE-K or early next years or early near/3 education)
#17 (education* NEAR/1 (achievement or attainment or performance)) NEAR/5 ( prepare* or readiness or ready or preschool* or pre-school* or "PRE K" or PRE-K or early next years or early near/3 education)
#18 (school NEAR/1 (achievement or attainment or performance)) NEAR/5 ( prepare* or readiness or ready or preschool* or pre-school* or "PRE K" or PRE-K or early next years or early near/3 education)
#19 (personal NEAR/1 development ) NEAR/5 ( prepare* or readiness or ready or preschool* or pre-school* or "PRE K" or PRE-K or early next years or early near/3 education)
#20 ("Physical well being" or "physical wellbeing" or "physical development" ) NEAR/5 ( prepare* or readiness or ready or preschool* or pre-school* or "PRE K" or PRE-K or "early years" or early near/3 education)
#21 (social* NEAR/1 (develop* or competenc* or learn*)) NEAR/5 ( prepare* or readiness or ready or preschool* or pre-school* or "PRE K" or PRE-K or early next years or early near/3 education)
#22 {or #6-#21} in Trials
#23 "Institute of Developmental Studies" or "Learning to Learn Program" or "Open Court Reading" or "Perry Preschool" or "Chicago School Readiness Project" or "Ready Steady Leap" or "Project Construct" or "Early Childhood Express" or "Literacy Express" or "Language Focussed Curriculum" or "Language Focused Curriculum" or "Early Literacy and Learning Model " or "Letter People" or "Doors to Discovery" or "Curiosity Corner" or "Ladders to Literacy" or "Creative Curriculum" or "Bright Beginnings" or ("Head Start" AND (PREPARE* OR READY OR READINESS)) in Trials
#24 #22 or #23

### MEDLINE Ovid

1 "Early Intervention (Education)"/
2 (prepare$ or readiness or ready).tw,kf.
3 1 and 2
4 (school$ adj3 (prepar$ or ready or readiness$)).tw,kf.
5 ((kindergar$ or nurser$ or preschool$ or pre-school$ or prekindergar$ or pre-kindergar$ or pre-k or preprimary or pre-primary) adj3 (prepar$ or ready or readiness$)).tw,kf. (155)
6 or/3-5
7 (academic$ adj5 (prepar$ or ready or readiness$)).tw,kf.
8 (education$ adj5 (prepar$ or ready or readiness$)).tw,kf.
9 (language adj5 (prepar$ or ready or readiness$)).tw,kf.
10 (literac$ adj5 (prepar$ or ready or readiness$)).tw,kf.
11 (math$ adj5 (prepar$ or ready or readiness$)).tw,kf.
12 (reading$ adj5 (prepar$ or ready or readiness$)).tw,kf.
13 (cognit$ adj3 (acquisition or develop$ or skill$)).tw,kf.
14 (communicat$ adj3 (acquisition or develop$ or skill$)).tw,kf.
15 (emotion$ adj3 (develop$ or competenc$ or learn$)).tw,kf.
16 (knowledge develop$ or develop$ knowledge).tw,kf.
17 (language$ adj3 (acquisition or develop$ or skill$)).tw,kf.
18 (learn$ approach$ or approach$ to learning).tw,kf.
19 (literacy adj3 (acquisition or develop$ or skill$)).tw,kf.
20 (motor adj3 (develop$ or skill$)).tw,kf.
21 (academic adj3 (achievement or attainment or performance)).tw,kf.
22 (education$ adj3 (achievement or attainment or performance)).tw,kf.
23 (school adj3 (achievement or attainment or performance)).tw,kf.
24 personal develop$.tw,kf.
25 (Physical well-being or physical wellbeing or physical development).tw,kf.
26 (social$ adj3 (develop$ or competenc$ or learn$)).tw,kf.
27 or/7-26
28 Schools/
29 Schools, Nursery/
30 child day care centres/
31 ((early adj2 education$) or early years or ECCE).tw,kf.
32 (creche$ or elementary school$ or nurser$ or kindergarten$ or kinder-garten$ or pre-k or preschool$ or pre-school$ or preprimary or pre-primary or playgroup$ or play-group$ or (child$ adj1 centre$) or (child$ adj1 center$)).tw,kf.
33 settings based.tw,kf.
34 ((school$ or classroom) adj2 (based or setting$)).tw,kf.
35 or/28-34
36 Day Care/
37 (daycare$ or day-care$ or daycentre$ or daycenter$ or (centre-based adj3 care$) or (center-based adj3 care$) or (day$ adj3 (centre$ or center$))).tw,kf.
38 or/36-37
39 exp Infant/
40 exp child/
41 (child$ or nurser$ or schoolage$ or kindergart$ or pre-kindergart$ or prekindergart$ or pre-k or preschool$ or pre-school$).tw,kf.
42 or/39-41
43 38 and 42
44 35 or 43
45 27 and 44
46 randomized controlled trial.pt.
47 controlled clinical trial.pt.
48 randomi#ed.ab.
49 placebo$.ab.
50 drug therapy.fs.
51 randomly.ab.
52 trial.ab.
53 groups.ab.
54 or/46-53
55 exp animals/ not humans.sh.
56 54 not 55
57 (Head Start and (ready or readiness or prepare$)).mp.
58 Bright Beginnings.mp.
59 Creative Curriculum.mp.
60 Ladders to Literacy.mp.
61 Curiosity Corner.mp.
62 Doors to Discovery.mp.
63 "Letter People".mp.
64 "Early Literacy and Learning Model ".mp.
65 Language Focus?ed Curriculum.mp.
66 Literacy Express.mp.
67 Early Childhood Express.mp.
68 Project Construct.mp.
69 Ready Steady Leap.mp.
70 Chicago School Readiness Project.mp.
71 Perry Preschool.mp.
72 Open Court Reading.mp.
73 "Learning to Learn Program".mp.
74 Institute of Developmental Studies.mp.
75 or/57-74
76 6 and 56
77 45 and 56
78 75 and 56
79 76 or 77 or 78

### Embase OVID

1 early childhood intervention/
2 (prepare$ or readiness or ready).tw,kw.
3 1 and 2
4 (school$ adj3 (prepar$ or ready or readiness$)).tw,kw.
5 ((kindergar$ or nurser$ or preschool$ or pre-school$ or prekindergar$ or pre-kindergar$ or pre-k or preprimary or pre-primary) adj3 (prepar$ or ready or readiness$)).tw,kw.
6 3 or 4 or 5
7 (academic$ adj5 (prepar$ or ready or readiness$)).tw,kw.
8 (education$ adj5 (prepar$ or ready or readiness$)).tw,kw.
9 (language adj5 (prepar$ or ready or readiness$)).tw,kw.
10 (literac$ adj5 (prepar$ or ready or readiness$)).tw,kw.
11 (math$ adj5 (prepar$ or ready or readiness$)).tw,kw.
12 (reading$ adj5 (prepar$ or ready or readiness$)).tw,kw.
13 (cognit$ adj3 (acquisition or develop$ or skill$)).tw,kw.
14 (communicat$ adj3 (acquisition or develop$ or skill$)).tw,kw.
15 (emotion$ adj3 (develop$ or competenc$ or learn$)).tw,kw.
16 (knowledge develop$ or develop$ knowledge).tw,kw.
17 (language$ adj3 (acquisition or develop$ or skill$)).tw,kw.
18 (learn$ approach$ or approach$ to learning).tw,kw.
19 (literacy adj3 (acquisition or develop$ or skill$)).tw,kw.
20 (motor develop$ or motor skill$).tw,kw.
21 (academic adj3 (achievement or attainment or performance)).tw,kw.
22 (education$ adj3 (achievement or attainment or performance)).tw,kw.
23 (school adj3 (achievement or attainment or performance)).tw,kw.
24 personal develop$.tw,kw.
25 (Physical well-being or physical wellbeing or physical development).tw,kw.
26 (social$ adj3 (develop$ or competenc$ or learn$)).tw,kw.
27 or/7-26
28 preschool child/
29 ((early adj2 education$) or early years or ECCE).tw,kw.
30 (creche$ or nurser$ or kindergarten$ or kinder-garten$ or pre-kindergart$ or prekindergart$ or pre-k or preschool$ or pre-school$ or preprimary or pre-primary or playgroup$ or play-group$ or (child$ adj1 centre$) or (child$ adj1 center$)).tw,kw.
31 or/28-30
32 27 and 31
33 6 or 32
34 (Head Start and (ready or readiness or prepare$)).mp.
35 Bright Beginnings.mp.
36 Creative Curriculum.mp.
37 Ladders to Literacy.mp.
38 Curiosity Corner.mp.
39 Doors to Discovery.mp.
40 "Letter People".mp.
41 "Early Literacy and Learning Model".mp.
42 Language Focus?ed Curriculum.mp.
43 Literacy Express.mp.
44 Early Childhood Express.mp.
45 Project Construct.mp.
46 Ready Steady Leap.mp.
47 Chicago School Readiness Project.mp.
48 Perry Preschool.mp.
49 Open Court Reading.mp.
50 "Learning to Learn Program".mp.
51 Institute of Developmental Studies.mp.
52 or/34-51
53 33 or 52
54 Randomized controlled trial/
55 controlled clinical trial/
56 Single blind procedure/
57 Double blind procedure/
58 triple blind procedure/
59 Crossover procedure/
60 (crossover or cross-over).tw.
61 ((singl$ or doubl$ or tripl$ or trebl$) adj1 (blind$ or mask$)).tw.
62 Placebo/
63 placebo.tw.
64 prospective.tw.
65 factorial$.tw.
66 random$.tw.
67 assign$.ab.
68 allocat$.tw.
69 volunteer$.ab.
70 or/54-69
71 53 and 70

### APA PsycINFO

1 School Readiness/
2 (school$ adj3 (prepar$ or ready or readiness$)).tw.
3 ((kindergar$ or nurser$ or preschool$ or pre-school$ or prekindergar$ or pre-kindergar$ or pre-k or preprimary or pre-primary) adj3 (prepar$ or ready or readiness$)).tw.
4 or/1-3
5 (academic$ adj5 (prepar$ or ready or readiness$)).tw.
6 (education$ adj5 (prepar$ or ready or readiness$)).tw.
7 (language adj5 (prepar$ or ready or readiness$)).tw.
8 (literac$ adj5 (prepar$ or ready or readiness$)).tw.
9 (math$ adj5 (prepar$ or ready or readiness$)).tw.
10 (reading$ adj5 (prepar$ or ready or readiness$)).tw.
11 (cognit$ adj3 (acquisition or develop$ or skill$)).tw.
12 (communicat$ adj3 (acquisition or develop$ or skill$)).tw.
13 (emotion$ adj3 (develop$ or competenc$ or learn$)).tw.
14 (knowledg$ adj3 develop$).tw.
15 (language$ adj3 (acquisition or develop$ or skill$)).tw.
16 (learn$ approach$ or approach$ to learning).tw.
17 (literacy adj3 (acquisition or develop$ or skill$)).tw.
18 (motor skill$ or motor develop$).tw.
19 (performance skills or education$ performance$ or academic performance).tw.
20 personal develop$.tw.
21 (Physical well-being or physical wellbeing or physical development).tw.
22 (social$ adj3 (develop$ or competenc$ or learn$)).tw.
23 or/5-22
24 Schools/
25 Elementary Schools/
26 Nursery Schools/
27 preschool students/
28 preschool education/
29 child day care/
30 ((early adj2 education$) or early years or ECCE).tw.
31 (creche$ or nurser$ or kindergarten$ or kinder-garten$ or preschool$ or pre-primary or preprimary or playgroup$ or play-group$ or pre-school$ or (child$ adj1 centre$) or (child$ adj1 center$)).tw.
32 setting$ based.tw.
33 ((school$ or classroom) adj2 (based or setting$)).tw.
34 or/24-33)
35 day care centres/
36 (daycare$ or day-care$ or daycentre$ or daycenter$ or (centre-based adj3 care$) or (center-based adj3 care$) or (day$ adj3 (centre$ or center$))).tw.
37 or/35-36
38 limit 37 to (100 childhood <birth to age 12 yrs> or 140 infancy <2 to 23 mo> or 160 preschool age <age 2 to 5 yrs> or 180 school age <age 6 to 12 yrs>)
39 (child$ or nurser$ or schoolage$ or kindergart$ or pre-kindergart$ or prekindergart$ or pre-k or preschool$ or pre-school$).tw.
40 or/38-39
41 37 and 40
42 34 or 41
43 23 and 42
44 4 or 43
45 ("Institute of Developmental Studies" or "Learning to Learn Program" or "Open Court Reading" or "Perry Preschool" or "Chicago School Readiness Project" or "Ready Steady Leap" or "Project Construct" or "Early Childhood Express" or "Literacy Express" or "Language Focussed Curriculum" or "Language Focused Curriculum" or "Early Literacy and Learning Model " or "Letter People" or "Doors to Discovery" or "Curiosity Corner" or "Ladders to Literacy" or "Creative Curriculum" or "Bright Beginnings" or ("Head Start" and (PREPARE$ or READY or READINESS))).mp.
46 44 or 45
47 randomized controlled trials/
48 clinical trials/
49 treatment effectiveness evaluation/
50 exp treatment outcomes/
51 followup studies/
52 longitudinal studies/
53 Placebo/
54 Experiment Controls/
55 exp program evaluation/
56 (randomis$ or randomiz$).tw.
57 (control$ adj (experiment$ or trial$)).tw.
58 (TAU or "treatment as usual" or "wait$ list").ab.
59 (random$ adj3 (allocat$ or assign$)).ab.
60 or/47-59
61 46 and 60

### ERIC EBSCOhost

S41 S17 OR S40
S40 S16 AND S39
S39 S18 OR S19 OR S20 OR S21 OR S22 OR S23 OR S24 OR S25 OR S26 OR S27 OR S28 OR S29 OR S30 OR S31 OR S32 OR S33 OR S34 OR S35 OR S36 OR S37 OR S38
S38 TX "Institute of Developmental Studies"
S37 TX "Learning to Learn Program"
S36 TX "Open Court Reading"
S35 TX "Perry Preschool"
S34 TX "Chicago School Readiness Project"
S33 TX "Chicago School Readiness Project"
S32 TX "Ready Steady Leap"
S31 TX "Project Construct"
S30 TX "Early Childhood Express"
S29 TX "Literacy Express"
S28 TX "Language Focussed Curriculum"
S27 TX "Language Focused Curriculum"
S26 TX "Language Focus*ed Curriculum"
S25 TX "Early Literacy and Learning Model "
S24 TX "Letter People"
S23 TX Doors to Discovery
S22 TX "Curiosity Corner"
S21 TX "Ladders to Literacy"
S20 TX "Creative Curriculum"
S19 TX Bright Beginnings
S18 TX "Head Start" AND (PREPARE* OR READY OR READINESS)
S17 S13 AND S16
S16 S14 OR S15
S15 TI (random* or trial* or experiment* or PROSPECTIVE* OR longitudinal or control*) OR AB (random* or trial* or experiment* or PROSPECTIVE* OR longitudinal or CONTROL*)
S14 DE "Randomized Controlled Trials" OR DE "Meta Analysis" OR DE "Evaluation Research" OR DE "Control Groups" OR DE "Experimental Groups" OR DE "Longitudinal Studies" OR DE "Followup Studies" OR DE "Program Effectiveness" OR DE "Program Evaluation"
S13 S8 OR S12
S12 S7 AND S11
S11 S9 OR S10
S10 DE "Preschool Curriculum"
S9 (DE "Preschool Education" OR DE "Preschool Learning (1966 1980)" OR DE "Preschool Children")
S8 TI(("early years" or "early education" or school or kindergarten or "kinder-garten" or "PRE K" or "PRE-K" OR read* OR write* OR learn*) N3 (ready OR readiness OR prepared*))
S7 S1 OR S2 OR S3 OR S4 OR S5 OR S6
S6 (DE "Communication Skills") OR (DE "Interpersonal Competence")
S5 DE "Physical Development" OR DE "Motor Development"
S4 DE "Mathematics Skills" OR DE "Mathematics Activities" OR DE "Mathematics Achievement"
S3 DE "Beginning Reading"
S2 DE "Reading Centers"
S1 (DE "Writing Readiness" OR DE "Learning Readiness" OR DE "School Readiness" OR DE "Reading

### British Education Index EBSCOhost

S38 S14 OR S37
S37 S13 AND S36
S36 S15 OR S16 OR S17 OR S18 OR S19 OR S20 OR S21 OR S22 OR S23 OR S24 OR S25 OR S26 OR S27 OR S28 OR S29 OR S30 OR S31 OR S32 OR S33 OR S34 OR S35
S35 TX "Institute of Developmental Studies"
S34 TX "Learning to Learn Program"
S33 TX "Open Court Reading"
S32 TX "Perry Preschool"
S31 TX "Chicago School Readiness Project"
S30 TX "Chicago School Readiness Project"
S29 TX "Ready Steady Leap"
S28 TX "Project Construct"
S27 TX "Early Childhood Express"
S26 TX "Literacy Express"
S25 TX "Language Focussed Curriculum"
S24 TX "Language Focused Curriculum"
S23 TX "Language Focus*ed Curriculum"
S22 TX "Early Literacy and Learning Model"
S21 TX "Letter People"
S20 TX Doors to Discovery
S19 TX "Curiosity Corner"
S18 TX "Ladders to Literacy"
S17 TX "Creative Curriculum"
S16 TX Bright Beginnings
S15 TX "Head Start"
S14 S10 AND S13
S13 S11 OR S12
S12 TI (random* or trial* or experiment* or PROSPECTIVE* OR longitudinal or BLIND* or CONTROL*) OR AB (random* or trial* or experiment* or PROSPECTIVE* OR longitudinal or BLIND* or CONTROL*)
S11 (DE "EVALUATION" OR DE "CURRICULUM evaluation" OR DE "EVALUATION research") OR (DE "PROGRAM effectiveness (Education)" OR DE "PROGRAM validation (Education)" OR DE "PRESCHOOL education -- Evaluation" OR DE "EDUCATIONAL evaluation")
S10 S8 OR S9
S9 TI((early N2 education* or early years or ECCE) N3 (curriculum or program* or experiment* or model or project* or prepar* or ready or readiness))
S8 S6 OR S7
S7 TI((kindergar* or nurser* or preschool* or pre-school* or prekindergar* or pre-kindergar* or pre-k or school* ) N3 ( prepar* or ready or readiness)) OR AB((kindergar* or nurser* or preschool* or pre-school* or prekindergar* or pre-kindergar* or pre-k or school* ) N3 (prepar* or ready or readiness))
S6 S3 OR S5
S5 S1 AND S4
S4 TI(curriculum or program* or experiment* or model* or project* )
S3 S1 AND S2
S2 (DE "PRESCHOOL education" OR DE "PRESCHOOL children") OR (DE "ELEMENTARY schools" OR DE "KINDERGARTEN")
S1 (DE "MATHEMATICAL readiness" OR DE "ARITHMETIC readiness" OR DE "PREPAREDNESS" OR DE "LEARNING readiness" OR DE "DEVELOPMENTAL tasks" OR DE "READING readiness" OR DE "WRITING readiness")

### Social Sciences Citation Index and Conference Proceedings Citation Index - Social Science & Humanities Web of Science, Clarivate

# 7 #6 AND #5
Indexes=SSCI, CPCI-SSH Timespan=All years
# 6 Ts=(random* or control or group )
Indexes=SSCI, CPCI-SSH Timespan=All years
# 5 #4 OR #3 OR #2 OR #1
Indexes=SSCI, CPCI-SSH Timespan=All years
# 4 TS=((school* or kindergar* or preschool* or "pre-school*" or prekindergar* or "pre-kindergar*" or "pre-k" ) near/1 (prepar* or ready or readiness*))
Indexes=SSCI, CPCI-SSH Timespan=All years
# 3 TS= (((cognit* or communication* or emotion* or language or literacy or reading or writing or math* ) near/1 (acquisition or develop* or promot* or skill*)) near/3 (kindergar* or nurser* or preschool* or "pre-school*" or prekindergar* or "pre-kindergar*" or "pre-k" or preprimary or "pre-primary"))
Indexes=SSCI, CPCI-SSH Timespan=All years

### Cochrane Database of Systematic Reviews

#1 MeSH descriptor: [Early Intervention, Educational] this term only
#2 (prepare* or readiness or ready):TI
#3 #1 and #2
#4 (school* NEAR/3 (prepar* or ready or readiness*)):TI
#5 ((kindergar* or nurser* or preschool* or pre-school* or prekindergar* or pre-kindergar* or pre-k or preprimary or pre-primary) NEAR/3 (prepar* or ready or readiness*)):TI
#6 #3 or #4 or #5 in Cochrane Reviews, Cochrane Protocols
#7 "Institute of Developmental Studies" or "Learning to Learn Program" or "Open Court Reading" or "Perry Preschool" or "Chicago School Readiness Project" or "Ready Steady Leap" or "Project Construct" or "Early Childhood Express" or "Literacy Express" or "Language Focussed Curriculum" or "Language Focused Curriculum" or "Early Literacy and Learning Model " or "Letter People" or "Doors to Discovery" or "Curiosity Corner" or "Ladders to Literacy" or "Creative Curriculum" or "Bright Beginnings" or ("Head Start" AND (PREPARE* OR READY OR READINESS)):TI,AB in Cochrane Reviews, Cochrane Protocols
#8 #6 OR #7

### EPISTEMONIKOS

(title:((school OR literacy OR numeracy OR math*) AND (ready OR readiness OR prepare*)) OR abstract:((school OR literacy OR numeracy OR math*) AND (ready OR readiness OR prepare*)))

### Campbell Collaboration Library

Searched using the key terms "school readiness" "preparedness" " preschool"

### EPPI-Centre Evidence Library

Browsed index to Knowledge Library for keywords ( school readiness; literacy)

### ProQuest Dissertations & Theses Global

ti(SCHOOL READINESS) AND noft(RANDOMLY OR RANDOMISED OR RANDOMIZED) AND ti(PRESCHOOL* OR PRE-SCHOOL* OR KINDERGARTEN* OR PRE-KINDERGARTEN* OR "DAY CARE")

### ClinicalTrials.gov

Basic search school readiness | Child

### WHO International Clinical Trials Registry Platform

Home search page : school readiness

Automatic synonyms included in the search : School; Academic achievement; Boarding school; College (environment); Day Care Centers for Children; Details of education; Educational environment; Educational Learning Centers; Educational process of instructing; Educational Status; Elementary Schools; general concepts related to organizations; higher education institution; Knowledge acquisition; Primary Schools; Schools, Nursery; technical or vocational school; Universities; workshop or seminar || readiness; Assessment of readiness to learn; Bereitschaftspotential; Caregiver behavior: home care readiness; Independent living discharge readiness; Learning readiness enhancement; Readiness finding; Readiness for discharge; Readiness for transport; Readiness to learn; Readiness to undergo procedure; Ready for enhanced immunization status; Ready for enhanced parenting; Ready for enhanced religiosity; Ready for enhanced self-concept; Supported living discharge readiness

### ISRCTN registry

school readiness

## Appendix 3. Searching other resources

**Keywords**

School readiness
Head Start
Ypsilanti Project
Bright Beginnings
Chicago Readiness Project
Creative Curriculum
High/Scope
Montessori
PATHS
PCER
Preschool
Randomisation
Randomised controlled trial
social skills
literacy
numeracy
well-being
emotional
language
reading
phonics
math*
RCT
Tools of the Mind

## Appendix 4. Unused methods

| **Section** | **Proposed methods** | **Reason for non-use** |
| --- | --- | --- |
| **Measures of treatment effect** | **Dichotomous data**  Where dichotomous data were presented, we stated that we would calculate an odds ratio with a 95% confidence interval (CI) for each outcome in each trial ( [Higgins 2021](#REF-Higgins-2021), Chapter 15.4.1). For meta-analyses of dichotomous outcomes included in the ’Summary of findings’ table, we stated we would express the results as absolute risks, using high and low observed risks amongst the control groups as our reference point. However, all our data were continuous measures. | None of the studies reported dichotomous data for the outcomes of interest. |
| **Unit of analysis issues** | **Cluster-randomised trials**  We had planned to seek direct estimates of the effect from analysis that accounted for the cluster design. | Although we did find some cluster adjusted designs, the effect size data were not usable as there were additional covariates in the model. |
| **Dealing with missing data** | **Meta-regression**  We had planned to assess the sensitivity of any primary meta-analyses to missing data using meta-regression to test for any effect of missingness on the summary estimates. | We did not have enough missing data to conduct meta-regression. |
| **Assessment of reporting biases** | As a direct test for publication bias, we proposed conducting sensitivity analyses to compare the results from published data with unpublished data. | We did not find unpublished data to compare with published data. |
| **Data synthesis** | **Funnel plot asymmetry**  In the event of severe funnel plot asymmetry, we would have presented the results of both fixed-effect and random-effects analyses, under the assumption that asymmetry would have suggested that neither model was appropriate. If both had indicated a presence (or absence) of effect, we would have reported this. | We inspected the funnel plots for the 14 meta-analyses with 10 or more studies. There was no evidence of severe funnel plot asymmetry for any of the 14 plots. |
| **Subgroup analysis & investigation of heterogeneity** | **Subgroup analysis**  We had pre-planned subgroup analysis for intervention intensity and duration, socioeconomic status and English language learners. Additional covariates were considered including mean child age, parental curriculum component, and second language learners. | In eight of the analyses that contained significant heterogeneity, the number of studies was too few to consider further exploration via meta regression or sub analyses (i.e. below 10; [Higgins 2021](#REF-Higgins-2021), Chapter 12). For the other three analyses with significant heterogeneity, while the number of studies did exceed 10, there was insufficient distribution of the covariates values across studies ([Higgins 2021](#REF-Higgins-2021), Chapter 12) to consider further exploration. Additional covariates were considered, including mean child age, parental curriculum component, and second language learners; however, the number of studies meeting these criteria was less than four and therefore subgroup analyses were not possible. |

## Appendix 5. Assessment of risk of bias judgement criteria

### Sequence generation

The method used to generate the allocation sequence was described, in detail, to assess whether it should have produced comparable groups is described. We made a judgement on the sequence generation process as follows (see Higgins 2011):

- ‘low’ when participants and researchers were unaware of participants’ future allocation to treatment condition until after decisions about eligibility were made and informed consent was obtained;
- ‘unclear’ when allocation concealment was not clearly stated or unknown; or
- ‘high’ when allocation was not concealed from either participants before informed consent or from researchers before decisions about inclusion were made, or allocation was not used.

### Allocation concealment

The method used to conceal the allocation sequence was described in sufficient detail to assess whether intervention schedules could have been identified in advance of, or during, recruitment. We made a judgement on whether the allocation was adequately concealed as follows:

- ‘low’ when participants and researchers were unaware of participants’ allocation to treatment;
- ‘unclear’ when allocation concealment was not clearly stated or unknown; or
- ‘high’ when allocation was not concealed from participants or allocation was not used.

### Blinding of participants, personnel and outcome assessors

The measures used to blind participants, personnel and outcome assessors were described to assess knowledge of any group as to which intervention a given participant might have received. We made a judgement on whether knowledge of the allocated intervention was adequately prevented during the study.

##### Blinding of participants and personnel

The risk of bias was judged as follows:

- ‘low’ when blinding of participants and personnel was ensured;
- ‘unclear’ where there was not adequate information provided in the study report or blinding of participants or personnel was not assessed; or
- ‘high’ when blinding of participants or personnel was not assured.

##### Blinding of outcome assessors

The risk of bias was judged as follows:

- ‘low’ when blinding of outcome assessment was ensured;
- ‘unclear’ where there was not adequate information provided in the study report or blinding of outcome assessment was not assessed; or
- ‘high’ when blinding of outcome assessment was not assured.

### Incomplete outcome data

We extracted and report data on attrition and exclusions, as well the numbers involved (compared with those randomised), reasons for attrition/exclusion (where reported or obtained from investigators), and any re-inclusions performed by review authors following the retrieval of missing data. We made a judgement on whether incomplete outcome data had been adequately addressed. We judged the risk of bias as follows:

- ‘low’ when the number of participants randomised to groups is clear and data for all participants who completed the trial were included in the analyses;
- ‘unclear’ when information about which participants completed the study could not be acquired by contacting the researchers of the study; or
- ‘high’ when there was clear evidence that there was attrition or exclusion from analysis in at least one participant group.

### Selective outcome reporting

We determined the likelihood that the authors of the trial omitted some of the collected data when presenting the results by comparing methods and results outcomes in identified studies. We judged the risk of bias as follows:

- ‘low’ when all collected data seemed to be reported;
- ‘unclear’ when it was not clear whether other data were collected and not reported; or
- ‘high’ when the data from some measures used in the trial were not reported.

### Other sources of bias

Any important concerns about bias present in the trial that were not addressed by the other domains of the tool, such as such as stopping the trial early, changing methods during the trial or other anomalies, were described and assessed (i.e. we made a judgement on whether the study was free of other problems that could put it at a high risk of bias). We judged the risk of bias as follows:

- ‘low’ when allocation was by community, institution or school, and it was unlikely that the control group received the intervention;
- ‘unclear’ when professionals were allocated within a clinic or school, and it is possible that the communication between intervention and control professional could have occurred; or
- ‘high’ when it was likely that the control group received part of the intervention.
