# Supplementary material for: Centre‐based early education interventions for improving school readiness: A systematic review
Source: Campbell Syst Rev. 2023 Dec 13;19(4):e1363. doi: 10.1002/cl2.1363 (PMC10718474; doi:10.1002/cl2.1363)
Supplement: Supplementary file 2 — Supporting information. [file CL2-19-e1363-s002.pdf]

Analysis 1.1

Study or Subgroup	Mean Difference	SE	No treatment Total	Centre-based intervention Total	Mean Difference IV, Fixed, 95% CI	Mean Difference IV, Fixed, 95% CI	A	B	C	D	E	F	G	H
**1.1.1 General (long term)**														
Weikart 1967 (1)	16.1	13.433	49	56	16.10 [-10.23 , 42.43]		+	+	−	−	?	+	+	+
**1.1.2 Language (short-term): ICC 0.12**														
Weikart 1967 (1)	0.12	0.1747	55	62	0.12 [-0.22 , 0.46]		+	+	−	−	?	+	+	+
**1.1.3 Language (long-term): ICC 0.12**														
Weikart 1967 (1)	0.23	0.283	54	56	0.23 [-0.32 , 0.78]		+	+	−	−	?	+	+	+

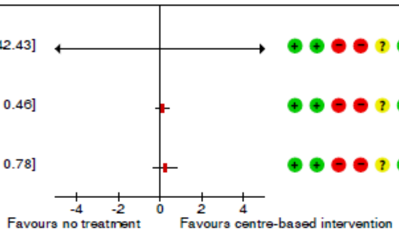

Footnotes

(1) Data have been adjusted for clustering using an ICC of 0.12

Risk of bias legend

(A) Random sequence generation (selection bias)
(B) Timing and recruitment of clusters
(C) Allocation concealment (selection bias)
(D) Blinding of participants and personnel (performance bias)
(E) Blinding of outcome assessment (detection bias)
(F) Incomplete outcome data (attrition bias)
(G) Selective reporting (reporting bias)
(H) Other bias

Comparison 1: Centre-based early education interventions for improving school readiness versus no treatment, Outcome 1: Academic achievement (MDs, FE)

Analysis 1.2

Study or Subgroup	Mean Difference	SE	No treatment Total	Centre-based intervention Total	Weight	Mean Difference IV, Random, 95% CI	A	B	C	D	E	F	G	H
**1.2.1 Mathmatics (long term): ICC 0.12**														
Deutsch 1971	0.230717	0.13651	127	94	91.6%	0.23 [-0.04 , 0.50]	?		?	?	?	?	+	?
Weikart 1967 (1)	0.37703671	0.44969767	49	56	8.4%	0.38 [-0.50 , 1.26]	+	+	−	−	?	+	+	+
**Subtotal (95% CI)**			**176**	**150**	**100.0%**	**0.24 [-0.01 , 0.50]**								
Heterogeneity: Tau² = 0.00; Chi² = 0.10, df = 1 (P = 0.76); I² = 0%														
Test for overall effect: Z = 1.86 (P = 0.06)														
**1.2.2 Vocabulary (short term): ICC 0.12**														
Deutsch 1971	0.255957	0.094879	145	487	59.9%	0.26 [0.07 , 0.44]	?		?	?	?	?	+	?
Peisner-Feinberg 2019	0.177987	0.12736595	103	458	33.2%	0.18 [-0.07 , 0.43]	+	+	+	?	+	+	+	+
Weikart 1967	0.3330323	0.28068998	64	56	6.8%	0.33 [-0.22 , 0.88]	+	+	−	−	?	+	+	+
**Subtotal (95% CI)**			**312**	**1001**	**100.0%**	**0.24 [0.09 , 0.38]**								
Heterogeneity: Tau² = 0.00; Chi² = 0.37, df = 2 (P = 0.83); I² = 0%														
Test for overall effect: Z = 3.20 (P = 0.001)														
**1.2.3 Vocabulary (medium term): ICC 0.12**														
Deutsch 1971	0.4354	0.0795	375	285	92.5%	0.44 [0.28 , 0.59]	?		?	?	?	?	+	?
Weikart 1967 (1)	0.2504	0.27974587	58	61	7.5%	0.25 [-0.30 , 0.80]	+	+	−	−	?	+	+	+
**Subtotal (95% CI)**			**433**	**346**	**100.0%**	**0.42 [0.27 , 0.57]**								
Heterogeneity: Tau² = 0.00; Chi² = 0.40, df = 1 (P = 0.52); I² = 0%														
Test for overall effect: Z = 5.51 (P < 0.00001)														
**1.2.4 Vocabulary (long term): ICC 0.12**														
Deutsch 1971	0.4566	0.1307	146	102	71.6%	0.46 [0.20 , 0.71]	?		?	?	?	?	+	?
Weikart 1967 (1)	0.079	0.28038932	55	61	28.4%	0.08 [-0.47 , 0.63]	+	+	−	−	?	+	+	+
**Subtotal (95% CI)**			**201**	**163**	**100.0%**	**0.35 [0.02 , 0.68]**								
Heterogeneity: Tau² = 0.02; Chi² = 1.49, df = 1 (P = 0.22); I² = 33%														
Test for overall effect: Z = 2.05 (P = 0.04)														

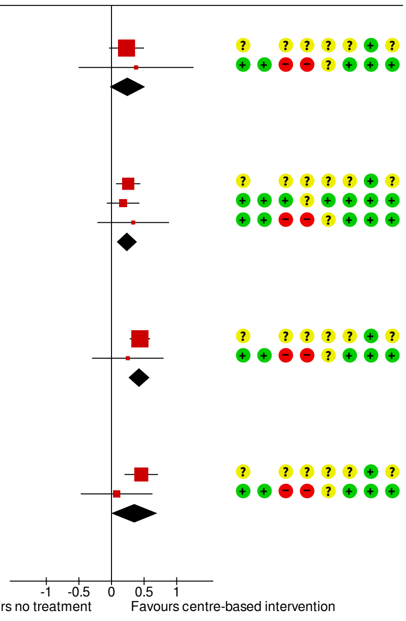

Footnotes
(1) Data have been adjusted for clustering using an ICC of 0.12

Risk of bias legend
(A) Random sequence generation (selection bias)
(B) Timing and recruitment of clusters
(C) Allocation concealment (selection bias)
(D) Blinding of participants and personnel (performance bias)
(E) Blinding of outcome assessment (detection bias)
(F) Incomplete outcome data (attrition bias)
(G) Selective reporting (reporting bias)
(H) Other bias

Comparison 1: Centre-based early education interventions for improving school readiness versus no treatment, Outcome 2: Academic achievement (MDs, RE)

Analysis 1.3

Study or Subgroup	Std. Mean Difference	SE	No treatment Total	Centre-based intervention Total	Weight	Std. Mean Difference IV, Random, 95% CI	A	B	C	D	E	F	G	H
**1.3.1 Reading (long term): ICC 0.12**														
Deutsch 1971	0.0347	0.1308	145	98	82.9%	0.03 [-0.22 , 0.29]	?		?	?	?	?	+	?
Weikart 1967 (1)	0.2816	0.28760101	49	56	17.1%	0.28 [-0.28 , 0.85]	+	+	−	−	?	+	+	+
**Subtotal (95% CI)**			**194**	**154**	**100.0%**	**0.08 [-0.16 , 0.31]**								
Heterogeneity: Tau² = 0.00; Chi² = 0.61, df = 1 (P = 0.43); I² = 0%														
Test for overall effect: Z = 0.65 (P = 0.52)														
**1.3.2 Vocabulary (short term): ICC 0.12**														
Deutsch 1971	0.2562	0.0949	487	145	53.5%	0.26 [0.07 , 0.44]	?		?	?	?	?	+	?
Peisner-Feinberg 2019	0.177987	0.109181	458	103	40.4%	0.18 [-0.04 , 0.39]	+	+	+	?	+	+	+	+
Weikart 1967 (1)	0.333	0.28068998	56	64	6.1%	0.33 [-0.22 , 0.88]	+	+	−	−	?	+	+	+
**Subtotal (95% CI)**			**1001**	**312**	**100.0%**	**0.23 [0.09 , 0.37]**								
Heterogeneity: Tau² = 0.00; Chi² = 0.44, df = 2 (P = 0.80); I² = 0%														
Test for overall effect: Z = 3.30 (P = 0.0010)														

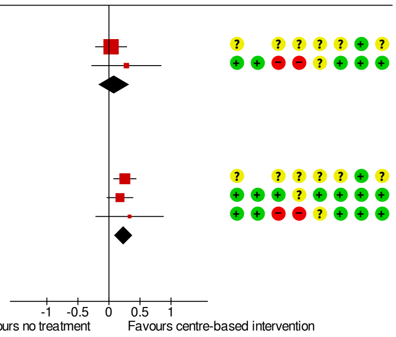

Footnotes
(1) Data have been adjusted for clustering using an ICC of 0.12

Risk of bias legend
(A) Random sequence generation (selection bias)
(B) Timing and recruitment of clusters
(C) Allocation concealment (selection bias)
(D) Blinding of participants and personnel (performance bias)
(E) Blinding of outcome assessment (detection bias)
(F) Incomplete outcome data (attrition bias)
(G) Selective reporting (reporting bias)
(H) Other bias

Comparison 1: Centre-based early education interventions for improving school readiness versus no treatment, Outcome 3: Academic achievement (SMDs)

Analysis 1.4

Study or Subgroup	Mean Difference	SE	No treatment Total	Centre-based intervention Total	Weight	Mean Difference IV, Random, 95% CI	A	B	C	D	E	F	G	H
**1.4.1 Intelligence (short term): ICC 0.12**														
Deutsch 1971	3.575	1.1986	440	142	89.2%	3.58 [1.23 , 5.92]	?		?	?	?	?	+	?
Weikart 1967 (1)	2.6	3.43955142	56	64	10.8%	2.60 [-4.14 , 9.34]	+	+	−	−	?	+	+	+
**Subtotal (95% CI)**			**496**	**206**	**100.0%**	**3.47 [1.25 , 5.69]**								
Heterogeneity: Tau² = 0.00; Chi² = 0.07, df = 1 (P = 0.79); I² = 0%														
Test for overall effect: Z = 3.07 (P = 0.002)														
**1.4.2 Intelligence (medium term): ICC 0.12**														
Deutsch 1971	8.1065	1.1823	275	281	60.2%	8.11 [5.79 , 10.42]	?		?	?	?	?	+	?
Weikart 1967 (1)	1.4	3.24984459	58	61	39.8%	1.40 [-4.97 , 7.77]	+	+	−	−	?	+	+	+
**Subtotal (95% CI)**			**333**	**342**	**100.0%**	**5.44 [-1.00 , 11.87]**								
Heterogeneity: Tau² = 16.51; Chi² = 3.76, df = 1 (P = 0.05); I² = 73%														
Test for overall effect: Z = 1.66 (P = 0.10)														
**1.4.3 Intelligence (long term): ICC 0.12**														
Deutsch 1971	3.844	1.7546	144	100	78.8%	3.84 [0.41 , 7.28]	?		?	?	?	?	+	?
Weikart 1967 (1)	1.2	3.38571212	55	62	21.2%	1.20 [-5.44 , 7.84]	+	+	−	−	?	+	+	+
**Subtotal (95% CI)**			**199**	**162**	**100.0%**	**3.28 [0.23 , 6.34]**								
Heterogeneity: Tau² = 0.00; Chi² = 0.48, df = 1 (P = 0.49); I² = 0%														
Test for overall effect: Z = 2.11 (P = 0.04)														

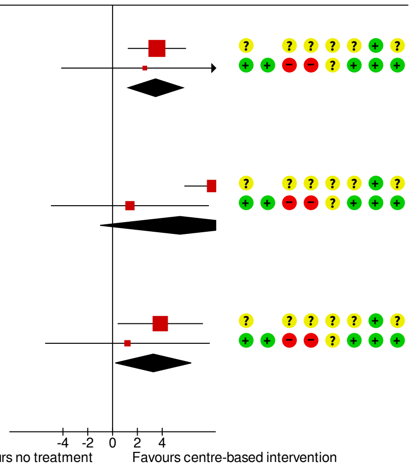

Footnotes

(1) Data have been adjusted for clustering using an ICC of 0.12

Risk of bias legend

(A) Random sequence generation (selection bias)
(B) Timing and recruitment of clusters
(C) Allocation concealment (selection bias)
(D) Blinding of participants and personnel (performance bias)
(E) Blinding of outcome assessment (detection bias)
(F) Incomplete outcome data (attrition bias)
(G) Selective reporting (reporting bias)
(H) Other bias

Comparison 1: Centre-based early education interventions for improving school readiness versus no treatment, Outcome 4: Cognitive development (MDs)

Analysis 1.5

Study or Subgroup	Std. Mean Difference	SE	No treatment Total	Centre-based intervention Total	Weight	Std. Mean Difference IV, Random, 95% CI	A	B	C	D	E	F	G	H
Abbott-Shim 2000	-0.0595	0.2223	35	48	20.8%	-0.06 [-0.50 , 0.38]	+		+	?	?	+	?	−
Peisner-Feinberg 2019 (1)	-0.2799	0.1219	84	348	51.2%	-0.28 [-0.52 , -0.04]	+	+	+	?	+	+	+	+
Weikart 1967 (1)	0.0606	0.1853	62	55	28.0%	0.06 [-0.30 , 0.42]	+	+	−	−	?	+	+	+
**Total (95% CI)**			**181**	**451**	**100.0%**	**-0.14 [-0.35 , 0.08]**								
Heterogeneity: Tau² = 0.01; Chi² = 2.59, df = 2 (P = 0.27); I² = 23%														
Test for overall effect: Z = 1.26 (P = 0.21)														
Test for subgroup differences: Not applicable														

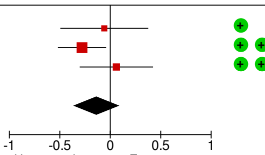

Footnotes

(1) Data have been adjusted for clustering using an ICC of 0.12

Risk of bias legend

(A) Random sequence generation (selection bias)
(B) Timing and recruitment of clusters
(C) Allocation concealment (selection bias)
(D) Blinding of participants and personnel (performance bias)
(E) Blinding of outcome assessment (detection bias)
(F) Incomplete outcome data (attrition bias)
(G) Selective reporting (reporting bias)
(H) Other bias

Comparison 1: Centre-based early education interventions for improving school readiness versus no treatment, Outcome 5: Emotional well-being & social competence: problem behaviours (short term) (SMDs)

Analysis 1.6

Study or Subgroup	Std. Mean Difference	SE	No treatment Total	Centre-based intervention Total	Weight	Std. Mean Difference IV, Random, 95% CI	A	B	C	D	E	F	G	H
Abbott-Shim 2000	-0.5304	0.2262	35	48	31.7%	-0.53 [-0.97 , -0.09]	+		+	?	?	+	?	−
Peisner-Feinberg 2019 (1)	0.149	0.1217	84	348	41.5%	0.15 [-0.09 , 0.39]	+	+	+	?	+	+	+	+
Weikart 1967 (1)	0	0.27989106	55	62	26.9%	0.00 [-0.55 , 0.55]	+	+	−	−	?	+	+	+
**Total (95% CI)**			**174**	**458**	**100.0%**	**-0.11 [-0.54 , 0.33]**								
Heterogeneity: Tau² = 0.10; Chi² = 7.00, df = 2 (P = 0.03); I² = 71%														
Test for overall effect: Z = 0.48 (P = 0.63)														
Test for subgroup differences: Not applicable														

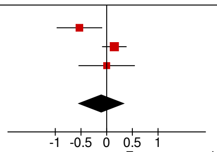

Footnotes

(1) Data have been adjusted for clustering using an ICC of 0.12

Risk of bias legend

(A) Random sequence generation (selection bias)
(B) Timing and recruitment of clusters
(C) Allocation concealment (selection bias)
(D) Blinding of participants and personnel (performance bias)
(E) Blinding of outcome assessment (detection bias)
(F) Incomplete outcome data (attrition bias)
(G) Selective reporting (reporting bias)
(H) Other bias

Comparison 1: Centre-based early education interventions for improving school readiness versus no treatment, Outcome 6: Emotional well-being & social competence: social skills (short term) (SMDs)

Analysis 2.1

Study or Subgroup	Std. Mean Difference	SE	TAU Total	Centre-based intervention Total	Weight	Std. Mean Difference IV, Random, 95% CI	Std. Mean Difference IV, Random, 95% CI	A	B	C	D	E	F	G	H
Warden 1988	2.1224	0.4031	20	20	47.7%	2.12 [1.33 , 2.91]		?		?	?	?	?	−	?
Yousafzai 2018 (1)	0.3004	0.1101	168	166	52.3%	0.30 [0.08 , 0.52]		?	−	?	−	+	+	+	+
**Total (95% CI)**			**188**	**186**	**100.0%**	**1.17 [-0.61 , 2.95]**									

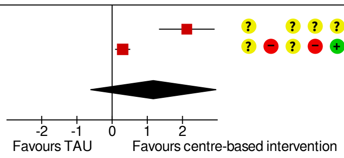

Risk of Bias

Heterogeneity: Tau² = 1.57; Chi² = 19.01, df = 1 (P < 0.0001); I² = 95%
Test for overall effect: Z = 1.29 (P = 0.20)
Test for subgroup differences: Not applicable

Footnotes
(1) Authors adjusted data for clustering using an ICC of 0.14

Risk of bias legend
(A) Random sequence generation (selection bias)
(B) Timing and recruitment of clusters
(C) Allocation concealment (selection bias)
(D) Blinding of participants and personnel (performance bias)
(E) Blinding of outcome assessment (detection bias)
(F) Incomplete outcome data (attrition bias)
(G) Selective reporting (reporting bias)
(H) Other bias

Comparison 2: Centre-based early education interventions for improving school readiness versus treatment as usual, Outcome 1: School readiness (short-term) (SMDs)

Analysis 2.2

Study or Subgroup	Mean Difference	SE	TAU Total	Centre-based intervention Total	Weight	Mean Difference IV, Random, 95% CI	A	B	C	D	E	F	G	H
**2.2.1 Language (medium term): ICC 0.12**														
PCER 2008, Bright Beginnings & Creative Curriculum (1)	0.19075496	0.17418317	196	103	16.6%	0.19 [-0.15 , 0.53]	?	−	?	?	+	+	+	+
PCER 2008, Creative Curriculm (1)	-0.146982	0.22094568	85	76	10.3%	-0.15 [-0.58 , 0.29]	+	−	+	−	+	−	+	+
PCER 2008, Doors to Discovery & Let's Begin (1)	0.04092267	0.16167777	162	72	19.3%	0.04 [-0.28 , 0.36]	+	−	+	?	+	+	+	?
PCER 2008, Language-Focused Curriculum (1)	0.012879	0.23046016	93	94	9.5%	0.01 [-0.44 , 0.46]	+	−	−	?	+	?	+	?
PCER 2008, Literacy Express + DLM + OCR Pre-K (1)	0.36762532	0.19493925	157	79	13.3%	0.37 [-0.01 , 0.75]	+	−	−	?	+	+	+	?
PCER 2008, Project Construct (1)	-0.008239	0.20552669	101	84	12.0%	-0.01 [-0.41 , 0.39]	−	−	−	?	+	+	+	?
PCER 2008, Ready, Set, Leap! (1)	-0.050504	0.16329611	131	116	18.9%	-0.05 [-0.37 , 0.27]	+	−	−	?	+	+	+	?
**Subtotal (95% CI)**			**925**	**624**	**100.0%**	**0.06 [-0.08 , 0.20]**								
Heterogeneity: Tau² = 0.00; Chi² = 4.55, df = 6 (P = 0.60); I² = 0%														
Test for overall effect: Z = 0.90 (P = 0.37)														
**2.2.2 Language (long term): ICC 0.12**														
Blatt 1965	0.02470456	0.2719189	38	21	48.1%	0.02 [-0.51 , 0.56]	?		?	?	+	−	?	−
Van de Riet 1972	0.731862	0.234304	39	39	51.9%	0.73 [0.27 , 1.19]	?		−	?	?	?	?	?
**Subtotal (95% CI)**			**77**	**60**	**100.0%**	**0.39 [-0.30 , 1.08]**								
Heterogeneity: Tau² = 0.19; Chi² = 3.88, df = 1 (P = 0.05); I² = 74%														
Test for overall effect: Z = 1.11 (P = 0.27)														
**2.2.3 Mathematics (medium term): ICC 0.12**														
Blair 2014 (1)	0.105786	0.10881207	393	268	18.2%	0.11 [-0.11 , 0.32]	+	+	−	?	−	?	+	+
Farran 2015 (1)	-0.031682	0.11210274	459	351	17.1%	-0.03 [-0.25 , 0.19]	?	−	?	?	?	?	?	?
Lillard 2017	0.3936	0.1751	67	66	7.0%	0.39 [0.05 , 0.74]	−		?	?	?	+	+	+
PCER 2008, Bright Beginnings & Creative Curriculum (1)	0.23591166	0.17406875	196	104	7.1%	0.24 [-0.11 , 0.58]	?	−	?	?	+	+	+	+
PCER 2008, Creative Curriculm (1)	0.149977	0.22219427	85	73	4.4%	0.15 [-0.29 , 0.59]	+	−	+	−	+	−	+	+
PCER 2008, Doors to Discovery & Let's Begin (1)	-0.00532987	0.09584515	162	72	23.5%	-0.01 [-0.19 , 0.18]	+	−	+	?	+	+	+	?
PCER 2008, Language-Focused Curriculum (1)	0.161989	0.23128549	92	93	4.0%	0.16 [-0.29 , 0.62]	+	−	−	?	+	?	+	?
PCER 2008, Literacy Express + DLM + OCR Pre-K (1)	0.35520064	0.19571242	71	76	5.6%	0.36 [-0.03 , 0.74]	+	−	−	?	+	+	+	?
PCER 2008, Project Construct (1)	0.101013	0.20751373	99	80	5.0%	0.10 [-0.31 , 0.51]	−	−	−	?	+	+	+	?
PCER 2008, Ready, Set, Leap! (1)	0.020163	0.16392552	128	115	8.0%	0.02 [-0.30 , 0.34]	+	−	−	?	+	+	+	?
**Subtotal (95% CI)**			**1752**	**1298**	**100.0%**	**0.10 [0.01 , 0.19]**								
Heterogeneity: Tau² = 0.00; Chi² = 8.07, df = 9 (P = 0.53); I² = 0%														
Test for overall effect: Z = 2.08 (P = 0.04)														
**2.2.4 Phonics (short term) ICC 0.12**														
Bierman 2008 (1)	0.41266	0.1481966	178	157	14.7%	0.41 [0.12 , 0.70]	?	−	?	?	+	?	+	?
Courtier 2021 (1)	0.6224	0.39165	77	98	2.6%	0.62 [-0.15 , 1.39]	?	+	?	?	−	?	+	?
PCER 2008, Bright Beginnings & Creative Curriculum (1)	0.006	0.17505843	191	100	11.3%	0.01 [-0.34 , 0.35]	?	−	?	?	+	+	+	+
PCER 2008, Creative Curriculm (1)	0.0614	0.21772799	90	81	7.8%	0.06 [-0.37 , 0.49]	+	−	+	−	+	−	+	+
PCER 2008, Doors to Discovery & Let's Begin (1)	0.1147	0.10142702	188	89	24.7%	0.11 [-0.08 , 0.31]	+	−	+	?	+	+	+	?
PCER 2008, Language-Focused Curriculum (1)	0.3146	0.23187248	191	95	7.0%	0.31 [-0.14 , 0.77]	+	−	−	?	+	?	+	?
PCER 2008, Literacy Express + DLM + OCR Pre-K (1)	0.0529	0.1880483	113	98	10.0%	0.05 [-0.32 , 0.42]	+	−	−	?	+	+	+	?
PCER 2008, Project Construct (1)	-0.0935	0.20051039	140	122	9.0%	-0.09 [-0.49 , 0.30]	−	−	−	?	+	+	+	?
PCER 2008, Ready, Set, Leap! (1)	-0.0935	0.16091448	140	122	12.9%	-0.09 [-0.41 , 0.22]	+	−	−	?	+	+	+	?
**Subtotal (95% CI)**			**1308**	**962**	**100.0%**	**0.12 [-0.01 , 0.24]**								
Heterogeneity: Tau² = 0.01; Chi² = 9.77, df = 8 (P = 0.28); I² = 18%														
Test for overall effect: Z = 1.81 (P = 0.07)														
**2.2.5 Phonics (medium term) ICC 0.12**														
PCER 2008, Bright Beginnings & Creative Curriculum (1)	0.0199	0.17382565	196	103	16.7%	0.02 [-0.32 , 0.36]	?	−	?	?	+	+	+	+
PCER 2008, Creative Curriculm (1)	0.1017	0.22078587	85	76	10.3%	0.10 [-0.33 , 0.53]	+	−	+	−	+	−	+	+
PCER 2008, Doors to Discovery & Let's Begin (1)	-0.0281	0.1616181	162	72	19.3%	-0.03 [-0.34 , 0.29]	+	−	+	?	+	+	+	?
PCER 2008, Language-Focused Curriculum (1)	0.0607	0.23007918	94	95	9.5%	0.06 [-0.39 , 0.51]	+	−	−	?	+	?	+	?
PCER 2008, Literacy Express + DLM + OCR Pre-K (1)	0.2794	0.19592624	158	76	13.1%	0.28 [-0.10 , 0.66]	+	−	−	?	+	+	+	?
PCER 2008, Project Construct (1)	-0.0322	0.20468589	102	86	12.0%	-0.03 [-0.43 , 0.37]	−	−	−	?	+	+	+	?
PCER 2008, Ready, Set, Leap! (1)	-0.045	0.1629336	132	117	19.0%	-0.04 [-0.36 , 0.27]	+	−	−	?	+	+	+	?
**Subtotal (95% CI)**			**929**	**625**	**100.0%**	**0.04 [-0.10 , 0.18]**								
Heterogeneity: Tau² = 0.00; Chi² = 2.17, df = 6 (P = 0.90); I² = 0%														
Test for overall effect: Z = 0.54 (P = 0.59)														
**2.2.6 Reading (medium) ICC 0.12**														
Bierman 2008 (1)	0	0.14682776	176	157	7.8%	0.00 [-0.29 , 0.29]	?	−	?	?	+	?	+	?
Blair 2014 (1)	0.1006	0.10870446	396	268	14.2%	0.10 [-0.11 , 0.31]	+	+	−	?	−	?	+	+
Farran 2015 (1)	-0.051495	0.12441792	459	351	10.8%	-0.05 [-0.30 , 0.19]	?	−	?	?	?	?	?	?
Lillard 2017	0.548943	0.176761	67	66	5.4%	0.55 [0.20 , 0.90]	−		?	?	?	+	+	+
PCER 2008, Bright Beginnings & Creative Curriculum (1)	-0.0060953	0.10750571	196	103	14.5%	-0.01 [-0.22 , 0.20]	?	−	?	?	+	+	+	+
PCER 2008, Creative Curriculm (1)	0.04626061	0.22067046	85	76	3.4%	0.05 [-0.39 , 0.48]	+	−	+	−	+	−	+	+
PCER 2008, Doors to Discovery & Let's Begin	0.00275912	0.09398137	162	72	19.0%	0.00 [-0.18 , 0.19]	+	−	+	?	+	+	+	?
PCER 2008, Language-Focused Curriculum	0.05213937	0.23049772	94	93	3.2%	0.05 [-0.40 , 0.50]	+	−	−	?	+	?	+	?
PCER 2008, Literacy Express + DLM + OCR Pre-K	0.07897031	0.11914802	154	79	11.8%	0.08 [-0.15 , 0.31]	+	−	−	?	+	+	+	?
PCER 2008, Project Construct	0.02467423	0.20622743	99	83	3.9%	0.02 [-0.38 , 0.43]	−	−	−	?	+	+	+	?
PCER 2008, Ready, Set, Leap!	0.08952074	0.16568166	123	111	6.1%	0.09 [-0.24 , 0.41]	+	−	−	?	+	+	+	?
**Subtotal (95% CI)**			**2011**	**1459**	**100.0%**	**0.06 [-0.02 , 0.14]**								
Heterogeneity: Tau² = 0.00; Chi² = 9.60, df = 10 (P = 0.48); I² = 0%														
Test for overall effect: Z = 1.39 (P = 0.17)														

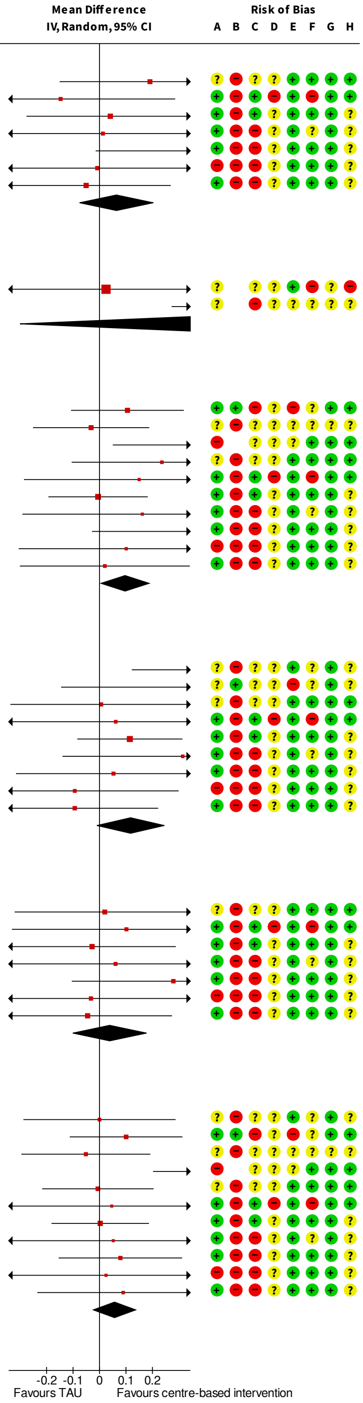

Footnotes
(1) Data have been adjusted for clustering using an ICC of 0.12

Risk of bias legend
(A) Random sequence generation (selection bias)
(B) Timing and recruitment of clusters
(C) Allocation concealment (selection bias)
(D) Blinding of participants and personnel (performance bias)
(E) Blinding of outcome assessment (detection bias)
(F) Incomplete outcome data (attrition bias)
(G) Selective reporting (reporting bias)
(H) Other bias

Comparison 2: Centre-based early education interventions for improving school readiness versus treatment as usual, Outcome 2: Academic achievement (MDs)

Analysis 2.3

Study or Subgroup	Std. Mean Difference	SE	TAU Total	Centre-based intervention Total	Weight	Std. Mean Difference IV, Random, 95% CI
**2.3.1 General (long term): ICC 0.12**						
Blatt 1965	0.4531	0.2753	38	21	46.8%	0.45 [-0.09 , 0.99]
Van de Riet 1972	0.97276	0.241729	38	39	53.2%	0.97 [0.50 , 1.45]
**Subtotal (95% CI)**			**76**	**60**	**100.0%**	**0.73 [0.22 , 1.24]**
Heterogeneity: Tau^2 = 0.07; Chi^2 = 2.01, df = 1 (P = 0.16); I^2 = 50%						
Test for overall effect: Z = 2.81 (P = 0.005)						
**2.3.2 Language (short term): ICC 0.12**						
Bierman 2008 (1)	0.047861	0.109175	179	158	28.7%	0.05 [-0.17 , 0.26]
Blatt 1965	0.3014	0.273423	38	21	4.6%	0.30 [-0.23 , 0.84]
PCER 2008, Bright Beginnings & Creative Curriculum (1)	0.1366	0.21868438	190	100	7.1%	0.14 [-0.29 , 0.57]
PCER 2008, Creative Curriculm (1)	-0.1781	0.21868438	89	80	7.1%	-0.18 [-0.61 , 0.25]
PCER 2008, Doors to Discovery & Let's Begin (1)	0.2284	0.15137333	189	89	14.9%	0.23 [-0.07 , 0.53]
PCER 2008, Language-Focused Curriculum (1)	0.0168	0.23048827	92	95	6.4%	0.02 [-0.43 , 0.47]
PCER 2008, Literacy Express + DLM + OCR Pre-K (1)	0.2327	0.1886133	184	92	9.6%	0.23 [-0.14 , 0.60]
PCER 2008, Project Construct (1)	-0.0209	0.20090437	110	94	8.5%	-0.02 [-0.41 , 0.37]
PCER 2008, Ready, Set, Leap! (1)	-0.1576	0.16183267	139	119	13.0%	-0.16 [-0.47 , 0.16]
**Subtotal (95% CI)**			**1210**	**848**	**100.0%**	**0.06 [-0.05 , 0.17]**
Heterogeneity: Tau^2 = 0.00; Chi^2 = 6.18, df = 8 (P = 0.63); I^2 = 0%						
Test for overall effect: Z = 1.02 (P = 0.31)						
**2.3.3 Mathmatics (short term): ICC 0.12**						
Barnes 2016 (1)	0.1544	0.10741809	347	171	10.7%	0.15 [-0.06 , 0.36]
Blair 2014 (1)	0.1497	0.10715822	410	289	10.8%	0.15 [-0.06 , 0.36]
Courtier 2021 (1)	0.4037	0.15140849	77	98	5.4%	0.40 [0.11 , 0.70]
Farran 2015 (1)	0.064205	0.1122356	465	348	9.8%	0.06 [-0.16 , 0.28]
Hsueh 2014 (1)	0.13	0.14	713	220	6.3%	0.13 [-0.14 , 0.40]
Lillard 2017	0.2015	0.1732	68	66	4.1%	0.20 [-0.14 , 0.54]
Lonigan 2015 (1)	0.3114	0.10915542	602	142	10.4%	0.31 [0.10 , 0.53]
PCER 2008, Bright Beginnings & Creative Curriculum (1)	0.18645159	0.17540559	191	100	4.0%	0.19 [-0.16 , 0.53]
PCER 2008, Creative Curriculm (1)	0.14641796	0.21854168	89	80	2.6%	0.15 [-0.28 , 0.57]
PCER 2008, Doors to Discovery & Let's Begin (1)	0.00937274	0.15097153	189	89	5.4%	0.01 [-0.29 , 0.31]
PCER 2008, Language-Focused Curriculum (1)	0.21695665	0.23137951	92	94	2.3%	0.22 [-0.24 , 0.67]
PCER 2008, Literacy Express + DLM + OCR Pre-K (1)	0.31880454	0.1898605	186	88	3.4%	0.32 [-0.05 , 0.69]
PCER 2008, Project Construct (1)	0.26388311	0.20359939	106	90	3.0%	0.26 [-0.14 , 0.66]
PCER 2008, Ready, Set, Leap! (1)	0.04460665	0.16218705	136	118	4.7%	0.04 [-0.27 , 0.36]
Raver 2009 (1)	0.01986688	0.14581054	238	229	5.8%	0.02 [-0.27 , 0.31]
Warden 1988	0.9491685	0.335392061	20	20	1.1%	0.95 [0.29 , 1.61]
Yousafzai 2018 (2)	0.2872	0.11	166	168	10.2%	0.29 [0.07 , 0.50]
**Subtotal (95% CI)**			**4095**	**2410**	**100.0%**	**0.19 [0.12 , 0.26]**
Heterogeneity: Tau^2 = 0.00; Chi^2 = 15.07, df = 16 (P = 0.52); I^2 = 0%						
Test for overall effect: Z = 5.35 (P < 0.00001)						
**2.3.4 Mathematics (long term) ICC 0.12**						
Farran 2015 (1)	0.0691	0.11299968	443	335	35.7%	0.07 [-0.15 , 0.29]
Lillard 2017	0.3655	0.1851	57	62	33.5%	0.37 [0.00 , 0.73]
Van de Riet 1972	1.370616	0.252913	39	39	30.8%	1.37 [0.87 , 1.87]
**Subtotal (95% CI)**			**539**	**436**	**100.0%**	**0.57 [-0.10 , 1.24]**
Heterogeneity: Tau^2 = 0.31; Chi^2 = 22.22, df = 2 (P < 0.0001); I^2 = 91%						
Test for overall effect: Z = 1.67 (P = 0.10)						
**2.3.5 Reading (short term) ICC 0.12**						
Bierman 2008 (1)	0.1843	0.14672819	179	158	7.2%	0.18 [-0.10 , 0.47]
Blair 2014 (1)	0.0461	0.09892949	413	290	8.9%	0.05 [-0.15 , 0.24]
Courtier 2021 (1)	0.6989	0.15446029	77	98	7.0%	0.70 [0.40 , 1.00]
Farran 2015 (1)	-0.0265	0.11221186	465	348	8.4%	-0.03 [-0.25 , 0.19]
Lillard 2017	-0.155155	0.173061	68	66	6.4%	-0.16 [-0.49 , 0.18]
Lonigan 2015 (1)	0.2707	0.109353965	607	141	8.5%	0.27 [0.06 , 0.49]
PCER 2008, Bright Beginnings & Creative Curriculum (1)	0.16917127	0.17534421	191	100	6.3%	0.17 [-0.17 , 0.51]
PCER 2008, Creative Curriculm (1)	-0.18941694	0.21852812	90	80	5.1%	-0.19 [-0.62 , 0.24]
PCER 2008, Doors to Discovery & Let's Begin (1)	0.04476987	0.15141959	189	88	7.1%	0.04 [-0.25 , 0.34]
PCER 2008, Language-Focused Curriculum (1)	0.21867809	0.23143037	91	95	4.8%	0.22 [-0.23 , 0.67]
PCER 2008, Literacy Express + DLM + OCR Pre-K (1)	0.50811792	0.18968814	187	93	5.9%	0.51 [0.14 , 0.88]
PCER 2008, Project Construct (1)	-0.05818012	0.20141906	111	92	5.6%	-0.06 [-0.45 , 0.34]
PCER 2008, Ready, Set, Leap! (1)	0.07758538	0.1618606	139	118	6.7%	0.08 [-0.24 , 0.39]
Raver 2009 (1)	0.207387	0.14620203	238	229	7.3%	0.21 [-0.08 , 0.49]
Warden 1988	0.94312979	0.23632	40	40	4.7%	0.94 [0.48 , 1.41]
**Subtotal (95% CI)**			**3085**	**2036**	**100.0%**	**0.18 [0.05 , 0.32]**
Heterogeneity: Tau^2 = 0.04; Chi^2 = 39.71, df = 14 (P = 0.0003); I^2 = 65%						
Test for overall effect: Z = 2.69 (P = 0.007)						
**2.3.6 Reading (long term) ICC 0.12**						
Blatt 1965	0.05494863	0.271955028	38	21	23.4%	0.05 [-0.48 , 0.59]
Farran 2015 (1)	0	0.11300592	443	335	27.0%	0.00 [-0.22 , 0.22]
Lillard 2017	0.952138	0.193939	57	62	25.5%	0.95 [0.57 , 1.33]
Van de Riet 1972	1.273421	0.249459	39	39	24.1%	1.27 [0.78 , 1.76]
**Subtotal (95% CI)**			**577**	**457**	**100.0%**	**0.56 [-0.08 , 1.21]**
Heterogeneity: Tau^2 = 0.39; Chi^2 = 34.11, df = 3 (P < 0.00001); I^2 = 91%						
Test for overall effect: Z = 1.71 (P = 0.09)						
**2.3.7 Vocabulary (short term) ICC 0.12**						
Bierman 2008 (1)	0.15395934	0.14685113	178	157	7.0%	0.15 [-0.13 , 0.44]
Blair 2014 (1)	0.1553	0.10652362	417	297	13.3%	0.16 [-0.05 , 0.36]
Blatt 1965	0.33790451	0.273804574	38	21	2.0%	0.34 [-0.20 , 0.87]
Courtier 2021 (1)	0.0105	0.12277984	77	98	10.0%	0.01 [-0.23 , 0.25]
Farran 2015 (1)	-0.0217	0.11223719	465	348	12.0%	-0.02 [-0.24 , 0.20]
Lillard 2017	0.2852	0.1757	67	64	4.9%	0.29 [-0.06 , 0.63]
Lonigan 2015 (1)	0.3518	0.10954919	609	141	12.6%	0.35 [0.14 , 0.57]
PCER 2008, Bright Beginnings & Creative Curriculum (1)	0.22683666	0.17571767	189	99	4.9%	0.23 [-0.12 , 0.57]
PCER 2008, Creative Curriculm (1)	0.0428036	0.21944133	87	78	3.1%	0.04 [-0.39 , 0.47]
PCER 2008, Doors to Discovery & Let's Begin (1)	0.15424329	0.15196256	183	89	6.5%	0.15 [-0.14 , 0.45]
PCER 2008, Language-Focused Curriculum (1)	0.00605458	0.23025066	93	95	2.8%	0.01 [-0.45 , 0.46]
PCER 2008, Literacy Express + DLM + OCR Pre-K (1)	0.43208889	0.1886847	190	94	4.2%	0.43 [0.06 , 0.80]
PCER 2008, Project Construct (1)	0.06741315	0.20055858	113	94	3.8%	0.07 [-0.33 , 0.46]
PCER 2008, Ready, Set, Leap! (1)	0.14348454	0.16136346	139	121	5.8%	0.14 [-0.17 , 0.46]
Raver 2009 (1)	0.12452377	0.1459493	238	229	7.1%	0.12 [-0.16 , 0.41]
**Subtotal (95% CI)**			**3083**	**2025**	**100.0%**	**0.16 [0.08 , 0.23]**
Heterogeneity: Tau^2 = 0.00; Chi^2 = 11.33, df = 14 (P = 0.66); I^2 = 0%						
Test for overall effect: Z = 4.00 (P < 0.0001)						
**2.3.8 Vocabulary (medium term) ICC 0.12**						
Bierman 2008 (1)	0.0899	0.14682776	176	157	10.2%	0.09 [-0.20 , 0.38]
Blair 2014 (1)	0.205438	0.10881488	397	270	18.6%	0.21 [-0.01 , 0.42]
Blatt 1965 (1)	0.07042843	0.271987429	38	21	3.0%	0.07 [-0.46 , 0.60]
Farran 2015 (1)	0.0266	0.11210274	459	351	17.5%	0.03 [-0.19 , 0.25]
Lillard 2017	0.0214	0.1734	67	66	7.3%	0.02 [-0.32 , 0.36]
PCER 2008, Bright Beginnings & Creative Curriculum (1)	0.13780148	0.17401044	196	103	7.3%	0.14 [-0.20 , 0.48]
PCER 2008, Creative Curriculm (1)	0.22550166	0.22159826	84	76	4.5%	0.23 [-0.21 , 0.66]
PCER 2008, Doors to Discovery & Let's Begin (1)	0.12985523	0.16239462	160	71	8.4%	0.13 [-0.19 , 0.45]
PCER 2008, Language-Focused Curriculum (1)	-0.04665082	0.2307593	91	95	4.1%	-0.05 [-0.50 , 0.41]
PCER 2008, Literacy Express + DLM + OCR Pre-K (1)	0.46511628	0.19667268	152	78	5.7%	0.47 [0.08 , 0.85]
PCER 2008, Project Construct (1)	0.21388153	0.206661	96	85	5.2%	0.21 [-0.19 , 0.62]
PCER 2008, Ready, Set, Leap! (1)	-0.01896789	0.16460867	128	112	8.1%	-0.02 [-0.34 , 0.30]
**Subtotal (95% CI)**			**2044**	**1485**	**100.0%**	**0.12 [0.03 , 0.21]**
Heterogeneity: Tau^2 = 0.00; Chi^2 = 6.47, df = 11 (P = 0.84); I^2 = 0%						
Test for overall effect: Z = 2.57 (P = 0.01)						
**2.3.9 Vocabulary (long term) ICC 0.12**						
Blair 2014 (1)	0.171968	0.272403	38	21	11.1%	0.17 [-0.36 , 0.71]
Farran 2015 (1)	0.0749	0.11299968	443	335	64.5%	0.07 [-0.15 , 0.30]
Lillard 2017	0.1928	0.1839	57	62	24.4%	0.19 [-0.17 , 0.55]
**Subtotal (95% CI)**			**538**	**418**	**100.0%**	**0.11 [-0.06 , 0.29]**

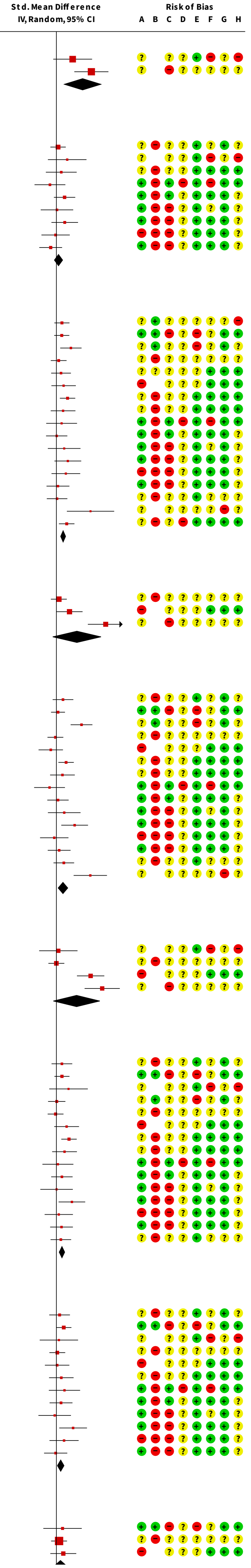

Subtotal (95% CI) [illegible]
Heterogeneity: $Tau^2 = 0.00$; $Chi^2 = 0.35$, df = 2 (P = 0.84); $I^2 = 0\%$
Test for overall effect: Z = 1.26 (P = 0.21)

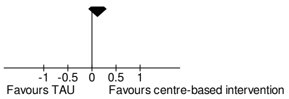

Footnotes
(1) Data have been adjusted for clustering using an ICC of 0.12
(2) Author adjusted data for clustering using an ICC of 0.14

Risk of bias legend
(A) Random sequence generation (selection bias)
(B) Timing and recruitment of clusters
(C) Allocation concealment (selection bias)
(D) Blinding of participants and personnel (performance bias)
(E) Blinding of outcome assessment (detection bias)
(F) Incomplete outcome data (attrition bias)
(G) Selective reporting (reporting bias)
(H) Other bias

Comparison 2: Centre-based early education interventions for improving school readiness versus treatment as usual, Outcome 3: Academic achievement (SMDs)

Analysis 2.4

	TAU			Centre-based intervention				Mean Difference	Risk of Bias							
**Study or Subgroup**	**Mean**	**SD**	**Total**	**Mean**	**SD**	**Total**	**Weight**	**IV, Random, 95% CI**	**A**	**B**	**C**	**D**	**E**	**F**	**G**	**H**
Blatt 1965	98.0158	13.7902	38	97	13.71	21	49.0%	1.02 [-6.31 , 8.34]	?		?	?	+	−	?	−
Van de Riet 1972	103.64	13.62	39	86.31	11.96	38	51.0%	17.33 [11.61 , 23.05]	?		−	?	?	?	?	?
**Total (95% CI)**			**77**			**59**	**100.0%**	**9.34 [-6.64 , 25.32]**								

Heterogeneity: $Tau^2 = 121.84$; $Chi^2 = 11.84$, df = 1 (P = 0.0006); $I^2 = 92\%$
Test for overall effect: Z = 1.15 (P = 0.25)
Test for subgroup differences: Not applicable

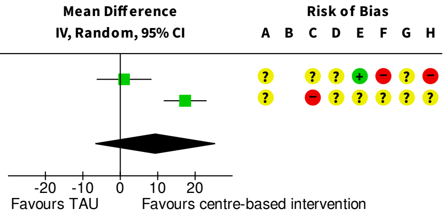

Risk of bias legend
(A) Random sequence generation (selection bias)
(B) Timing and recruitment of clusters
(C) Allocation concealment (selection bias)
(D) Blinding of participants and personnel (performance bias)
(E) Blinding of outcome assessment (detection bias)
(F) Incomplete outcome data (attrition bias)
(G) Selective reporting (reporting bias)
(H) Other bias

Comparison 2: Centre-based early education interventions for improving school readiness versus treatment as usual, Outcome 4: Cognitive development (long-term) (MDs)

Analysis 2.5

			TAU	Centre-based intervention		Std. Mean Difference	Risk of Bias							
**Study or Subgroup**	**Std. Mean Difference**	**SE**	**Total**	**Total**	**Weight**	**IV, Random, 95% CI**	**A**	**B**	**C**	**D**	**E**	**F**	**G**	**H**
**2.5.1 Emotional regulation (short term): ICC 0.12**														
Bierman 2008	0.1417	0.14713278	179	158	12.4%	0.14 [-0.15 , 0.43]	?	−	?	?	+	?	+	?
Blair 2014	0.1925	0.10881076	392	275	21.9%	0.19 [-0.02 , 0.41]	+	+	−	?	−	?	+	+
Courtier 2021	0.4282	0.154	77	98	11.3%	0.43 [0.13 , 0.73]	?	+	?	?	−	?	+	?
Farran 2015	0	0.1132099	467	349	20.3%	0.00 [-0.22 , 0.22]	?	−	?	?	?	?	?	?
Raver 2009	0.1353	0.14601774	238	229	12.5%	0.14 [-0.15 , 0.42]	?	−	?	?	+	?	?	?
Yousafzai 2018	0.2012	0.1097	166	168	21.6%	0.20 [-0.01 , 0.42]	?	−	?	−	+	+	+	+
**Subtotal (95% CI)**			**1519**	**1277**	**100.0%**	**0.17 [0.06 , 0.27]**								
Heterogeneity: $Tau^2 = 0.00$; $Chi^2 = 5.28$, df = 5 (P = 0.38); $I^2 = 5\%$														
Test for overall effect: Z = 3.19 (P = 0.001)														
**2.5.2 Emotional regulation (medium term): ICC 0.12**														
Blair 2014	0.0606	0.10729679	227	331	52.6%	0.06 [-0.15 , 0.27]	+	+	−	?	−	?	+	+
Farran 2015	0	0.11293917	351	459	47.4%	0.00 [-0.22 , 0.22]	?	−	?	?	?	?	?	?
**Subtotal (95% CI)**			**578**	**790**	**100.0%**	**0.03 [-0.12 , 0.18]**								
Heterogeneity: $Tau^2 = 0.00$; $Chi^2 = 0.15$, df = 1 (P = 0.70); $I^2 = 0\%$														
Test for overall effect: Z = 0.41 (P = 0.68)														
**2.5.3 Social skills (short term): ICC 0.12**														
Bierman 2008	0.1999	0.1461916	161	182	8.9%	0.20 [-0.09 , 0.49]	?	−	?	?	+	?	+	?
Farran 2015	0.0908	0.11211849	349	472	9.9%	0.09 [-0.13 , 0.31]	?	−	?	?	?	?	?	?
Hsueh 2014	0.06	0.11392459	220	713	9.9%	0.06 [-0.16 , 0.28]	?	?	?	?	?	+	+	+
Lonigan 2015	0.3034	0.10887587	136	567	10.0%	0.30 [0.09 , 0.52]	?	−	?	?	+	+	+	+
PCER 2008, Bright Beginnings & Creative Curriculum	-0.2795	0.17648171	99	180	7.9%	-0.28 [-0.63 , 0.07]	?	−	?	?	+	+	+	+
PCER 2008, Creative Curriculm	0.6736	0.24804117	81	88	5.9%	0.67 [0.19 , 1.16]	+	−	+	−	+	−	+	+
PCER 2008, Doors to Discovery & Let's Begin	-0.1222	0.16176329	70	182	8.4%	-0.12 [-0.44 , 0.19]	+	−	+	?	+	+	+	?
PCER 2008, Language-Focused Curriculum	-0.4536	0.23483466	91	90	6.3%	-0.45 [-0.91 , 0.01]	+	−	−	?	+	?	+	?
PCER 2008, Literacy Express + DLM + OCR Pre-K	-0.0494	0.188827	88	186	7.5%	-0.05 [-0.42 , 0.32]	+	−	−	?	+	+	+	?
PCER 2008, Project Construct	0.3608	0.20759666	79	183	7.0%	0.36 [-0.05 , 0.77]	−	−	−	?	+	+	+	?
PCER 2008, Ready, Set, Leap!	-0.094	0.16223204	117	138	8.4%	-0.09 [-0.41 , 0.22]	+	−	−	?	+	+	+	?
Yousafzai 2018	0.57	0.112245	168	166	9.9%	0.57 [0.35 , 0.79]	?	−	?	−	+	+	+	+
**Subtotal (95% CI)**			**1659**	**3147**	**100.0%**	**0.11 [-0.05 , 0.28]**								
Heterogeneity: $Tau^2 = 0.06$; $Chi^2 = 41.23$, df = 11 (P < 0.0001); $I^2 = 73\%$														
Test for overall effect: Z = 1.32 (P = 0.19)														
**2.5.4 Social skills (medium term) ICC 0.12**														
Bierman 2008	0.2906	0.1347919	154	167	17.8%	0.29 [0.03 , 0.55]	?	−	?	?	+	?	+	?
Farran 2015	0.0999	0.11298433	352	459	25.3%	0.10 [-0.12 , 0.32]	?	−	?	?	?	?	?	?
PCER 2008, Bright Beginnings & Creative Curriculum	0.0334	0.17763279	96	179	10.2%	0.03 [-0.31 , 0.38]	?	−	?	?	+	+	+	+
PCER 2008, Creative Curriculm	-0.056	0.24900699	44	60	5.2%	-0.06 [-0.54 , 0.43]	+	−	+	−	+	−	+	+
PCER 2008, Doors to Discovery & Let's Begin	-0.0205	0.19356792	42	67	8.6%	-0.02 [-0.40 , 0.36]	+	−	+	?	+	+	+	?
PCER 2008, Language-Focused Curriculum	0.0047	0.2392277	80	74	5.6%	0.00 [-0.46 , 0.47]	+	−	−	?	+	?	+	?
PCER 2008, Literacy Express + DLM + OCR Pre-K	-0.148	0.1966092	74	76	8.4%	-0.15 [-0.53 , 0.24]	+	−	−	?	+	+	+	?
PCER 2008, Project Construct	0.1505	0.21415624	75	79	7.0%	0.15 [-0.27 , 0.57]	−	−	−	?	+	+	+	?
PCER 2008, Ready, Set, Leap!	-0.0431	0.16578711	109	125	11.8%	-0.04 [-0.37 , 0.28]	+	−	−	?	+	+	+	?
**Subtotal (95% CI)**			**1026**	**1286**	**100.0%**	**0.07 [-0.04 , 0.18]**								
Heterogeneity: $Tau^2 = 0.00$; $Chi^2 = 5.18$, df = 8 (P = 0.74); $I^2 = 0\%$														
Test for overall effect: Z = 1.22 (P = 0.22)														
**2.5.5 Social skills (long term) ICC 0.12**														
Farran 2015	0	0.11288992	337	442	57.9%	0.00 [-0.22 , 0.22]	?	−	?	?	?	?	?	?
Van de Riet 1972	0.5093	0.2318	39	38	42.1%	0.51 [0.05 , 0.96]	?		−	?	?	?	?	?
**Subtotal (95% CI)**			**376**	**480**	**100.0%**	**0.21 [-0.28 , 0.71]**								
Heterogeneity: $Tau^2 = 0.10$; $Chi^2 = 3.90$, df = 1 (P = 0.05); $I^2 = 74\%$														
Test for overall effect: Z = 0.85 (P = 0.39)														

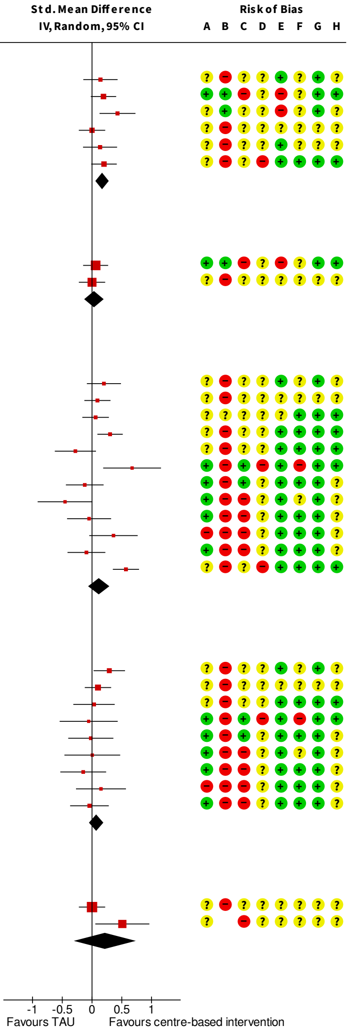

Risk of bias legend
(A) Random sequence generation (selection bias)
(B) Timing and recruitment of clusters
(C) Allocation concealment (selection bias)
(D) Blinding of participants and personnel (performance bias)
(E) Blinding of outcome assessment (detection bias)
(F) Incomplete outcome data (attrition bias)
(G) Selective reporting (reporting bias)
(H) Other bias

Comparison 2: Centre-based early education interventions for improving school readiness versus treatment as usual, Outcome 5: Emotional well-being & social competence (SMDs)

Analysis 2.6

Study or Subgroup	Std. Mean Difference	SE	TAU Total	Centre-based intervention Total	Weight	Std. Mean Difference IV, Random, 95% CI
**2.6.1 Problem behaviours (short term): ICC 0.12**						
Bierman 2008	-0.2226	0.14690957	157	178	13.9%	-0.22 [-0.51 , 0.07]
Blair 2014	-0.1476	0.1084732	274	391	22.6%	-0.15 [-0.36 , 0.07]
Hsueh 2014	-0.04	1.16456251	220	713	0.3%	-0.04 [-2.32 , 2.24]
PCER 2008, Bright Beginnings & Creative Curriculum	0.168	0.17593069	99	180	10.2%	0.17 [-0.18 , 0.51]
PCER 2008, Creative Curriculm	-0.2413	0.21870238	81	89	6.8%	-0.24 [-0.67 , 0.19]
PCER 2008, Doors to Discovery & Let's Begin	-0.1628	0.16187521	70	181	11.8%	-0.16 [-0.48 , 0.15]
PCER 2008, Language-Focused Curriculum	0.4619	0.23494588	91	90	6.0%	0.46 [0.00 , 0.92]
PCER 2008, Literacy Express + DLM + OCR Pre-K	-0.0973	0.18928588	88	186	8.9%	-0.10 [-0.47 , 0.27]
PCER 2008, Project Construct	-0.2388	0.20539412	86	102	7.7%	-0.24 [-0.64 , 0.16]
PCER 2008, Ready, Set, Leap!	-0.0319	0.16116438	121	139	11.9%	-0.03 [-0.35 , 0.28]
**Subtotal (95% CI)**			**1287**	**2249**	**100.0%**	**-0.09 [-0.20 , 0.03]**
Heterogeneity: $Tau^2 = 0.00$; $Chi^2 = 10.10$, df = 9 (P = 0.34); $I^2 = 11\%$						
Test for overall effect: Z = 1.45 (P = 0.15)						
**2.6.2 Problem behaviours (medium term): ICC 0.12**						
Bierman 2008	-0.0193	0.14682776	157	176	15.3%	-0.02 [-0.31 , 0.27]
Blair 2014	-0.032	0.11316812	228	330	25.8%	-0.03 [-0.25 , 0.19]
PCER 2008, Bright Beginnings & Creative Curriculum	0.0965	0.17580937	96	180	10.7%	0.10 [-0.25 , 0.44]
PCER 2008, Creative Curriculm	0.1194	0.24847868	45	59	5.4%	0.12 [-0.37 , 0.61]
PCER 2008, Doors to Discovery & Let's Begin	0	0.19367132	42	100	8.8%	0.00 [-0.38 , 0.38]
PCER 2008, Language-Focused Curriculum	0.0673	0.23797758	81	77	5.8%	0.07 [-0.40 , 0.53]
PCER 2008, Literacy Express + DLM + OCR Pre-K	-0.0146	0.19424885	78	157	8.8%	-0.01 [-0.40 , 0.37]
PCER 2008, Project Construct	0.0724	0.21296461	76	81	7.3%	0.07 [-0.35 , 0.49]
PCER 2008, Ready, Set, Leap!	0.0852	0.16521813	113	123	12.1%	0.09 [-0.24 , 0.41]
**Subtotal (95% CI)**			**916**	**1283**	**100.0%**	**0.02 [-0.09 , 0.14]**
Heterogeneity: $Tau^2 = 0.00$; $Chi^2 = 0.93$, df = 8 (P = 1.00); $I^2 = 0\%$						
Test for overall effect: Z = 0.41 (P = 0.68)						

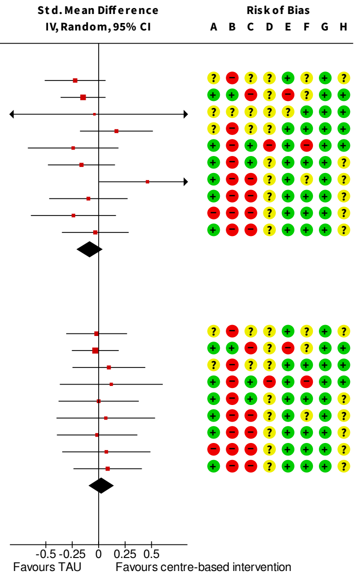

Risk of bias legend
(A) Random sequence generation (selection bias)
(B) Timing and recruitment of clusters
(C) Allocation concealment (selection bias)
(D) Blinding of participants and personnel (performance bias)
(E) Blinding of outcome assessment (detection bias)
(F) Incomplete outcome data (attrition bias)
(G) Selective reporting (reporting bias)
(H) Other bias

Comparison 2: Centre-based early education interventions for improving school readiness versus treatment as usual, Outcome 6: Emotional well-being & social competence: problem behaviours (SMDs)

Analysis 2.7

Study or Subgroup	TAU Mean	TAU SD	TAU Total	Centre-based intervention Mean	Centre-based intervention SD	Centre-based intervention Total	Mean Difference IV, Fixed, 95% CI
**2.7.1 Fine motor skills (short term)**							
Yousafzai 2018	8.8	8.87	166	8	8.93	168	0.80 [-1.11 , 2.71]
**2.7.2 Gross motor skills (short term)**							
Yousafzai 2018	6.8	10.19	166	5.2	11.24	168	1.60 [-0.70 , 3.90]

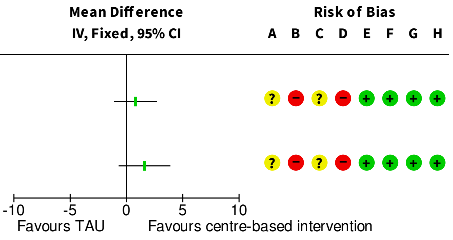

Risk of bias legend
(A) Random sequence generation (selection bias)
(B) Timing and recruitment of clusters
(C) Allocation concealment (selection bias)
(D) Blinding of participants and personnel (performance bias)
(E) Blinding of outcome assessment (detection bias)
(F) Incomplete outcome data (attrition bias)
(G) Selective reporting (reporting bias)
(H) Other bias

Comparison 2: Centre-based early education interventions for improving school readiness versus treatment as usual, Outcome 7: Physical development (short-term)

Analysis 2.8

Study or Subgroup	TAU Mean	TAU SD	TAU Total	Centre-based intervention Mean	Centre-based intervention SD	Centre-based intervention Total	Mean Difference IV, Fixed, 95% CI
Van de Riet 1972	4.74	2.27	39	7.21	3.8	39	-2.47 [-3.86 , -1.08]

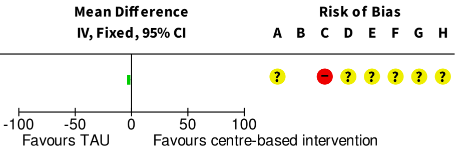

Risk of bias legend
(A) Random sequence generation (selection bias)
(B) Timing and recruitment of clusters
(C) Allocation concealment (selection bias)
(D) Blinding of participants and personnel (performance bias)
(E) Blinding of outcome assessment (detection bias)
(F) Incomplete outcome data (attrition bias)
(G) Selective reporting (reporting bias)
(H) Other bias

Comparison 2: Centre-based early education interventions for improving school readiness versus treatment as usual, Outcome 8: Physical development: perceptual motor skills (long-term)

Analysis 3.1

Study or Subgroup	Std. Mean Difference	SE	Weight	Std. Mean Difference IV, Random, 95% CI	Std. Mean Difference IV, Random, 95% CI
**3.1.1 Mathmatics (short term): ICC 0.12**					
Barnes 2016	0.1544	0.10741809	10.7%	0.15 [-0.06 , 0.36]	
Blair 2014	0.1497	0.10715822	10.8%	0.15 [-0.06 , 0.36]	
Courtier 2021	0.4037	0.15140849	5.4%	0.40 [0.11 , 0.70]	
Farran 2015	0.064205	0.1122356	9.8%	0.06 [-0.16 , 0.28]	
Hsueh 2014	0.13	0.14	6.3%	0.13 [-0.14 , 0.40]	
Lillard 2017	0.2015	0.1732	4.1%	0.20 [-0.14 , 0.54]	
Lonigan 2015	0.3114	0.10915542	10.4%	0.31 [0.10 , 0.53]	
PCER 2008, Bright Beginnings & Creative Curriculum	0.18645159	0.17540559	4.0%	0.19 [-0.16 , 0.53]	
PCER 2008, Creative Curriculm	0.14641796	0.21854168	2.6%	0.15 [-0.28 , 0.57]	
PCER 2008, Doors to Discovery & Let's Begin	0.00937274	0.15097153	5.4%	0.01 [-0.29 , 0.31]	
PCER 2008, Language-Focused Curriculum	0.21695665	0.23137951	2.3%	0.22 [-0.24 , 0.67]	
PCER 2008, Literacy Express + DLM + OCR Pre-K	0.31880454	0.1898605	3.4%	0.32 [-0.05 , 0.69]	
PCER 2008, Project Construct	0.26388311	0.20359939	3.0%	0.26 [-0.14 , 0.66]	
PCER 2008, Ready, Set, Leap!	0.04460665	0.16218705	4.7%	0.04 [-0.27 , 0.36]	
Raver 2009	0.01986688	0.14581054	5.8%	0.02 [-0.27 , 0.31]	
Warden 1988	0.9491685	0.335392061	1.1%	0.95 [0.29 , 1.61]	
Yousafzai 2018	0.2872	0.11	10.2%	0.29 [0.07 , 0.50]	
**Subtotal (95% CI)**			**100.0%**	**0.19 [0.12 , 0.26]**	
Heterogeneity: Tau² = 0.00; Chi² = 15.07, df = 16 (P = 0.52); I² = 0%					
Test for overall effect: Z = 5.35 (P < 0.00001)					

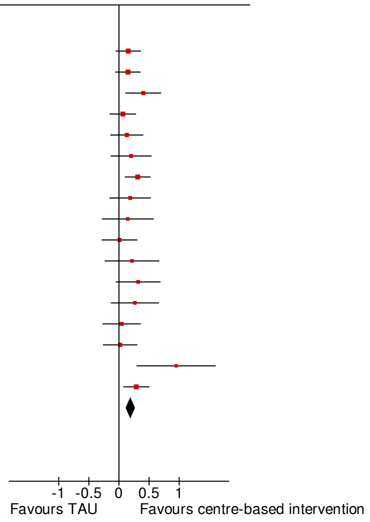

Comparison 3: Funnel plot data, Outcome 1: Academic achievement: maths (short term)

Analysis 3.2

Study or Subgroup	Mean Difference	SE	Weight	Mean Difference IV, Random, 95% CI	Mean Difference IV, Random, 95% CI
**3.2.1 Mathematics (medium term): ICC 0.12**					
Blair 2014	0.105786	0.10881207	18.2%	0.11 [-0.11 , 0.32]	
Farran 2015	-0.031682	0.11210274	17.1%	-0.03 [-0.25 , 0.19]	
Lillard 2017	0.3936	0.1751	7.0%	0.39 [0.05 , 0.74]	
PCER 2008, Bright Beginnings & Creative Curriculum	0.23591166	0.17406875	7.1%	0.24 [-0.11 , 0.58]	
PCER 2008, Creative Curriculm	0.149977	0.22219427	4.4%	0.15 [-0.29 , 0.59]	
PCER 2008, Doors to Discovery & Let's Begin	-0.00532987	0.09584515	23.5%	-0.01 [-0.19 , 0.18]	
PCER 2008, Language-Focused Curriculum	0.161989	0.23128549	4.0%	0.16 [-0.29 , 0.62]	
PCER 2008, Literacy Express + DLM + OCR Pre-K	0.35520064	0.19571242	5.6%	0.36 [-0.03 , 0.74]	
PCER 2008, Project Construct	0.101013	0.20751373	5.0%	0.10 [-0.31 , 0.51]	
PCER 2008, Ready, Set, Leap!	0.020163	0.16392552	8.0%	0.02 [-0.30 , 0.34]	
**Subtotal (95% CI)**			**100.0%**	**0.10 [0.01 , 0.19]**	
Heterogeneity: Tau² = 0.00; Chi² = 8.07, df = 9 (P = 0.53); I² = 0%					
Test for overall effect: Z = 2.08 (P = 0.04)					

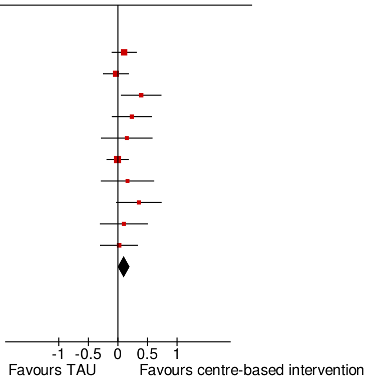

Comparison 3: Funnel plot data, Outcome 2: Academic achievement: maths (medium term)

Analysis 3.3

Study or Subgroup	Std. Mean Difference	SE	Weight	Std. Mean Difference IV, Random, 95% CI	Std. Mean Difference IV, Random, 95% CI
**3.3.1 Reading (short term): ICC 0.12**					
Bierman 2008	0.1843	0.14672819	7.2%	0.18 [-0.10 , 0.47]	
Blair 2014	0.0461	0.09892949	8.9%	0.05 [-0.15 , 0.24]	
Courtier 2021	0.6989	0.15446029	7.0%	0.70 [0.40 , 1.00]	
Farran 2015	-0.0265	0.11221186	8.4%	-0.03 [-0.25 , 0.19]	
Lillard 2017	-0.155155	0.173061	6.4%	-0.16 [-0.49 , 0.18]	
Lonigan 2015	0.2707	0.109353965	8.5%	0.27 [0.06 , 0.49]	
PCER 2008, Bright Beginnings & Creative Curriculum	0.16917127	0.17534421	6.3%	0.17 [-0.17 , 0.51]	
PCER 2008, Creative Curriculm	-0.18941694	0.21852812	5.1%	-0.19 [-0.62 , 0.24]	
PCER 2008, Doors to Discovery & Let's Begin	0.04476987	0.15141959	7.1%	0.04 [-0.25 , 0.34]	
PCER 2008, Language-Focused Curriculum	0.21867809	0.23143037	4.8%	0.22 [-0.23 , 0.67]	
PCER 2008, Literacy Express + DLM + OCR Pre-K	0.50811792	0.18968814	5.9%	0.51 [0.14 , 0.88]	
PCER 2008, Project Construct	-0.05818012	0.20141906	5.6%	-0.06 [-0.45 , 0.34]	
PCER 2008, Ready, Set, Leap!	0.07758538	0.1618606	6.7%	0.08 [-0.24 , 0.39]	
Raver 2009	0.207387	0.14620203	7.3%	0.21 [-0.08 , 0.49]	
Warden 1988	0.94312979	0.23632	4.7%	0.94 [0.48 , 1.41]	
**Subtotal (95% CI)**			**100.0%**	**0.18 [0.05 , 0.32]**	
Heterogeneity: Tau² = 0.04; Chi² = 39.71, df = 14 (P = 0.0003); I² = 65%					
Test for overall effect: Z = 2.69 (P = 0.007)					

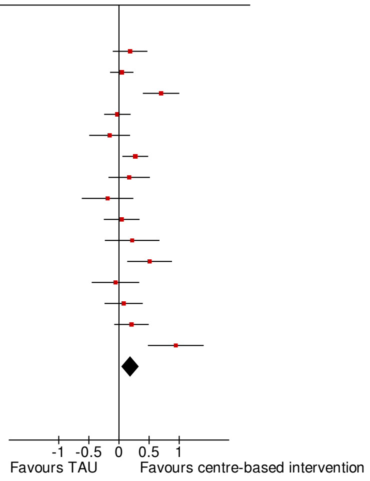

Comparison 3: Funnel plot data, Outcome 3: Academic achievement: reading (short term)

Analysis 3.4

Study or Subgroup	Mean Difference	SE	Weight	Mean Difference IV, Random, 95% CI
**3.4.1 Reading (medium): ICC 0.12**				
Bierman 2008	0	0.14682776	7.8%	0.00 [-0.29 , 0.29]
Blair 2014	0.1006	0.10870446	14.2%	0.10 [-0.11 , 0.31]
Farran 2015	-0.051495	0.12441792	10.8%	-0.05 [-0.30 , 0.19]
Lillard 2017	0.548943	0.176761	5.4%	0.55 [0.20 , 0.90]
PCER 2008, Bright Beginnings & Creative Curriculum	-0.0060953	0.10750571	14.5%	-0.01 [-0.22 , 0.20]
PCER 2008, Creative Curriculm	0.04626061	0.22067046	3.4%	0.05 [-0.39 , 0.48]
PCER 2008, Doors to Discovery & Let's Begin	0.00275912	0.09398137	19.0%	0.00 [-0.18 , 0.19]
PCER 2008, Language-Focused Curriculum	0.05213937	0.23049772	3.2%	0.05 [-0.40 , 0.50]
PCER 2008, Literacy Express + DLM + OCR Pre-K	0.07897031	0.11914802	11.8%	0.08 [-0.15 , 0.31]
PCER 2008, Project Construct	0.02467423	0.20622743	3.9%	0.02 [-0.38 , 0.43]
PCER 2008, Ready, Set, Leap!	0.08952074	0.16568166	6.1%	0.09 [-0.24 , 0.41]
**Subtotal (95% CI)**			**100.0%**	**0.06 [-0.02 , 0.14]**
Heterogeneity: $Tau^2 = 0.00$; $Chi^2 = 9.60$, df = 10 (P = 0.48); $I^2 = 0\%$				
Test for overall effect: Z = 1.39 (P = 0.17)				

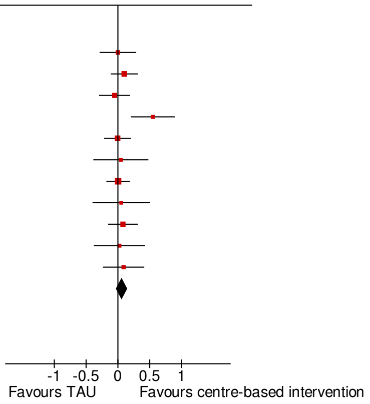

Comparison 3: Funnel plot data, Outcome 4: Academic achievement: reading (medium term)

Analysis 3.5

Study or Subgroup	Std. Mean Difference	SE	Weight	Std. Mean Difference IV, Random, 95% CI
**3.5.1 Vocabulary (short term): ICC 0.12**				
Bierman 2008	0.15395934	0.14685113	7.0%	0.15 [-0.13 , 0.44]
Blair 2014	0.1553	0.10652362	13.3%	0.16 [-0.05 , 0.36]
Blatt 1965	0.33790451	0.273804574	2.0%	0.34 [-0.20 , 0.87]
Courtier 2021	0.0105	0.12277984	10.0%	0.01 [-0.23 , 0.25]
Farran 2015	-0.0217	0.11223719	12.0%	-0.02 [-0.24 , 0.20]
Lillard 2017	0.2852	0.1757	4.9%	0.29 [-0.06 , 0.63]
Lonigan 2015	0.3518	0.10954919	12.6%	0.35 [0.14 , 0.57]
PCER 2008, Bright Beginnings & Creative Curriculum	0.22683666	0.17571767	4.9%	0.23 [-0.12 , 0.57]
PCER 2008, Creative Curriculm	0.0428036	0.21944133	3.1%	0.04 [-0.39 , 0.47]
PCER 2008, Doors to Discovery & Let's Begin	0.15424329	0.15196256	6.5%	0.15 [-0.14 , 0.45]
PCER 2008, Language-Focused Curriculum	0.00605458	0.23025066	2.8%	0.01 [-0.45 , 0.46]
PCER 2008, Literacy Express + DLM + OCR Pre-K	0.43208889	0.1886847	4.2%	0.43 [0.06 , 0.80]
PCER 2008, Project Construct	0.06741315	0.20055858	3.8%	0.07 [-0.33 , 0.46]
PCER 2008, Ready, Set, Leap!	0.14348454	0.16136346	5.8%	0.14 [-0.17 , 0.46]
Raver 2009	0.12452377	0.1459493	7.1%	0.12 [-0.16 , 0.41]
**Subtotal (95% CI)**			**100.0%**	**0.16 [0.08 , 0.23]**
Heterogeneity: $Tau^2 = 0.00$; $Chi^2 = 11.33$, df = 14 (P = 0.66); $I^2 = 0\%$				
Test for overall effect: Z = 4.00 (P < 0.0001)				

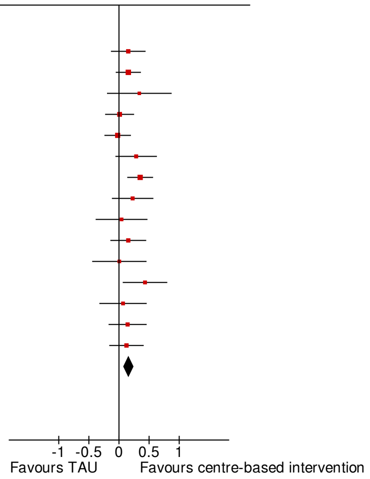

Comparison 3: Funnel plot data, Outcome 5: Academic achievement: vocabulary (short term)

Analysis 3.6

Study or Subgroup	Std. Mean Difference	SE	Weight	Std. Mean Difference IV, Random, 95% CI
**3.6.1 Vocabulary (medium term): ICC 0.12**				
Bierman 2008	0.0899	0.14682776	10.2%	0.09 [-0.20 , 0.38]
Blair 2014	0.205438	0.10881488	18.6%	0.21 [-0.01 , 0.42]
Blatt 1965	0.07042843	0.271987429	3.0%	0.07 [-0.46 , 0.60]
Farran 2015	0.0266	0.11210274	17.5%	0.03 [-0.19 , 0.25]
Lillard 2017	0.0214	0.1734	7.3%	0.02 [-0.32 , 0.36]
PCER 2008, Bright Beginnings & Creative Curriculum	0.13780148	0.17401044	7.3%	0.14 [-0.20 , 0.48]
PCER 2008, Creative Curriculm	0.22550166	0.22159826	4.5%	0.23 [-0.21 , 0.66]
PCER 2008, Doors to Discovery & Let's Begin	0.12985523	0.16239462	8.4%	0.13 [-0.19 , 0.45]
PCER 2008, Language-Focused Curriculum	-0.04665082	0.2307593	4.1%	-0.05 [-0.50 , 0.41]
PCER 2008, Literacy Express + DLM + OCR Pre-K	0.46511628	0.19667268	5.7%	0.47 [0.08 , 0.85]
PCER 2008, Project Construct	0.21388153	0.206661	5.2%	0.21 [-0.19 , 0.62]
PCER 2008, Ready, Set, Leap!	-0.01896789	0.16460867	8.1%	-0.02 [-0.34 , 0.30]
**Subtotal (95% CI)**			**100.0%**	**0.12 [0.03 , 0.21]**
Heterogeneity: $Tau^2 = 0.00$; $Chi^2 = 6.47$, df = 11 (P = 0.84); $I^2 = 0\%$				
Test for overall effect: Z = 2.57 (P = 0.01)				

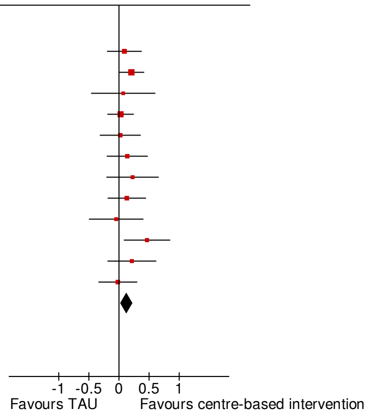

Comparison 3: Funnel plot data, Outcome 6: Academic achievement: vocabulary (medium term)

Analysis 3.7

Study or Subgroup	Std. Mean Difference	SE	Weight	Std. Mean Difference IV, Random, 95% CI
**3.7.1 Problem behaviours (short term): ICC 0.12**				
Bierman 2008	-0.2226	0.14690957	13.9%	-0.22 [-0.51 , 0.07]
Blair 2014	-0.1476	0.1084732	22.6%	-0.15 [-0.36 , 0.07]
Hsueh 2014	-0.04	1.16456251	0.3%	-0.04 [-2.32 , 2.24]
PCER 2008, Bright Beginnings & Creative Curriculum	0.168	0.17593069	10.2%	0.17 [-0.18 , 0.51]
PCER 2008, Creative Curriculm	-0.2413	0.21870238	6.8%	-0.24 [-0.67 , 0.19]
PCER 2008, Doors to Discovery & Let's Begin	-0.1628	0.16187521	11.8%	-0.16 [-0.48 , 0.15]
PCER 2008, Language-Focused Curriculum	0.4619	0.23494588	6.0%	0.46 [0.00 , 0.92]
PCER 2008, Literacy Express + DLM + OCR Pre-K	-0.0973	0.18928588	8.9%	-0.10 [-0.47 , 0.27]
PCER 2008, Project Construct	-0.2388	0.20539412	7.7%	-0.24 [-0.64 , 0.16]
PCER 2008, Ready, Set, Leap!	-0.0319	0.16116438	11.9%	-0.03 [-0.35 , 0.28]
**Subtotal (95% CI)**			**100.0%**	**-0.09 [-0.20 , 0.03]**

Heterogeneity: $Tau^2 = 0.00$; $Chi^2 = 10.10$, $df = 9$ ($P = 0.34$); $I^2 = 11\%$
Test for overall effect: $Z = 1.45$ ($P = 0.15$)

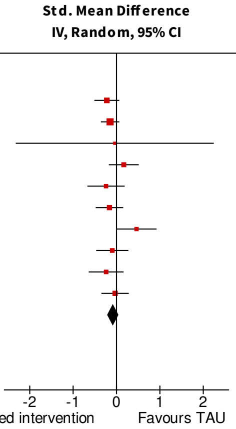

Comparison 3: Funnel plot data, Outcome 7: Emotional well-being & social competence: problem behaviours (short term)

Analysis 3.8

Study or Subgroup	Std. Mean Difference	SE	Weight	Std. Mean Difference IV, Random, 95% CI
**3.8.1 Social skills (short term): ICC 0.12**				
Bierman 2008	0.1999	0.1461916	8.9%	0.20 [-0.09 , 0.49]
Farran 2015	0.0908	0.11211849	9.9%	0.09 [-0.13 , 0.31]
Hsueh 2014	0.06	0.11392459	9.9%	0.06 [-0.16 , 0.28]
Lonigan 2015	0.3034	0.10887587	10.0%	0.30 [0.09 , 0.52]
PCER 2008, Bright Beginnings & Creative Curriculum	-0.2795	0.17648171	7.9%	-0.28 [-0.63 , 0.07]
PCER 2008, Creative Curriculm	0.6736	0.24804117	5.9%	0.67 [0.19 , 1.16]
PCER 2008, Doors to Discovery & Let's Begin	-0.1222	0.16176329	8.4%	-0.12 [-0.44 , 0.19]
PCER 2008, Language-Focused Curriculum	-0.4536	0.23483466	6.3%	-0.45 [-0.91 , 0.01]
PCER 2008, Literacy Express + DLM + OCR Pre-K	-0.0494	0.188827	7.5%	-0.05 [-0.42 , 0.32]
PCER 2008, Project Construct	0.3608	0.20759666	7.0%	0.36 [-0.05 , 0.77]
PCER 2008, Ready, Set, Leap!	-0.094	0.16223204	8.4%	-0.09 [-0.41 , 0.22]
Yousafzai 2018	0.57	0.112245	9.9%	0.57 [0.35 , 0.79]
**Subtotal (95% CI)**			**100.0%**	**0.11 [-0.05 , 0.28]**

Heterogeneity: $Tau^2 = 0.06$; $Chi^2 = 41.23$, $df = 11$ ($P < 0.0001$); $I^2 = 73\%$
Test for overall effect: $Z = 1.32$ ($P = 0.19$)

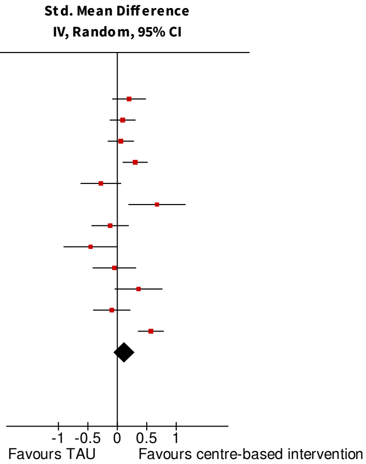

Comparison 3: Funnel plot data, Outcome 8: Emotional well-being & social competence: social skills (short term)
